# Sucralose Consumption Ablates Cancer Immunotherapy Response through Microbiome Disruption

**DOI:** 10.1158/2159-8290.CD-25-0247

**Published:** 2025-07-30

**Authors:** Kristin M. Morder, Madison Nguyen, Drew N. Wilfahrt, Zakaria Larbi Dahmani, Ansen B.P. Burr, Bingxian Xie, Michael Morikone, Hector Nieves-Rosado, William G. Gunn, Drew E. Hurd, Hong Wang, Steven J. Mullett, Kaitlin Bossong, Stacy L. Gelhaus, Dhivyaa Rajasundaram, Lawrence P. Kane, Greg M. Delgoffe, Jishnu Das, Diwakar Davar, Abigail E. Overacre-Delgoffe

**Affiliations:** 1 UPMC Hillman Cancer Center, Pittsburgh, Pennsylvania.; 2 Department of Immunology, University of Pittsburgh, Pittsburgh, Pennsylvania.; 3 Center for Systems Immunology, Pittsburgh, Pennsylvania.; 4 Department of Medicine, University of Pittsburgh, Pittsburgh, Pennsylvania.; 5 Bioinformatics Core, Department of Pediatrics, University of Pittsburgh, Pittsburgh, Pennsylvania.; 6 Biostatistics Facility, UPMC Hillman Cancer Center, University of Pittsburgh, Pittsburgh, Pennsylvania.; 7 Department of Pharmacology and Chemical Biology, University of Pittsburgh, Pittsburgh, Pennsylvania.; 8 Tumor Microenvironment Center, Pittsburgh, Pennsylvania.

## Abstract

Gut microbiota composition is directly associated with response to immunotherapies in cancer. The impact of diet on the gut microbiota and downstream immune responses to cancer remains unclear. In this study, we show that consumption of a common nonnutritive sweetener, sucralose, modifies microbiome composition, restricts T-cell metabolism and function, and limits immunotherapy response in preclinical models of cancer and patients with advanced cancer treated with anti–PD-1–based immune checkpoint inhibitors. Sucralose consumption is associated with a reduction in microbiota-accessible arginine, and amino acid supplementation or fecal microbiome transfer from anti–PD-1 responder mice completely restores T-cell function and immunotherapy response. Overall, sucralose consumption destabilizes the gut microbiota, resulting in compromised T-cell function and ablated immune checkpoint inhibitor response in cancer.

**Significance:**

This study highlights an unappreciated role of sucralose in reducing immunotherapy efficacy in both mouse models and samples from patients with cancer through shifts in the microbiome and arginine degradation that lead to T-cell exhaustion. T-cell function and immunotherapy responses are restored through amino acid supplementation.

*See related commentary by Chandra et al., p. 2196*

## Teaser

Studying preclinical models and human patients with cancer treated with immune checkpoint blockade reveals that sucralose alters gut microbiota, impeding T-cell function and reducing immunotherapeutic efficacy.

## Introduction

Immune checkpoint inhibitors (ICI) targeting negative regulatory checkpoints, including PD-1 and cytotoxic T-lymphocyte–associated protein 4, produce durable responses in many cancers (1). In multiple cancers including melanoma and non–small cell lung cancer (NSCLC), ICIs singly or in combination with other agents, including chemotherapy or tyrosine kinase inhibitors, produce high objective response rates (ORR) and durable progression-free survival (PFS) and overall survival (OS; ref. 2). However, the majority of ICI-treated patients fail to respond durably, and biomarkers are needed to tailor the management in these patients. Multiple predictive biomarkers of ICI response have been described, including CD8^+^ tumor-infiltrating hymphocytes (3, 4), PD-L1 expression (5, 6), tumor mutation burden (TMB; refs. 7, 8), and HLA class I haplotype (9). Recently, the gut microbiome has emerged as a major tumor-extrinsic regulator of response to multiple immunotherapies in human patients with cancer, including ICIs and anti-CD19 chimeric antigen receptor T-cell therapy (10–14). The gut microbiota can be shaped and altered by external factors including, but not limited to, host diet, antibiotic or probiotic use, and chemical exposures (12, 15–19).

Over the past several decades, humans have experienced major dietary changes, particularly a reduction in fiber intake in Westernized populations, which has paralleled general loss in the diversity of gut microbiota in Westernized populations compared with non-industrialized populations (20–22). These dietary changes have fueled an increase in obesity in industrialized countries (23). Nonnutritive sweeteners (NNS) were developed as an alternative to sugar, and NNS consumption has historically been considered safe and beneficial owing to their low caloric content, although the data underlying this are scarce. NNS intake is prevalent in the general population, both in lean and obese individuals alike, with 24% to 37% of US adults reporting some NNS intake in dietary recall surveys (20). Two of the most used NNS, sucralose and saccharin, have been shown to significantly change the gut microbiome in mice and humans (24, 25). Moreover, sucralose-induced microbial shifts are sufficient to negatively affect overall host health, including driving glucose intolerance (24–26). Furthermore, although high doses of sucralose directly impair T-cell proliferation and effector function in preclinical models of autoimmune disease (27), the link between NNS intake in general, and sucralose intake in particular, with gut microbiota and immunotherapy outcomes in cancer is not well understood.

In this study, we observed that sucralose intake negatively affected anti–PD-1 ICI efficacy in cancer in both mice and humans. In patients with ICI-treated advanced melanoma, advanced NSCLC, and high-risk resectable melanoma, food frequency questionnaire (FFQ)–determined, weight-normalized higher sucralose intake was associated with lower response and poorer survival compared with lower or no sucralose intake. Tumor-bearing mice given sucralose had poor responses to anti–PD-1 blockade, and this effect required sucralose-driven changes to the gut microbiota. Overall, these findings suggest that high intake of sucralose contributes to ICI nonresponse in a gut microbiota–dependent, T cell–centric fashion across preclinical models and patients with cancer.

## Results

### Sucralose Ablates Immunotherapeutic Response

**Figure 1.**
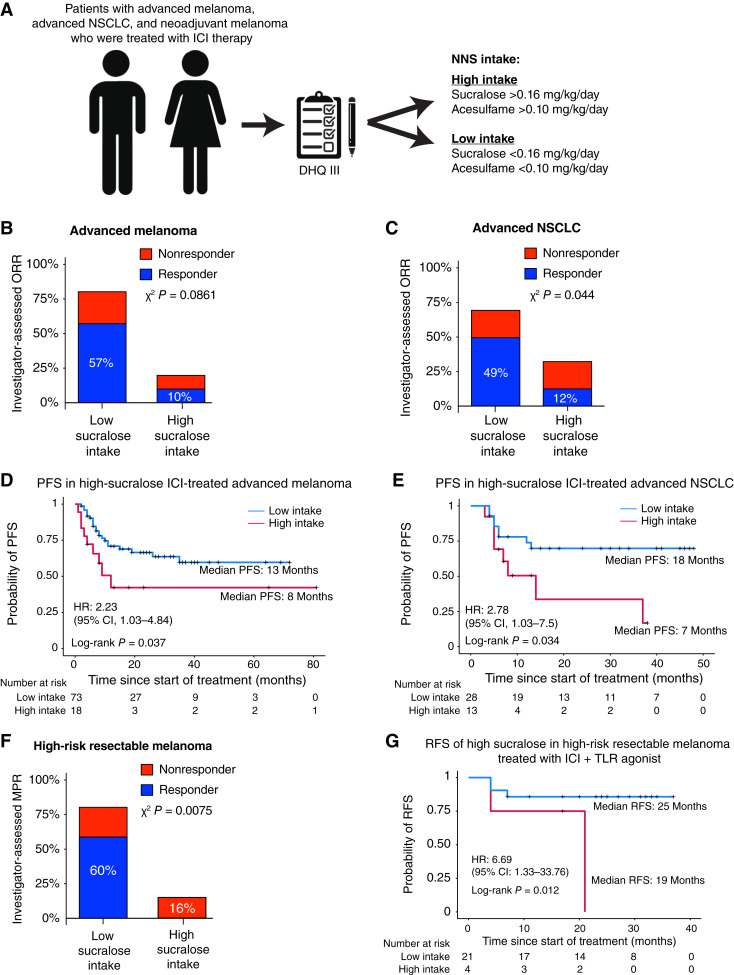
NNS intake is associated with poor response to ICI in advanced melanoma, advanced NSCLC, and neoadjuvant melanoma. **A,** Patients with advanced melanoma, advanced NSCLC, and neoadjuvant melanoma pending receipt of ICI therapy completed web-based semi-quantitative FFQ DHQ III. Response to therapy was evaluated using investigator-assessed ORR using RECIST v1.1 or pathologist-assessed immune-related pathologic response criteria, along with time-to-event analyses including PFS (advanced melanoma or NSCLC) or RFS (neoadjuvant melanoma). RFS/PFS were evaluated every 3 months, and relapse/progression was defined based on radiographic and/or clinical relapse/progression at each treatment visit (every 3–4 weeks). **A,** Patients were dichotomized into high- and low-intake groups based on cutpointr-determined endpoints. **B** and **C,** Proportion of investigator-assessed ORR in either melanoma (**B**) or NSCLC (**C**) cohorts. *χ*^2^*P* values comparing responder ORR between high vs. low intake are shown. **D** and **E,** Kaplan–Meier plots of PFS probability of patients with ICI-treated melanoma (**D**) and NSCLC (**E**) based on dichotomized sucralose intake levels by a two-sided log-rank test are shown. The number of people at risk in either group (high vs. low intake) is shown below each panel. Vertical ticks show censored data. **F,** Proportion of pathologist-assessed MPR (defined as 0%–10% residual viable tumor) between high and low sucralose intake in patients with high-risk resectable melanoma treated with nivolumab and TLR9 agonist vidutolimod. *χ*^2^*P* values comparing responder MPR between high and low intake are shown. **G,** Kaplan–Meier plots of RFS probability of patients with neoadjuvant nivolumab-/vidutolimod-treated melanoma based on dichotomized sucralose intake levels by a two-sided log-rank test are shown. The number of people at risk in either group (high vs. low intake) is shown below each panel. Vertical ticks show censored data.

To evaluate whether sucralose intake affected the efficacy of anti–PD-1 ICI, we evaluated three separate cohorts of ICI-treated patients spanning a spectrum of histologies and stages: ICI-treated advanced melanoma, ICI-treated advanced NSCLC, and high-risk resectable melanoma treated with neoadjuvant ICI and the TLR9 agonist vidutolimod (Supplementary Fig. S1A). Patients with advanced metastatic cutaneous melanoma or NSCLC who were scheduled to be treated with systemic anti–PD-1–based immunotherapy or chemoimmunotherapy were enrolled in a prospective research registry [Hillman Cancer Center (HCC) 20-019] that included the administration of a validated, web-based, semi-quantitative Diet History Questionnaire III (DHQ III) FFQ that quantified self-reported dietary intake (Supplementary Fig. S1A; Supplementary Appendix S1 and S2). Briefly, patients with advanced melanoma or NSCLC who had received systemic anti–PD-1–based immunotherapy or chemoimmunotherapy had undergone dietary history evaluation using DHQ III, had received treatment for at least 3 months, had at least one post-treatment imaging study evaluable for response, had adequate follow-up time (6 months), and had provided informed consent were included (Fig. 1A; Supplementary Fig. S1A). Separately, we evaluated 25 patients with high-risk resectable melanoma who received neoadjuvant anti–PD-1 nivolumab along with intratumoral TLR9 agonist vidutolimod, the primary results of which have previously been reported (Supplementary Fig. S1A; Supplementary Appendix S3 and S4; refs. 28). Overall, we included 91 patients with advanced melanoma, 41 patients with advanced NSCLC, and 25 patients with high-risk resectable melanoma (Supplementary Fig. S1A). In all cases, DHQ III FFQ was administered prior to initiation of systemic therapy in person by a trained provider.

To evaluate the impact of pretreatment NNS intake upon ICI efficacy, we determined the weight-normalized intake levels of sucralose and other NNS (aspartame, acesulfame, and saccharin) relative to US FDA acceptable daily intake (ADI) levels and evaluated the impact of sucralose intake upon ICI treatment outcomes, including investigator-assessed ORR and PFS (advanced melanoma and NSCLC) or major pathologic response (MPR) and relapse-free survival (RFS; high-risk resectable melanoma; Supplementary Fig. S1B; Supplementary Tables S1–S4). No significant demographic differences were observed between groups, and details about baseline demographics, concomitant medication and dietary intake, and treatment exposures are summarized in Supplementary Fig. S1C.

Daily NNS intake was dichotomized using a *cutpointr*-defined cutpoint (sucralose, 0.16 mg/kg/day; acesulfame, 0.10 mg/kg/day) across cohorts (Fig. 1A; Supplementary Figs S2A–S2C; ref. 29). We observed that a high daily intake of sucralose (>0.16 mg/kg/day) was associated with a trend toward lower ORR in ICI-treated melanoma (Fig. 1B) and a significantly lower ORR in ICI-treated NSCLC (Fig. 1C). Commensurately, high sucralose intake (>0.16 mg/kg/day) was significantly associated with poorer PFS in both ICI-treated advanced cutaneous melanoma (median 13.0 vs. 8.0 months, log-rank *P =* 0.037) and NSCLC (median 18.0 vs. 7.0 months, log-rank *P =* 0.034; Fig. 1D and E). High sucralose intake was independently associated with low probability of ORR in ICI-treated advanced cutaneous melanoma [OR = 0.28; 95% confidence interval (CI), 0.085–0.924; *P* = 0.0366] in a multivariate model after correcting for potential confounders (Supplementary Table S1). High sucralose intake was independently predictive of poorer PFS in ICI-treated melanoma in both univariate (HR = 2.21; 95% CI, 1.02–4.79; *P* = 0.0449) and multivariate (HR = 2.91; 95% CI, 1.22–6.96; *P* = 0.0164) models (Supplementary Table S4A). High sucralose intake had a similarly adverse impact upon ORR (Supplementary Table S2) and PFS (Fig. 1B and C; Supplementary Table S4B) in ICI-treated NSCLC. Additionally, in an independent cohort of patients with high-risk resectable melanoma treated with anti–PD-1 and a TLR9 agonist, high sucralose intake was associated with a significantly lower probability of MPR (Fig. 1F) and lower RFS (median 25.0 vs. 19.0 months, log-rank *P* = 0.012; Fig. 1G; Supplementary Tables S3 and S4C).

High daily intake of acesulfame (>0.10 mg/kg/day) had a similar trend toward lower ORR and poorer PFS in ICI-treated melanoma and NSCLC, as well as lower MPR and RFS in high-risk resectable melanoma treated with ICI and TLR9 agonist (Supplementary Figs. S2D-L). There was no significant effect of high intake of other NNS evaluated, including aspartame and saccharin, upon ORR, PFS, or RFS (Supplementary Figs. S3A-L), except between aspartame intake and reduced MPR in high-risk resectable melanoma (Supplementary Fig. S3C).

**Figure 2.**
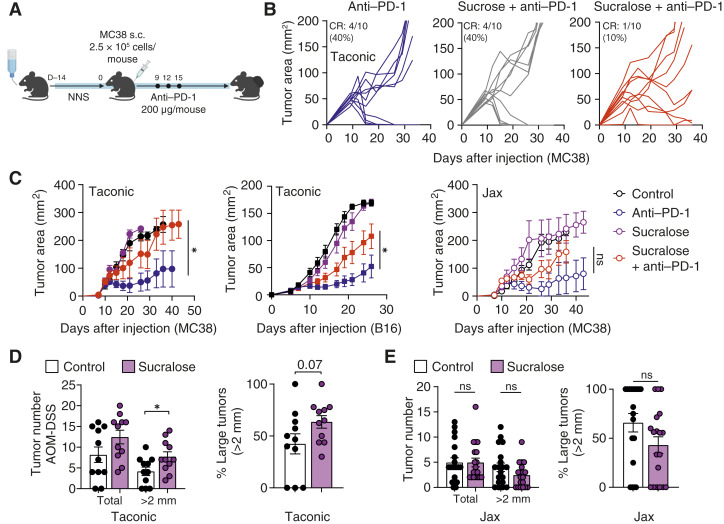
Sucralose ablates immunotherapeutic responses. C57BL/6 mice from Taconic consumed sucralose in the drinking water (0.09 mg/mL) for 2 weeks prior to tumor injection and for the duration of the experiment. Mice were injected with 2.5 × 10^5^ MC38 cells subcutaneously and treated with 200 µg anti–PD-1 on days 9, 12, and 15. Tumor area was measured every 3 days until endpoint. **A,** Experimental schematic. **B,** Tumor growth curves of mice consuming sucrose or sucralose in the drinking water during treatment with anti–PD-1. CR, complete response. **C,** Tumor growth curves in MC38 subcutaneous (circles) or B16 intradermal (squares) treated with anti–PD-1. Mice were sourced from either The Jackson Laboratory (Jax, open circles) or Taconic Biosciences (Taconic, closed circles). Mice were removed from study either when tumors reached 2 cm in either direction or if there was unresolved ulceration. Mean tumor growth lines halt once all mice in a treatment group were removed. **D** and **E,** C57BL/6 mice from either Taconic (**D**) or Jackson (**E**) consumed sucralose in their drinking water as in (**C**). They were subjected to the AOM-DSS protocol (injected with 10 mg/kg AOM on day 0 and given 3% DSS in the drinking water on days 7–14 and 28–35). Overall tumor number and composition of “large” tumors (>2 mm in any direction) are shown. Data are a composite of three (**C**) or two (**B** and **D**) independent experiments with five mice per group per experiment. Error bars represent the mean ± SEM. A two-way ANOVA (**C**) or Student *t* test (**D**) was used. *, *P* < 0.05; ns, not significant.

In order to dissect potential mechanisms of resistance as a result of sucralose consumption, we utilized two mouse models of cancer: MC38 (adenocarcinoma) and B16 (melanoma). Mice were exposed to a physiologically equivalent dosage of sucralose (0.09 mg/mL, ∼0.45 mg/day) based on their increased basal metabolic rates compared with humans (30) in the drinking water starting 2 weeks before tumor injection and were maintained on sucralose-containing water for the duration of treatment with anti–PD-1 (days 9, 12, and 15; Fig. 2A). Similar to our findings in patients with anti–PD-1–treated NSCLC and melanoma, mice consuming sucralose were resistant to PD-1 blockade in both MC38 (circles) and B16 (squares) and had significantly increased tumor growth, less CD8^+^ T-cell infiltration, and reduced survival across multiple tumor models (Fig. 2B–D; Supplementary Fig. S4A–S4E). Conversely, consumption of sucrose (table sugar) had no negative impact on response to anti–PD-1 (Fig. 2B). Tumor growth kinetics differed between mice sourced from Taconic (filled circles) compared with The Jackson Laboratory (open circles; Fig. 2C; Supplementary Fig. S4B), which house genetically identical mice with significantly distinct gut microbiomes (31, 32). Additionally, tumor burden and size were significantly increased in sucralose-consuming mice after azoxymethane-dextran sulfate sodium (AOM-DSS)–driven colorectal cancer; however, this increase was only observable in mice sourced from Taconic and not in those from The Jackson Laboratory (Fig. 2D and E). Furthermore, cohousing of mice sourced from The Jackson Laboratory and Taconic reduced source effects on tumor growth (Supplementary Fig. S4F and S4G), suggesting a potential microbiome effect; therefore, we focused downstream analyses on Taconic mice. Taken together, these data suggest that consumption of NNS prior to or during immunotherapy contributes to immunotherapy resistance in both mouse models and patients with cancer.

### Sucralose Alters the Tumor Microenvironment via Dysregulation of T-cell Function

**Figure 3.**
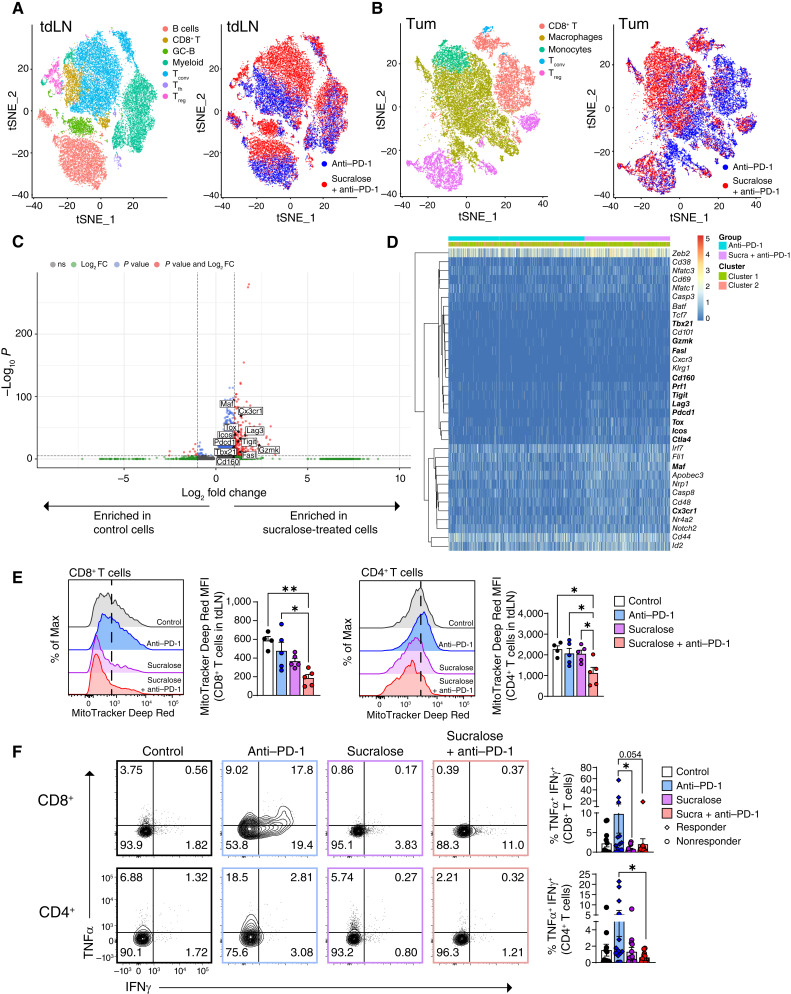
Sucralose alters the tumor microenvironment and supports T-cell dysfunction. C57BL/6 mice from Taconic consumed sucralose in the drinking water (0.09 mg/mL) for 2 weeks prior to tumor injection and for the duration of the experiment. Mice were injected with 2.5 × 10^5^ MC38 cells subcutaneously and treated with 200 µg anti–PD-1 on days 9 and 12. **A–D,** CD45^+^ cells were isolated from the tumor and tdLN prior to single-cell RNA sequencing on day 14 after tumor injection. **A** and **B,** Uniform Manifold Approximation and Projection of clusters identified in tdLN (**A**) and tumor (**B**) in mice treated with anti–PD-1 ± sucralose. tSNE, t-distributed stochastic neighbor embedding. **C,** Volcano plot of gene expression from CD8^+^ T cells in the tumor of sucralose + anti–PD-1 vs. anti–PD-1–treated mice. **D,** Exhaustion signature heatmap for CD8^+^ T cells in the tumor comparing sucralose + anti–PD-1 (purple) with anti–PD-1 (teal). **E,** Representative flow cytometry plots and quantification of MitoTracker DeepRed staining in CD8^+^ T cells or CD4^+^ T_conv_ cells from tdLN. **F,** Representative flow cytometry plots of TNFα and IFNγ staining in CD8^+^ T cells and CD4^+^ T_conv_ cells in the tumor tissue of mice consuming sucralose and/or anti–PD-1. Responder mice in anti–PD-1 ± sucralose groups are shown as diamonds. Data are representative (**E**) or a composite (**F**) of three or one (**A–E**) independent experiments, respectively, with five mice per group per experiment. Error bars represent the mean ± SEM. A one-way ANOVA with the Tukey multiple comparisons test (**E** and **F**) was used. *, *P* < 0.05; **, *P* < 0.005.

In order to determine how sucralose affects the tumor microenvironment, we performed single-cell RNA sequencing on 76,804 cells isolated from the tumors (41,850 cells) and tumor-draining lymph nodes (tdLN; 34,954 cells) of sucralose-consuming, anti–PD-1–treated Taconic mice. We identified seven clusters in the tdLN and five clusters in the tumor that were annotated using marker genes (Fig. 3A and B). An unsupervised visualization indicated clear differences in the global transcriptomic profiles of both tumor and tdLN cells from anti–PD-1–treated mice with and without sucralose treatment (Fig. 3A and B). To better dissect these differences, we used a novel supervised latent factor regression approach, Significant Latent Factor Interaction Discovery and Exploration (SLIDE), to identify latent factors that likely drive immunotherapy resistance during sucralose consumption (Supplementary Fig. S5; ref. 33). Its unique statistical properties enable SLIDE to move beyond simple biomarkers to actual inference of the basis of immunotherapy resistance. Leveraging transcript abundances solely of CD8^+^ T cells in the tdLN, the SLIDE model provided significant discrimination between cells with and without sucralose treatment, and it converged on three significant latent factors (context-specific co-expression modules) underlying altered CD8^+^ T-cell phenotype and function (Supplementary Fig. S5A–S5J). These three latent factors contained gene signatures associated with proliferative and functional stunting (downregulation of *Cdk8*, *Actb*, and *Pfn1*; refs. 34–36) and severe dysregulation or potential exhaustion (including downregulation of *Il2rg* and upregulation of *Rps28* and *Rpl38*; refs. 37, 38) found within the mice consuming sucralose (Fig. 3C and D). Interestingly, CD4^+^ conventional T (T_conv_) cells in the tdLN were similarly affected and independently provided significant discrimination between the two groups (Supplementary Fig. S5D and S5E). Although regulatory T (Treg) cells also had distinctly altered transcriptomic profiles, the corresponding significant latent factors were suggestive of increased suppressive and functional capacity (*Ctla4* and *Il2ra*; Supplementary Fig. S5F; refs. 39–42). Separate SLIDE models were also built using transcript abundances within the tumor compartment. We identified three significant latent factors within the CD8^+^ T-cell compartment, most of which suggested metabolic stress and exhaustion (*Sirpa*, *Apoe*, and *Nrf2*) and provided clear discrimination between groups (Supplementary Fig. S5G–S5J; refs. 43–45). Interestingly, changes in the CD4^+^ T_conv_ subset within the tumor were far less discriminative than those in the tdLN, likely reflective of the known predominant role of CD8^+^ T cells in mediating antitumor immunity and corresponding immunotherapy resistance upon sucralose treatment (Supplementary Fig. S5K–S5N). Further analysis of CD8^+^ T cells from the tumor and tdLN of sucralose + anti–PD-1 mice showed an increase in T-cell exhaustion signatures, including increases in *Pdcd1*, *Tox*, *Lag3*, *Tigit*, *Icos*, *Ctla4*, and *Prf1*, as well as downregulation of some solute carrier (SLC) family members, including those that transport glucose (*Slc2a3* and *Slc2a1*) and those required for mitochondrial respiration (*Slc25a36* and *Slc25a19*; Fig. 3C and D; Supplementary Fig. S6A–S6E).

When assessed at the protein level, both CD8^+^ T cells and CD4^+^ T_conv_ cells displayed signs of reduced T-cell cytotoxic function, including a significant reduction in total mitochondrial mass (Fig. 3E), reduced TCF1:Tox ratios (Supplementary Fig. S7A), and reduced polyfunctionality as shown by TNFα and IFNγ (Fig. 3F; ref. 37). No significant differences in cell number were noted; however, the activation status of T cells was significantly altered, especially in the tdLN of sucralose-consuming mice (Supplementary Fig. S7B–S7D). We next sought to determine whether sucralose-driven T-cell dysfunction was unique to the tumor microenvironment. We infected mice consuming sucralose with LCMV cl-13 prior to treatment with anti–PD-1. Sucralose-consuming mice experienced more severe disease and an increase of T-cell exhaustion, especially in gp33^+^ LCMV-specific CD8^+^ T cells (Supplementary Fig. S7E-l), suggesting that the effects of sucralose on T-cell function were not restricted to cancer but rather may contribute to overall T-cell dysfunction across disease states ranging from cancer to chronic viral infection.

We next performed a series of *in vitro* assays to determine whether sucralose could have a direct effect on T-cell function and whether the timing of sucralose exposure affected T-cell function. CD8^+^ T cells expanded in sucralose-supplemented media proliferated slower than untreated controls (Supplementary Fig. S8A and S8B). Furthermore, CD8^+^ T cells had increased apoptosis as measured by annexin V after 20-hour exposure to a downstream microbial metabolite, sucralose-6-acetate (Supplementary Fig. S8C). T cells cultured in sucralose were moderately less functional, as shown through reduced granzyme B expression and reduced ability to kill target cells *in vitro* (Supplementary Fig. S8D–S8F). Additionally, T cells expanded for 7 days in sucralose (in the absence of persistent antigen) showed moderate signs of metabolic exhaustion with reduced mitochondrial mass (Supplementary Fig. S8G); however, other markers of T-cell exhaustion such as PD-1, Tox, oxidative respiration, and glycolysis were not affected (Supplementary Figs. S8H–S8O and S9A–S9D). There were no observable differences in proximal T-cell receptor signaling (Supplementary Fig. S10). RNA sequencing of T cells exposed to sucralose *in vitro* for 7 days showed shifts in metabolic pathways, including downregulation of amino acid metabolism (*Slc7a3*), the tricarboxylic acid (TCA) cycle, and glycolysis (Supplementary Fig. S11A and S11B). Although some of the macroscopic transcriptional properties identified via *in vitro* readouts mimicked those found directly *ex vivo* (decreased cell killing and metabolic rates), there was only mild overlap between *in vitro* and *ex vivo* analyses, suggesting that the host environment is critical for sucralose-driven T-cell dysfunction. Taken together, these data not only suggest that sucralose consumption has deleterious effects across multiple T-cell processes, including proliferation, cytotoxic function, and metabolism, but also that the severity of T-cell dysfunction is dependent upon the host factors, most notably the microbiome.

### The Gut Microbiome Is Necessary and Sufficient to Limit Immunotherapy Response after Sucralose Consumption

**Figure 4.**
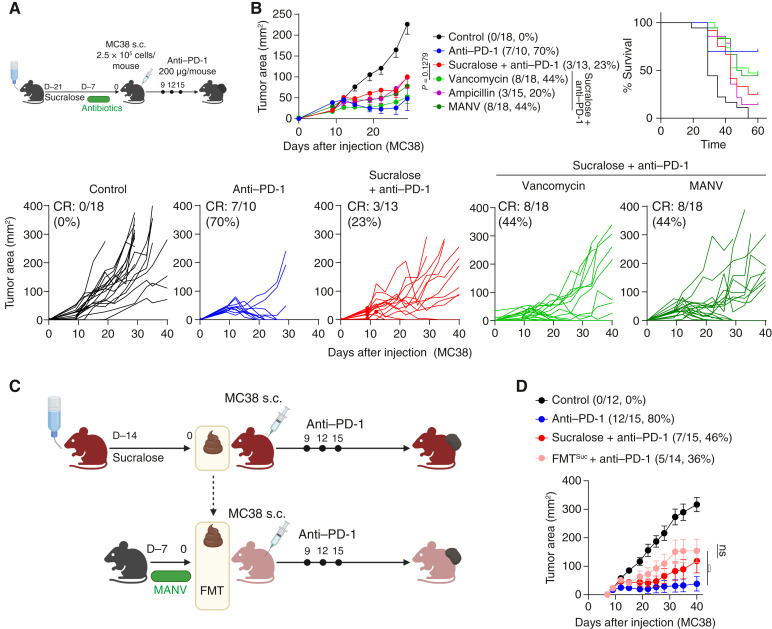
The gut microbiota is necessary and sufficient to drive immunotherapy resistance due to sucralose. **A,** Mice consumed sucralose (0.09 mg/mL) in the drinking water for 14 days prior to antibiotic treatment and for the duration of the experiment. After 21 days of sucralose supplementation with or without antibiotics, mice were injected with 2.5 × 10^5^ MC38 cells subcutaneously and treated with anti–PD-1 on days 9, 12, and 15. **B,** Tumor growth curve and overall survival plot of experiment described in **A**. CR, complete response. **C,** FMT experimental overview. Donor mice (red) were given sucralose-supplemented drinking water (0.09 mg/mL) for 2 weeks prior to donating stool. Stool was transferred to sucralose-naïve recipient mice (light pink) that had received broad-spectrum antibiotics for 7 days prior to transfer. Tumors were injected as previously described in 4**A** and measured until endpoint. **D,** Tumor growth curve of the experiment described in **C**. Data are a composite of three (**B** and **C**) independent experiments with five mice per group per experiment. Error bars represent the mean ± SEM. Two-way ANOVA (**B** and **D**) was used. *, *P* < 0.05.

**Figure 5.**
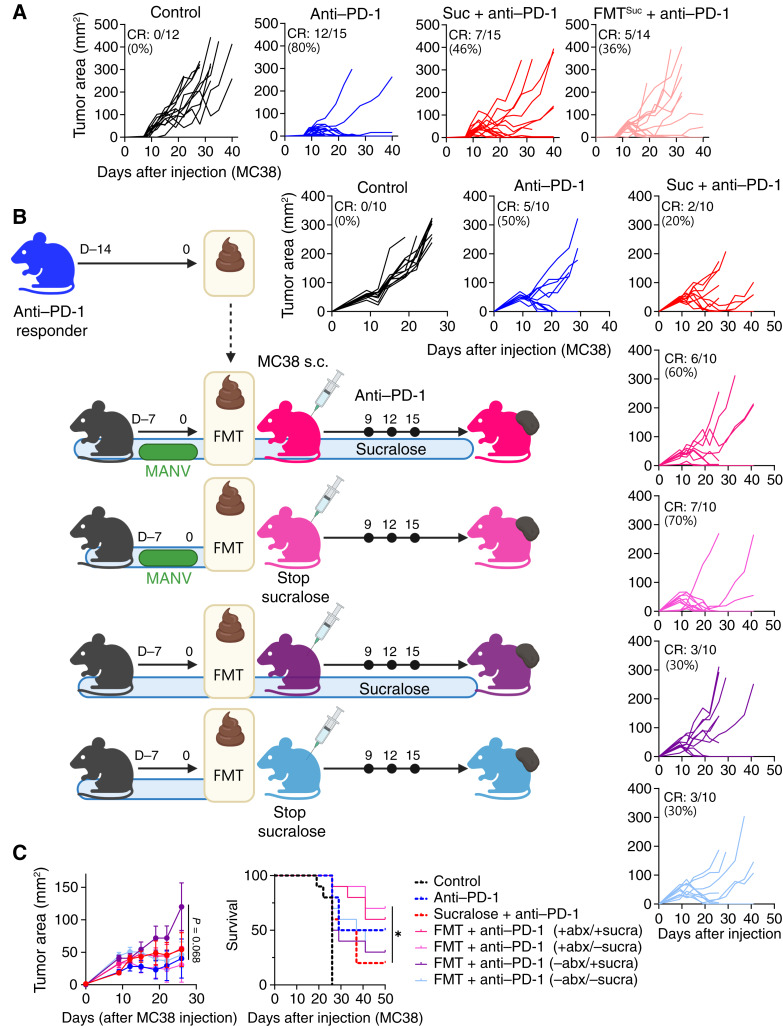
Responder-derived FMT is sufficient to restore immunotherapeutic response. **A,** Individual tumor growth curves from Fig. 4D with FMT. **B** and **C,** C57BL/6 mice from Taconic consumed sucralose in the drinking water (0.09 mg/mL) for 2 weeks prior to tumor injection and for the duration of the experiment. Mice were injected with 2.5 × 10^5^ MC38 cells subcutaneously and treated with 200 µg anti–PD-1 on days 9, 12, and 15. Tumor area was measured every 3 days until endpoint. Four groups all received an FMT from an anti–PD-1 responder mouse donor. The following four groups were used: antibiotic treatment pre-FMT, continued sucralose consumption (hot pink), antibiotic treatment pre-FMT, stopped sucralose consumption (light pink), no antibiotics, continued sucralose consumption (purple), no antibiotics, stopped sucralose consumption (light blue). Individual tumor growth curves (**B**) and a composite growth curve and OS (**C**) are shown. Data are a composite of three (**A**) or two (**B** and **C**) independent experiments with five mice per group per replicate. Error bars represent the mean ± SEM. For growth curve statistics, two-way ANOVA was used. OS statistics were calculated using a log-rank Mantel–Cox test. *, *P* < 0.05. abx, antibiotics; CR, complete response.

ICI response in patients with cancer is associated with select members of the gut microbiome (46–50). We observed that mice sourced from different vendors (harboring distinct microbiomes) varied in their response to anti–PD-1 upon consuming sucralose (Fig. 2C–E). Therefore, we hypothesized that the gut microbiome may be responsible for T-cell dysfunction and ICI resistance in the presence of sucralose. Therefore, we subjected mice to sucralose as before with the addition of select antibiotics, including vancomycin, ampicillin, or broad-spectrum antibiotics (metronidazole, ampicillin, neomycin, and vancomycin; Fig. 4A). Interestingly, although ampicillin had no impact on tumor growth during sucralose consumption (complete response: 20%), vancomycin, a bacteriostatic antibiotic that targets Gram-positive bacteria (complete response: 44%), increased overall response rates in mice consuming sucralose and receiving anti–PD-1 (Fig. 4B), suggesting that the gut microbiome may be associated with sucralose-driven resistance to anti–PD-1. To determine whether the gut microbiota was sufficient to drive ICI resistance after sucralose consumption, we performed fecal microbial transfers (FMT) from sucralose-consuming donor mice to sucralose-naïve mice prior to injecting tumors and treating with anti–PD-1 (Fig. 4C). Strikingly, FMTs from sucralose-consuming mice phenocopied direct consumption of sucralose in both tumor progression and OS (Fig. 4D). Therapeutic FMTs from responder patients or healthy donors have shown great promise as a therapeutic option in conjunction with anti–PD-1 to patients with resistant or refractory melanomas (51, 52). Therefore, we sought to determine whether sucralose-driven resistance could be therapeutically overcome with an FMT. Indeed, ICI resistance of sucralose-consuming mice was reversible by performing an FMT from anti–PD-1 responder mice, with the most significant benefit shown in those that were prepped with antibiotics before the FMT, suggesting that removal of sucralose-associated bacteria was required for response to anti–PD-1 (Fig. 5A–C).

**Figure 6.**
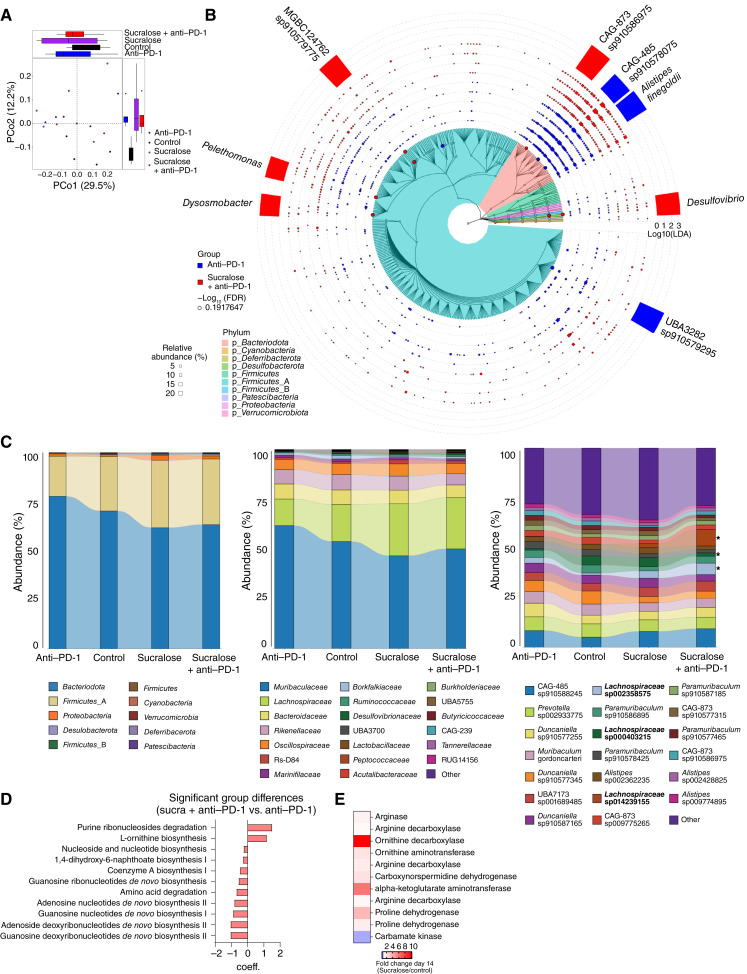
Sucralose consumption shifts gut microbiome diversity and function. C57BL/6 mice from Taconic consumed sucralose in the drinking water (0.09 mg/mL) for 2 weeks prior to tumor injection and for the duration of the experiment. Mice were injected with 2.5 × 10^5^ MC38 cells subcutaneously and treated with 200 µg anti–PD-1 on days 9, 12, and 15. **A,** Principal coordinate analysis (PCoA) plot of the gut microbiome of sucralose-consuming vs. -abstaining mice on day 38. Serial stool collections were obtained on days 0, 14, 28, and 38 after the start of sucralose consumption and sent for shallow shotgun sequencing. **B,** Cladogram with significantly overexpressed taxa in anti–PD-1 or sucralose + anti–PD-1 groups at day 38. In the cladogram, the size of the circle (node) encodes the –log10(FDR) value. **C,** Taxonomic relative abundance bar plots for the groups at day 38. Percent abundance on the *y*-axis indicates the mean abundance for all mice within each group. Asterisks label bacterial species that are arginine degrading and enriched in sucralose-consuming groups. **D,** Functional pathway analysis of sucralose + anti–PD-1 vs. anti–PD-1. Bar chart shows significant pathways with the default MaAsLin2 fdr threshold of <0.25. The MaAsLin2 coef value is reported on the *x*-axis. **E,** Functional pathway analysis of the arginine degradation pathway between sucralose + anti–PD-1 and anti–PD-1. Heatmap shows fold change of arginine-degrading enzymes expressed by bacteria in sucralose-consuming groups. Data are representative of one independent experiment with five mice per group. FDR, false discovery rate.

In order to determine how the gut microbiome is contributing to sucralose-driven resistance, we performed shallow shotgun metagenomic sequencing on serial stool samples of mice consuming sucralose and receiving anti–PD-1. Interestingly, sucralose significantly shifted the gut microbiome, regardless of anti–PD-1 treatment (Fig. 6A and B; Supplementary Fig. S12A and S12B). α-diversity and evenness were reduced in both sucralose and sucralose + anti–PD-1–treated groups compared with controls, although this was not significant (Supplementary Figs. S12C and S13A). Specifically, we observed increases in *Firmicutes* and *Proteobacteria* phyla, with a relative outgrowth of select Gram-positive bacteria, including *Clostridiaceae* and *Lachnospiraceae*, after sucralose consumption (Fig. 6C; Supplementary Fig. S13B). Thus, these data suggest that ICI resistance after sucralose consumption is possibly due to phenotypic or functional shifts in the gut microbiota and is associated with an outgrowth of Gram-positive bacteria.

### Arginine Supplementation during Sucralose Consumption Restores T-cell Function and Immunotherapy Efficacy

**Figure 7.**
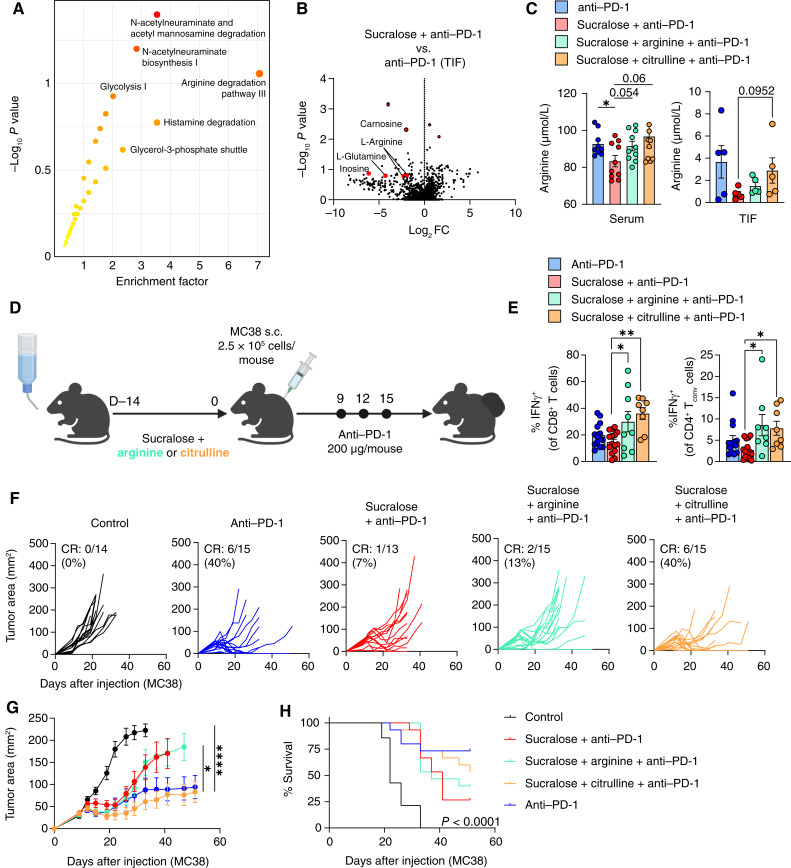
Citrulline supplementation restores T-cell function and immunotherapy efficacy. **A–C,** Serum and TIF were isolated from mice consuming sucralose-supplemented (0.09 mg/mL) drinking water or regular drinking water for 14 days prior to tumor injection and throughout tumor growth. High-resolution LC-HRMS metabolomic analysis was performed. **A,** Pathway analysis of TIF, with significant pathways shown. **B,** Volcano plot comparing metabolite abundance from the TIF of mice consuming sucralose in the drinking water vs. regular water. **C,** Quantification of arginine abundance within the serum and TIF of mice indicated. **D,** Taconic mice were given sucralose-supplemented (0.09 mg/mL) or control drinking water in the presence of absence of arginine or citrulline (3.75 mg/mL) for 2 weeks prior to tumor injection and throughout the duration of the experiment. Mice were injected with 2.5 × 10^5^ MC38 cells subcutaneously and treated with 200 µg anti–PD-1 at days 9, 12, and 15. **E,** Quantification of IFNγ^+^ CD8^+^ T cells and CD4^+^ T_conv_ cells within the tumor 14 days after tumor injection. **F** and **G,** Tumor growth curves of mice from experiment described in **D**. **G,** Tumor growth curves from experiment described in **D**. Data are a composite of three (**E–H**), two (**C**), or one (**A** and **B**) independent experiments with 3–5 mice per group per experiment. Error bars represent the mean ± SEM. The Student *t* test (**C**), one-way ANOVA with the Tukey multiple comparisons test (**E**), two-way ANOVA (**F** and **G**), and Mantel–Cox (**H**) were used. *, *P* < 0.05; **, *P* < 0.005; ****, *P* < 0.00005.

In order to assess how the gut microbiota was functionally affected by sucralose consumption, we further analyzed the functional characteristics from stool samples through both metagenomics and untargeted/targeted metabolomics. Through pathway analysis, we found several significantly altered pathways involving amino acids (Fig. 6D). Upon further investigation, we identified an increase in enzymes associated with arginine degradation pathways (Fig. 6E). Metabolomic analysis of stool samples showed that sucralose and its microbial metabolite, sucralose-6-acetate, were only found within stool samples from mice consuming sucralose in the drinking water as expected (Supplementary Fig. S13C). Pathway analysis of untargeted stool metabolomics revealed that a number of pathways were enriched because of sucralose consumption during anti–PD-1 treatment, including arginine degradation (Fig. 7A). Indeed, levels of arginine, as well as many associated metabolites (citrulline), were significantly reduced in the stool of sucralose-consuming mice (Supplementary Fig. S13D), whereas other metabolites were unaffected (Supplementary Fig. S13E), suggesting a potential role for arginine degradation after sucralose consumption. Upon further analysis, we also found that arginine levels in the sucralose + anti–PD-1 group were significantly reduced in both the serum and tumor interstitial fluid (TIF; Fig. 7B and C). Additionally, we found that sucralose exposure led to a reduction in the amino acid transporter, *Slc7a3*, which is responsible for arginine uptake (Supplementary Fig. S11A). Arginine is a key metabolite required for optimal T-cell metabolism and function, and it has been previously associated with cytotoxic T-cell function in cancer (53). To test the possibility that a reduction in arginine was responsible for ICI resistance, we supplemented sucralose-containing drinking water with arginine (3.75 mg/mL) or citrulline (3.75 mg/mL), the latter of which has been shown to lead to the highest levels of serum arginine *in vivo* (Fig. 7D; refs. 54, 55). Metabolomic analysis of the serum and TIF revealed that citrulline could restore levels of arginine in both sites to that of anti–PD-1–treated mice (Fig. 7C). In addition, both CD4^+^ and CD8^+^ T cells from arginine- or citrulline-fed mice displayed enhanced function shown by an increase in IFNγ production in the tumor, with the biggest increase found in citrulline-treated groups (Fig. 7E). Quite strikingly, supplementation with citrulline restored response to anti–PD-1, even in the presence of sucralose, and led to an OS advantage (Fig. 7F–H; Supplementary Fig. S13F). Taken together, these data suggest that sucralose shifts the gut microbiota in a way that reduces arginine levels and ultimately drives resistance to anti–PD-1.

## Discussion

The composition of the gut microbiota has been directly correlated with ICI response in multiple cancers, including melanoma and NSCLC (10, 11, 13, 14, 46, 47, 56). The development of therapeutic strategies to shift the gut microbiota is underway through the use of probiotics, FMT, and dietary interventions. Certain diets, including those high in fiber and fermented foods, can significantly alter the diversity and makeup of gut microbiota and consequently alter immunologic outcomes in healthy volunteers and ICI-treated patients with cancer (57, 58). Although various dietary elements, such as dietary fiber, augment ICI efficacy by favorably modulating gut microbiome, how other dietary elements, including NNS intake, affect ICI efficacy is unknown. Our work suggests that increased sucralose consumption is associated with poorer efficacy of ICI-based immunotherapy in patients with melanoma and NSCLC and sheds light on how sucralose may affect T-cell functionality and ICI efficacy in a gut microbiome–centric fashion. Future prospective studies will be necessary to assess potential causation of sucralose in driving immunotherapy resistance, as well as to determine how other demographic factors, including location and food access, may affect overall responses.

NNS use has increased significantly over the last 50 years in an attempt to reduce table sugar consumption. Given that most NNS are low or zero calorie, these have traditionally been overlooked as contributing factors to overall health and disease. Multiple NNS, including sucralose, saccharin, and others, have recently been shown to reduce microbial diversity and lead to glucose intolerance in healthy hosts (12, 24, 59). Interestingly, sucralose has been associated with poor T-cell function in multiple disease states when supraphysiologic amounts are consumed, suggesting a potential immunosuppressive mechanism of some NNS such as sucralose. How NNS may contribute to cancer progression and immunotherapy response remained unknown.

Our results show that increased high pretreatment sucralose intake was associated with lower PFS in patients with advanced cutaneous melanoma and NSCLC treated with PD-1–based ICI regimens, and lower RFS in patients with high-risk resectable melanoma treated with anti–PD-1 and TLR9 agonist, hinting at the ability of sucralose to blunt the efficacy of ICI immunotherapy regardless of histology or stage. Preclinical studies of melanoma and carcinogen-induced colorectal cancer suggested that sucralose consumption drove poor response to checkpoint inhibitors through microbial dysbiosis and downstream CD8^+^ T-cell dysfunction, a fundamental but not well-understood mechanism of ICI resistance. Given that this is limited to two tumor models in mice, further analysis of other cancer types and immunotherapy modalities may provide more insights into the broad implications of sucralose consumption. Although sucralose may have some effects directly upon T-cell activation (Supplementary Fig. S8; ref. 27), our data suggest that sucralose consumption interacts directly with gut microbiota to diminish sensitivity to anti–PD-1 therapy and is both necessary and sufficient to drive degradation of key amino acids, CD8^+^ T-cell dysfunction, increased tumor growth, and reduced response to anti–PD-1. These results are in line with prior observations about the host-specific microbiome-dependent metabolic alterations observed in patients treated with sucralose (25). Therefore, dietary effects on the microbiota may represent a previously unappreciated mechanism of resistance to checkpoint blockade in melanoma or other cancer types. Furthermore, our study bolsters the growing notion that artificial NNS, even those manufactured from sugar-like sucralose, are not inert and can have broad immunomodulatory effects that adversely affect patient outcomes. Our results suggest that select metabolites that are regulated by the gut microbiota may be required for ICI response and may represent a fundamental component of antitumor immunity.

## Methods

### Patient Samples

#### Human Study Subjects – HCC 20-019

This study includes dietary history data from patients with advanced cancer treated with anti–PD-1–based ICI therapy and evaluated at the University of Pittsburgh Medical Center HCC in Pittsburgh, PA. All patients provided voluntary written informed consent to research procedures, including biospecimen collection, under Institutional Review Board–approved protocol HCC 20-019 (Comprehensive Intestinal Microbiome and Dietary History Evaluation of Patients with Advanced Cancers on Treatment with Immune Checkpoint Blockade, Institutional Review Board approval number MOD20010266-016). All studies included were conducted in accordance with recognized ethical guidelines as applicable (Declaration of Helsinki, Council for International Organizations of Medical Sciences (CIOMS), Belmont Report, and US Common Rule).

Specifically, we included patients who had received systemic anti–PD-1–based immunotherapy or chemoimmunotherapy between September 2020 and June 2024. Eligible patients had received systemic anti–PD-1–based immunotherapy or chemoimmunotherapy for advanced/metastatic melanoma or NSCLC as part of standard treatment (outside of a clinical trial), had undergone dietary history evaluation using DHQ III, had received treatment for at least 3 months, had at least one posttreatment imaging study evaluable for response, and had adequate follow-up time (6 months) following initiation to therapy.

Radiographic response to therapy was determined by the investigators providing the clinical treatment and assessed using RECIST v1.1 (60). Clinical response to therapy was assessed at each visit. Progression was defined based on first documented clinical and/or radiographic progression and confirmed in all instances.

Overall, 132 patients with advanced cancer (melanoma = 91; NSCLC = 41) who received systemic PD-1–based immunotherapy singly or in combination with chemotherapy and met other criteria as above were included in the primary analysis of patient outcomes (Supplementary Fig. S1).

#### Human Study Subjects – HCC 17-169 (Phase II Trial of Neoadjuvant Nivolumab and TLR9 Agonist Vidutolimod in High-Risk Resectable Melanoma)

This study includes dietary history data from patients who were enrolled in a prospective phase II trial of neoadjuvant nivolumab and TLR9 agonist vidutolimod in high-risk resectable melanoma, the primary results of which were previously reported (28).

Briefly, this single-institution, non-randomized, investigator-initiated phase II study enrolled histologically proven clinical stage III cutaneous melanoma. Eligible patients received neoadjuvant intratumoral vidutolimod and systemic nivolumab prior to surgery. Patients received three dosages of intravenous nivolumab (240 mg) every 2 weeks, together with seven weekly dosages of vidutolimod (5 mg subcutaneous on week 1 and then 10 mg intratumoral on weeks 2–7). Following neoadjuvant treatment, patients underwent restaging imaging and proceeded to surgery. Following surgery, all patients received adjuvant systemic nivolumab and subcutaneous vidutolimod.

The primary endpoint of the trial was MPR as assessed using consensus criteria (61–63). A key secondary endpoint was RFS, defined as time from surgery to disease recurrence or death from any cause.

Briefly, 34 patients were enrolled, but three withdrew consent, and hence, 31 were evaluable for safety, whereas 30 were evaluable for pathologic response (one patient had systemic disease progression after neoadjuvant therapy precluding surgery). DHQ III data were available on 25 of the 31 enrolled patients.

#### Clinicodemographic Variables

Body mass index, neutrophil:lymphocyte ratio, and lactate dehydrogenase were based on values obtained immediately before therapy or on the day of ICI therapy initiation. Tumor-specific characteristics, including TMB and PD-L1 status, were abstracted from the electronic record.

#### Survival Endpoints

PFS was defined as the time from the start of therapy to first confirmed clinical and/or radiographic progression. RFS was defined as time from surgery to disease recurrence or death from any cause. Patients were censored as of the date of last contact.

#### Assessment of Dietary Data

Dietary intake data were collected using DHQ III, a standardized and validated tool for nutritional assessment developed by the NCI’s Division of Cancer Control and Population Sciences. DHQ III was designed based on a compilation of national 24-hour dietary recall data from the National Health and Nutrition Examination Surveys conducted in 2007 to 2014 (64). We utilized the “past month with portion size” DHQ III FFQ version, and this was self-administered via a web-based portal using a tablet to patients within 3 weeks of them starting ICI therapy. To facilitate dietary data intake by patients who may not be comfortable with self-administered questionnaires, administration was proctored by trained research coordinators. Responses to all 135 questions were converted to daily frequencies.

DHQ III consists of 135 food and beverage line items and 26 dietary supplement questions. Some line items for foods and beverages have additional embedded questions that allow for final assignment to items in the nutrient and food group database, leading to 263 foods/beverages listed in the database. For example, a single line item asks frequency of intake and portion size of soda or soft drinks. Embedded underneath are questions about whether the soft drinks consumed are regular versus diet or caffeinated versus decaffeinated. Answers to these questions lead to assignment of one of four food codes in the database: diet soda with caffeine, diet soda without caffeine, regular soda with caffeine, or regular soda without caffeine.

Overall, DHQ III FFQ permits estimation of each patient’s daily dietary intake of any particular nutrient, macronutrient, or vitamin as an average over the previous month. Correlation between DHQ III and 24-hour recall studies has been previously published (65).

All patients who completed DHQ III were included in the analysis, but those who did not complete the entire questionnaire, had received <3 months of ICI therapy, or were found to consume <1,000 kcal/day (suggestive of inaccurate reporting) were excluded.

DHQ III output includes NNS intake as an average daily intake of mg/day. NNS dietary data were normalized by taking each patient’s respective NNS intake (mg/day) and dividing by their weight (kg) to have a weight-normalized ADI of mg/kg/day. The weight-normalized ADI could then be compared with the FDA’s ADI limit, previously reported for the various NNS: sucralose (5 mg/kg/day), aspartame (50 mg/kg/day), acesulfame (15 mg/kg/day), and saccharin (15 mg/kg/day).

#### Statistical Methods

To evaluate sucralose (and other NNS) thresholds, we utilized CutpointR (29), which robustly estimates the optimal cutpoint in any given distribution. The CutpointR-defined threshold for any given NNS was used to dichotomize that cohort. Kaplan–Meier survival analysis was performed with StataSE, using the optimal cutpoint determined by CutpointR with the minimize_metric, to define the association of high or low NNS intake with ORR or PFS. The *χ*^2^ test was used to compare the investigator-assessed ORR between high- and low-intake groups.

Univariate and multivariate analyses were performed by a PhD biostatistician (H.W.) to investigate the association of normalized sucralose intake and other covariables with PFS (or RFS) and ORR (or MPR). The covariables assessed included gender, body mass index, pretreatment neutrophil:lymphocyte ratio, pretreatment lactate dehydrogenase, soluble dietary fiber intake (g/day), insoluble dietary fiber intake (g/day), hypertension, and heart disease status. We also assessed the effect of combination versus single-agent ICI therapy (only patients with melanoma) and the effects of chemoimmunotherapy versus single-agent ICI therapy, TMB (continuous variable), and PD-L1 status (1% vs. >1%) in only patients with NSCLC.

Univariate logistic regression models were used to test the association of ORR (or MPR) with each variable. We used the stepwise model selection procedure to build a multivariate logistic regression model in which high sucralose intake (>0.16 mg/kg/day) was forced in the model. In this procedure, we started from a univariate model with high sucralose intake (>0.16 mg/kg/day). Then, at each step along the way, we either entered or removed a predictor based on the *χ*^2^ test *P* value for the effect of this predictor in the multivariate model. We stopped when no more predictors could be justifiably entered or removed from our stepwise model, thereby leading us to a final model. We set a significance level for deciding when to enter a predictor into the stepwise model and a significance level for deciding when to remove a predictor from the model. We set the two significance levels to be 0.35 and 0.10, respectively. Patients with advanced melanoma, advanced NSCLC, and neoadjuvant melanoma were analyzed using separate univariate logistic regression models.

To test the association of PFS (or RFS) with each variable, a univariate Cox model was used. Patients with advanced melanoma, advanced NSCLC, and neoadjuvant melanoma were analyzed using separate univariate Cox models.

The data were analyzed in SAS version 9.4 (SAS Institute Inc.).

### *In vitro* T-cell Cultures

Spleen and lymph nodes were isolated from C57BL/6-Tg(TcraTcrb)1100Mjb/J mice (RRID: IMSR_JAX:003831). Cells were activated in complete RPMI-1640 (Gibco, 11875119) supplemented with 10% FBS (Gibco, 16000069), 250 ng/mL SIINFEKL peptide (Genscript, RP10611), and 50 U/mL IL-2 (PeproTech, 212-12). The cells were activated for 24 hours, and then cells were washed and cultured for 6 additional days in RPMI-1640 supplemented with 50 U/mL of complete RPMI supplemented with FBS. For *in vitro* sucralose treatment, control complete RPMI-1640 was supplemented with 0.22 g/L sucralose, and 10-fold dilutions were made using complete RPMI.

### Flow Cytometry

tdLN, non-draining lymph nodes, spleens, and tumors were harvested in 10% complete RPMI-1640 at day 14 after tumor injection. Tumors were also harvested with DNase I and Liberase TL for digestion. Tissues were processed through a 70-μm strainer to obtain single-cell suspension. Single-cell suspensions were stained with live/dead and required surface markers for 15 minutes on ice and then fixed with either eBioscience Foxp3 Transcription Factor (for intracellular transcription factor analysis) reagents or BD Cytofix (for intracellular cytokine analysis) reagents prior to staining intracellular markers for 45 minutes on ice.

Single-cell suspensions were stimulated with phorbol 12-myristate 13-acetate, ionomycin, and brefeldin A for 2.5 hours at 37°C prior to staining and fixation for flow cytometry analysis. After stimulation, single-cell suspensions were stained as described above.

Flow analysis was performed on a Cytoflex (Beckman Coulter) cytometer or a Fortessa (Becton Dickinson, BD) cytometer. All experiments were analyzed using FlowJo Software (v10.9). Cells from tumors, spleens, and lymph nodes were isolated, resuspended in PBS, and placed on ice. Surface markers were stained with fluorescently conjugated antibodies for 30 minutes on ice. All flow analysis included size exclusion (FSC x SSC) and doublet exclusion (FSC-height/FSC-area). Zombie Dye Viability Kits (BioLegend) or Live/Dead Fixable Aqua Stain Kits (Thermo Fisher Scientific) were used to distinguish live and dead cells, and positive dead cells were excluded from analysis. Antibodies were purchased from Thermo Fisher Scientific Invitrogen, BioLegend, BD, Cell Signaling Technology, and R&D Systems.

The following antibodies were used: IL-2 Pe-Cy7 (BD Pharmingen, 560538, RRID: AB_1727545), IFNγ AF647 (BioLegend, 505816, RRID: AB_493314), granzyme B–FITC (BioLegend, 515403, RRID: AB_2114575), TNF–PerCPCy5.5 (BioLegend, 506322, RRID: AB_961435), Tcf1–Af488 (Cell Signaling Technology, 6444S, RRID: AB_2797627), PD-1–BV785 (BioLegend, 135225, RRID: AB_2563680), Tim-3–BV421 (BioLegend, 119723, RRID: AB_2616908), CD44–PerCPCy5.5 (BioLegend, 103032, RRID: AB_2076206), CD73–APC (BioLegend, 127210, RRID: AB_11219400), CD39–PE-Cy7 (BioLegend, 143806, RRID: AB_2563393), Tox–APC (eBioscience, 50-6502-82, RRID: AB_2574265), CD8a–PE (BioLegend, 100707, RRID: AB_312747), CD8a–Pe-Cy7 (BioLegend, 100721, RRID: AB_312760), IFNγ–eFluor450 (Invitrogen, 48-7311-82, RRID: AB_1834366), CD8b–BV786 (BD OptiBuild, 740952, RRID: AB_2740577), CD4–PE-Cy7 (BD Pharmingen, 552775, RRID: AB_394461) TCR β–PerCP-Cy5.5 (Invitrogen, 45-5961-82, RRID: AB_925763), IL-17A–APC (Invitrogen, 17-7177-81, RRID: AB_763580), TNFα–AF700 (BioLegend, 506338, RRID: AB_2562918), CD62L–BV785 (BioLegend, 104440, RRID: AB_2629685), LAG-3–PerCP-Cy5.5 (BioLegend, 125212, RRID: AB_2561517), Tim-3–PE (BioLegend, 119703, RRID: AB_345377), CD8a–BUV737 (BD Horizon, 612759, RRID: AB_2870090), TCF7/TCF1–AF488 (R&D Systems, IC8224G, RRID: AB_3656680), CD19–eFluor450 (Invitrogen, 48-0193-82, RRID: AB_2734905), FOXP3–AF488 (Invitrogen, 53-5773-82, RRID: AB_763537), CD44–PE-Cy7 (Invitrogen, 25-0441-82, RRID: AB_469623), TOX–eFluor660 (Invitrogen, 50-6502-82, RRID: AB_2574265), CD4–APC (Invitrogen, 17-0042-82, RRID: AB_469323), Rpl18 tetramer–PE (NIH Tetramer Facility), and Rpl18 tetramer–APC (NIH Tetramer Facility).

### Metabolic Labeling for Flow Cytometry

For metabolic staining, neutral lipids were labeled using 1.9 µmol/L Bodipy 493/503 (Thermo Fisher Scientific, D3922) in PBS for 30 minutes at 37°C. Glucose uptake was measured using glucose-Cy3. Single-cell suspensions of ∼1 million cells/mL were placed in serum-free RPMI-1640 containing 0.4 µmol/L glucose-Cy3 for 30 minutes at 37°C. Mitochondrial mass was determined using 10 nmol/L MitoTracker Deep Red FM (Thermo Fisher Scientific, M22426) or MitoTracker Green (Thermo Fisher Scientific, M7514). Cells were labeled with MitoTracker on ice for 30 minutes. Mitochondrial membrane potential was measured using tetramethylrhodamine. Cells were resuspended with PBS supplemented with 20 nmol/L tetramethylrhodamine just prior to running on the cytometer, and cell suspensions were run directly on the cytometer. All metabolic labeling was conducted on live cells, and these samples were not fixed prior to running on the cytometer.

### Extracellular Flux Analysis

Isolated T cells were plated on PDL-Coated Cell Culture Microplates (Agilent, 103799-100) in unbuffered Seahorse XF RPMI assay medium supplemented with 2 mmol/L glutamine, 1 mmol/L sodium pyruvate, and 10 mmol/L glucose. The oxygen consumption rate (OCR) and extracellular acidification rate (ECAR) were measured in an Agilent Seahorse XFe96 extracelluar flux analyzer. During the assay, the cells received four separate injections of (i) 2 µmol/L oligomycin, (ii) 2 µmol/L carbonyl cyanide-p-(trifluoromethoxy)phenylhydrazone (FCCP), (iii) 10 mmol/L 2-deoxy-D-glucose, (iv), and 0.5 µmol/L rotenone and 0.5 µmol/L antimycin A was used.

### xCELLigence Real-Time Killing Assay

A total of 15,000 B16_OVA cells were seeded onto the E-Plate VIEW 96 (Agilent, 300601020) and incubated for a minimum of 5 hours. Subsequently, primary CD8^+^ T cells, isolated from C57BL/6-Tg(TcraTcrb)1100Mjb/J (strain #003831, common name: OT1), were introduced to the E-Plate 96 at various effector to target ratios. Following this, the system was left undisturbed for a minimum of 18 hours to allow the Agilent xCELLigence RTCA DP system to record impedance changes. The RTCA Software Pro was utilized for cytolysis calculations.

### Western Blotting

CD8^+^ T cells were magnetically enriched from splenocytes derived from C57BL/6J mice. After isolation, the cells were resuspended in control serum-free RPMI or serum-free RPMI supplemented with 2% Splenda or 0.22 g/L sucralose and stimulated with 3 μg/mL biotin-labeled αCD3 antibody, 4 μg/mL of αCD28 antibody, and + 1.5 μg/mL streptavidin for the indicated times: 5, 15, 30 minutes, or left unstimulated (0 minute) at 37°C. After stimulation, the cells were lysed, and Western blots were conducted using the following antibodies: Phospho-Akt (Ser473; D9E) XP Rabbit mAb #4060, Akt (pan; C67E7) Rabbit mAb #4691, mTOR Antibody #2972, Phospho-mTOR (Ser2448) Antibody #2971, PLCγ1 (D9H10) XP Rabbit mAb #5690, Phospho-PLCγ1 (Ser1248; D25A9) Rabbit mAb #8713, Zap-70 (D1C10E) XP Rabbit mAb #3165, Phospho-Zap-70 (Tyr319)/Syk (Tyr352) Antibody #2701, p70 S6 Kinase (49D7) Rabbit mAb #2708, Phospho-p70 S6 Kinase (Thr389) Antibody #9205, Lck Antibody #2752, Phospho-Lck (Tyr505) Antibody #2751, p44/42 MAPK (Erk1/2; 137F5) Rabbit mAb #4695, and Phospho-p44/42 MAPK (Erk1/2; Thr202/Tyr204) Antibody #9101.

### Flow Cytometry Single-Cell Suspensions and Staining

tdLN, non-draining lymph nodes, spleens, and tumors were harvested in 10% complete RPMI-1640 at day 14 after tumor injection. Tumors were also harvested with DNase I and Liberase TL for digestion. Tissues were processed through a 70-μm strainer to obtain single-cell suspension. Single-cell suspensions were stained with live/dead and required surface markers for 15 minutes on ice and then fixed with either eBioscience Foxp3 Transcription Factor (for intracellular transcription factor analysis) reagents or BD Cytofix (for intracellular cytokine analysis) reagents prior to staining intracellular markers for 45 minutes on ice.

Single-cell suspensions were stimulated with phorbol 12-myristate 13-acetate, ionomycin, and brefeldin A for 2.5 hours at 37°C prior to staining and fixation for flow cytometry analysis. After stimulation, single-cell suspensions were stained as described above.

### Mice

Female 6-week-old C57BL/6 mice were obtained from The Jackson Laboratory and Taconic Biosciences. All animal experiments were performed in the American Association for the Accreditation of Laboratory Animal Care–accredited specific pathogen-free facilities in the Division of Laboratory Animal Resources at the University of Pittsburgh School of Medicine. All animal protocols were approved by the Institutional Animal Care and Use Committees of the University of Pittsburgh. Mice in the same group were cohoused unless otherwise stated.

C57BL/6 mice sourced from both The Jackson Laboratory and Taconic Biosciences were given approximately 24 hours to acclimate to the BSL2 animal facility prior to starting treatment with sucralose. All mice were always kept in immunocompromised housing conditions. All Jackson-sourced mice were handled prior to handling any Taconic-sourced mice. Full disinfection of all surfaces by lab staff was performed between Jackson and Taconic mice to minimize cross contamination. In cohousing experiments, Jackson- and Taconic-sourced C57BL/6 mice were mixed into cages equally and lab staff took care in disinfecting all surfaces and tools in between cages. Mice were always handled in the order of: Jackson, Taconic, and cohoused mice with disinfection between each group.

### Sucralose Supplementation

With 36 mg sucralose (Sigma-Aldrich), 400 mL bottles of sterilized water were spiked, equivalent to three packets of sucralose containing NNS. Based on differing basal metabolic rates and surface area between mice and humans, we estimate that mice consuming 18.75 mg/kg/day sucralose is roughly equivalent to humans consuming 1.458 mg/kg/day (ADI is 5 mg/kg/day) based on the previously calculated equation, whereby the reference body weight is 0.02 kg for mice and 60 kg for humans:

(animal dose) × (y) = human equivalent dose     y=0.081 mouse to human

(18 mg/kg) × (0.081) = 1.458 mg/kg/day

Bottles were replaced every 7 to 10 days. Mice consumed sucralose for 14 days prior to tumor cell injections and throughout the duration of the experiment or as noted. For some experiments, water was also supplemented with either 3.75 g/L arginine or citrulline for the duration of the experiment.

### Murine Tumor Models

C57BL/6 mice were injected with MC38 murine colorectal adenocarcinoma (2.5 × 10^5^ cells subcutaneously) or B16 melanoma (1.25 × 10^5^ intradermally). Cell lines were obtained from the Vignali Lab (University of Pittsburgh) and were confirmed *Mycoplasma*-free by PCR in 2022. Cells were saved no later than passage 2 and were injected after two passages directly from freeze thaw. Tumors were measured every 3 days with calipers to calculate tumor area. Mice were randomly assigned to groups prior to tumor injection. Intraperitoneally, 200 μg of anti–PD-1 (Bio X Cell) was injected on days 9, 12, and 15 after tumor injection. Mice were marked as responders if tumor size decreased upon treatment with anti–PD-1. Complete responder was defined as total eradication of tumor, resulting in tumor area of zero.

To establish carcinogen-induced colorectal cancer in mice, we injected 10 mg/kg AOM i.p. on day 0, prior to three cycles of a “1 week on, 2 weeks off” schedule of administering 3% DSS in the drinking water beginning on day 7. Mouse weights were monitored weekly, and mice were euthanized if more than 25% weight was lost. Mice were sacrificed 12 weeks after AOM, and tumors were enumerated and measured.

### Fecal Microbiome Transplants

Female C57BL/6 recipient mice were treated with broad-spectrum antibiotics (metronidazole, ampicillin, neomycin, and vancomycin) for 7 days prior to transplant, unless otherwise noted. Stool pellets were collected from donor mice and mixed into 500 μL of sterile 1× PBS. The supernatant was then filtered through a 70-μM filter and transferred into a syringe with a murine feeding needle attached. Into each mouse, 200 μL of the filtered supernatant was then orally gavaged.

### Antibiotic Depletion

Broad-spectrum antibiotics were administered to mice through sterile tap drinking water spiked with metronidazole (0.5 g/L), ampicillin (1 g/L), neomycin (1 g/L), and vancomycin (1 g/L). Broad-spectrum antibiotic mix was also sweetened with sucralose as described above. This was administered over 7 days and mice were monitored daily to ensure consumption.

### Metabolomics: Untargeted High-Resolution LC-HRMS

#### Sample Preparation-Mouse Stool

Taconic mice were given sucralose-containing or regular water for 14 days prior to tumor injection (MC38) and were maintained on the same water regimen for the remainder of the experiment. Mice were treated with 200 μg anti–PD-1 on day 12, and stool, serum, and TIF were collected 14 days after tumor injection.

To isolate TIF, tumors were harvested into an empty 15-mL conical tube on ice. A single slice was made in each tumor, penetrating 50% of the tissue to allow for better fluid extraction. Tumors were then placed on a 5-μm nylon membrane in an empty Eppendorf or 15-mL tube and spun at 3000 rpm for 60 minutes at 4°C. Fluid was immediately collected and frozen at –80°C until analysis.

Metabolic quenching and polar metabolite pool extraction were performed by adding ice-cold 80% methanol (aqueous) at a ratio of 1:15 wt input tissue:vol. (D_3_)-creatinine, (D_4_)-taurine, (D_3_)-lactate, and (D_3_)-alanine (Sigma-Aldrich) were added to the sample lysates as an internal standard for a final concentration of 10 µmol/L. Samples were homogenized using an MP Bio FastPrep system using Matrix D (ceramic sphere) for 60 seconds at 60 hz. The supernatant was then cleared of protein by centrifugation at 16,000× *g*. Then 2 µL of cleared supernatant was subjected to online LC-MS analysis.

#### LC-HRMS Method

Analyses were performed by untargeted Liquid Chromatography-High Resolution Mass Spectrometry (LC-HRMS). Briefly, samples were injected via a Thermo Vanquish UHPLC and separated over a reversed phase Thermo HyperCarb porous graphite column (2.1 × 100 mm, 3 μm particle size) maintained at 55°C. For the 20-minute LC gradient, the mobile phase consisted of the following: solvent A (water/0.1% FA (formic acid)) and solvent B (ACN (acetonitrile)/0.1% FA). The gradient was the following: 0 to 1 minute 1% B, increase to 15% B over 5 minutes, continue increasing to 98% B over 5 minutes, hold at 98% B for 5 minutes, and re-equilibrate at 1% B for 5 minutes. The Thermo IDX tribrid mass spectrometer was operated in both positive and ion mode, scanning in ddMS^2^ mode (2 μscans) from 70 to 800 m/z at 120,000 resolution with an AGC target of 2e5 for full scan, 2e4 for ms^2^ scans using HCD fragmentation at stepped 15,35,50 collision energies. Source ionization setting was 3.0 and 2.4 kV spray voltage, respectively, for positive and negative mode. Source gas parameters were 35 sheath gas, 12 auxiliary gas at 320°C, and eight sweep gas. Calibration was performed prior to analysis using the Pierce FlexMix Ion Calibration Solutions (Thermo Fisher Scientific). Integrated peak areas were then extracted manually using Quan Browser (Thermo Fisher Xcalibur ver. 2.7). Untargeted differential comparisons were performed using Compound Discoverer 3.0 (Thermo Fisher Scientific) to generate a ranked list of significant compounds with tentative identifications from BioCyc, Kyoto Encyclopedia of Genes and Genomes, and internal compound databases. Purified standards were then purchased and compared in retention time and m/z, along with ms2 fragmentation patterns, to validate the identity of significant hits.

### Stool Metabolomics: 3NP-Short Chain Fatty Acids and Tricarboxylic Acids

#### Sample Preparation

Mouse stool samples were homogenized with 50% aqueous acetonitrile at a ratio of 1:15 vol:wt. Then 5 µg/mL deuterated internal standards (D_2_)-formate, (D_4_)-acetate, (D_5_)-butyrate, (D_6_)-propionate, (D_2_)-valerate and (D_4_)-hexanoate, and (D_3_)-lactate (CDN Isotopes) were added. Samples were homogenized using a FastPrep-24 system (MP-Bio), with Matrix D at 60 hz for 30 seconds, before being cleared of protein by centrifugation at 16,000 × *g*. Then 60 µL cleared supernatants were collected and derivatized using 3-nitrophenylhydrazine. Each sample was mixed with 20 µL of 200 mmol/L 3-nitrophenylhydrazine in 50% aqueous acetonitrile and 20 µL of 120 mmol/L N-(3-dimethylaminopropyl)-N0-ethylcarbodiimide −6% pyridine solution in 50% aqueous acetonitrile. The mixture was reacted at 50°C for 40 minutes and the reaction was stopped with 0.45 mL of 50% acetonitrile.

#### LC-MS Analysis

Derivatized samples were injected (50 µL) via a Thermo Vanquish UHPLC and separated over a reversed phase Phenomenex Kinetex 150 mm × 2.1 mm 1.7 μm particle C18 maintained at 55°C. For the 20-minute LC gradient, the mobile phase consisted of the following: solvent A (water/0.1% FA) and solvent B (ACN/0.1% FA). The gradient was the following: 0 to 2 minutes 15% B, increase to 60% B over 10 minutes, continue increasing to 100% B over 1 minute, hold at 100% B for 3 minutes, and re-equilibrate at 15% B for 4 minutes. The Thermo IDX tribrid mass spectrometer was operated in both positive ion mode, scanning in ddMS2 mode (2 μscans) from 75 to 1,000 m/z at 120,000 resolution with an AGC target of 2e5 for full scan, 2e4 for ms2 scans using HCD fragmentation at stepped 15,35,50 collision energies. Source ionization setting was 3.0 kV spray voltage for positive mode. Source gas parameters were 45 sheath gas, 12 auxiliary gas at 320°C, and three sweep gas. Calibration was performed prior to analysis using the Pierce FlexMix Ion Calibration Solutions (Thermo Fisher Scientific). Integrated peak areas were then extracted manually using Quan Browser (Thermo Fisher Xcalibur ver. 2.7). SCFA and TCA are reported as the area ratio of SCFA or TCA to the internal standard (66).

### Shallow Shotgun Sequencing

Stool pellets were collected from each mouse at day 0, 14, 28, and 38 of sucralose treatment. Each pellet was collected into a sterile microcentrifuge tube and stored at −80°C prior to gDNA extraction with a Qiagen QIAamp Fast DNA Stool Mini Kit. After gDNA isolation, DNA was checked for concentration and purity before shipping to Microbiome Insights where paired-end sequencing (150 bp × 2) was done on a NovaSeq 6000 instrument.

#### Microbiome Taxonomic Analysis

Metagenomic sequencing data were processed using bioBakery KneadData (http://huttenhower.sph.harvard.edu/kneaddata, RRID: SCR_016596), which removed adapters and low-quality reads with Trimmomatic (67) and repetitive sequences with TRF (68), as well as non-bacterial reads using Bowtie 2 (69) by aligning against the human reference genome GRCh38 and the mouse reference genome GRCm39. MetaPhlAn 4 (70) was run using the cleaned microbial reads to assign taxonomic classifications with absolute abundances using the “-t rel_ab_w_read_stats” option. The native bioBakery SGB taxonomy assignments were converted to GTDB taxonomy (71) form using the sgb_to_gtdb.py script provided by the bioBakery developers. To maintain absolute abundance data, the taxonomy conversion script was modified as it only natively worked with relative abundance. MicrobiotaProcess (72) was used for downstream analysis and visualization of the absolute abundance data with GTDB taxonomy assignments. Specifically, MicrobiotaProcess was used to calculate α diversity, β diversity, principal coordinate analysis of β diversity, and cladogram plotting. Visualizations not created by MicrobiotaProcess were done using ggplot2 (73). Group-wise comparisons for the species level were made between each treatment group relative to the control group at each timepoint using MaAsLin2 (74).

#### Microbiome Functional Profiling

The cleaned microbial reads were functionally profiled using HUMAnN 3 (75), which utilizes pan-genomes detected by MetaPhlAn to annotate reads using UniRef90 (76) gene family clusters. These UniRef90 gene family annotations are then further grouped into MetaCyc pathways (77). The output pathway abundance was normalized to copies per million using the human_renorm_table script provided by the bioBakery developers. Normalized output was analyzed using MaAsLin2 within the microeco R package to utilize the full hierarchical pathway structure for MetaCyc (78). Group-wise comparisons were conducted across all timepoints and groups relative to day 0 and the control group, respectively. Significant pathways were plot using ggplot2.

### Single-Cell and Bulk RNA Sequencing *ex Vivo*

#### Sample Prep

C57BL/6 mice sourced from Taconic were treated with sucralose 14 days prior to tumor injection with MC38. Mice were implanted with 250,000 MC38 cells on day 0 and treated with anti–PD-1 antibody on day 9 and 12 after implantation. Three mice from each of the sucralose + anti–PD-1 and anti–PD-1 groups were sacrificed on day 14. tdLN (right inguinal) and MC38 tumors were harvested and processed into single-cell suspensions. For bulk, single-cell suspensions were stained and sorted on the Bigfoot Cell Sorter. CD8^+^ T cells and CD4^+^ T cells were sorted out separately (up to 1,000 cells per sample) based on live^+^, CD90.2^+^, TCRb^+^, and CD4^+^ or CD8^+^. CD8^+^ T cells were further sorted out into CD44^hi^ and CD44^hi^ RPL18^+^. Cells were then placed into buffer in a 96 well plate for storage for further analysis. For single cell, cell suspensions from tumor and lymph node were washed and labeled with 10x Genomics TotalSeq-C hashtags supplied by the University of Pittsburgh Sequencing Core as explained in the protocol provided by 10x Genomics.

#### Preprocessing Data and Clustering

We used CellRanger-7.0.0 to process the raw reads. Subsequent data processing, filtering, clustering, and reduced dimensionality projections were all carried out using the Seurat package version 4.4.0 (79) in R 4.3.0. Both the tdLN and tumor data were processed using the same pipeline. Processing of the datasets involved the filtering of cells with fewer than 100 genes expressed and cells with % of mitochondrial genes greater than 20%. Genes were also filtered based on the percentage of cells in which they were expressed. Finally, doublets and negatives, as identified by the Seurat function HTODemux, were taken out of the dataset. Overall, for subsequent analyses, we used 34,954 tdLN cells and 41,850 tumor cells.

To identify clusters in the dataset, we performed a K-nearest neighbor graph–based clustering as implemented in Seurat. Default parameters were applied throughout our analyses. All dimensionality reduction and projections were performed using Seurat, principal component analysis was run using default parameters, and Uniform Manifold Approximation and Projection and t-distributed stochastic neighbor embedding were computed using the 10 first components of the principal component analysis.

#### SLIDE Analyses

We used SLIDE (33), an interpretable machine learning approach that relies on latent factor regression with strong statistical guarantees, to infer transcriptomic differences across mice with and without exposure to sucralose. We built cell-specific SLIDE models for CD4^+^ T_conv_, CD8^+^ T cells, and Tregs from the tdLN and CD4^+^ T_conv_ and CD8^+^ T cells from the tumor. Clusters were annotated with cell types using known marker genes – CD4^+^ T_conv_ clusters were CD4^+^CD25^−^CD62L^+^CD44^−^, CD8^+^ T cells clusters were CD3^+^, CD5^+^, CD8^+^, CD27^+^, and CD28^+^, and Treg clusters were CD4^+^CD25^+^. Overall, five models were built. Delta and spec were tuned using grid search; for other parameters, default values were used.

SLIDE takes cell × gene matrices as inputs. The inputs to the three dLN models were 3,308 cells × 3,002 genes (CD8^+^ T cells), 12,039 cells × 3,032 genes matrix (for CD4^+^ T_conv_), and 1,242 cells × 3,028 genes (Tregs). For the two tumor models, the inputs were 7,276 cells × 3,346 genes (CD8^+^ T cells) and 299 cells × 6,079 genes (for CD4^+^ T_conv_). Latent factor discovery and identification of significant latent factors were performed as described in SLIDE (33).

To rigorously evaluate the performance of the SLIDE models, we used a k-fold cross-validation (k-fold CV) framework with multiple replicates and permutation testing. Briefly, replicate k-fold CV assesses the robustness of the model with data held out. It extends the basic k-fold CV method by conducting the entire k-fold process multiple times, with each iteration (replication) involving a new random partitioning of the dataset into *k* distinct folds. In each of these folds, k-1 folds are used for training the model, and 1-fold for testing it, ensuring that every data point is in the test fold exactly once (for each replicate). This process helps assess model performance on data held out (an overfit model would only do well on training but not test data). Additionally, model significance is assessed using permutation testing. This involves shuffling the labels of the dataset in a matched cross-validation framework. By comparing actual model performance against this empirical null derived from models built using shuffled labels, we assess the significance of the models (exact *P* values calculated using permutation testing). We used 10 replicates of 10-fold CV for the tdLN models, and 20 replicates of 10-fold CV for the tumor models.

### Bulk RNA Sequencing from *in vitro* Cell Cultures

RNA was isolated from day 7 *in vitro* cell cultures using Qiagen RNeasy Mini Kits. After RNA isolation, libraries were prepared using the Illumina stranded mRNA Preps. Sequencing was performed using an Illumina NextSeq 2000 Sequencing System. After sequencing, reads were aligned to the mm10 genome assembly using STAR alignment. Reads were quantified to an annotation model with Partek Flow using Partek E/M quantitation. Counts were then normalized by median ratio, and differential expression analysis was performed using DESeq2.

### Statistics

Statistical analyses were performed in GraphPad Prism v10. Data are presented as the mean ± SEM. Statistical significance was determined using a Student *t* test when comparing two groups, one-way ANOVA when comparing more than two groups, or a two-way ANOVA when comparing more than two groups over time. For more detail on significance, please see figure legends.

## Supplementary Material

Supplementary Tables S1-4 Supplementary Tables S1-4 show univariate and multivariate analysis of patient data.

Supplementary Fig S1 Supplementary Fig S1 shows the enrollment and dietary data collected from patients receiving ICI therapy for advanced melanoma, advanced NSCLC, and neoadjuvant melanoma patients.

Supplementary Fig S2 Supplementary Fig S2 shows the normalized intake of artificial sweeteners as well as overall response rate and progression free response rates.

Supplementary Fig S3 Supplementary Fig S3 shows overall response rate and progression free response rate for patients consuming aspartame or saccharin.

Supplementary Fig S4 Supplementary Fig S4 shows the individual tumor growth curves from mice consuming artificial sweeteners, overall survival, and tumor area. It also shows tumor growth curves for mice that were cohoused.

Supplementary Fig S5 Supplementary Fig S5 shows SLIDE analysis of CD8 T cells and CD4 T cells from the dLN and tumor of mice consuming sucralose or regular drinking water.

Supplementary Fig S6 Supplementary Fig S6 shows volcano plots, heatmaps, and pathway enrichment of CD45+ cells from the tumor and dLN.

Supplementary Fig S7 Supplementary Fig S7 shows flow cytometry of T cells isolated from ndLN, dLN, and tumor of mice consuming sucralose in both tumor models and chronic LCMV infection.

Supplementary Fig S8 Supplementary Fig S8 shows T cell proliferation, function, and metabolism following culture in sucralose.

Supplementary Fig S9 Supplementary Fig S9 shows T cell proliferation and metabolism following sucralose culture during activation, expansion, or the entirety of culture.

Supplementary Fig S10 Supplementary Fig S10 shows a western blot assessing TCR signaling of T cells cultured in sucralose.

Supplementary Fig S11 Supplementary Fig S11 shows a volcano plot and pathway enrichment of T cells cultured in sucralose during activation and expansion.

Supplementary Fig S12 Supplementary Fig S12 shows principle coordinate analysis and beta diversity of stool samples from mice consuming sucralose or regular drinking water.

Supplementary Fig S13 Supplementary Fig S13 shows beta diversity and heatmaps of stool from mice consuming sucralose. It also shows metabolomics readouts of stool of these mice as well as tumor growth curves of mice consuming sucralose with or without arginine or citrulline supplementation.

Appendix 1 Appendix 1 shows the IRB approval for HCC 20-019.

Appendix 2 Appendix 2 shows the full protocol for HCC 20-019.

Appendix 3 Appendix 3 shows the IRB approval for HCC 17 to 169.

Appendix 4 Appendix 4 shows the full protocol for HCC 17 to 169.

## Data Availability

The datasets generated are available at Gene Expression Omnibus for bulk RNA sequencing on CD8^+^ T cells after activation and expansion in sucralose and single-cell RNA sequencing on CD45^+^ cells isolated from tumor and tdLN of mice consuming sucralose-containing water or regular water (GSE260936). Codebase is available on GitHub (https://github.com/jishnu-lab/SucralosePD1). Shallow shotgun data are deposited at Sequence Read Archive (PRJNA1090098).
