# Supplementary material for: Sucralose Consumption Ablates Cancer Immunotherapy Response through Microbiome Disruption
Source: Cancer Discov. 2025 Jul 30;15(11):2278–97. doi: 10.1158/2159-8290.CD-25-0247 (PMC12580791; doi:10.1158/2159-8290.CD-25-0247)
Supplement: Supplementary Tables S1-4 — show univariate and multivariate analysis of patient data. [file cd-25-0247_supplementary_tables_s1-4_suppst1.pdf]

Univariate and multivariate model evaluating covariates and ORR in advanced melanoma patients treated with anti-PD-1 ICI singly or in combination				
**Variable**	**Univariate**		**Multivariate**	
	**Odds Ratio (95% CI)**	**P-value (Chi square test with Cox model)**	**Odds Ratio (95% CI)**	**P-value (Chi square test with Cox model)**
**Sucralose intake (high vs. low)**	0.40 (0.14, 1.16)	0.0917	0.28 (0.085, 0.924)	0.0366
**Ace-K intake (high vs. low)**	0.35 (0.12, 0.98)	0.0452	N/A	N/A
**Energy intake (kcal, continuous)**	1.00 (1.00, 1.00)	0.1643	N/A	N/A
**Weight (continuous, kg)**	0.97 (0.97, 1.00)	0.0437	0.968 (0.94, 0.997)	0.0296
**History of Hypertension (yes vs. no)**	0.50 (0.20, 1.21)	0.1215	0.38 (0.143. 1.011)	0.0525
**BMI (continuous, kg/m^2)**	0.94 (0.86, 1.02)	0.1375	N/A	N/A
**Pre-treatment LDH (elevated vs. normal, IU/L)**	0.91 (0.28, 2.90)	0.8722	N/A	N/A
**Pre-treatment NLR (≥4 vs. <4)**	1.37 (0.56, 3.37)	0.4904	N/A	N/A
**Soluble dietary fiber (continuous, g)**	1.13 (0.97, 1.31)	0.1208	N/A	N/A
**Insoluble dietary fiber (continuous, g)**	1.02 (0.95, 1.08)	0.6453	N/A	N/A
**Combination ICI therapy (vs. monotherapy)**	0.65 (0.23, 1.86)	0.4189	N/A	N/A

Supplementary Table 1. Univariate and multivariate models evaluating ORR of ICI-treated advanced melanoma (n=91). Univariate logistic regression models were used to test the association of clinical response with each variable. Multivariate logistic regression models were built using stepwise model selection procedure where sucralose intake was forced in the model. Inclusion or removal of candidate predictive factors were based on Chi-squared test p-values with thresholds for entry (0.35) and removal (0.10).

Univariate and multivariate model evaluating covariates and ORR in advanced NSCLC patients treated with anti-PD-1 ICI singly or in combination with chemotherapy				
Variable	Univariate		Multivariate	
	Odds Ratio (95% CI)	P-value (Chi square test with Cox model)	Odds Ratio (95% CI)	P-value (Chi square test with Cox model)
**Sucralose intake (high vs. low)**	0.25 (0.06, 1.00	0.0499	N/A	N/A
**Ace-K intake (high vs. low)**	0.42 (0.10, 1.70)	0.2233	N/A	N/A
**Energy intake (kcal, continuous)**	1.000 (0.999, 1.000)	0.5056	N/A	N/A
**Weight (continuous, kg)**	0.98 (0.96, 1.02)	0.3267	N/A	N/A
**Smoking status (yes vs. no)**	0.75 (0.12, 4.66)	0.7576	N/A	N/A
**BMI (continuous, kg/m^2)**	0.93 (0.83, 1.04)	0.202	N/A	N/A
**History of Hypertension (yes vs. no)**	0.37 (0.10, 1.34)	0.1288	N/A	N/A
**Pre-treatment LDH (elevated vs. normal, IU/L)**	1.70 (0.43, 6.72)	0.4494	N/A	N/A
**Pre-treatment NLR (≥4 vs. <4)**	0.97 (0.25, 3.73)	0.9599	N/A	N/A
**Soluble dietary fiber (continuous, g)**	0.90 (0.76, 1.07)	0.2361	N/A	N/A
**Insoluble dietary fiber (continuous, g)**	0.93 (0.85, 1.01)	0.102	N/A	N/A
**Combination ICI therapy (vs. monotherapy)**	0.32 (0.07, 1.38)	0.1267	N/A	N/A
**PD-L1 (1% vs. >1%)**	0.72 (0.18, 2.84)	0.6413	N/A	N/A
**TMB (continuous, Mut/Mb)**	0.99 (0.87, 1.12)	0.8692	N/A	N/A

NOTE: A multivariate model was not generated for this comparison as based on the variable section method described in Methods, we could not find a we could not find a multivariariate logistic model with at least two variables in the model.

Supplementary Table 2. Univariate and multivariate models evaluating ORR of ICI-treated advanced NSCLC (n=41). Univariate logistic regression models were used to test the association of clinical response with each variable. Multivariate logistic regression models were built using stepwise model selection procedure where sucralose intake was forced in the model. Inclusion or removal of candidate predictive factors were based on Chi-squared test p-values with thresholds for entry (0.35) and removal (0.10).

Univariate and multivariate model evaluating covariates and MPR in High Risk Resectable Melanoma treated with anti-PD-1 ICI and intratumoral TLR9 agonist vidutolimod				
	**Univariate**		**Multivariate**	
**Variable**	**Odds Ratio (95% CI)**	**P-value (Chi square test with Cox model)**	**Odds Ratio (95% CI)**	**P-value (Chi square test with Cox model)**
**Sucralose intake (high vs. low)**	0.00 (0.00, 0.00)	0.9546	N/A	N/A
**Ace-K intake (high vs. low)**	0.11 (0.01, 1.17)	0.0671	N/A	N/A
**Energy intake (kcal, continuous)**	1 (0.99, 1.02)	0.8181	N/A	N/A
**Weight (continuous, kg)**	0.99 (0.95, 1.03)	0.7066	N/A	N/A
**Smoking status (yes vs. no)**	0.33 (0.06, 1.75)	0.1944	N/A	N/A
**BMI (continuous, kg/m^2)**	0.95 (0.84, 1.08)	0.4614	N/A	N/A
**History of Hypertension (yes vs. no)**	3.50 (0.50, 22.30)	0.1849	N/A	N/A
**Pre-treatment LDH (elevated vs. normal, IU/L)**	1.00 (0.98, 1.03)	0.7347	N/A	N/A
**Pre-treatment NLR (≥4 vs. <4)**	2.22 (0.76, 6.49)	0.1458	N/A	N/A
**Soluble dietary fiber (continuous, g)**	0.92 (0.68, 1.25)	0.5881	N/A	N/A
**Insoluble dietary fiber (continuous, g)**	0.98 (0.83, 1.15)	0.8039	N/A	N/A

NOTE: A multivariate model was not generated for this comparison as based on the variable section method described in Methods, we could not find a we could not find a multivariariate logistic model with at least two variables in the model.

Supplementary Table 3. Univariate models evaluating MPR High Risk Resectable Melanoma treated with ICI supplemented with TLR Agonist (n=25). Univariate logistic regression models were used to test the association of clinical response with each variable. Inclusion or removal of candidate predictive factors were based on Chi-squared test p-values with thresholds for entry (0.35) and removal (0.10).

a

Univariate and multivariate model evaluating covariates and PFS in advanced melanoma patients treated with anti-PD-1 ICI singly or in combination				
**Variable**	**Univariate**		**Multivariate**	
	**Hazard Ratio (95% CI)**	**P-value (Chi square test with Cox model)**	**Hazard Ratio Ratio (95% CI)**	**P-value (Chi square test with Cox model)**
**Sucralose intake (high vs. low)**	2.21 (1.02, 4.79)	0.0449	2.91 (1.22, 6.96)	0.0164
**Ace-K intake (high vs. low)**	2.74 (1.29, 5.83)	0.0087	N/A	N/A
**Energy intake (kcal, continuous)**	1.00 (1.000, 1.001)	0.0084	1.001 (1.000, 1.001)	0.0002
**Weight (continuous, kg)**	1.02 (0.99, 1.04)	0.1465	N/A	N/A
**History of Hypertension (yes vs. no)**	1.85 (0.91, 3.79)	0.0914	2.68 (1.18, 6.06)	0.0178
**BMI (continuous, kg/m2)**	1.03 (0.96, 1.10)	0.4009	N/A	N/A
**Pre-treatment LDH (elevated vs. normal, IU/L)**	0.85 (0.35, 2.06)	0.7128	N/A	N/A
**Pre-treatment NLR (≥4 vs. <4)**	0.93 (0.46, 1.88)	0.8327	N/A	N/A
**soluble dietary fiber (continuous, g)**	0.95 (0.85, 1.07)	0.4109	0.80 (0.68, 0.95)	0.008
**Insoluble dietary fiber (continuous, g)**	1.01 (0.96, 1.05)	0.8286	N/A	N/A
**Combination ICI therapy (vs. monotherapy)**	2.09 (0.85, 5.11)	0.1064	2.59 (1.03, 6.53)	0.0433

b

Univariate and multivariate model evaluating covariates and PFS in advanced NSCLC patients treated with anti-PD-1 ICI singly or in combination with chemotherapy				
**Variable**	**Univariate**		**Multivariate**	
	**Hazard Ratio (95% CI)**	**P-value (Chi square test with Cox model)**	**Hazard Ratio (95% CI)**	**P-value (Chi square test with Cox model)**
**Sucralose intake (high vs. low)**	2.81 (1.04, 7.59)	0.042	28.19 (0.96, 815.71)	0.0518
**Ace-K intake (high vs. low)**	2.25 (0.85, 6.06)	0.1074	N/A	N/A
**Energy intake (kcal, continuous)**	1.00 (0.999, 1.000)	0.9455	0.999 (0.998, 1.000)	0.0823
**Weight (continuous, kg)**	1.01 (0.99, 1.03)	0.5694	N/A	N/A

**Smoking status (yes vs. no)**	1.14 (0.26, 5.04)	0.8618	N/A	N/A
**BMI (continuous, kg/m2)**	1.03 (0.98, 1.10)	0.3334	1.09 (0.98, 1.20)	0.097
**History of Cardiovascular Disease (yes vs. no)**	2.20 (0.49, 9.77)	0.3004	44.72	0.0279
**Antibiotics use 3 months prior (yes vs. no)**	5.48 (1.94, 15.50)	0.0013	N/A	N/A
**Pre-treatment LDH (elevated vs. normal, IU/L)**	0.62 (0.22, 1.70)	0.3501	N/A	N/A
**Pre-treatment NLR (≥4 vs. <4)**	0.84 (0.29, 2.44)	0.7514	N/A	N/A
**Soluble dietary fiber (continuous, g)**	1.05 (0.94, 1.17)	0.3957	N/A	N/A
**Insoluble dietary fiber (continuous, g)**	1.04 (0.98, 1.10)	0.2308	N/A	N/A
**Combination ICI therapy (vs. monotherapy)**	1.76 (0.60, 5.13)	0.2997	N/A	N/A
**PD-L1 (1% vs. >1%**	0.83 (0.30, 2.35)	0.7313	0.058 (0.005, 0.663)	0.022
**TMB (continuous, Mut/Mb)**	0.99 (0.89, 1.10)	0.8305	N/A	N/A

c

**Univariate and multivariate model evaluating covariates and RFS in High Risk Resectable Melanoma treated with anti-PD-1 ICI and intratumoral TLR9 agonist vidutolimod**				
**Variable**	**Univariate**		**Multivariate**	
	**Hazard Ratio (95% CI)**	**P-value (Chi square test with Cox model)**	**Hazard Ratio Ratio (95% CI)**	**P-value (Chi square test with Cox model)**
**Sucralose intake (high vs. low)**	5.82 (1.17, 28.88)	0.0313	N/A	N/A
**Ace-K intake (high vs. low)**	4.08 (0.82, 20.22)	0.0852	N/A	N/A
**Energy intake (kcal, continuous)**	1.001 (0.99, 1.00)	0.2009	N/A	N/A
**Weight (continuous, kg)**	0.99 (0.95, 1.04)	0.7737	N/A	N/A
**History of Hypertension (yes vs. no)**	0.32 (0.04, 2.77)	0.3034	N/A	N/A
**BMI (continuous, kg/m2)**	0.99 (0.87, 1.11)	0.8075	N/A	N/A
**Pre-treatment LDH (elevated vs. normal, IU/L)**	0.99 (0.97, 1.02)	0.5508	N/A	N/A

**Pre-treatment NLR (≥4 vs. <4)**	0.30 (0.08, 1.05)	0.06	N/A	N/A
**soluble dietary fiber (continuous, g)**	0.91 (0.64, 1.30)	0.6076	N/A	N/A
**Insoluble dietary fiber (continuous, g)**	0.94 (0.78, 1.14)	0.5518	N/A	N/A

Supplementary Table 4. a, b, and c Univariate and multivariate models evaluating PFS of ICI-treated advanced melanoma (a, n=91) and advanced NSCLC (b, n=41) and RFS of high risk resectable melanoma treated with ICI supplemented with a TLR agonist (n=25). Univariate logistic regression models were used to test the association of clinical response with each variable. Multivariate logistic regression models were built using stepwise model selection procedure where sucralose intake was forced in the model. Inclusion or removal of candidate predictive factors were based on Chi-squared test p-values with thresholds for entry (0.35) and removal (0.10).
