# Supplementary material for: Sucralose Consumption Ablates Cancer Immunotherapy Response through Microbiome Disruption
Source: Cancer Discov. 2025 Jul 30;15(11):2278–97. doi: 10.1158/2159-8290.CD-25-0247 (PMC12580791; doi:10.1158/2159-8290.CD-25-0247)
Supplement: Supplementary Fig S1 — shows the enrollment and dietary data collected from patients receiving ICI therapy for advanced melanoma, advanced NSCLC, and neoadjuvant melanoma patients. [file cd-25-0247_supplementary_fig_s1_suppsf1.pdf]

Figure S1

a

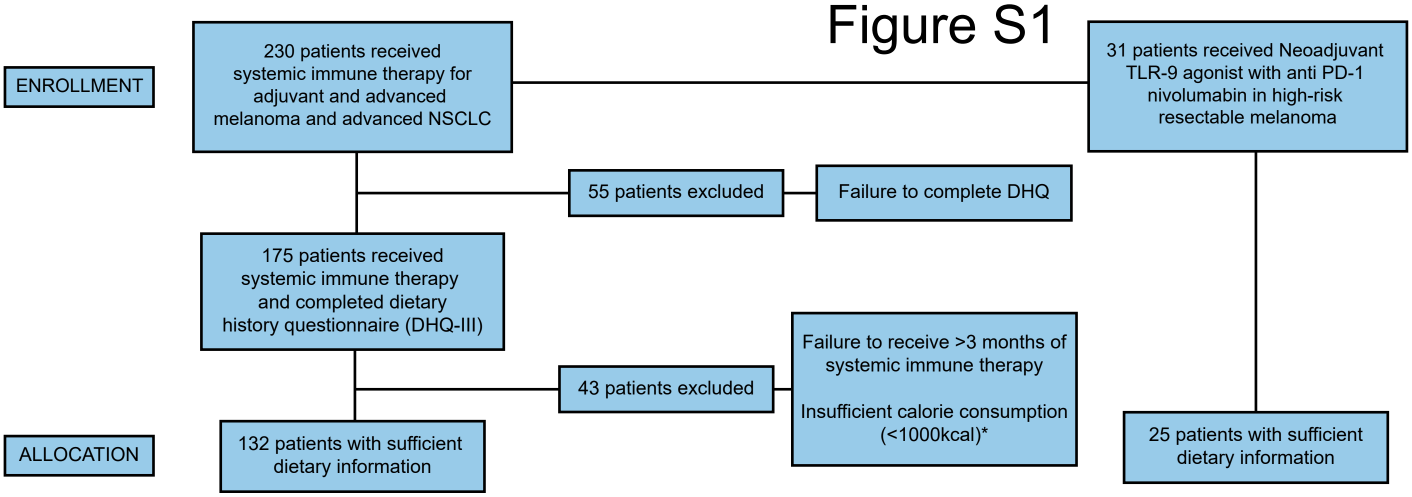

*The recommended daily calorie intake is 2000kcal/2500kcal

b

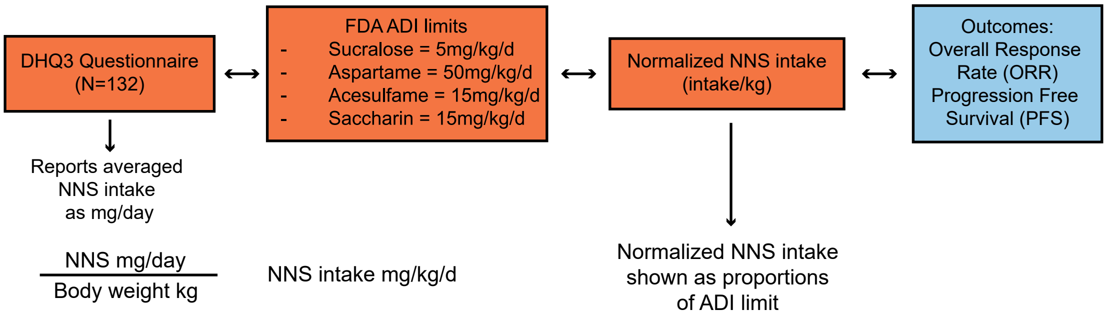

c

	**Advanced Melanoma (n=91)**			**Advanced NSCLC (n=41)**			**High Risk Resectable Melanoma (n=25)**		
Characteristic	**low intake (n=73)**	**high intake (n=18)**	p-value	**low intake (n=28)**	**high intake (n=13)**	p-value	**low intake (n=21)**	**high intake (n=4)**	p-value
Demographics									
Age at Start of Treatment, median	68.2	65.9	0.3651	69	64.9	0.6087	65.7	49.6	0.0381
Gender (male), n (%)	56 (76.7)	13 (72.2)	0.6903	11 (39.2)	6 (46.2)	0.6779	15 (71.4)	0 (0)	0.0075
Ethnicity (Not Hispanic or Latino), n (%)	63 (98.6)	18 (100)	0.6176	26 (92.8)	12 (92.3)	0.9499	21 (100)	4 (100)	0.99
BMI in kg/m^2, mean (SD)	29.5 (5.0)	29.2 (6.0)	0.659	26.5 (5.1)	27.1 (9.0)	0.6043	29.6 (6.3)	30.6 (10.4)	0.6945
Current Smoker, n (%)	22 (30.1)	8 (44.4)	0.2475	25 (89.3)	10 (76.9)	0.2973	9 (42.9)	2 (50)	0.792
History of Hypertension, n (%)	38 (52.1)	6 (33.3)	0.1546	12 (42.8)	5 (38.5)	0.7904	8 (38.1)	1 (25)	0.617
History of Cardiovascular Disease, n (%)	5 (6.8)	1 (5.5)	0.843	2 (7.14)	1 (7.7)	0.9499	2 (9.5)	0 (0)	0.5199
Treatment Response, n (%)	52 (71.3)	9 (50)	0.0861	20 (71.4)	5 (38.5)	0.044	15 (71.4)	0 (0)	0.0075
Concomitant Dietary Factors									
Total Energy (kcal/day), mean (SD)	1887 (732.0)	2158 (865.2)	0.2282	2066 (987.4)	2166 (720.3)	0.3383	1967 (530.1)	1577.9 587.5	0.2032
Soluble Dietary Fiber (g/day), mean (SD)	7.2 (3.1)	7.3 (3.2)	0.6975	8.7 (4.6)	8.5 (2.7)	0.9619	6.4 (2.8)	5.3 (1.2)	0.7478
Insoluble Dietary Fiber (g/day), mean (SD)	12.7 (7.1)	14.5 (6.6)	0.1624	14.5 (7.4)	15.3 (8.3)	0.7716	11.8 (4.9)	6.3 (1.5)	0.0381

Supplementary Figure S1. a, CONSORT diagram depicting enrollment and dietary data collection from patients receiving ICI therapy for advanced melanoma, advanced NSCLC and neoadjuvant melanoma patients. **b,** Normalized NNS intake (mg/kg/day) by NNS category to evaluate intake relative to US FDA acceptable daily intake (ADI) levels. **c,** Characteristics of patients used within this study stratified by high and low sucralose intake.
