# Supplementary material for: Sucralose Consumption Ablates Cancer Immunotherapy Response through Microbiome Disruption
Source: Cancer Discov. 2025 Jul 30;15(11):2278–97. doi: 10.1158/2159-8290.CD-25-0247 (PMC12580791; doi:10.1158/2159-8290.CD-25-0247)
Supplement: Supplementary Fig S2 — shows the normalized intake of artificial sweeteners as well as overall response rate and progression free response rates. [file cd-25-0247_supplementary_fig_s2_suppsf2.pdf]

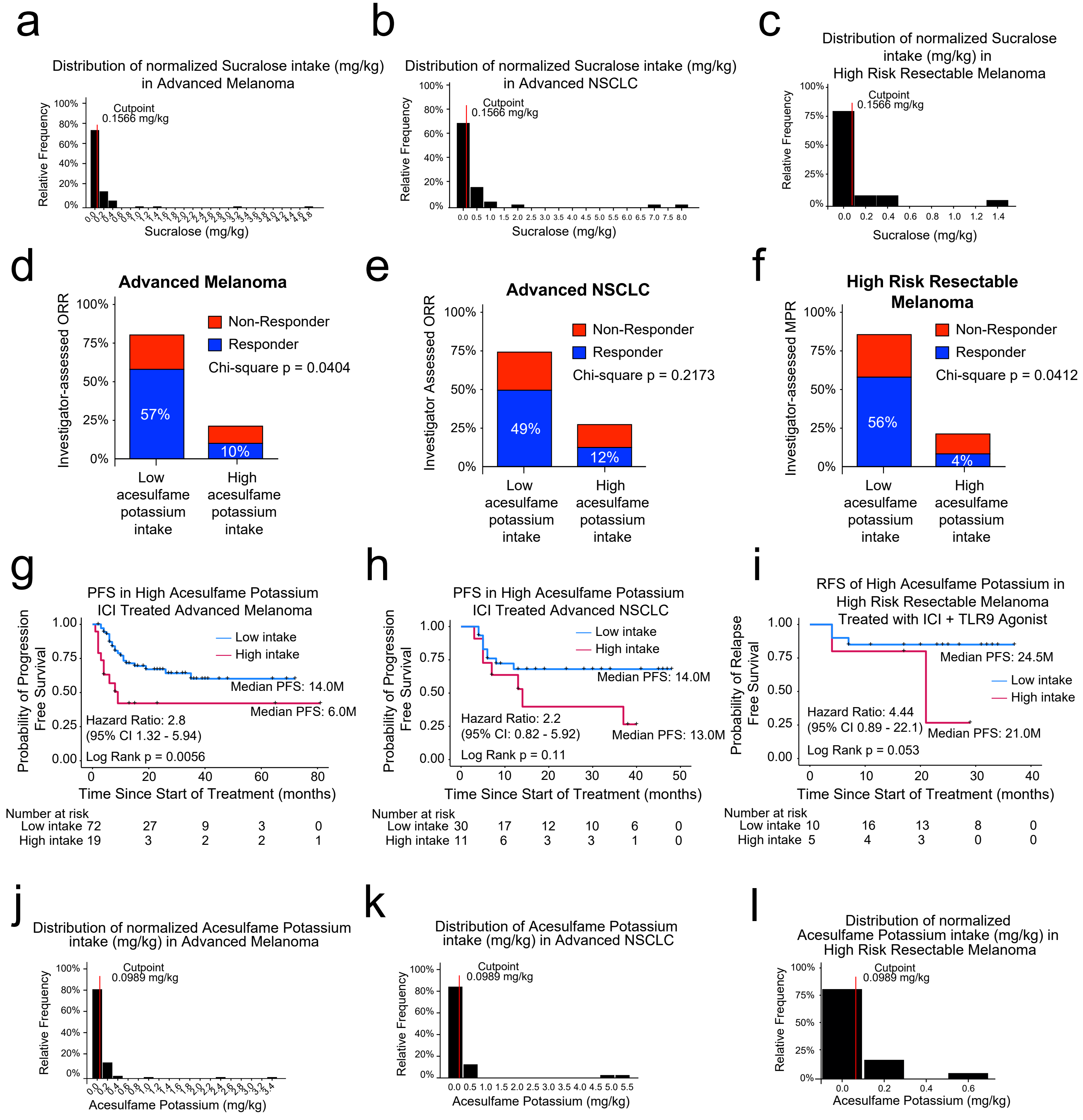

a
Distribution of normalized Sucralose intake (mg/kg) in Advanced Melanoma
Cutpoint 0.1566 mg/kg
Relative Frequency
Sucralose (mg/kg)
b
Distribution of normalized Sucralose intake (mg/kg) in Advanced NSCLC
Cutpoint 0.1566 mg/kg
Relative Frequency
Sucralose (mg/kg)
c
Distribution of normalized Sucralose intake (mg/kg) in High Risk Resectable Melanoma
Cutpoint 0.1566 mg/kg
Relative Frequency
Sucralose (mg/kg)
d
Advanced Melanoma
Investigator-assessed ORR
Non-Responder
Responder
Chi-square p = 0.0404
57%
10%
Low acesulfame potassium intake
High acesulfame potassium intake
e
Advanced NSCLC
Investigator Assessed ORR
Non-Responder
Responder
Chi-square p = 0.2173
49%
12%
Low acesulfame potassium intake
High acesulfame potassium intake
f
High Risk Resectable Melanoma
Investigator-assessed MPR
Non-Responder
Responder
Chi-square p = 0.0412
56%
4%
Low acesulfame potassium intake
High acesulfame potassium intake
g
PFS in High Acesulfame Potassium ICI Treated Advanced Melanoma
Probability of Progression Free Survival
Low intake
High intake
Median PFS: 14.0M
Median PFS: 6.0M
Hazard Ratio: 2.8
(95% CI 1.32 - 5.94)
Log Rank p = 0.0056
Time Since Start of Treatment (months)
Number at risk
Low intake 72 27 9 3 0
High intake 19 3 2 2 1
h
PFS in High Acesulfame Potassium ICI Treated Advanced NSCLC
Probability of Progression Free Survival
Low intake
High intake
Median PFS: 14.0M
Hazard Ratio: 2.2
(95% CI: 0.82 - 5.92)
Median PFS: 13.0M
Log Rank p = 0.11
Time Since Start of Treatment (months)
Number at risk
Low intake 30 17 12 10 6 0
High intake 11 6 3 3 1 0
i
RFS of High Acesulfame Potassium in High Risk Resectable Melanoma Treated with ICI + TLR9 Agonist
Probability of Relapse Free Survival
Median PFS: 24.5M
Low intake
High intake
Hazard Ratio: 4.44
(95% CI 0.89 - 22.1)
Median PFS: 21.0M
Log Rank p = 0.053
Time Since Start of Treatment (months)
Number at risk
Low intake 10 16 13 8 0
High intake 5 4 3 0 0
j
Distribution of normalized Acesulfame Potassium intake (mg/kg) in Advanced Melanoma
Cutpoint 0.0989 mg/kg
Relative Frequency
Acesulfame Potassium (mg/kg)
k
Distribution of Acesulfame Potassium intake (mg/kg) in Advanced NSCLC
Cutpoint 0.0989 mg/kg
Relative Frequency
Acesulfame Potassium (mg/kg)
l
Distribution of normalized Acesulfame Potassium intake (mg/kg) in High Risk Resectable Melanoma
Cutpoint 0.0989 mg/kg
Relative Frequency
Acesulfame Potassium (mg/kg)

Figure S2

Supplementary Figure S2. Ace-K intake is associated with poor immunotherapeutic response. Patients with advanced melanoma, advanced NSCLC, and neoadjuvant melanoma patients receiving checkpoint inhibitors completed a dietary history questionnaire (DHQ III), including artificial/non-nutritive sweetener consumption. Patients were split into high and low artificial sweetener intake. **a-c,** Histograms showing patient distribution of sucralose intake in advanced melanoma (g,j), advanced NSCLC (h,k), and high risk resectable melanoma (i,l). Cutpoint used for high and low intake is shown in red. **d-f,** Overall response rates (ORR) for (**d**) advanced melanoma or (**e**) advanced NSCLC patients consuming high or low amounts of Ace-K or (**f**) Major pathologic response (MPR) for high risk resectable melanoma patients receiving ICI and TLR9 agonist consuming high or low amounts of Ace-K. Responders are shown in blue and non-responders are shown in red. **g-i,** Progression free survival (PFS) (**g-h**) or relapse free survival (RFS) (**i**) in months (M) for patients from (**d-f**). Low Ace-K intake shown in blue and high intake shown in red. **j-l,** Histograms showing patient distribution Acesulfame potassium intake in advanced melanoma (j), advanced NSCLC (k), and high risk resectable melanoma (l). Cutpoint used for high and low intake is shown in red.
