# Supplementary material for: Sucralose Consumption Ablates Cancer Immunotherapy Response through Microbiome Disruption
Source: Cancer Discov. 2025 Jul 30;15(11):2278–97. doi: 10.1158/2159-8290.CD-25-0247 (PMC12580791; doi:10.1158/2159-8290.CD-25-0247)
Supplement: Supplementary Fig S3 — shows overall response rate and progression free response rate for patients consuming aspartame or saccharin. [file cd-25-0247_supplementary_fig_s3_suppsf3.pdf]

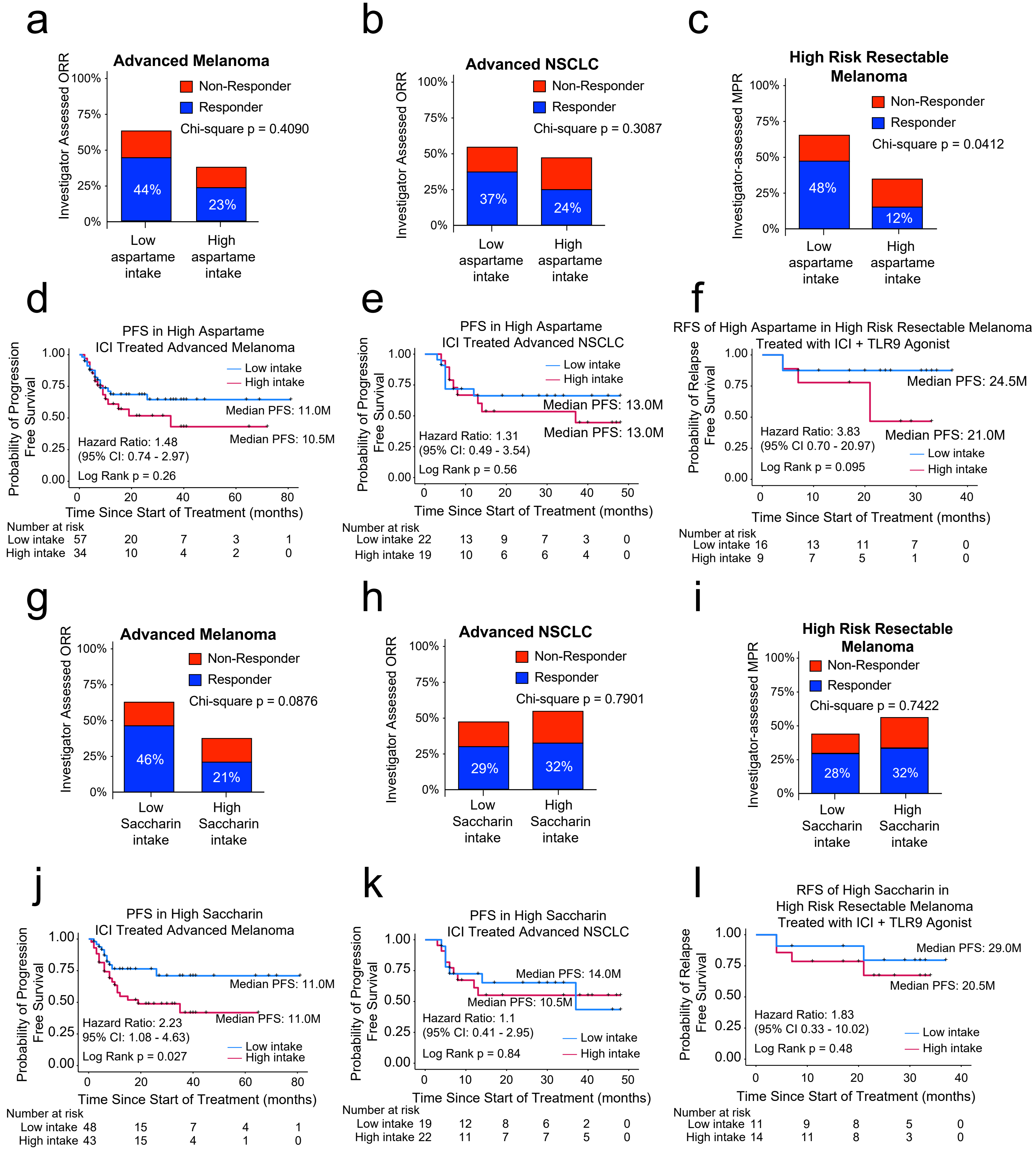
a
Advanced Melanoma
Investigator Assessed ORR
100%
75%
50%
25%
0%
Non-Responder
Responder
Chi-square p = 0.4090
44%
23%
Low aspartame intake
High aspartame intake
b
Advanced NSCLC
Investigator Assessed ORR
Non-Responder
Responder
Chi-square p = 0.3087
37%
24%
Low aspartame intake
High aspartame intake
c
High Risk Resectable Melanoma
Investigator-assessed MPR
Non-Responder
Responder
Chi-square p = 0.0412
48%
12%
Low aspartame intake
High aspartame intake
d
PFS in High Aspartame ICI Treated Advanced Melanoma
Probability of Progression Free Survival
Low intake
High intake
Median PFS: 11.0M
Median PFS: 10.5M
Hazard Ratio: 1.48
(95% CI: 0.74 - 2.97)
Log Rank p = 0.26
Time Since Start of Treatment (months)
Number at risk
Low intake 57 20 7 3 1
High intake 34 10 4 2 0
e
PFS in High Aspartame ICI Treated Advanced NSCLC
Probability of Progression Free Survival
Low intake
High intake
Median PFS: 13.0M
Median PFS: 13.0M
Hazard Ratio: 1.31
(95% CI: 0.49 - 3.54)
Log Rank p = 0.56
Time Since Start of Treatment (months)
Number at risk
Low intake 22 13 9 7 3 0
High intake 19 10 6 6 4 0
f
RFS of High Aspartame in High Risk Resectable Melanoma Treated with ICI + TLR9 Agonist
Probability of Relapse Free Survival
Median PFS: 24.5M
Median PFS: 21.0M
Hazard Ratio: 3.83
(95% CI 0.70 - 20.97)
Log Rank p = 0.095
Low intake
High intake
Time Since Start of Treatment (months)
Number at risk
Low intake 16 13 11 7 0
High intake 9 7 5 1 0
g
Advanced Melanoma
Investigator Assessed ORR
Non-Responder
Responder
Chi-square p = 0.0876
46%
21%
Low Saccharin intake
High Saccharin intake
h
Advanced NSCLC
Investigator Assessed ORR
Non-Responder
Responder
Chi-square p = 0.7901
29%
32%
Low Saccharin intake
High Saccharin intake
i
High Risk Resectable Melanoma
Investigator-assessed MPR
Non-Responder
Responder
Chi-square p = 0.7422
28%
32%
Low Saccharin intake
High Saccharin intake
j
PFS in High Saccharin ICI Treated Advanced Melanoma
Probability of Progression Free Survival
Median PFS: 11.0M
Median PFS: 11.0M
Hazard Ratio: 2.23
95% CI: 1.08 - 4.63)
Log Rank p = 0.027
Low intake
High intake
Time Since Start of Treatment (months)
Number at risk
Low intake 48 15 7 4 1
High intake 43 15 4 1 0
k
PFS in High Saccharin ICI Treated Advanced NSCLC
Probability of Progression Free Survival
Median PFS: 14.0M
Median PFS: 10.5M
Hazard Ratio: 1.1
(95% CI: 0.41 - 2.95)
Log Rank p = 0.84
Low intake
High intake
Time Since Start of Treatment (months)
Number at risk
Low intake 19 12 8 6 2 0
High intake 22 11 7 7 5 0
l
RFS of High Saccharin in High Risk Resectable Melanoma Treated with ICI + TLR9 Agonist
Probability of Relapse Free Survival
Median PFS: 29.0M
Median PFS: 20.5M
Hazard Ratio: 1.83
(95% CI 0.33 - 10.02)
Log Rank p = 0.48
Low intake
High intake
Time Since Start of Treatment (months)
Number at risk
Low intake 11 9 8 5 0
High intake 14 11 8 3 0

Figure S3

Supplementary Figure S3. Some non-nutritive sweeteners are not associated with poor immunotherapeutic response. Patients with advanced melanoma or lung cancer or high risk resectable melanoma receiving checkpoint inhibitors completed a dietary history questionnaire (DHQ III), including artificial/non-nutritive sweetener consumption. Patients were split into high and low artificial sweetener intake. **a-c,** Overall response rates (ORR) for (**a**) advanced melanoma or (**b**) advanced NSCLC patients consuming high or low amounts of aspartame or (**c**) Major pathologic response (MPR) for high risk resectable melanoma patients receiving ICI and TLR9 agonist consuming high or low amounts of aspartame. Responders are shown in blue and non-responders are shown in red. **d-f,** Progression free survival (PFS) (**d-e**) or relapse free survival (RFS) (**f**) in months (M) for patients from (**a-c**). Low aspartame intake shown in blue and high intake shown in red. **g-i,** Overall response rates (ORR) for (**a**) advanced melanoma or (**b**) advanced NSCLC patients consuming high or low amounts of saccharin or (**c**) Major pathologic response (MPR) for high risk resectable melanoma patients receiving ICI and TLR9 agonist consuming high or low amounts of saccharin. Responders are shown in blue and non-responders are shown in red. **j-l,** Progression free survival (PFS) (**j-k**) or relapse free survival (RFS) (**l**) in months (M) for patients from (**g-i**). Low saccharin intake shown in blue and high intake shown in red.
