# Supplementary material for: Sucralose Consumption Ablates Cancer Immunotherapy Response through Microbiome Disruption
Source: Cancer Discov. 2025 Jul 30;15(11):2278–97. doi: 10.1158/2159-8290.CD-25-0247 (PMC12580791; doi:10.1158/2159-8290.CD-25-0247)
Supplement: Supplementary Fig S4 — shows the individual tumor growth curves from mice consuming artificial sweeteners, overall survival, and tumor area. It also shows tumor growth curves for mice that were cohoused. [file cd-25-0247_supplementary_fig_s4_suppsf4.pdf]

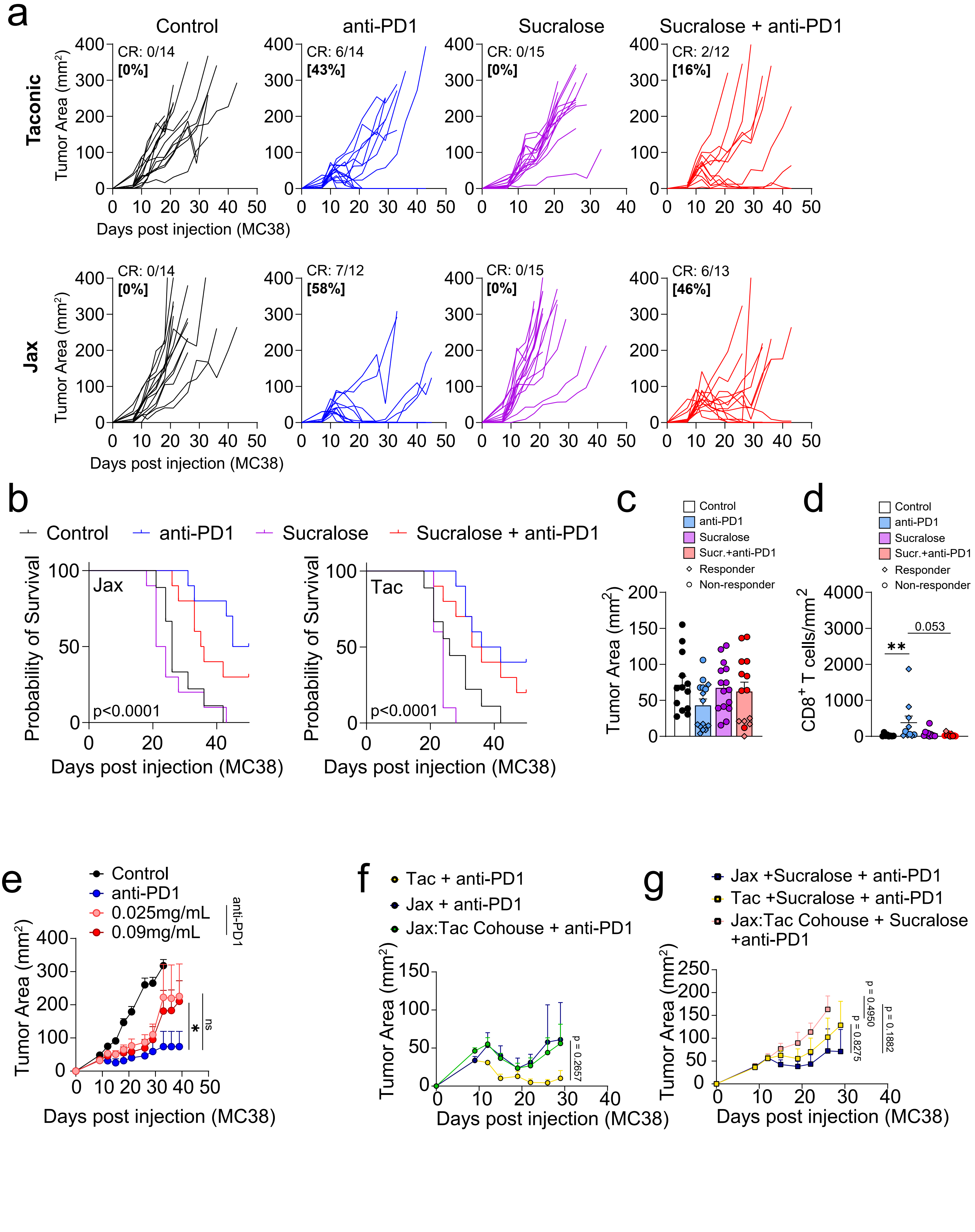
a
Control
anti-PD1
Sucralose
Sucralose + anti-PD1
Taconic
Tumor Area (mm²)
CR: 0/14 [0%]
CR: 6/14 [43%]
CR: 0/15 [0%]
CR: 2/12 [16%]
Days post injection (MC38)
Jax
CR: 0/14 [0%]
CR: 7/12 [58%]
CR: 0/15 [0%]
CR: 6/13 [46%]
b
Control
anti-PD1
Sucralose
Sucralose + anti-PD1
Probability of Survival
Jax
Tac
p<0.0001
Days post injection (MC38)
c
Control
anti-PD1
Sucralose
Sucr.+anti-PD1
Responder
Non-responder
Tumor Area (mm²)
d
CD8+ T cells/mm²
**
0.053
e
Control
anti-PD1
0.025mg/mL
0.09mg/mL
anti-PD1
Tumor Area (mm²)
*
ns
Days post injection (MC38)
f
Tac + anti-PD1
Jax + anti-PD1
Jax:Tac Cohouse + anti-PD1
Tumor Area (mm²)
p = 0.2657
Days post injection (MC38)
g
Jax +Sucralose + anti-PD1
Tac +Sucralose + anti-PD1
Jax:Tac Cohouse + Sucralose +anti-PD1
Tumor Area (mm²)
p = 0.4950
p = 0.8275
p = 0.1882
Days post injection (MC38)

Figure S4

Supplementary Figure S4. a, Tumor growth curves for C57Bl/6 mice from Taconic (top) or Jax (bottom). Mice were given sucralose-supplemented drinking water (0.09mg/mL) for 2 weeks prior to tumor injection and for the duration of the experiment. Mice were injected with $2.5x10^5$ MC38 cells subcutaneously and treated with 200µg anti-PD1 on days 9, 12, and 15. Tumor area was measured every 3 days until endpoint. **b,** Survival curves from (**a**) with mice sourced from either Jackson Labs (Jax) or Taconic (Tac). **c,** Tumor area of Taconic mice 26 days post MC38 injection. Responder mice in the anti-PD1+/- sucralose groups are marked by diamonds. **d,** CD8+ T cell infiltration per mm^2 of tumor from mice in **b**. Responder mice are marked by diamonds. **e,** Mice sourced from Jackson were treated as in (**c**) and consumed 0.09mg/mL sucralose (18 mg/kg/day, equivalent to 1.458mg/kg/day for humans based on metabolic rates) or 0.025mg/mL (5mg/kg/day, equivalent to the human ADI). **f-g,** Mice sourced from Taconic and Jackson were either housed separately (navy, yellow) or together (green, salmon) throughout sucralose consumption and tumorigenesis. Data are a composite (**a-b**) or representative of (**c-g**) 2-3 independent experiments with 4-5 mice per group per experiment. Error bars represent the mean ± SEM. two-way ANOVA (**e-g**), Mantel-Cox (**b**), or student's t test (**c-d**) were used. *p<0.05, **p<0.005
