# Supplementary material for: Sucralose Consumption Ablates Cancer Immunotherapy Response through Microbiome Disruption
Source: Cancer Discov. 2025 Jul 30;15(11):2278–97. doi: 10.1158/2159-8290.CD-25-0247 (PMC12580791; doi:10.1158/2159-8290.CD-25-0247)
Supplement: Supplementary Fig S5 — shows SLIDE analysis of CD8 T cells and CD4 T cells from the dLN and tumor of mice consuming sucralose or regular drinking water. [file cd-25-0247_supplementary_fig_s5_suppsf5.pdf]

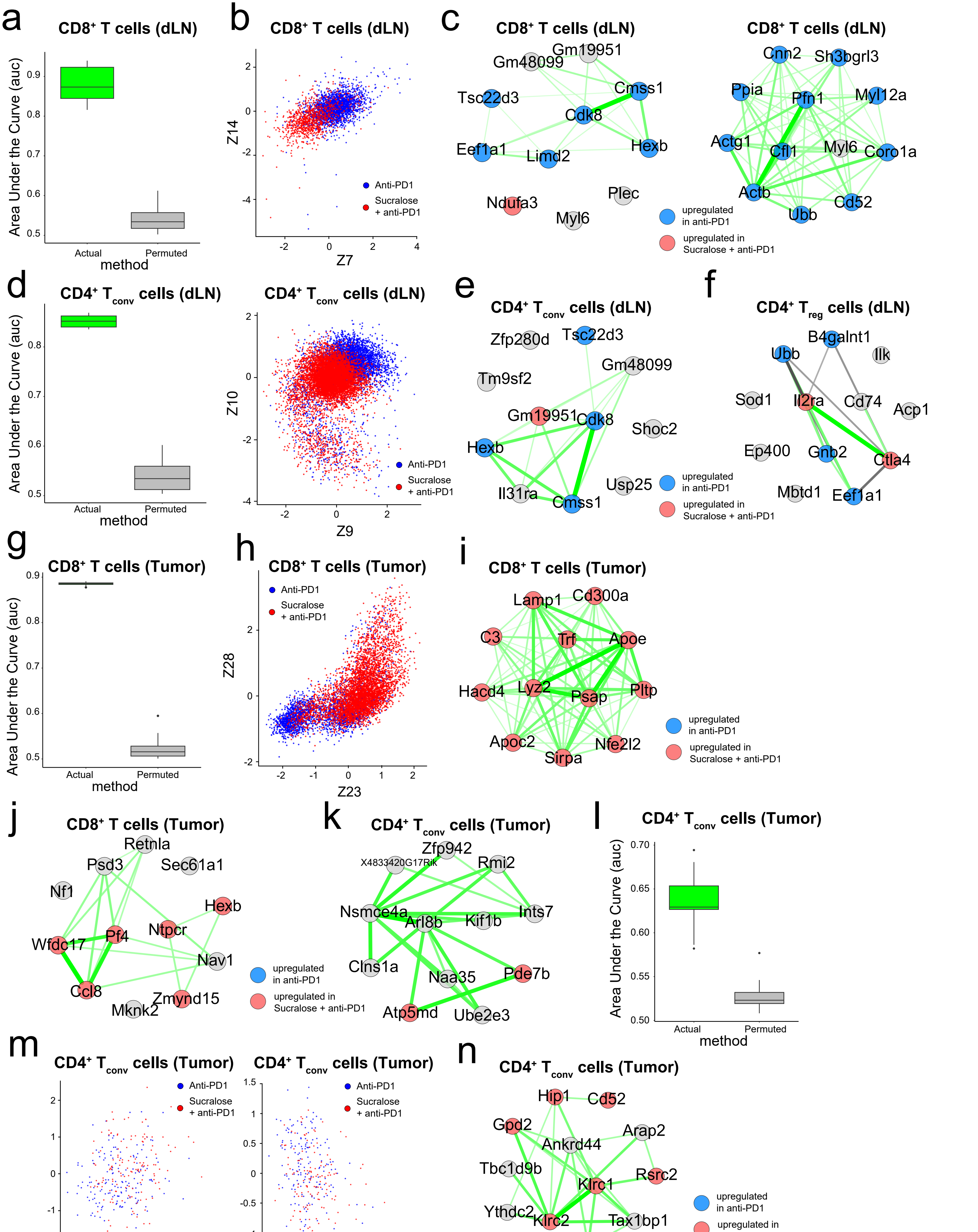
a
CD8+ T cells (dLN)
Area Under the Curve (auc)
Actual
Permuted
method
b
CD8+ T cells (dLN)
Z14
Z7
Anti-PD1
Sucralose + anti-PD1
c
CD8+ T cells (dLN)
Gm19951
Gm48099
Tsc22d3
Cmss1
Cdk8
Eef1a1
Limd2
Hexb
Ndufa3
Plec
Myl6
CD8+ T cells (dLN)
Cnn2
Sh3bgrl3
Ppia
Pfn1
Myl12a
Actg1
Cfl1
Myl6
Coro1a
Actb
Cd52
Ubb
upregulated in anti-PD1
upregulated in Sucralose + anti-PD1
d
CD4+ Tconv cells (dLN)
Area Under the Curve (auc)
Actual
Permuted
method
CD4+ Tconv cells (dLN)
Z10
Z9
Anti-PD1
Sucralose + anti-PD1
e
CD4+ Tconv cells (dLN)
Zfp280d
Tsc22d3
Gm48099
Tm9sf2
Gm19951
Cdk8
Shoc2
Hexb
Il31ra
Cmss1
Usp25
upregulated in anti-PD1
upregulated in Sucralose + anti-PD1
f
CD4+ Treg cells (dLN)
B4galnt1
Ubb
Ilk
Sod1
Il2ra
Cd74
Acp1
Ep400
Gnb2
Ctla4
Mbtd1
Eef1a1
g
CD8+ T cells (Tumor)
Area Under the Curve (auc)
Actual
Permuted
method
h
CD8+ T cells (Tumor)
Anti-PD1
Sucralose + anti-PD1
Z28
Z23
i
CD8+ T cells (Tumor)
Lamp1
Cd300a
C3
Trf
Apoe
Hacd4
Lyz2
Psap
Pltp
Apoc2
Nfe2l2
Sirpa
upregulated in anti-PD1
upregulated in Sucralose + anti-PD1
j
CD8+ T cells (Tumor)
Retnla
Psd3
Sec61a1
Nf1
Hexb
Ntpcr
Wfdc17
Pf4
Nav1
Ccl8
Zmynd15
Mknk2
upregulated in anti-PD1
upregulated in Sucralose + anti-PD1
k
CD4+ Tconv cells (Tumor)
Zfp942
X4833420G17Rik
Rmi2
Nsmce4a
Arl8b
Kif1b
Ints7
Clns1a
Naa35
Pde7b
Atp5md
Ube2e3
l
CD4+ Tconv cells (Tumor)
Area Under the Curve (auc)
Actual
Permuted
method
m
CD4+ Tconv cells (Tumor)
Anti-PD1
Sucralose + anti-PD1
CD4+ Tconv cells (Tumor)
Anti-PD1
Sucralose + anti-PD1
n
CD4+ Tconv cells (Tumor)
Hip1
Cd52
Gpd2
Arap2
Ankrd44
Tbc1d9b
Rsrc2
Klrc1
Ythdc2
Klrc2
Tax1bp1
Exoc2
upregulated in anti-PD1
upregulated in Sucralose + anti-PD1

Figure S5

Supplementary Figure S5. $CD45^+$ cells were isolated from the tumor and tumor draining lymph node (dLN) prior to single cell RNAsequencing. **a,** Spearman correlations between true and predicted $CD8^+$ T cell phenotypes in the dLN comparing the SLIDE method versus a permutated method (spec=0.1) **b,** Latent Factor (LF) scatter plot for $CD8^+$ T cells in the dLN. **c,** LF correlation network from (**b**). **d,** Spearman correlations between true and predicted $CD4^+$ T_{conv} cell phenotypes comparing the SLIDE method versus a permutated method (spec=0.05) and LF scatter plot. LF scatter plot of $CD4^+$ T_{reg} cells isolated from the dLN. **e-f,** LF correlation networks from $CD4^+$ T_{conv} (**e**) and T_{reg} (**f**) in the dLN. **g,** Spearman correlations between true and predicted $CD8^+$ T cell phenotypes in the tumor comparing the SLIDE method versus a permutated method (spec=0.1). **h,** Latent Factor (LF) scatter plot for $CD8^+$ T cells in the tumor. **i-k,** LF correlation network from (**h**). **l,** Spearman correlations between true and predicted $CD4^+$ T_{conv} cell phenotypes in the tumor comparing the SLIDE method versus a permutated method (spec=0.1). **m**, Latent Factor (LF) scatter plot for $CD4^+$ T_{conv} cells in the tumor. **n,** LF correlation network from (**m**).
