# Supplementary material for: Sucralose Consumption Ablates Cancer Immunotherapy Response through Microbiome Disruption
Source: Cancer Discov. 2025 Jul 30;15(11):2278–97. doi: 10.1158/2159-8290.CD-25-0247 (PMC12580791; doi:10.1158/2159-8290.CD-25-0247)
Supplement: Supplementary Fig S6 — shows volcano plots, heatmaps, and pathway enrichment of CD45+ cells from the tumor and dLN. [file cd-25-0247_supplementary_fig_s6_suppsf6.pdf]

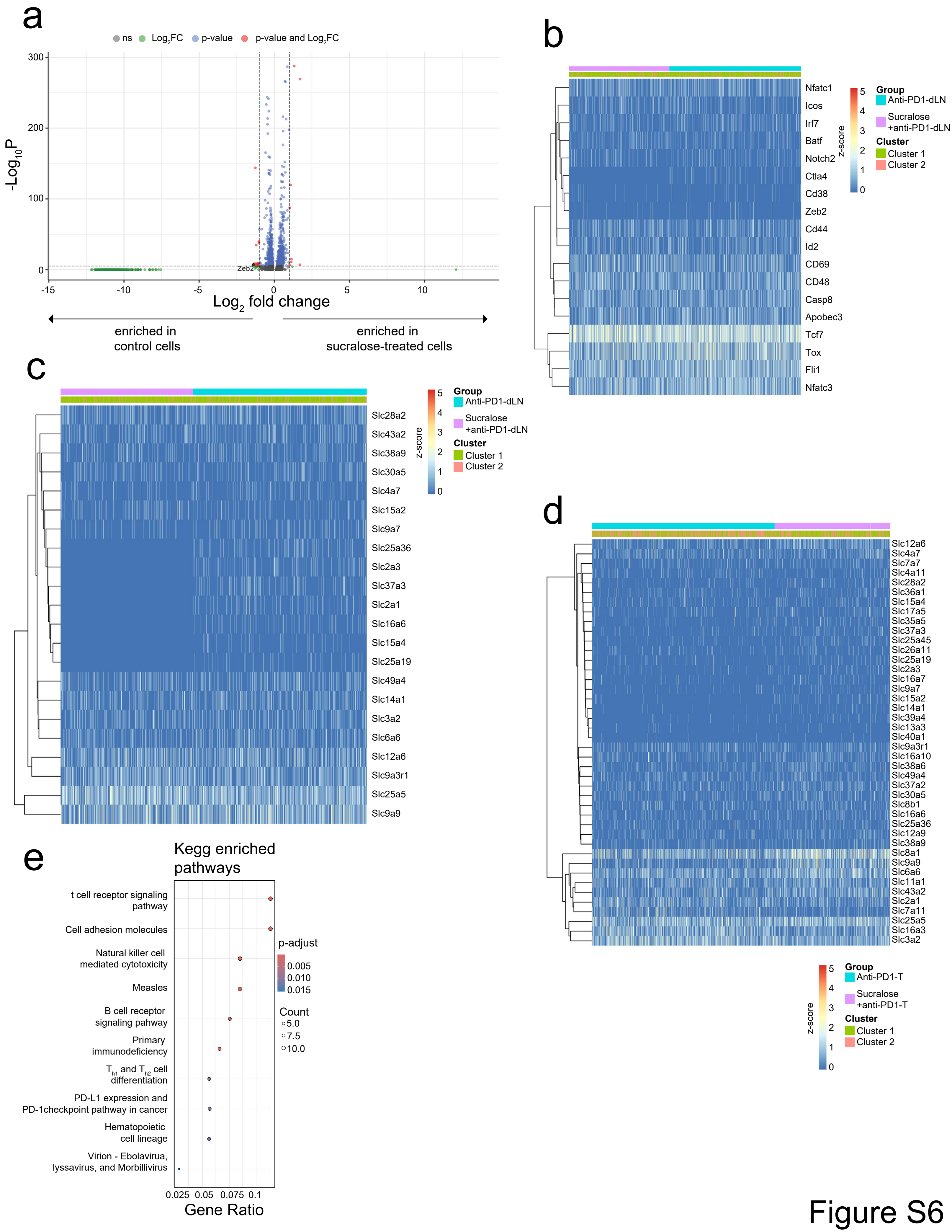

a
ns
Log2FC
p-value
p-value and Log2FC
-Log10P
300
200
100
0
Zeb2
-15
-10
-5
0
5
10
Log2 fold change
enriched in control cells
enriched in sucralose-treated cells
b
Nfatc1
Icos
Irf7
Batf
Notch2
Ctla4
Cd38
Zeb2
Cd44
Id2
CD69
CD48
Casp8
Apobec3
Tcf7
Tox
Fli1
Nfatc3
z-score
Group
Anti-PD1-dLN
Sucralose +anti-PD1-dLN
Cluster
Cluster 1
Cluster 2
c
Slc28a2
Slc43a2
Slc38a9
Slc30a5
Slc4a7
Slc15a2
Slc9a7
Slc25a36
Slc2a3
Slc37a3
Slc2a1
Slc16a6
Slc15a4
Slc25a19
Slc49a4
Slc14a1
Slc3a2
Slc6a6
Slc12a6
Slc9a3r1
Slc25a5
Slc9a9
z-score
Group
Anti-PD1-dLN
Sucralose +anti-PD1-dLN
Cluster
Cluster 1
Cluster 2
d
Slc12a6
Slc4a7
Slc7a7
Slc4a11
Slc28a2
Slc36a1
Slc15a4
Slc17a5
Slc35a5
Slc37a3
Slc25a45
Slc26a11
Slc25a19
Slc2a3
Slc16a7
Slc9a7
Slc15a2
Slc14a1
Slc39a4
Slc13a3
Slc40a1
Slc9a3r1
Slc16a10
Slc38a6
Slc49a4
Slc37a2
Slc30a5
Slc8b1
Slc16a6
Slc25a36
Slc12a9
Slc38a9
Slc8a1
Slc9a9
Slc6a6
Slc11a1
Slc43a2
Slc2a1
Slc7a11
Slc25a5
Slc16a3
Slc3a2
z-score
Group
Anti-PD1-T
Sucralose +anti-PD1-T
Cluster
Cluster 1
Cluster 2
e
Kegg enriched pathways
t cell receptor signaling pathway
Cell adhesion molecules
Natural killer cell mediated cytotoxicity
Measles
B cell receptor signaling pahway
Primary immunodeficiency
Th1 and Th2 cell differentiation
PD-L1 expression and PD-1checkpoint pathway in cancer
Hematopoietic cell lineage
Virion - Ebolavirua, lyssavirus, and Morbillivirus
p-adjust
0.005
0.010
0.015
Count
5.0
7.5
10.0
0.025
0.05
0.075
0.1
Gene Ratio

Figure S6

Supplementary Figure S6. CD45$^+$ cells were isolated from the tumor and tumor draining lymph node (dLN) prior to single cell RNAsequencing. **a,** Volcano plot of CD8+ T cells from the dLN of sucralose+anti-PD1 vs anti-PD1. Significant genes are marked in red. **b-c,** Heatmaps of **a**) showing T cell exhaustion (**b**) and SLC (**c**) gene signatures. **d,** Heatmap of SLC gene signatures in CD8+ T cells from the tumor of sucralose+anti-PD1 vs anti-PD1 groups. **e,** KEGG pathway analysis showing significant pathways of CD8+ T cells from the tumor of sucralose+anti-PD1 vs anti-PD1 groups.
