# Supplementary material for: Sucralose Consumption Ablates Cancer Immunotherapy Response through Microbiome Disruption
Source: Cancer Discov. 2025 Jul 30;15(11):2278–97. doi: 10.1158/2159-8290.CD-25-0247 (PMC12580791; doi:10.1158/2159-8290.CD-25-0247)
Supplement: Supplementary Fig S7 — shows flow cytometry of T cells isolated from ndLN, dLN, and tumor of mice consuming sucralose in both tumor models and chronic LCMV infection. [file cd-25-0247_supplementary_fig_s7_suppsf7.pdf]

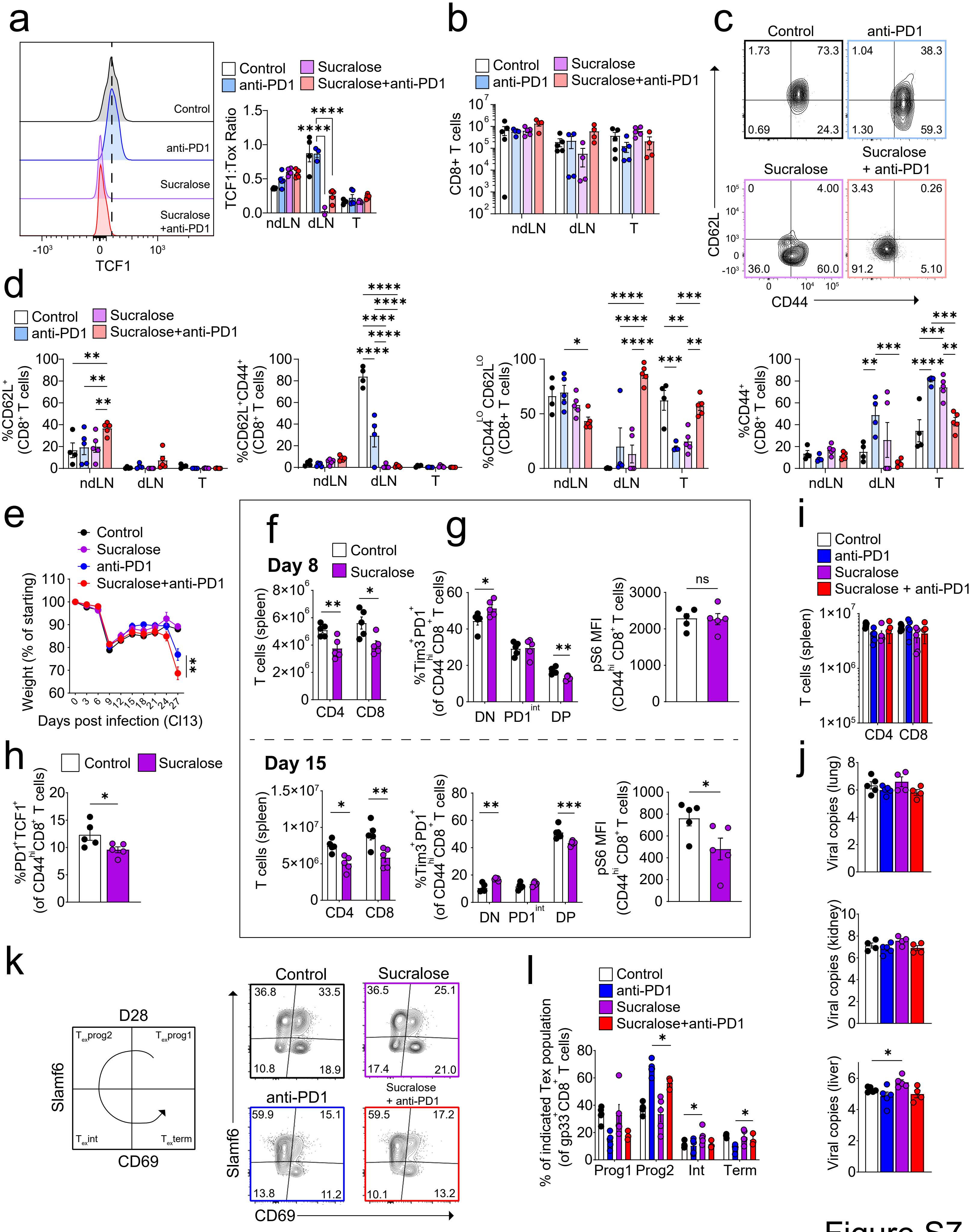
a
Control
anti-PD1
Sucralose
Sucralose +anti-PD1
TCF1
TCF1:Tox Ratio
ndLN dLN T
b
CD8+ T cells
c
CD62L
CD44
d
%CD62L+ (CD8+ T cells)
%CD62L+CD44+ (CD8+ T cells)
%CD44LO CD62LLO (CD8+ T cells)
%CD44+ (CD8+ T cells)
e
Weight (% of starting)
Days post infection (Cl13)
f
Day 8
Day 15
T cells (spleen)
CD4 CD8
g
%Tim3+PD1+ (of CD44hiCD8+ T cells)
DN PD1int DP
pS6 MFI (CD44hi CD8+ T cells)
ns
h
%PD1+TCF1+ (of CD44hiCD8+ T cells)
i
j
Viral copies (lung)
Viral copies (kidney)
Viral copies (liver)
k
D28
Tex prog2
Tex prog1
Tex int
Tex term
Slamf6
CD69
l
% of indicated Tex population (of gp33+CD8+ T cells)
Prog1 Prog2 Int Term
Figure S7

Supplementary Figure S7. C57Bl/6 mice from Taconic consumed sucralose in the drinking water (0.09mg/mL) for 2 weeks prior to tumor injection and for the duration of the experiment. Mice were injected with $2.5x10^5$ MC38 cells subcutaneously and treated with 200µg anti-PD1 on days 9 and 12. Lymphocytes were isolated from ndLN, dLN, and tumor (T) 14 days post tumor injection. Lymphocytes were isolated from tissues indicated and stained. **a,** Representative flow plot and quantification for TCF1/Tox. **b,** Total $CD8^+$ T cells in tissue indicated. **c-d,** $CD62L^+$ and $CD44^+$ $CD8^+$ T cells in tissues indicated. **e-l,** C57Bl/6 mice were infected with LCMV Cl13 and given sucralose-supplemented (0.09mg/mL) drinking water or regular drinking water. For long-term experiments, mice were treated with 200µg anti-PD1 on days 21, 24, and 27 post infection. **e,** Weight curve of mice exposed to LCMV Cl13 with treatments indicated. **f**, Total T cells present in spleen at D8 and D15 post infection. **g,** T cell exhaustion status as visualized by $\%Tim3^+PD1^+$ and pS6 MFI on D8 and D15 post infection. **h,** $\%PD1^+TCF1^+$ T cells D15 post infection. **i,** Total T cells in the spleen at D28 post infection. **j,** Total viral copies in the lung, kidney, and liver at D28 post infection. **k,** Representative flow plots of CD69 and Slamf6 expression in LCMV-specific ($gp33^+$) $CD8^+$ T cells in the spleen D28 post infection. **l,** Quantification of (**k**). Data are representative of 3 (**a-d**) or 2 (**e-l**) independent experiments with 4-5 mice per group per experiment. Error bars represent the mean ± SEM. two-way ANOVA (**e**) or student's T test (**a-d**, **f-l**) were used. *p<0.05, **p<0.005, ***p<0.0005, ****p<0.00005.
