# Supplementary material for: Sucralose Consumption Ablates Cancer Immunotherapy Response through Microbiome Disruption
Source: Cancer Discov. 2025 Jul 30;15(11):2278–97. doi: 10.1158/2159-8290.CD-25-0247 (PMC12580791; doi:10.1158/2159-8290.CD-25-0247)
Supplement: Supplementary Fig S8 — shows T cell proliferation, function, and metabolism following culture in sucralose. [file cd-25-0247_supplementary_fig_s8_suppsf8.pdf]

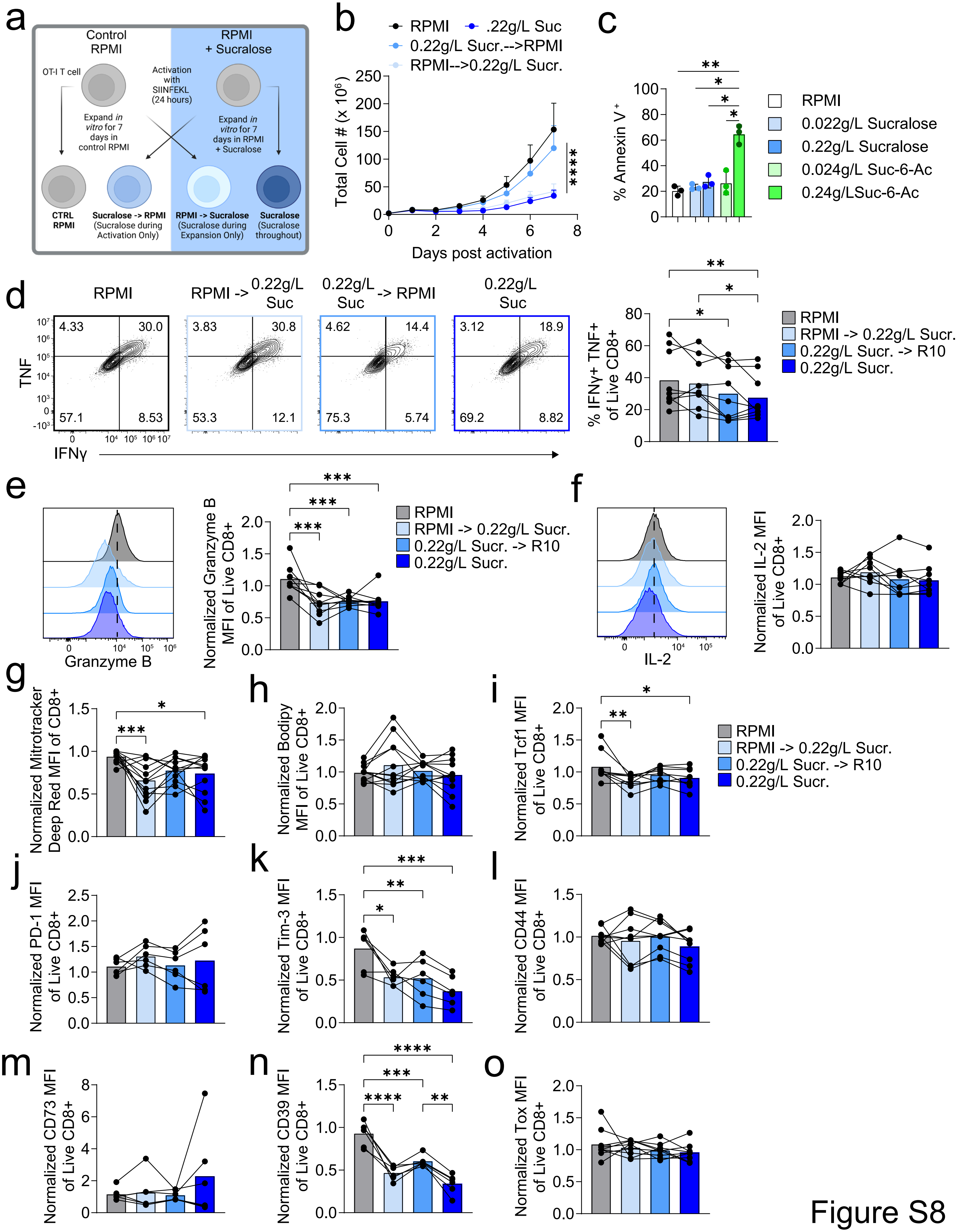
a
Control RPMI
RPMI + Sucralose
OT-I T cell
Activation with SIINFEKL (24 hours)
Expand in vitro for 7 days in control RPMI
Expand in vitro for 7 days in RPMI + Sucralose
CTRL RPMI
Sucralose -> RPMI (Sucralose during Activation Only)
RPMI -> Sucralose (Sucralose during Expansion Only)
Sucralose (Sucralose throughout)
b
RPMI
.22g/L Suc
0.22g/L Sucr.-->RPMI
RPMI-->0.22g/L Sucr.
Total Cell # (x 10^6)
Days post activation
****
c
% Annexin V+
RPMI
0.022g/L Sucralose
0.22g/L Sucralose
0.024g/L Suc-6-Ac
0.24g/LSuc-6-Ac
d
RPMI
RPMI -> 0.22g/L Suc
0.22g/L Suc -> RPMI
0.22g/L Suc
TNF
IFNγ
4.33 30.0 57.1 8.53
3.83 30.8 53.3 12.1
4.62 14.4 75.3 5.74
3.12 18.9 69.2 8.82
% IFNγ+ TNF+ of Live CD8+
RPMI
RPMI -> 0.22g/L Sucr.
0.22g/L Sucr. -> R10
0.22g/L Sucr.
e
Granzyme B
Normalized Granzyme B MFI of Live CD8+
f
IL-2
Normalized IL-2 MFI of Live CD8+
g
Normalized Mitrotracker Deep Red MFI of CD8+
h
Normalized Bodipy MFI of Live CD8+
i
Normalized Tcf1 MFI of Live CD8+
j
Normalized PD-1 MFI of Live CD8+
k
Normalized Tim-3 MFI of Live CD8+
l
Normalized CD44 MFI of Live CD8+
m
Normalized CD73 MFI of Live CD8+
n
Normalized CD39 MFI of Live CD8+
o
Normalized Tox MFI of Live CD8+

Figure S8

Supplementary Figure S8. a, OT-I T cells were activated with SIINFEKL peptide for 24 hours in either control RPMI (left) or RPMI supplemented with 0.22g/L sucralose (right). After 24 hours of activation, cells from each activation condition were expanded *in vitro* for 6 more days in either control RPMI or RPMI supplemented with sucralose. **b,** Total cell number was monitored daily for each condition. **c,** CD8+ T cells were activated in control RPMI as in **a**), then cells were transferred into RPMI media containing sucralose or sucralose-6-acetate at indicated concentrations. 20 hours after culture, annexin V staining of $CD8^+$ T cells was measured by flow cytometry **d-f,** Cells were cultured as in **a**) and then were restimulated with 3µg/mL plate-bound αCD3 and 1 µg/mL αCD28 for 5.5 hours in control RPMI supplemented with Protein Transport Inhibitor and analyzed for **d,** IFNγTNFα+ coexpression. **e,** Granzyme B expression and **f,** IL-2 expression via flow cytometry. **g-o,** Cells were cultured as described in **a**), then labeled with **g**) Mitotracker Deep Red **h**) Bodipy metabolic dyes and antibodies specific for **i**) Tcf1 **j**) PD-1 **k**) Tim-3 **l**) CD44 **m**) CD73 **n**) CD39 **o**) Tox. Data are representative of 4 (**b,g-h**), 3 (**d-e, i, l, o**) or 2 (**j-k, m-n**) independent experiments with 3 mice per group per experiment. Error bars represent the mean ± SEM. two-way ANOVA (**b**), or one-way ANOVA with Tukey's multiple comparisons test (**c-o**) were used. *p<0.05, **p<0.005, ***p<0.0005, ****p<0.00005. Connecting lines between samples on bar plots indicate paired samples (i.e. each mouse was split into each of the 4 treatment groups)
