# Supplementary material for: Sucralose Consumption Ablates Cancer Immunotherapy Response through Microbiome Disruption
Source: Cancer Discov. 2025 Jul 30;15(11):2278–97. doi: 10.1158/2159-8290.CD-25-0247 (PMC12580791; doi:10.1158/2159-8290.CD-25-0247)
Supplement: Supplementary Fig S9 — shows T cell proliferation and metabolism following sucralose culture during activation, expansion, or the entirety of culture. [file cd-25-0247_supplementary_fig_s9_suppsf9.pdf]

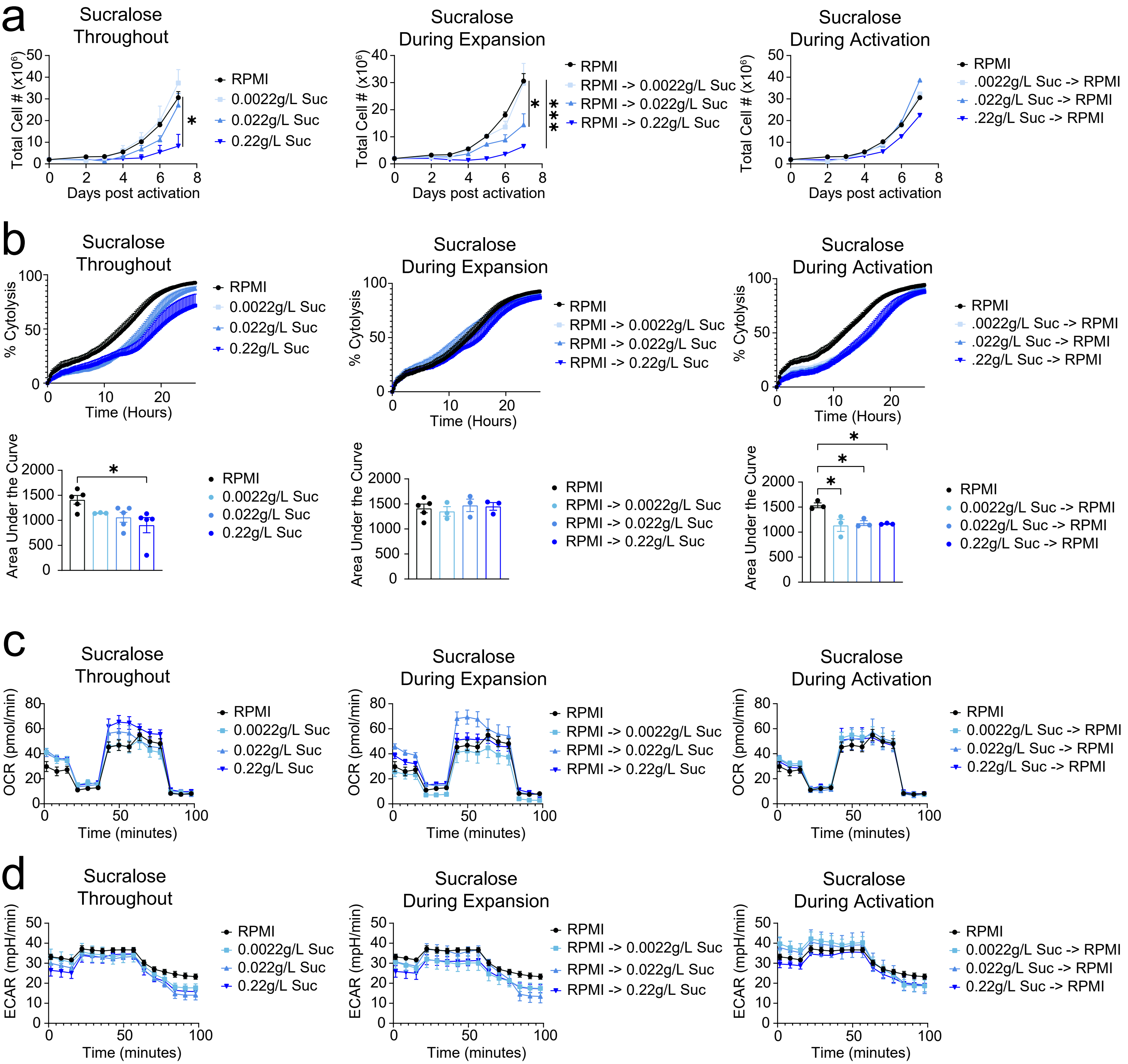
a
Sucralose Throughout
Total Cell # (x10⁶)
Days post activation
RPMI
0.0022g/L Suc
0.022g/L Suc
0.22g/L Suc
Sucralose During Expansion
RPMI -> 0.0022g/L Suc
RPMI -> 0.022g/L Suc
RPMI -> 0.22g/L Suc
Sucralose During Activation
.0022g/L Suc -> RPMI
.022g/L Suc -> RPMI
.22g/L Suc -> RPMI
b
% Cytolysis
Time (Hours)
Area Under the Curve
0.0022g/L Suc -> RPMI
0.022g/L Suc -> RPMI
0.22g/L Suc -> RPMI
c
OCR (pmol/min)
Time (minutes)
d
ECAR (mpH/min)

Figure S9

Supplementary Figure S9. For all panels, OT-I T cells were activated with SIINFEKL peptide for 24 hours in either control RPMI or RPMI supplemented with sucralose at varying concentrations. After 24 hours of activation, cells from each activation condition were expanded *in vitro* for 6 more days in either control RPMI or RPMI supplemented with sucralose. **a,** Total cell number was monitored for each condition. **b,** Day 7 cells were cultured at a 1:1 ratio with B16-OVA tumor cells, and cytolysis was monitored using an RTCA eSight. Data are shown as % Cytolysis vs. time (top) and area under the curve is quantified below (bottom). **c-d,** Day 7 cells from each group placed in a Seahorse Mito Stress Test and monitored for **c**) Oxygen Consumption Rate (OCR) and **d**) extracellular acidification rate (ECAR). Data are representative of 1 (**a**) or 3 (**b-d**) independent experiments with 3-5 mice per group across all experiments. Error bars represent the mean ± SEM. two-way ANOVA (**a**) or One-way ANOVA with Tukey's multiple comparisons test (**b**) were used to determine statistical significance. *$p<0.05$, ***$p<0.0005$.
