# Supplementary material for: Sucralose Consumption Ablates Cancer Immunotherapy Response through Microbiome Disruption
Source: Cancer Discov. 2025 Jul 30;15(11):2278–97. doi: 10.1158/2159-8290.CD-25-0247 (PMC12580791; doi:10.1158/2159-8290.CD-25-0247)
Supplement: Supplementary Fig S10 — shows a western blot assessing TCR signaling of T cells cultured in sucralose. [file cd-25-0247_supplementary_fig_s10_suppsf10.pdf]

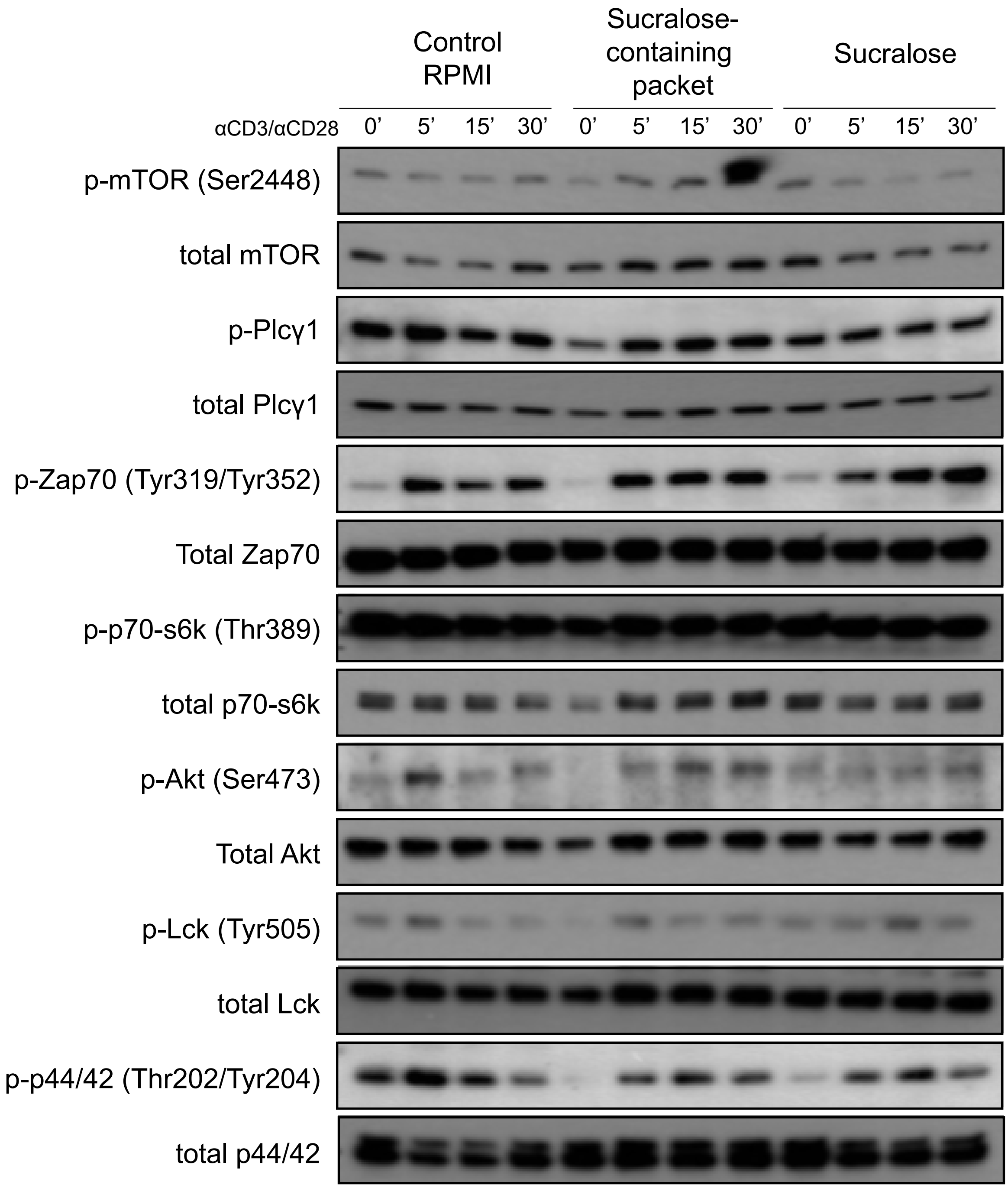
Control RPMI
Sucralose-containing packet
Sucralose
αCD3/αCD28 0' 5' 15' 30' 0' 5' 15' 30' 0' 5' 15' 30'
p-mTOR (Ser2448)
total mTOR
p-Plcγ1
total Plcγ1
p-Zap70 (Tyr319/Tyr352)
Total Zap70
p-p70-s6k (Thr389)
total p70-s6k
p-Akt (Ser473)
Total Akt
p-Lck (Tyr505)
total Lck
p-p44/42 (Thr202/Tyr204)
total p44/42

Figure S10

Supplementary Figure S10. Western blots showing TCR signaling of CD8+ T cells activated with CD3 crosslinking in vitro. Magnetically enriched CD8+ T cells were isolated from spleens of five different B6 mice by negative selection. Cells were pooled and split into three groups in different cell culture media: 1) control Serum-Free RPMI (NC, left), or 2) Serum-Free RPMI supplemented with 2% sucralose containing packet (middle) or 3) 0.22g/L sucralose (right) and stimulated with 3μg/mL biotin-labeled αCD3 antibody, 4μg/mL of αCD28 antibody, and + 1.5μg/mL streptavidin for the indicated times. Blots are shown for each antibody specific for each of the following: p-mTOR (Ser2448) and total mTOR, p-PLCγ (Ser1248) and total PLC-γ, p-Zap70 (Tyr 319/Tyr352) and total Zap70, p-p70-s6k (Thr389) and total p70-s6k, p-Akt (Ser473) and total Akt, pLck (Tyr505) and total Lck, and p-p44/42 (Thr202/Tyr204) and total p44/42.
