# Supplementary material for: Sucralose Consumption Ablates Cancer Immunotherapy Response through Microbiome Disruption
Source: Cancer Discov. 2025 Jul 30;15(11):2278–97. doi: 10.1158/2159-8290.CD-25-0247 (PMC12580791; doi:10.1158/2159-8290.CD-25-0247)
Supplement: Supplementary Fig S11 — shows a volcano plot and pathway enrichment of T cells cultured in sucralose during activation and expansion. [file cd-25-0247_supplementary_fig_s11_suppsf11.pdf]

a

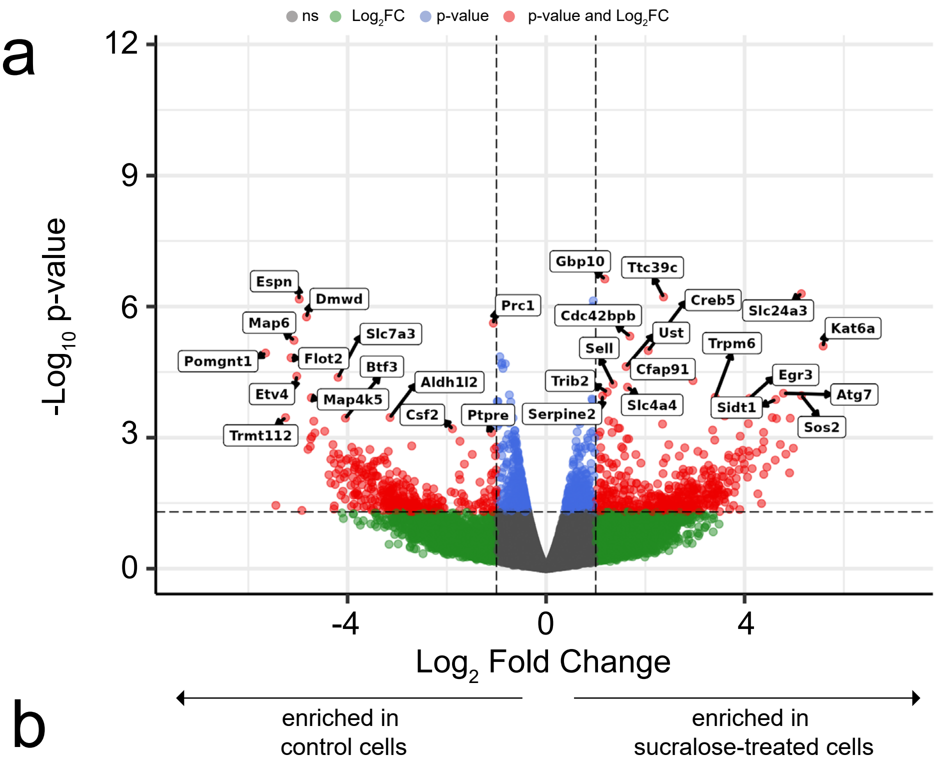

b

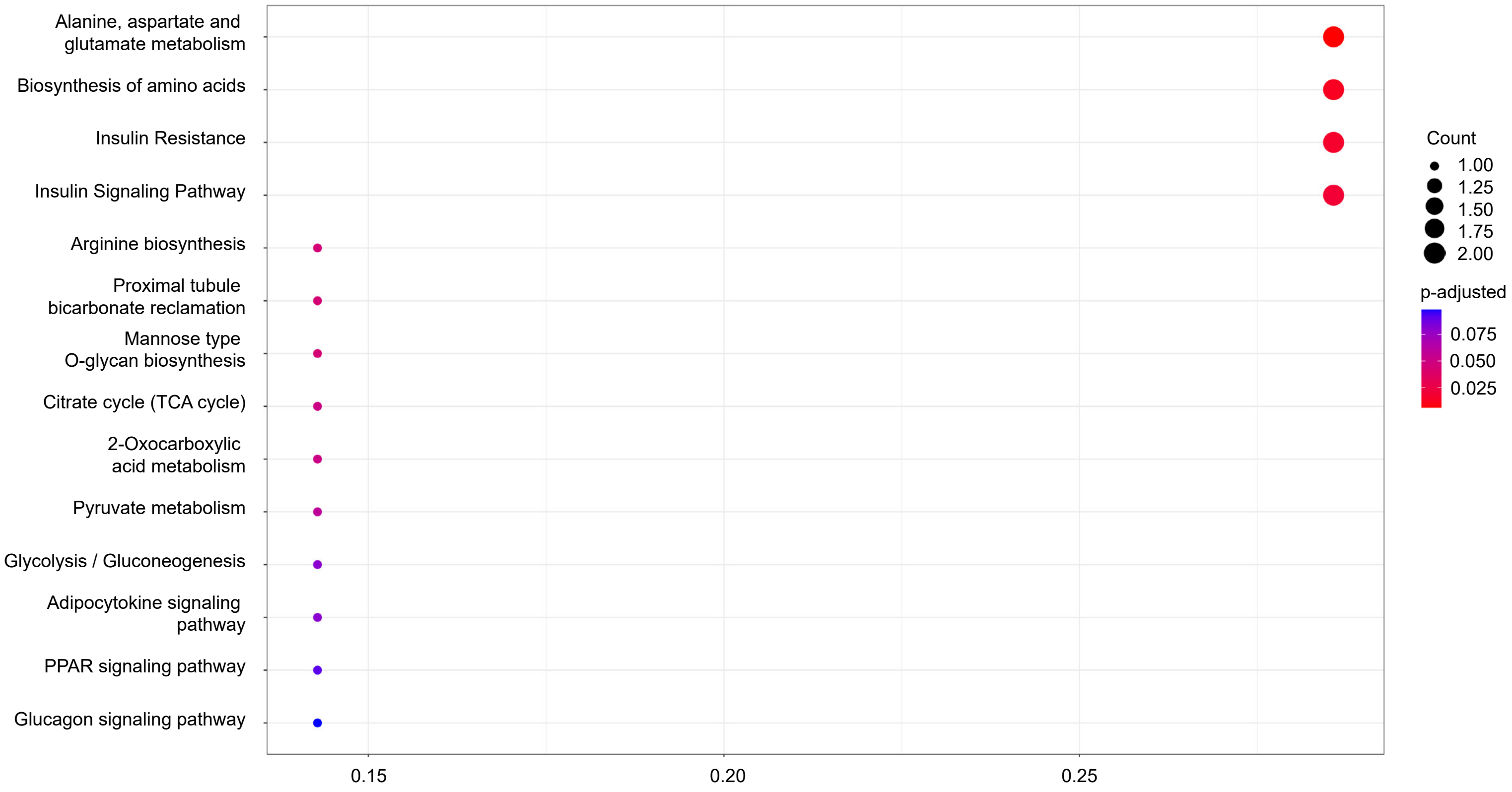

Figure S11

Supplementary Figure S11. OT-I T cells were activated and cultured for 7 days as described in Supplementary Figure 8a. Cells were cultured in control RPMI the entire time or in RPMI supplemented with 0.22g/L sucralose for the duration of the experiment. After 7 days in culture, RNA was isolated from $CD8^+$ T cells, and RNA-Sequencing was performed. **a,** volcano plot of all differentially expressed genes between control and sucralose exposed $CD8^+$ T cells. **b,** KEGG pathway analysis of the top enriched pathways between control RPMI treated and sucralose treated cells.
