# Supplementary material for: Sucralose Consumption Ablates Cancer Immunotherapy Response through Microbiome Disruption
Source: Cancer Discov. 2025 Jul 30;15(11):2278–97. doi: 10.1158/2159-8290.CD-25-0247 (PMC12580791; doi:10.1158/2159-8290.CD-25-0247)
Supplement: Supplementary Fig S12 — shows principle coordinate analysis and beta diversity of stool samples from mice consuming sucralose or regular drinking water. [file cd-25-0247_supplementary_fig_s12_suppsf12.pdf]

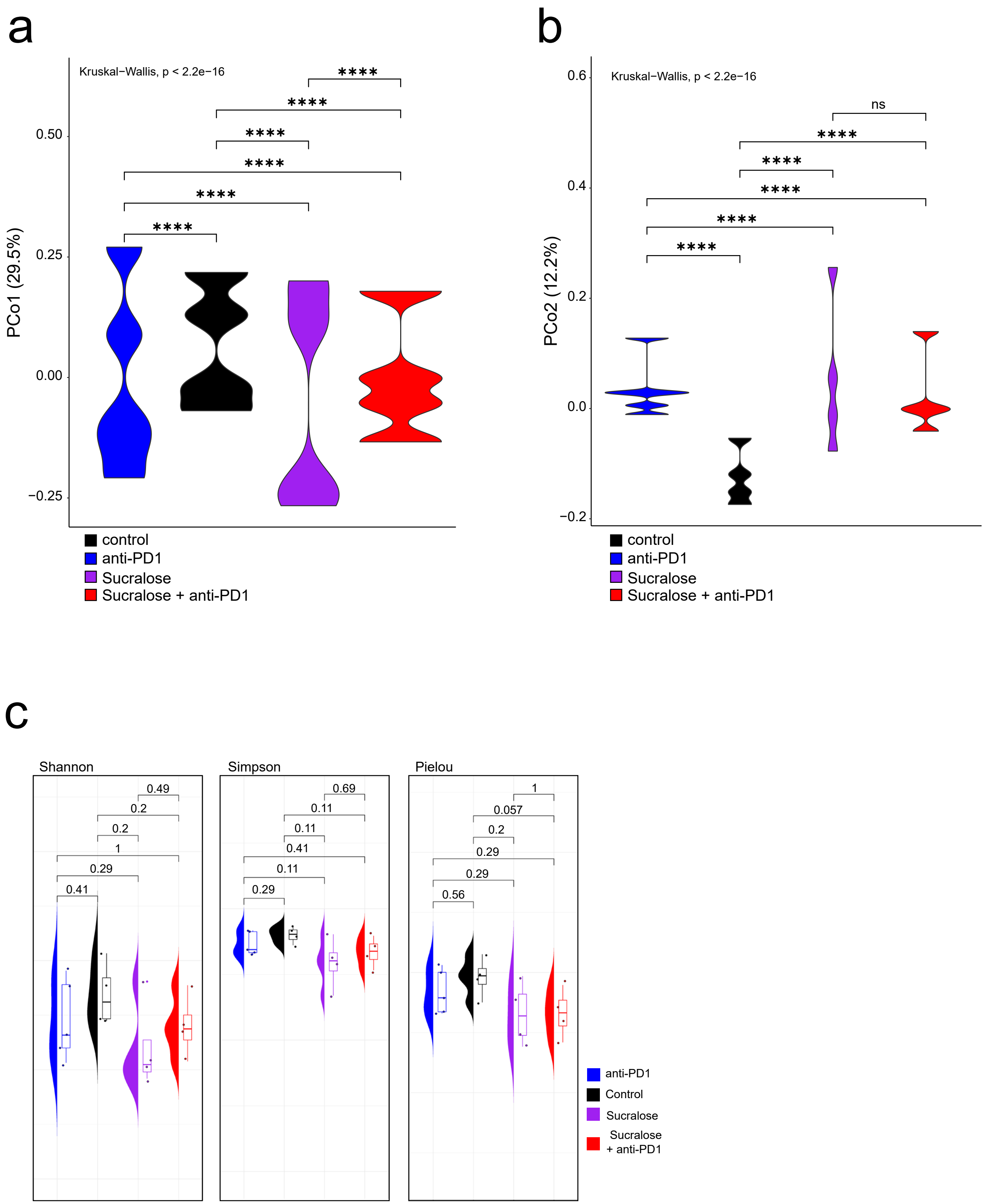

a
Kruskal−Wallis, p < 2.2e−16
PCo1 (29.5%)
0.50
0.25
0.00
−0.25
****
control
anti-PD1
Sucralose
Sucralose + anti-PD1
b
Kruskal−Wallis, p < 2.2e−16
PCo2 (12.2%)
0.6
0.4
0.2
0.0
−0.2
ns
****
control
anti-PD1
Sucralose
Sucralose + anti-PD1
c
Shannon
0.49
0.2
0.2
1
0.29
0.41
Simpson
0.69
0.11
0.11
0.41
0.11
0.29
Pielou
1
0.057
0.2
0.29
0.29
0.56
anti-PD1
Control
Sucralose
Sucralose + anti-PD1

Figure S12

Supplementary Figure S12. Sucralose consumption shifts gut microbiome diversity and function. Microbiome diversity at timepoint day 38. **a-b,** Principal coordinate 1 (PCo1) and principal coordinate 2 (PCo2) comparisons between groups from principal coordinate analysis of beta diversity. The Wilcoxon test was used for group comparisons where **** represents p <= 0.0001. The Kruskal-Wallis test was used for a global comparison between groups. **c,** Alpha diversity between groups where the diversity index is specified by the header on each plot. Comparisons between groups were done with the Wilcoxon test. Data are representative of 1 independent experiment with 5 mice per group.
