# Supplementary material for: Sucralose Consumption Ablates Cancer Immunotherapy Response through Microbiome Disruption
Source: Cancer Discov. 2025 Jul 30;15(11):2278–97. doi: 10.1158/2159-8290.CD-25-0247 (PMC12580791; doi:10.1158/2159-8290.CD-25-0247)
Supplement: Supplementary Fig S13 — shows beta diversity and heatmaps of stool from mice consuming sucralose. It also shows metabolomics readouts of stool of these mice as well as tumor growth curves of mice consuming sucralose with or without arginine or citrulline supplementation. [file cd-25-0247_supplementary_fig_s13_suppsf13.pdf]

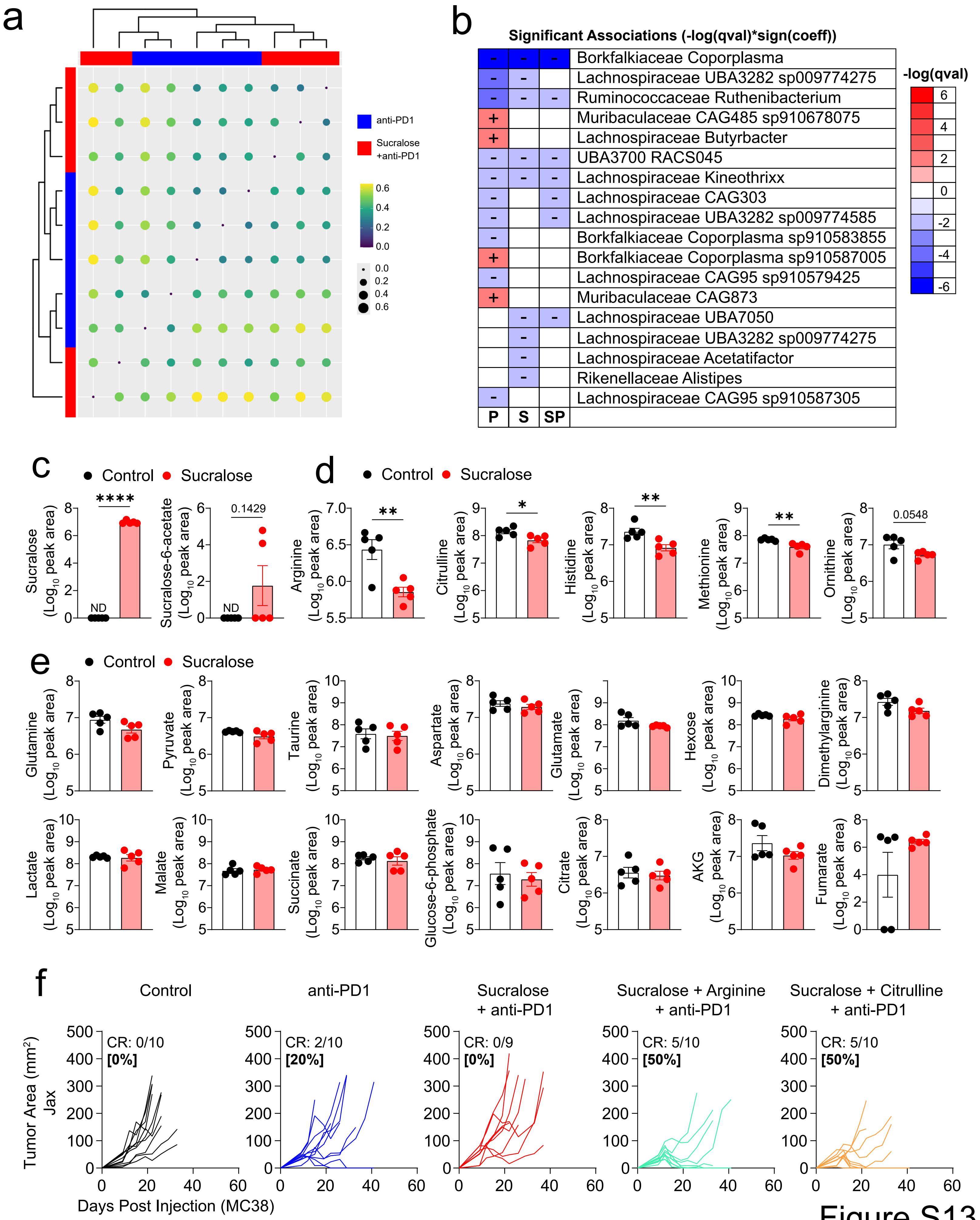
a
anti-PD1
Sucralose +anti-PD1
b
Significant Associations (-log(qval)*sign(coeff))
Borkfalkiaceae Coporplasma
Lachnospiraceae UBA3282 sp009774275
Ruminococcaceae Ruthenibacterium
Muribaculaceae CAG485 sp910678075
Lachnospiraceae Butyrbacter
UBA3700 RACS045
Lachnospiraceae Kineothrixx
Lachnospiraceae CAG303
Lachnospiraceae UBA3282 sp009774585
Borkfalkiaceae Coporplasma sp910583855
Borkfalkiaceae Coporplasma sp910587005
Lachnospiraceae CAG95 sp910579425
Muribaculaceae CAG873
Lachnospiraceae UBA7050
Lachnospiraceae UBA3282 sp009774275
Lachnospiraceae Acetatifactor
Rikenellaceae Alistipes
Lachnospiraceae CAG95 sp910587305
P S SP
-log(qval)
c
Control Sucralose
Sucralose (Log10 peak area)
Sucralose-6-acetate (Log10 peak area)
ND
0.1429
d
Control Sucralose
Arginine (Log10 peak area)
Citrulline (Log10 peak area)
Histidine (Log10 peak area)
Methionine (Log10 peak area)
Ornithine (Log10 peak area)
0.0548
e
Control Sucralose
Glutamine (Log10 peak area)
Pyruvate (Log10 peak area)
Taurine (Log10 peak area)
Aspartate (Log10 peak area)
Glutamate (Log10 peak area)
Hexose (Log10 peak area)
Dimethylarginine (Log10 peak area)
Lactate (Log10 peak area)
Malate (Log10 peak area)
Succinate (Log10 peak area)
Glucose-6-phosphate (Log10 peak area)
Citrate (Log10 peak area)
AKG (Log10 peak area)
Fumarate (Log10 peak area)
f
Control
anti-PD1
Sucralose + anti-PD1
Sucralose + Arginine + anti-PD1
Sucralose + Citrulline + anti-PD1
CR: 0/10 [0%]
CR: 2/10 [20%]
CR: 0/9 [0%]
CR: 5/10 [50%]
CR: 5/10 [50%]
Jax
Tumor Area (mm²)
Days Post Injection (MC38)

Figure S13

Supplementary Figure S13. Tac mice consumed sucralose in the drinking water for 14 days prior to tumor injection and for the duration of the experiment. Mice were injected with 2.5×10^5 MC38 cells subcutaneously and treated with anti-PD1 on days 9, 12, and 15. **a,** Bray-Curtis bubble plot comparing the microbiome beta diversity between samples of anti-PD1 (blue) and sucralose + anti-PD1 (red) mice at day 38 post sucralose. **b,** Heatmap of differentially expressed taxa identified with MaAsLin2 between groups on Day 38 post sucralose. P: anti-PD1, S: Sucralose, SP: Sucralose + anti-PD1. **c,** Quantification of sucralose and sucralose-6-acetate within the stool of mice indicated. **d-e,** Quantification of various metabolites within the stool of mice indicated. **f,** Jackson mice consumed sucralose or control drinking water in the presence of absence of arginine or citrulline for 2 weeks prior to tumor injection and throughout the duration of the experiment. Mice were injected with 2.5×10^5 MC38 cells subcutaneously and treated with 200µg anti-PD1 at days 9, 12, and 15. Individual tumor growth curves of mice from Jackson Labs. Data are a composite of 3 (**f**) or 1 (**a-e**) independent experiments with 5 mice per group per experiment. Error bars represent the mean ± SEM. Students t-test was used (**c-e**). *$p<0.05$, **$p<0.005$, ****$p<0.00005$.
