# Supplementary material for: Sucralose Consumption Ablates Cancer Immunotherapy Response through Microbiome Disruption
Source: Cancer Discov. 2025 Jul 30;15(11):2278–97. doi: 10.1158/2159-8290.CD-25-0247 (PMC12580791; doi:10.1158/2159-8290.CD-25-0247)
Supplement: Appendix 1 — shows the IRB approval for HCC 20-019. [file cd-25-0247_appendix_1_suppsa1.pdf]

Appendix 1. IRB approval for HCC 20-019 protocol.

University of Pittsburgh | Institutional Review Board
Office of Research Protections

MODIFICATION APPROVAL (Expedited)

Date:	April 25, 2024
IRB:	MOD20010266-016
PI:	Diwakar Davar, MD
Title:	Comprehensive Intestinal Microbiome and Dietary History Evaluation of Patients with Advanced Cancers on Treatment with Immune Checkpoint Blockade

The Institutional Review Board reviewed and approved the above referenced modification, and the study may continue as outlined in the University of Pittsburgh approved application and documents.

Approval Documentation

Review type:	Modification / Update
Approval Date:	4/25/2024
Expiration Date:	
Expedited Category:	(mm) Minor modification
Approved Documents:	• 2024-04-24 Protocol Version 4.0 Final_20-019.docx, Category: IRB Protocol;<br>• 2024-04-24 Protocol Version 4.0 TC_20-019.docx, Category: IRB Protocol;<br>• 2024-04-24 UPMC ICF_20-019.pdf, Category: Consent Form

As the Principal Investigator, you are responsible for the conduct of the research and to ensure accurate documentation, protocol compliance, reporting of possibly study-related adverse events and unanticipated problems involving risk to participants or others. The HRP Reportable Events policy, Chapter 17, is available at http://www.hrpo.pitt.edu/.

If you have any questions, please contact the University of Pittsburgh IRB Coordinator, Juliet Mancino.

Please take a moment to complete our Satisfaction Survey as we appreciate your feedback.

The University of Pittsburgh has a Federal Wide Assurance approved through the Office of Human Research Protections (FWA00006790).
