# Supplementary material for: Sucralose Consumption Ablates Cancer Immunotherapy Response through Microbiome Disruption
Source: Cancer Discov. 2025 Jul 30;15(11):2278–97. doi: 10.1158/2159-8290.CD-25-0247 (PMC12580791; doi:10.1158/2159-8290.CD-25-0247)
Supplement: Appendix 2 — shows the full protocol for HCC 20-019. [file cd-25-0247_appendix_2_suppsa2.pdf]

Appendix 2. Full protocol for HCC 20-019.

Comprehensive Intestinal Microbiome and Dietary History Evaluation of Patients with Advanced Cancers on Treatment with Immune Checkpoint Blockade

Principal Investigators: Diwakar Davar, MD

Co-Investigators: all UPCI MDs

Version: 4.0 (4/24/2024)

1. **Introduction and Background:**

PD-1 and CTLA-4 Immune Checkpoint Inhibitors (ICI) in Cancer

PD-1 is a receptor expressed by activated T cells which binds to PD-L1 (B7-H1)[1,2] and PD-L2 (B7-DC)[3,4]. PD-1 negatively regulates T cell functions through the engagement of PD-L1, which is expressed by a wide variety of tissues[1-4]. PD-L1 is also expressed by human tumors, including melanoma, either constitutively or after treatment with IFN-γ[5,6]. Cytotoxic T lymphocyte associated antigen-4 (CTLA-4, CD152) is an activation-induced glycoprotein that belongs to the Immunoglobulin (Ig) superfamily. CTLA-4 is homologous to the T cell co-stimulatory protein CD28; but where CD28 provides the co-stimulatory signal required for antigen-specific T cell activation and expansion after the initial interaction between T cell receptor (TCR) and antigen presenting cells (APCs), CTLA-4 down-regulates T cell responses by acting as a decoy receptor[7-9]. CTLA-4 is constitutively expressed on regulatory T cells (Tregs) while expression on CD8+ T cells occurs rapidly following TCR engagement (signal 1)[10,11]. Both CD28 and CTLA-4 have two natural ligands found on APCs: CD80 (B7.1) or CD86 (B7.2)[12-14]; although CTLA-4 has higher avidity and affinity for both compared to CD28[15-17]. Because B7.1/B7.2 provide the positive costimulatory signal (signal 2) through CD28 required for TCR activation; competitive inhibition of CD80 (B7.1) and CD86 (B7.2) by CTLA-4 effectively attenuates T cell activation. B7.1/B7.2 ligands are primarily expressed at sites of T-cell priming (e.g. secondary lymphoid organs), and to a lesser extent constitutively expressed to varying degrees on antigen-presenting cells (APC) and activated T cells. Hence, CTLA-4 blockade *primarily* increases T-cell priming; and *secondarily* maintains tolerogenic state of T cells upon antigenic stimulation.

Ex vivo, blockade of the PD-1/programmed death ligand 1 (PD-L1) pathway in combination with prolonged antigen stimulation with PD-L1+ antigen-presenting cells or melanoma cells augments the frequencies of cytokine-producing, proliferating and total tumor antigen-specific NY-ESO-1 CD8+ T cells[18]. In human cancer, CTLA-4 blockade induces expansion of ICOS+ T_h1-like CD4 effector population and exhausted CD8 T cells[19]; where the combination of CTLA-4/PD-1 blockade induces distinct changes[20].

Blockade of PD-1 singly or in combination with CTLA-4, is associated with improved response and survival rates in the setting of advanced cancer in multiple cutaneous malignancies including melanoma[21-27], non-small cell lung cancer (NSCLC)[28-31], and renal cell carcinoma (RCC)[32-36]. Multiple biomarkers of response to PD-1/PD-L1 blockade have been described including CD8+ TIL-infiltrate[37], PD-L1 expression[38,39], tumor mutation burden (TMB)[40,41], and changes in circulating exhausted-phenotype CD8 T cells[40-43]. While combination PD-1/CTLA-4 blockade is associated with greater overall response rates (ORR) compared to single-agent PD-1 blockade, this is associated with greater incidence of severe side effects, particularly immune-related adverse events (irAE). These are summarized in **Table 1**.

	**PD-1 Blockade**			**PD-1/CTLA-4 Combination Blockade**		
	**ORR**	**Incidence of Grade 3-4 irAE**	**Treatment-Discontinuation Rate**	**ORR**	**Incidence of Grade 3-4 irAE**	**Treatment-Discontinuation Rate**
**Melanoma (adjuvant)**	N/A	15-18%[44,45]	10-14%[44,45]	N/A	N/A	N/A
**Melanoma**	35%-41%[21,23]	18%[46]	9%[46]	58%[27]	55%[27]	38%[27]
**NSCLC**	45% (PD-L1 ≥50%)[28]	10%[28]	7%[28]	36% (PD-L1 ≥1%)[30]	33%[30]	18%[30]
**RCC**	34%[34]	18%[34]	18%[34]	42%[32,36]	35%[32,36]	22%[32,36]

Role of Intestinal Microbiota in Mediating Response and irAE to Anti-PD-1 and Anti-CTLA-4 ICI

In addition to tumor-intrinsic mechanisms supporting resistance to anti-PD-1, the gut microbiome is a major tumor-extrinsic regulator of responses to anti-PD-1[47-50] and anti-CTLA-4[51,52]. In mice, composition of the gut microbiome modulates therapeutic activity of anti-PD-1/programmed death-ligand 1 (PD-L1), and administration of certain gut commensals or responder-derived fecal microbiota transplantation (R-FMT) promotes anti-PD-1 efficacy in melanoma-bearing mice[53-55]. While multiple studies have reported that a favorable gut microbiome is associated with response to anti-PD-1 in cancer

patients, its precise composition is not yet fully understood[53-55], and the concordance among identified species in different studies is **limited**[53-57].

To evalaute the role of microbiome modulation in mediating PD-1 primary resistance, we previously conducted a single-arm clinical trial to evaluate the safety and efficacy of FMT obtained from individual long-term responder (R) melanoma patients together with anti-PD-1 in PD-1 primary refractory metastatic melanoma patients[58]. In this study, we demonstrated that a single administration of responder-derived FMT (R-FMT) resensitized patients with PD-1 primary refractory melanoma to pembrolizumab. We observed objective responses in 3 patients and durable stable disease (SD) lasting >12 months in 3 patients (i.e. ORR 20%, DCR 40%) (**Fig. 1A, B**).

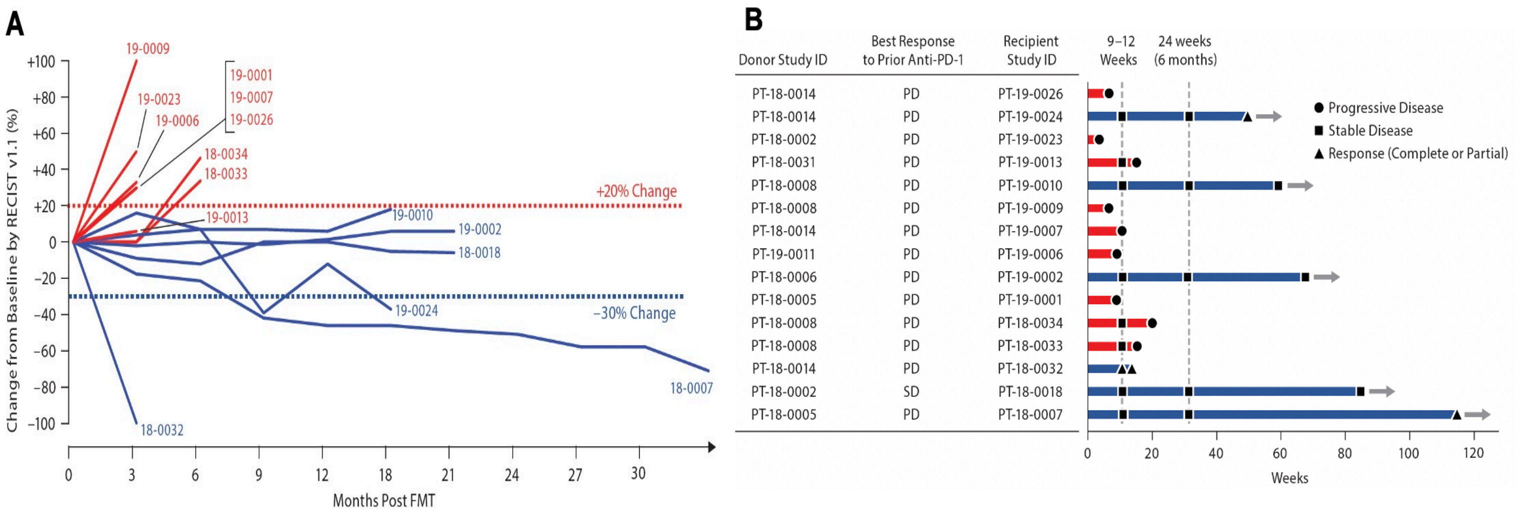

Figure 1. Radiographic Responses by RECIST v1.1 from a Phase II Study of Anti-PD-1 Responder-Derived FMT and Pembrolizumab in PD-1-Refractory Melanoma. A) Treatment exposure and B) Response duration by RECIST v1.1 (investigator assessed; n = 15). (A) FMT donor and best response to prior line(s) of anti-PD(L)1 therapy singly or in combination are shown for each FMT recipient patient. Length of each bar corresponds to duration of time patients received treatment (in weeks). Response status is color coded (R – blue; NR – red). Response symbols represent status at first restaging scan (9–12 weeks) and at most recent review. Patients with ongoing response on study are depicted with horizontal arrows. (B) Radiographic change of tumor burden from baseline (investigator assessed per RECIST v1.1; n = 15). One patient (PT-18-0018) had initial disease stability with subsequent progression following antibiotic therapy and was offered a re-transplant with the same donor, with subsequent disease stabilization.

Metagenomic analyses revealed that the vast majority of **significantly enriched** taxa in responders belonged to the phyla Firmicutes (*Lachnospiraceae* and *Ruminococcaceae* families) and Actinobacteria (*Bifidobacteriaceae* and *Coriobacteriaceae* families), while the majority of bacteria **decreased** in responders belonged to phylum Bacteroidetes. Response was also associated with distinct immunophenotypic changes including increased CD56+CD8+ T cells, higher percentages of TEMRA cells and increased mucosal-associated invariant T (MAIT) cells (**Fig. 2A-E**). **Our findings showed that a single R-FMT successfully colonized responders, shifting microbiome composition towards taxa favoring anti-PD-1 efficacy and was associated with increased anti-tumor immunity peripherally and intra-tumorally.**

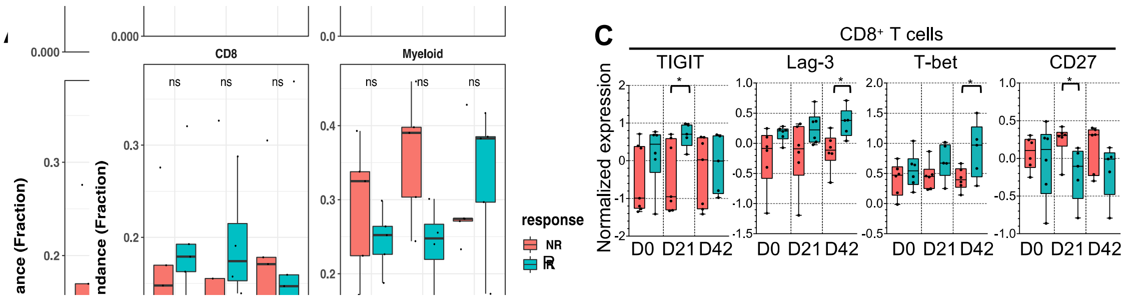

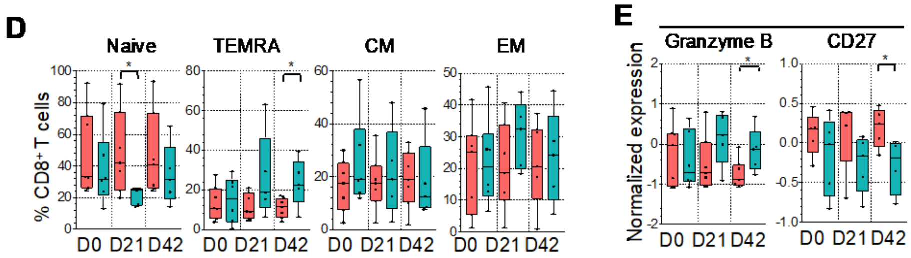

Figure 2. Unsupervised Multiparameter Flow Cytometry Analysis of Circulating Immune Cells (A-E) and High-Resolution Single-Cell Analysis of Intra-tumoral CD45+ Immune Cells (F-I). A) Unsupervised multiparameter flow cytometry analysis of circulating immune cells. UMAP visualization of 100,000 live single cells from Rs and NRs at three time points—pre-treatment (D0), day 21 (D21) and D42 — from 30-parameter flow panel analysis (n=14) after merging clusters based on expression of CD3, CD4, CD8, CD19, CD14, CD56, Tgd1, and Tgd2. Myeloid cells were identified as lineage-negative cell clusters based on presence or absence of CD14+ cells. **B) Frequency of CD56+CD8+ clustered T cells in PBMCs of patients.** Whisker boxes show frequencies of CD56+CD8+ clustered T cells in PBMCs between Rs and NRs at D0, D21, and D42. We observed a significant increase of CD56+CD8+ T cells in Rs at D42 using the unpaired t-test (*p<0.05). **C) and D)** Phenotypic analysis of circulating CD8+ T cells. Whisker boxes show markers that are significantly differentially expressed (normalized mean fluorescence intensity) in CD8+ T cells (C) and CCR7+CD45RA+ naïve, terminally differentiated effector memory CCR7-CD45RA+ (TEMRA), CCR7+CD45RA- effector memory (EM), and CCR7-CD45RA- central memory (CM) cells (D) between Rs (n=5–6) and NRs (n=5–7) at the three time points. Analysis was performed on live single CD3+ and TCRgd- T cells. In Rs, we observed upregulation of TIGIT, Lag-3, and T-bet post-treatment and downregulation of CD27 in CD8+ T cells using the unpaired t-test (*p<0.05). **E) Phenotypic analysis of circulating MAIT cells.** Whisker-boxes comparing MAIT cells between Rs (n=5–6) and NRs (n=5–7) at the three time points. Analysis was performed on live single CD3+ and TCRgd- T cells. In Rs, MAIT cells upregulated granzyme B expression and downregulated CD27 post-treatment using the unpaired t-test (*p<0.05).

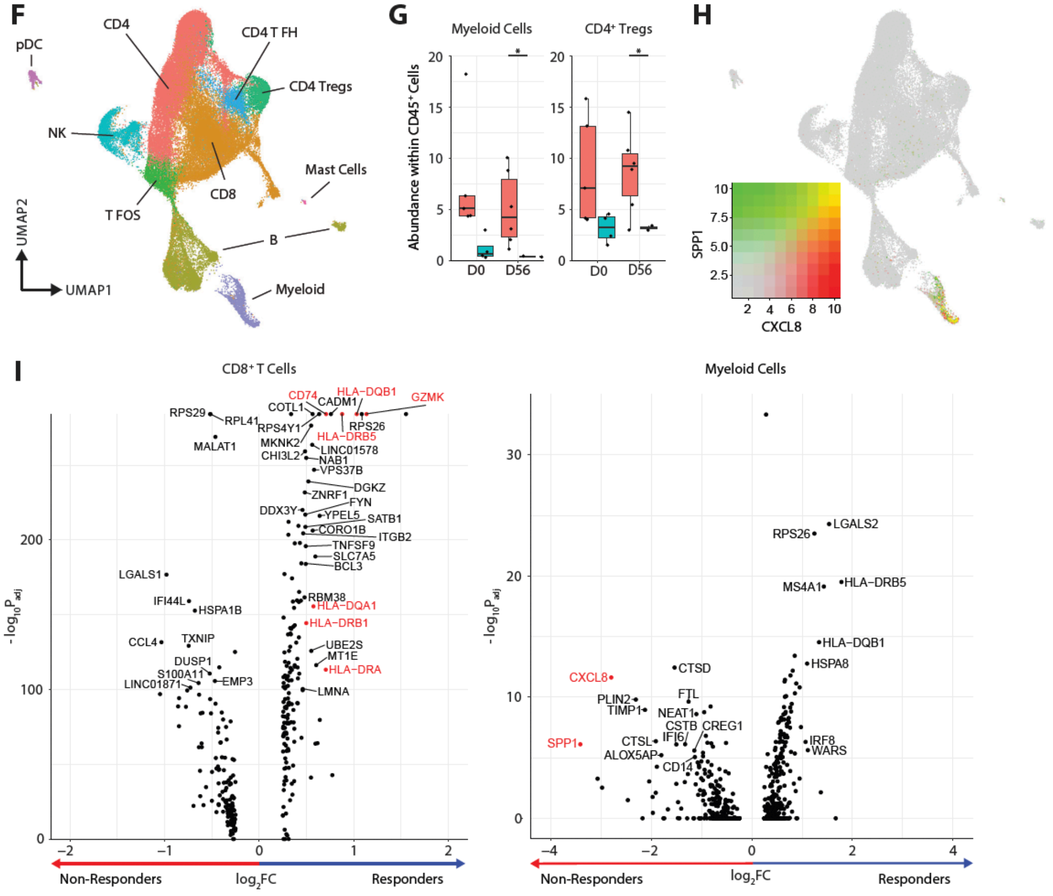

Figure 2 (cont.). F) scRNA-seq analysis of tumor-infiltrating immune cells. UMAP projection of 64,000 CD45+ cells that were clustered and manually identified based upon their expression profile. **G) Abundance of myeloid cells and CD4+ T regs in CD45+ tumor-infiltrating cells.** Whisker boxes showing the abundance of myeloid cells and CD4+ T regs in CD45+ tumor-infiltrating cells. We observed decreased abundance of myeloid cells and CD4+ T regs in Rs compared to NRs using the unpaired t-test (*p<0.05). **H) Cell-associated expression of two markers (CXCL8, SPP1) in UMAP projection.** These markers are predominantly expressed in suppressive myeloid cells. **I) Volcano plots showing the differences in phenotype of CD8+ T cells and myeloid cells between Rs and NRs post-FMT.** Rs show a CD8+ T phenotype with increased activation markers (GZMK, class II HLA genes, CD74), while NRs show a myeloid phenotype with an increased suppressive signature (CXCL8, SPP1) at day 56 post-treatment. Adjusted p-values were obtained by Wilcoxon rank-sum test.

observations across cohorts regarding the role of key commensal bacteria, we evaluated a ne[illegible] patient cohort, along with four previously published datasets using novel computational approaches (Davar D, et al; submitted). Specifically, we identified using time serial PERMANOVA, that taxonomic compositional abundances maximally separated progressors (Ps) and non-progressors (NPs) at 10 months (**Fig. 3A, B**); and thereby identified signatures of beneficial and detrimental taxa that contributed to response and resistance to anti-PD1 therapy (**Fig. 3C**).

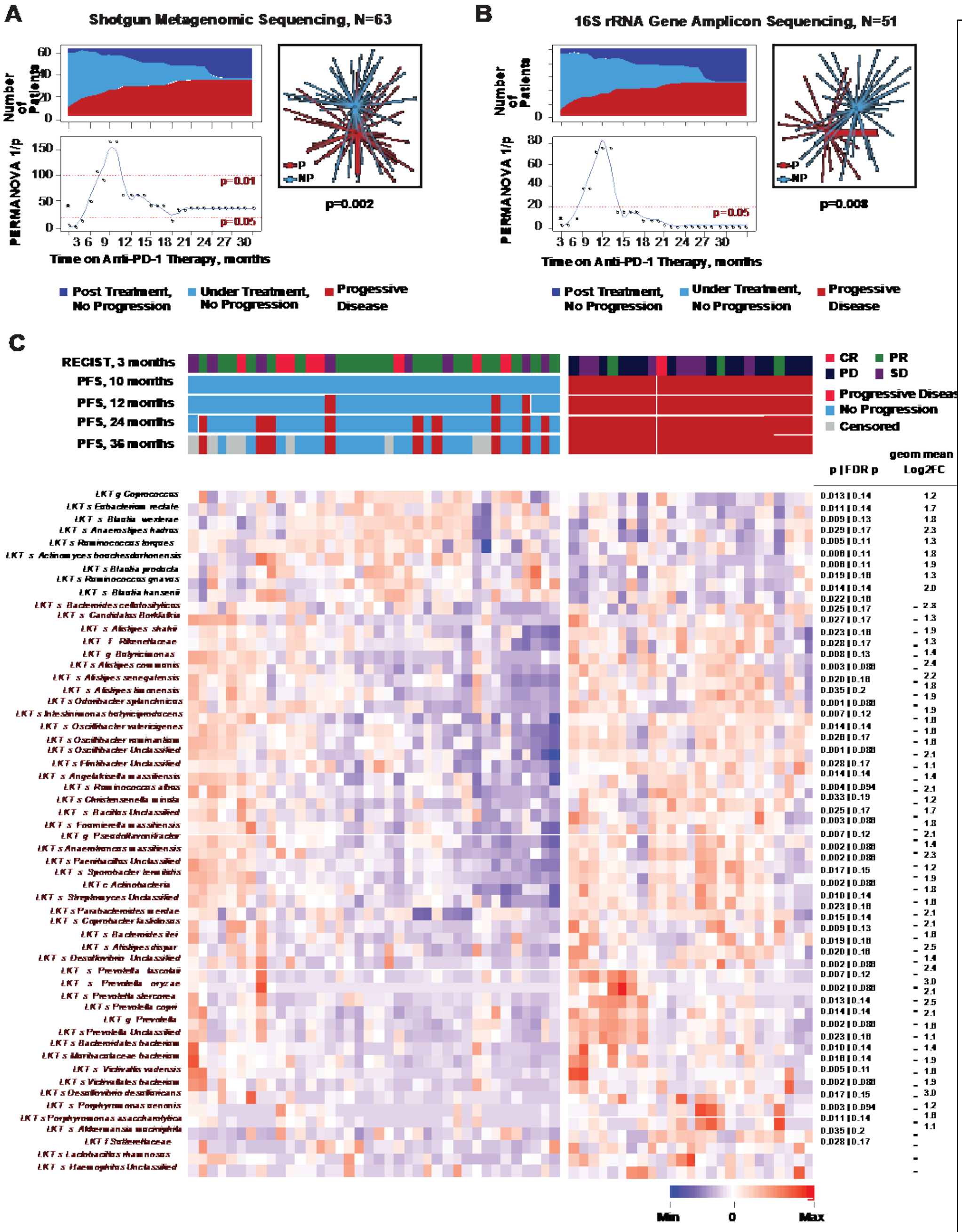

Figure 3. Compositional differences in the pre-treatment gut microbiome of anti-PD-1 treated melanoma patients are associated with differential progression-free survival (PFS). A and **B.** Evaluation of effects of initial (stools collected pre-treatment or up to 4 months on treatment) fecal microbiome composition upon response to anti-PD-1 therapy was assessed using shotgun metagenomic (**A**) or 16S rRNA gene amplicon (**b**) sequencing. Objective radiographic response to therapy was evaluated by treating investigators using RECIST v1.1 every 3 months, and progression was defined based on radiographic or clinical progression at each treatment visit (every 3 or 4 weeks). **Top left panels** depict number of patients on follow up at each timepoint in relation to response status (light blue – ongoing therapy, non-progressor; dark blue – off therapy, non-progressor; red – progressor). Compositional differences of the initial microbiome using PERMANOVA 1/p (y-axis, **bottom left panels**) were evaluated in progressors (P) and non-progressors (NP) identified at each treatment visit, thus permitting inference of the optimal time (9-10 months) to predict impact of microbiome composition upon response; the asterisk indicates PERMANOVA 1/p for RECIST v1.1 response (CR or PR) at 3 months. **Right panels** show t-distributed Uniform Manifold Approximation and Projection (t-UMAP) plots depicting fecal microbiota compositional differences between NP (blue) and P (red) at time of maximal difference from start of therapy (10 months). Empty black circles represent centroids with lines connecting them to corresponding samples from each group. p values were calculated using PERMANOVA. (**C**). Metagenomic shotgun sequencing of fecal microbiota samples identifies differentially abundant taxa in P vs. NP at 10 months from start of therapy. Heatmap shows differentially abundant taxa identified by metagenomic shotgun sequencing [false discovery rate (FDR) < 0.2 and fold change (FC) > 2]. Columns denote patients grouped by progression status and clustered within P/NP groups; rows denote bacterial taxa enriched (black) or depleted (red) in P versus NP clustered based on microbiota composition.

We had previously observed that non-response to anti-PD-1 was associated with increased systemic inflammatory tone[58], as evidenced by elevated circulating levels of neutrophil-to-lymphocyte ratio (NLR)[59-61] and interleukin 8 (IL-8)[62,63] – biomarkers with well characterized adverse impacts upon response to anti-PD-1 in melanoma and other solid cancers[59-63]. To evaluate the role of the microbiome upon systemic inflammation, we studied metagenomic composition in relation to markers of systemic inflammation in a large prospectively sampled cohort of patients treated with anti-PD-1. We observed that increased NLR correlated with worse clinical outcome in the Pittsburgh. Baseline fecal microbiome composition was significantly different in patients with high and low NLR (**Fig. S4A**). To evaluate the impact of baseline intestinal microbiome composition upon host tissues, we performed non-invasive transcriptomic analyses of cells shed into the intestinal lumen using next generation RNA sequencing of stool samples as described previously[64]. We identified that genes encoding pro-inflammatory cytokines (*IL1B* and *CXCL8*), transcription factors (*NFKBIZ, NFKBIA, TNFAIP3*, and *LITAF*), and superoxide dismutase (*SOD2*) were increased in progressing patients (Ps) (**Fig. S4B**). Conversely, non-progressors (NPs) exhibited increased expression of genes encoding membrane associated mucins (*MUC13*, and *MUC25*) and apolipoproteins (*APOA1, APOA4*, and *APOB*) (**Fig. S4B**). Ingenuity pathway analysis (IPA) of upstream regulators of

differentially expressed host genes identified LPS as a major contributor of pro-inflammatory gene signature identified in Ps (**Fig. S4C**). **Overall, our findings identify a TNF/CXCL8/IL-1β-mediated systemic inflammatory program triggered by LPS that is associated with a high systemic inflammatory tone and non-response to anti-PD-1.**

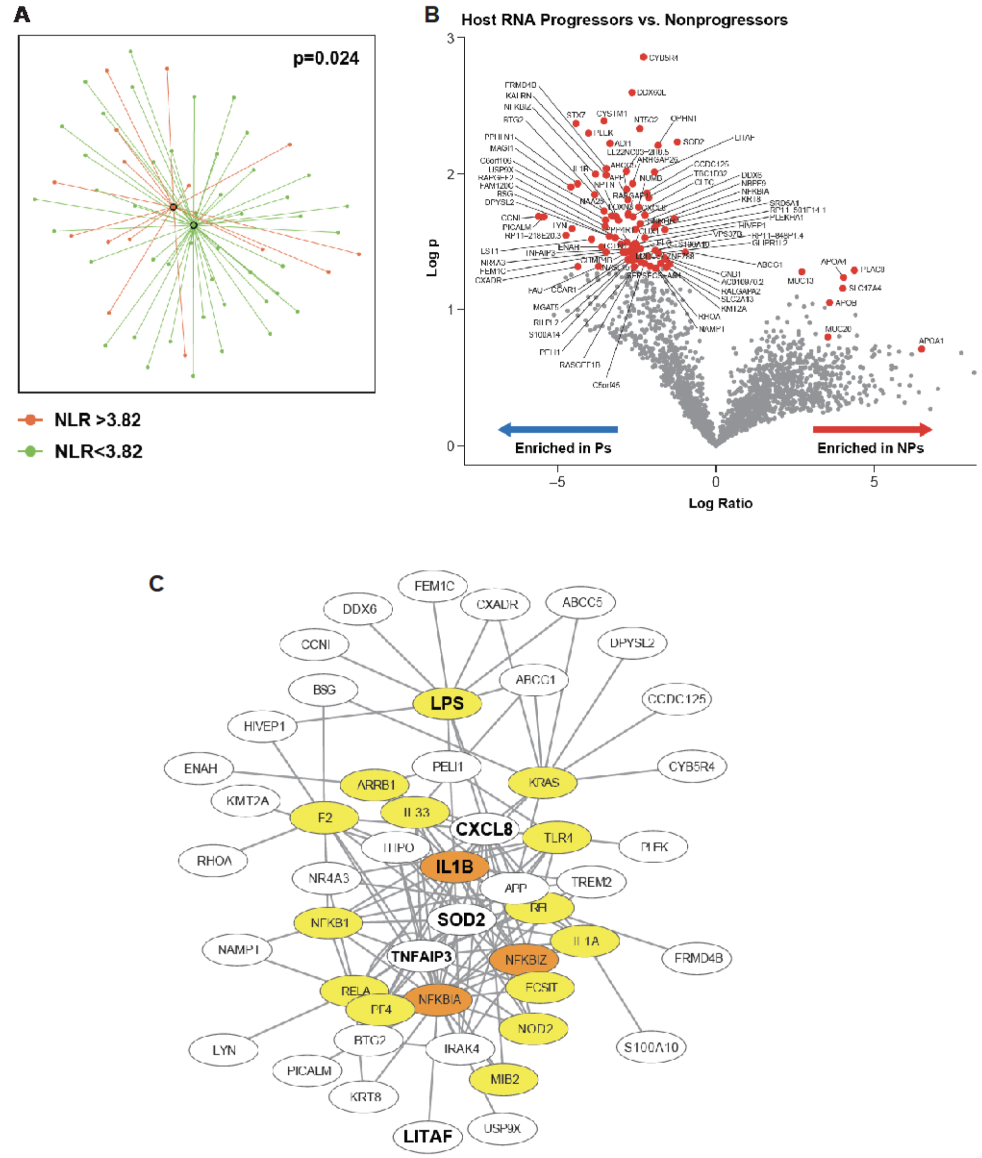

Figure 4. Relationship between microbiota composition, blood neutrophil/lymphocyte ratio (NLR) and associated host variables in relation to clinical response. (A) t-UMAP plots depicting fecal microbiota compositional differences between patient groups with high (≥ 3.82; orange) and low (< 3.82; green) pre-treatment NLR. Optimal NLR ratio cutoff (3.82) was determined as described in Methods. p value was calculated using PERMANOVA. (B) Volcano plot of differentially expressed human host genes identified in stool specimens as described in Methods from NPs compared to Ps at 10 months in anti-PD-1 treated melanoma patients. (C) Ingenuity pathway analysis of upstream regulators of differentially expressed host genes identified in (b). Predicted upstream regulators highlighted in yellow, while genes identified in (b) that were predicted upstream regulators are highlighted in orange.

Immune-related Adverse Events (irAE): Treatment and Burden

Immune-related adverse events (irAEs) represent an unique spectrum of toxicities with the use of anti-PD-1 and/or anti-CTLA-4 ICI that result from off-tumor mmune activation and can involve nearly any organ system. The reported incidence of any-grade irAEs is as high as 90% in studies with CTLA-4 inhibitor ipilimumab[65]; however, meta-analyses indicate an overall incidence of ~75% with anti-CTLA-4 ipilimumab[66] and ~30% in phase 3 trials of anti-PD-(L)1[67-69] across various histologies. IrAEs of ≥Grade 3 severity occur ~43% of melanoma patients with ipilimumab[65], ~20% with anti-PD-(L)1[67,70] across histologies, and ~33-55% with PD-1/CTLA-4 dual blockade[27,30,32]. The incidence of irAEs with ipilimumab and pembrolizumab is dose-dependent, with greater toxicity at higher dose levels and greater toxicity in the metastatic compared to the adjuvant disease settings[65,71-74]. The incidence of steroid-refractory irAEs is summarized in **Table 2**.

Table 2: Incidence of Select Steroid-Refractory irAEs in ICI Treated Patients

	Colitis[75,76]		Hepatitis[77]	MSK[78]
**ICI(s)**	PD-1, CTLA-4, PD-1/CTLA-4	PD-1, CTLA-4, PD-1/CTLA-4	PD-1, CTLA-4, PD-1/CTLA-4	PD-1, CTLA-4, PD-1/CTLA-4
**Sample size**	33	92	257	29

**Incidence of steroid-refractory disease**	66%	56%	36%	75%

We correlated irAE occurrence with intestinal microbome composition; and demonstrated that patients with irAEs had different metagenomic compositions compared to patients without irAEs (**Fig S5A**, p= 0.034); and that irAE development was associated with improved PFS, concordant with prior reports (**Fig. S5C**, p=0.0263)[79-81]. We identified distinct signatures of irAEs with opposing effects upon the anti-PD-1 response correlated with irAEs, and specifically identified an association between bacteria associated with elevated myeloid program with development of colitis (**Fig. S5B, 5D**). These data are inline with prior reports linking pre-treatment intestinal microbiome composition to the development of certain irAE **particularly colitis**[51,82]. **Specifically, we identified distinct metagenomic signatures associated with irAEs involving T cell rich barrier organs including colon, skin and lungs.**

Separately, single-cell analyses of intestinal luminal samples from ICI-treated patients who developed colitis identified transcriptionally-distinct CD4/CD8 T cells (characterized by *IFNG*, *GBP5*, *HLA-DR* and *CD74* expression) and myeloid cells (characterized by *TNF*, *IL1B*, and *OSM* expression) that drove the emergence of CD8 T effector cells[83]. Preclinically, the extent of dextran sulfate sodium (DSS)-mediated intestinal inflammation was mediated by the NLRP3 inflammasome and dependent upon IL-1β, induced by commensal microbiota[84], a pathway identified in ICI-treated colitis as well[85]. **Collectively, these data implicate TNF/CXCL8/IL-1β driven intestinal myeloid cells in the etiopathogenesis of ICI-colitis.**

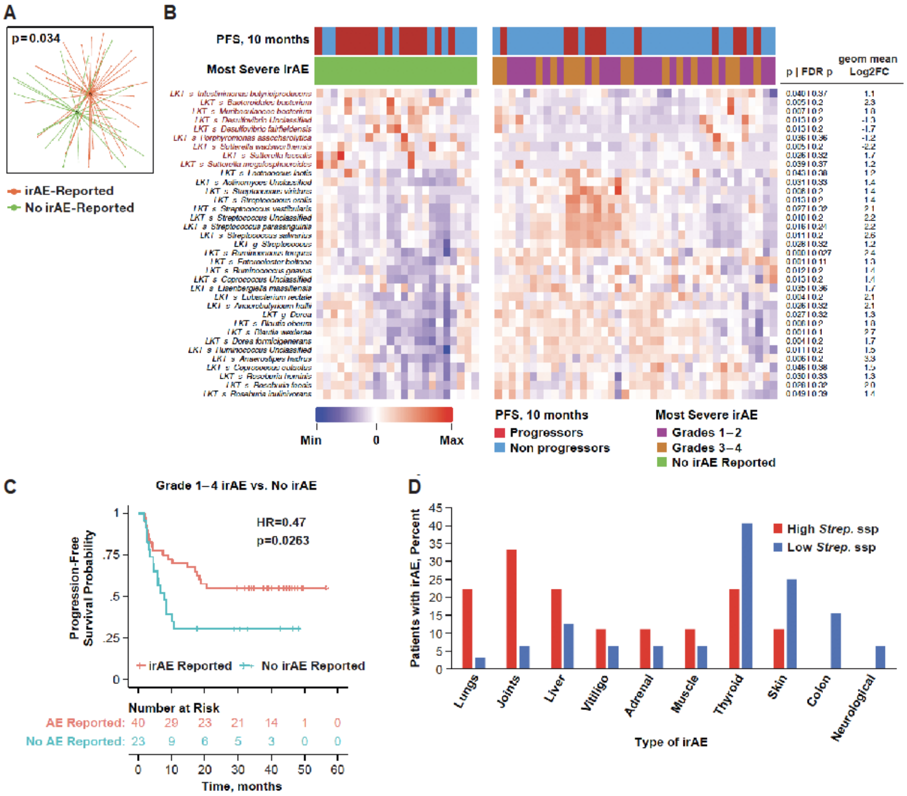

Figure 5. Fecal microbial signatures are differentially associated with immune-related adverse events (irAEs) and PFS in PD-1-treated melanoma patients. (**A**) tUMAP plot depicting compositional differences between melanoma patients who developed any irAEs (orange) and those who did not (green) at any timepoint from start of anti-PD-1 therapy. p values were calculated using PERMANOVA. (**B**) Heatmap of differentially abundant taxa (p<0.05 and FC >2) in patients with no irAEs (green) vs. grade 1-4 irAEs (1-2: purple, 3-4: orange, based on highest grade irAE reported). irAE severity was graded with CTCAE v5.0. Columns depict patients grouped by irAE grades and clustered within corresponding groups based on gut microbiome composition. Rows depict bacterial taxa enriched (black) or depleted (red) in melanoma patients with irAEs (grade 1-4) and clustered based on gut microbiota composition. (**C**) Kaplan-Meier plot of PFS probability based on irAE status (red – irAEs reported vs. green – irAEs not reported). Numbers of patients at risk at each time point are shown. (**D**) Percentages of PD-1 treated melanoma patients exhibiting specific types of irAE segregated by relative abundance of the 7 Streptococcus spp. Identified in (**B**) (8 patients clustered in the high group; high – red vs. low – blue).

The effects of dietary composition on immune regulation are complex and interlinked but involve both direct effects on immune cells via receptor-mediated signaling; and indirect effects via modulation of intestinal bacterial composition and consequent effects on host immune-metabolic homeostasis. Examples of direct effects include cruciferous vegetables which contain natural aryl hydrocarbon receptor (AhR) ligands[86]. AhR signaling promotes epithelial repair, supports intestinal lymphoid cells (ILCs) mediated intestinal lymphoid follicles (ILFs) formation required for intestinal IgA production and promotes IL-22 production by RORγt+ ILCs[87]. Dietary fibers are metabolized exclusively by the gut microbiome into short-chain fatty acids (SCFAs) which by signaling through G-protein coupled receptors (GPR41, GPR43, GPR109A) are implicated in cytokine and chemokine production by intestinal epithelial cells[88], T reg differentiation[89,90], and maintenance of intestinal epithelial integrity through NLRP3 inflammasome activation[91]. An integrated evaluation of dietary composition

along with blood/serum/tumor analyses combined with stool metagenomic sequencing may help clarify the bacterial species most tightly associated with response to PD-1 blockade.

Given the diverse host-dependent variables known to affect microbial composition, we propose to utilize a validated NHANES Diet History Questionnaire (DHQ-III; https://www.dhq3.org/study/questionnaires/) that incorporates questions regarding dietary intake. This DHQ-III permits the dietary intake of an individual patient to be analyzed for caloric content; but also to be broken down in to analytical components of interest including saturated and unsaturated fat, protein, carbohydrates, and dietary fiber.

2. <u>Rationale:</u>

We propose to develop a biorepository of microbiome samples along with blood and tumor tissue (**HCC Microbiome Initiative**).

The overarching goal of this initiative is to understand how the gut microbiome affects response to immunotherapies including immune checkpoint inhibitors (PD-1, CTLA-4 etc.), cell therapies and other novel agents. To do this, we will collect and bank microbiome specimens. Additionally, we will gather host-specific data including clinical response information, dietary intake, along with tumor and serological sampling.

In patients with available metagenomic data, the above variables (demographic and clinical variables and dietary information) along with response to PD-1 blockade will be used to construct an artificial neural network that integrates metagenomic, metatranscriptomic, metafunctional and phylogenetic information from microbial sequencing along with dietary information in order to determine the bacterial species most causally linked with response to immunotherapies.

This approach permits us to identify the microbial taxa that are most directly (causally) linked to immunotherapies independently, or not of demographics or other clinical variables (e.g., age, gender, smoking status, etc). This computational approach is more powerful, flexible and informative compared to regression or correlation methods.

3. <u>Aims:</u>

To assess intestinal microbial composition by metagenomic sequencing; and assess how various components of this (diversity, composition etc.) are associated with response to immunotherapies including immune checkpoint inhibitors (PD-1, CTLA-4 etc.), cell therapies and other novel agents.

To assess dietary intake variables including but not limited to saturated and unsaturated fat, protein, carbohydrates, and dietary fiber); and assess how these are related to intestinal microbial composition and response to immunotherapies including immune checkpoint inhibitors (PD-1, CTLA-4 etc.), cell therapies and other novel agents.

To assess serological variables including but not limited to serum IGF1, IL-6, TNF-α; and assess how these are related to intestinal microbial composition and response to immunotherapies including immune checkpoint inhibitors (PD-1, CTLA-4 etc.), cell therapies and other novel agents.

4. <u>Methods:</u>

This study comprises three parts as described below and outlined in **Figure 1**.

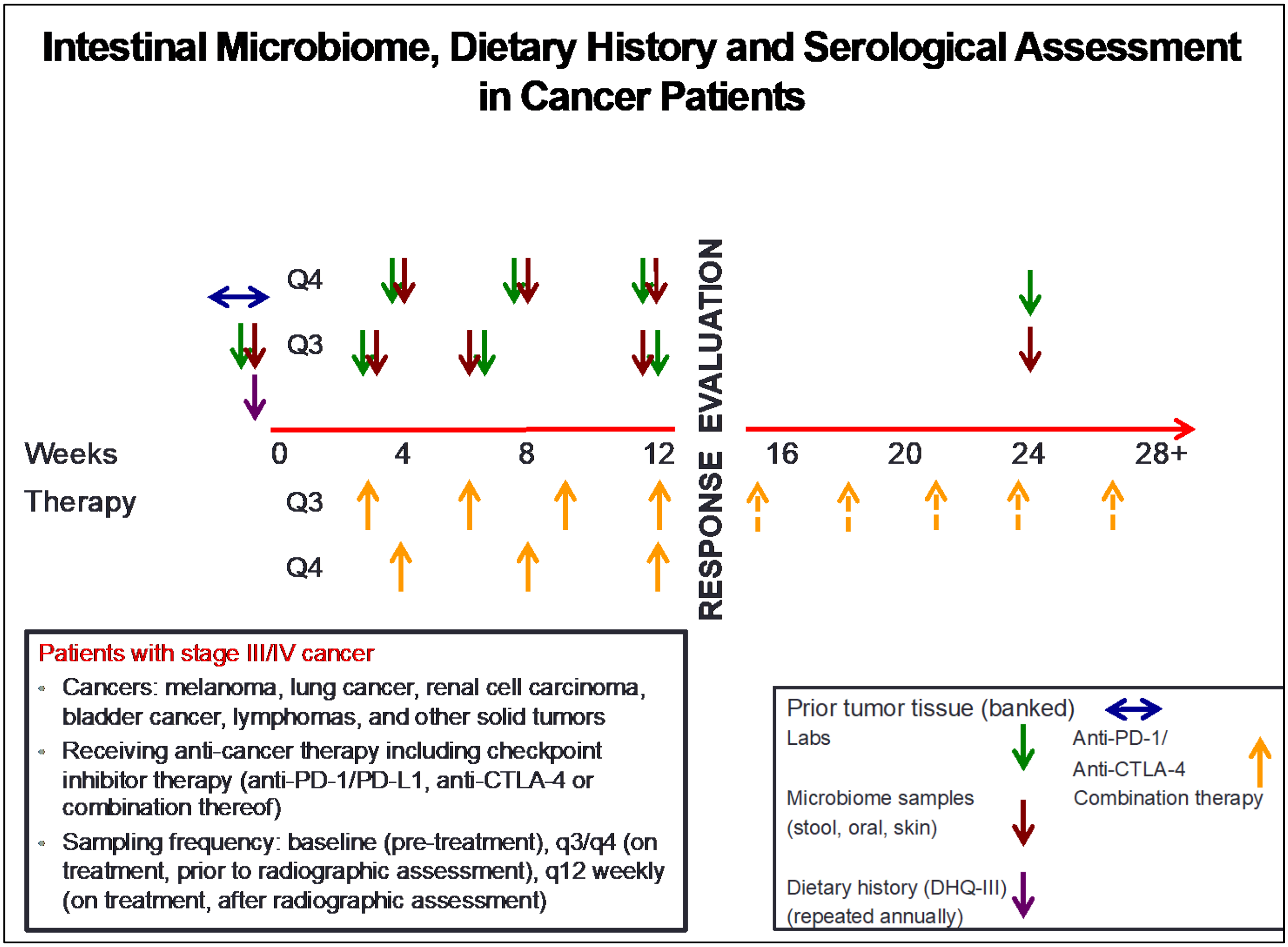

In **Part 1**, **microbiome samples (fecal, oral and skin)** from patients receiving immunotherapies including immune checkpoint inhibitors (PD-1, CTLA-4 etc.), cell therapies or any investigational combination thereof will be obtained at baseline (pre-treatment), at periodic intervals (on-treatment) and if "*event of interest*" (as defined in **Section 5**) occurs by study investigators (or designee). Sampling schedule is delineated in **Schedule of Events** (**Section 6.0**) and sampling methods are detailed in attached appendices (see **Appendix 1**). After radiographic assessment, patients who respond will continue to be sampled on an as needed basis. There is a window for samples to be collected and the absence of collection will not count as a deviation.

Samples will undergo metagenomic sequencing. Sequencing results will be correlated with clinical outcomes by artificial neural network analyses.

In **Part 2**, **Diet History Questionnaire (DHQ-III)** will be administered by study investigators (or designee) to patients receiving immunotherapies including immune checkpoint inhibitors (PD-1, CTLA-4 etc.), cell therapies or any investigational combination thereof. DHQ-3 administration schedule is delineated in **Schedule of Events** (**Section 6.0**) and includes situations if an "*event of interest*" (as defined in **Section** 5) occurs. Sampling methods are detailed in attached appendices (see **Appendix 2**). After radiographic assessment, patients who respond will continue to be sampled on an as needed basis. There is a window for samples to be collected and the absence of collection will not count as a deviation.

DHQ-III permits the dietary intake of an individual patient to be analyzed for caloric content; but also to be broken down in to analytical components of interest including saturated and unsaturated fat, protein, carbohydrates, and dietary fiber. Dietary variables will be summarized. ORR, PFS and OS, will be analyzed using the Kaplan-Meier method along with the corresponding exact 95% confidence intervals. Clinical variables including information pertaining to therapy (dose, duration), side effects of therapy (nature, severity, dates of initiation/resolution, therapy, etc.) and response (sites of disease, response to therapy) will be obtained by chart review.

In **Part 3**, blood/tissue samples from these patients will be obtained. Blood biospecimen sampling schedule is delineated in **Schedule of Events** (**Section 6.0**) and includes situations if an "*event of interest*" (as defined in **Section 5**) occurs. There is a window for samples to be collected and the absence of collection will not count as a deviation. At each timepoint, approximately **45mL of blood** will be collected in 3x 8mL sodium heparin (green top) tube, 1x 5mL SST (gold top) tube and 2 x 10mL cfDNA Streck tube.

Nature of analyses will include serum IgG sequencing, immunohistochemistry, T cell immunophenotyping, trancriptomic analyses (RNAseq) and whole exome sequencing (DNAseq). No biopsies will be obtained solely for the purpose of this study. Any prior tissue specimens obtained *or* a portion of any specimen(s) obtained for routine clinical care will be utilized for this.

5. Inclusion and Exclusion Criteria:

- Inclusion criteria
    - Be willing and able to provide written informed consent for the study.
    - Be ≥ 18 years of age on day of signing informed consent.
    - Undergoing treatment with immunotherapies including immune checkpoint inhibitors (ICI) and cell therapies – defined as PD-(L)1 blockade, PD-1/CTLA-4 blockade or any investigational combination or autologous T cell therapy – for any stage cancer including locally advanced (stage II-III) or advanced (stage IV) cancer of any histology.
        - **ICI-NEW:** Patients who are about to receive ICI therapy will be consented prior to onset of therapy. The schedule of events will be as outlined in **Section 6.0**.
        - **ICI-EXISTING:** Patients who have already started ICI therapy will be consented upon identification by study investigators (or designee). The schedule of events will be as outlined in **Section 6.0**.

        - **ICI-COMPLETED:** Patients who have completed ICI therapy and are currently on surveillance follow up will be consented upon identification by study investigators (or designee). The schedule of events will be as outlined in **Section 6.0**.
        - **ECI-COLITIS:** Patients who are receiving ICI therapy and develop confirmed or suspected colitis will be consented upon colitis diagnosis by study investigators (or designee). The schedule of events will be as outlined in **Section 6.0**.
        - **TIL-NEW:** Patients who are about to receive autologous T cell therapy will be consented prior to onset of therapy. The schedule of events will be as outlined in **Section 6.0**.
    - Events of clinical interest (ECI):
        - **ECI-COLITIS:** Patients who are receiving ICI therapy and develop confirmed or suspected colitis will be consented upon colitis diagnosis by study investigators (or designee). The schedule of events will be as outlined in **Section 6.0**.
        - Immune related adverse events of ≥Grade 3 greater severity (per CTCAE v5.0) including but not limited to pneumonitis, colitis, hypothyroidism, liver dysfunction, skin rash (including vitiligo, bullous dermatoses), hypophysitis, type 1 diabetes, renal dysfunction, myasthenia gravis, neuropathy, myositis, and uveitis.
        - Other ECI may be designated at investigator discretion including Grade 1-2 events.

- Exclusion criteria
    - Incapable of providing written informed consent.

6. Schedule of Events

Table 6.1: Schedule of Events

Procedure Assessment	Screening	Sampling Prior to Response Assessment			Sampling After Response Assessment	ECI[D]	ECI-COLITIS[E]	Surveillance Follow Up/ICI-COMPLETED[H]
		Cycle 1 (week 3 +/- 2 weeks)	Cycle 2 (week 3 +/- 2 weeks)	Cycle 3/4 (week 12 +/- 2 weeks)	Cycle X			
**Microbiome sampling[A]**<br>• **Stool**<br>• **Saliva swab**<br>• **Skin swab**	X	X	X	X	Every 12 weeks<br>X	X[G]<br>X[G]<br>X[G]	X	X
**DHQ-3[B]**	X					X[G]		
**Blood biospecimen[C]**	X	X	X	X	X (every 12 weeks)	X[G]	X	X
**Events of clinical interest(ECI)[D]**						X		
**ECI-COLITIS[E]**							Colitis specific testing<br>• Gut biopsies	
**ICI-NEW[F]**	X	X	X	X				
**ICI-EXISTING[G]**					X (every 12 weeks)			
**ICI-COMPLETED[H]**								X
**TIL-NEW[I]**								

Study Calendar Notes:

- [A]**Microbiome sampling**. Performed at the schedule depending on clinical status: ICI-NEW[E], ICI-EXISTING[F], ICI-COMPLETED[G].
  - Sampling methodology delineated in **Appendix 1**.
- [B]**DHQ-3 dietary assessment**. Performed at the schedule depending on clinical status: ICI-NEW[E], ICI-EXISTING[F], ICI-COMPLETED[G].
  - Sampling methodology delineated in **Appendix 2**.
  - In addition to DHQ-3, other questionnaires including food frequency questionnaires, and recall based questionnaires may be collected.
- [C]**Blood biospecimen banking**. Performed at the schedule depending on clinical status: ICI-NEW[E], ICI-EXISTING[F], ICI-COMPLETED[G].
  - Sampling methodology delineated below.
- [D]**Events of clinical interest (ECI).**
  - Defined as immune-related adverse events (irAE) of ≥Grade 3 greater severity (per CTCAE v5.0) although other events may be designated as ECI depending on study investigator(s).
  - Microbiome, DHQ-3 and blood biospecimens will be collected at this time.
  - These events will take place at the time of the patients' existing surveillance appointment. The patient will not be required to return at a separate time for sampling. The performance of these events is expected to take ~30mins.

- [E]**ECI-COLITIS**. Defined as patients with cancer meeting inclusion criteria (**Section 5.0**) who have clinical suspicion of imAE colitis.
    - Patients will be consented prior to colonoscopy.
    - Microbiome, DHQ-3 and blood biospecimens will be collected prior to/during 1st colonoscopy and repeated prior to/during 2nd colonoscopy.
    - Colonoscopy specimens will be processed as outlined in **Appendix 2.**
- [F]**ICI-NEW.** Defined as patients with cancer meeting inclusion criteria (**Section 5.0**) about to start therapy receive immune checkpoint inhibitor therapy (with anti-PD-1/PD-L1 blockade, PD-1/CTLA-4 blockade or any investigational combination).
    - Patients will be consented prior to onset of therapy.
    - Microbiome, DHQ-3 and blood biospecimens will be collected. Schedule of events as outlined above.
    - These events will take place at the time of the patients' existing anti-cancer therapy. The patient will not be required to return at a separate time for sampling. The performance of these events is expected to take ~30mins.
- [G]**ICI-EXISTING.** Defined as patients with cancer meeting inclusion criteria (**Section 5.0**) who are already on immune checkpoint inhibitor therapy (with anti-PD-1/PD-L1 blockade, PD-1/CTLA-4 blockade or any investigational combination).
    - Patients will be consented upon identification by study investigators (or designee).
    - Schedule of events as outlined above.
    - These events will take place at the time of the patients' existing anti-cancer therapy. The patient will not be required to return at a separate time for sampling. The performance of these events is expected to take ~30mins.
- [H]**ICI-COMPLETED.** Defined as patients with cancer meeting inclusion criteria (**Section 5.0**) who have completed a course of immune checkpoint inhibitor therapy (with anti-PD-1/PD-L1 blockade, PD-1/CTLA-4 blockade or any investigational combination) and are currently on surveillance alone.
    - Patients will be consented upon identification by study investigators (or designee).
    - Microbiome, DHQ-3 and blood biospecimens will be collected. Schedule of events will follow standard follow-up schedule as set the discretion of the treating physician.
    - These events will take place at the time of the patients' existing surveillance appointment. The patient will not be required to return at a separate time for sampling. The performance of these events is expected to take ~30mins.
- [I]**TIL-NEW.** Defined as patients with cancer meeting inclusion criteria (**Section 5.0**) who are pending initiation of autologous T cell therapy.
    - Patients will be consented upon identification by study investigators (or designee).
    - Microbiome, DHQ-3 and blood biospecimens will be collected.
    - Schedule of events will be as follows: **Screening, pre-NMA-LD (D-7 to D-5), pre-TIL (D0), post-TIL (D+5-10), count recovery (D+14 or later) and response assessment (D+60-90).**
    - These events will take place at the time of the patients' existing inpatient and/or outpatient stay. The patient will not be required to return at a separate time for sampling. The performance of these events is expected to take ~30mins.

7. Trial Procedures

The **Trial Flow Chart** in **Section 6.0** summarizes the trial procedures to be performed at each visit. Individual trial procedures are described in detail below.

There are multiple categories of patients (**ICI-NEW**, **ICI-EXISTING**, **ICI-COMPLETED, ECI** and **TIL-NEW**) in this study who will undergo a different schedule of events depending on category as summarized below.

7.1. ICI-NEW

7.1.1. Patients who are about to receive ICI therapy will be consented prior to onset of therapy.

7.1.2. These patients will be identified and consented prior to start of immunotherapy. They will undergo microbiome sampling during Screening, during each cycle of therapy prior to response assessment and every 12 weeks after fist response assessment.

7.1.3. Patients will only be seen during visits for treatment purposes and no additional study-specific visits will be scheduled. The performance of these events is expected to take ~30mins.

7.1.4. Patients may be resampled upon experiencing ECI (see **Section 7.4**).

7.1.5. Patients who complete therapy for reasons without experiencing disease progression (including patients who do not progress but discontinue for intolerance/toxicity) will move into the **Surveillance Follow Up phase** and be sampled per the **ICI-COMPLETED** schedule (see **Section 7.3**).

7.2. ICI-EXISTING

7.2.1. Patients who have already started ICI therapy will be consented upon identification.

7.2.2. The schedule of events will be determined by whether the patients have already completed first response assessment.

7.2.2.1. For patients who are consented *prior to* first response assessment, patients will undergo microbiome sampling during each cycle of therapy prior to response assessment.

7.2.2.2. For patients who are consented *after* first response assessment, patients will undergo microbiome sampling every 12 weeks.

7.2.3. Patients will only be seen during visits for treatment purposes and no additional study-specific visits will be scheduled. The performance of these events is expected to take ~30mins.

7.2.4. Patients may be resampled upon experiencing ECI (see **Section 7.4**).

7.2.5. Patients who complete therapy for reasons without experiencing disease progression (including patients who do not progress but discontinue for intolerance/toxicity) will move into the Surveillance Follow Up phase and be sampled per the **ICI-COMPLETED** schedule (see **Section 7.3**).

7.3. ICI-COMPLETED

7.3.1. Patients who have completed treatment for a reason other than disease progression (including patients who do not progress but discontinue for intolerance/toxicity) will move into the **Surveillance Follow-up Phase**.

7.3.2. These patients will be identified and consented during **Surveillance Follow-up Phase**. They will undergo microbiome sampling, DHQ-3 assessment and blood biospecimen collection. Schedule of events will follow standard follow-up schedule as set the discretion of the treating physician.

7.4. ECI

7.4.1. Patients who experience immune-related adverse events (irAE) of ≥Grade 3 greater severity (per CTCAE v5.0) although other events may be designated as ECI depending on study investigator(s).

7.4.2. These patients will be identified and consented at the time of recognition of ECI.

7.4.3. There is no set schedule of events for ECI patients as the need for resampling will depend upon resolution of irAE.

7.4.4. However, patients will only be seen during visits for treatment purposes and no additional study-specific visits will be scheduled. The performance of these events is expected to take ~30mins.

7.5. TIL-NEW

7.5.1. Patients who are about to receive autologous T cell (TIL) therapy will be consented prior to onset of therapy.

7.5.2. These patients will be identified and consented prior to start of TIL therapy. They will undergo microbiome sampling during **Screening**, prior to receipt of non-myeloablative lymphodepletion (NMA-LD, typically D-7 to D-5), prior to receipt of TIL (D-1), post TIL and IL-2 (D+5-10), post count recovery (D+14 or after) and prior to response assessment (D+60-90).

7.5.3. Patients will only be seen during visits for treatment purposes and no additional study-specific visits will be scheduled. The performance of these events is expected to take ~30mins.

7.5.4.

7.6. Duration of Study Participation

7.6.1. Patients will remain study participants for duration of receipt of immunotherapy.

8. Sample Management

Stool samples: FMT lab (Lab 1.47)

Tissue and blood samples: FMT lab (Lab 1. 47)

Biological samples harvested in the context of this protocol will generally be processed in the FMT Laboratory under the direction of Diwakar Davar, M.D.. Specimens obtained at hospitals throughout the UPMC system, and Magee Women's Hospital, will be delivered by courier to (FMT Laboratory) for processing. Fresh specimens for culture will be immediately delivered to the FMT Laboratory.

- Intake
    - Tissue, blood and stool specimen(s) received must be accompanied by the following information: (a) patient's name; (b) date of birth; (c) sex; (d) date and time specimen was drawn/collected; (e) treating physician; (f) current therapy or HCC protocol number.
    - Each specimen received is assigned a de-identified accession number, logged, and entered into the computer database.
    - Dietary information intake will occur separately via procedure as detailed in **Appendix 3**.
- Processing
    - **Tissue, blood and stool specimen(s) will be processed as delineated in the attached lab manual.**
    - FMT Laboratory personnel will record temperatures of all freezers daily. Freezers are equipped with alarm systems operating 24 hours/day and with liquid nitrogen tanks for emergency use. A written record is maintained of all power failures and/or emergencies by the Bank personnel.
    - A Bank diagnostic file for each patient is initiated and the FMT Laboratory's Database will be initiated at the same time, or before (if patient seen previously).

9. Sample Disbursement

In order to obtain serum, lymphocytes, stool, tumor tissues or other specimens of individual patients or groups by stage or other phenotype, individuals must submit a proposal for the use of tissue or clinical annotation of tissues, to the Principle Investigator.

Procedure for specimen retrieval is delineated in Laboratory Manual.

10. Significance

The HCC Microbiome Initiative will organize the collection of dietary information and microbiome specimens for use by HCC and other outside investigators, for current or future planned research.

The HCC Microbiome Initiative will provide a resource for use in clinical research by HCC investigators and collaborating investigators.

11. Risks and Benefits

Implementation of the HCC Microbiome Initiative poses minimal risk to patients, since only stool specimens, venipuncture specimens, and tumor specimens that would be obtained during surgery or other standard of care procedures, beyond requirements of pathology assessment, will be required. The methodology as outlined for specimen accrual will further reduce any risk to the patient by ensuring that allocation of blood to the bank does not occur in individuals whose clinical condition necessitates minimizing phlebotomy. Record keeping is designed to maximize confidentiality.

12. Costs and Payments

Patients who participate in HCC Microbiome Initiative will not receive any payment or accrue any costs related to specimen collection and processing for research purposes. Payment will not be provided in the event that new products, tests, or treatments are developed or discovered.

Costs of the HCC Microbiome Initiative operations will be subsumed by the HCC and in part supported by the extramural research support through federal and foundation grants.

13. Data Safety and Monitoring Plan

Duration of study participation: patients will remain on study for the duration they are receiving immunotherapy (including investigational therapy). Each line of therapy will be treated separately (i.e. if a patient progressed on 1st line immunotherapy X and switches to 2nd line immunotherapy Y, the patient will undergo events per ICI-COMPLETED[F] and events per ICI-NEW[E]).

There is minimal risk to participants associated with HCC Microbiome Initiative banking protocol.

Decisions and analysis regarding data safety are made by the PIs, and the IRB will be notified of any change in risk/benefit ratio affecting decisions about study continuation.

If any literature becomes available which suggests that conducting this trial is no longer ethical, the study will be terminated and the IRB will be notified of the new findings.

Serious adverse events are not expected as this is a non-interventional study.

Appendix 1: Microbiome Sampling SOP

Appendix 1-1: Stool Sample Collection for Microbiome Sampling

Supplies provided:

- EasySampler® Stool Collection Kit or Zymo DNA/RNA Shield Fecal Collection Tube
- Collection instructions as below.
- Ensure the patient understands:
  - The sample obtained is of the patient's own stool (not a relative's or pet's).
  - Commode and remainder of the stool specimen can be discarded once material has been transferred into the Alpco EazySampler® Kit.
  - Alpco EazySampler® Kit should always be stored at room temperature and returned to the site at the next visit.

Instructions for Stool Collection with Alpco EazySampler® Kit

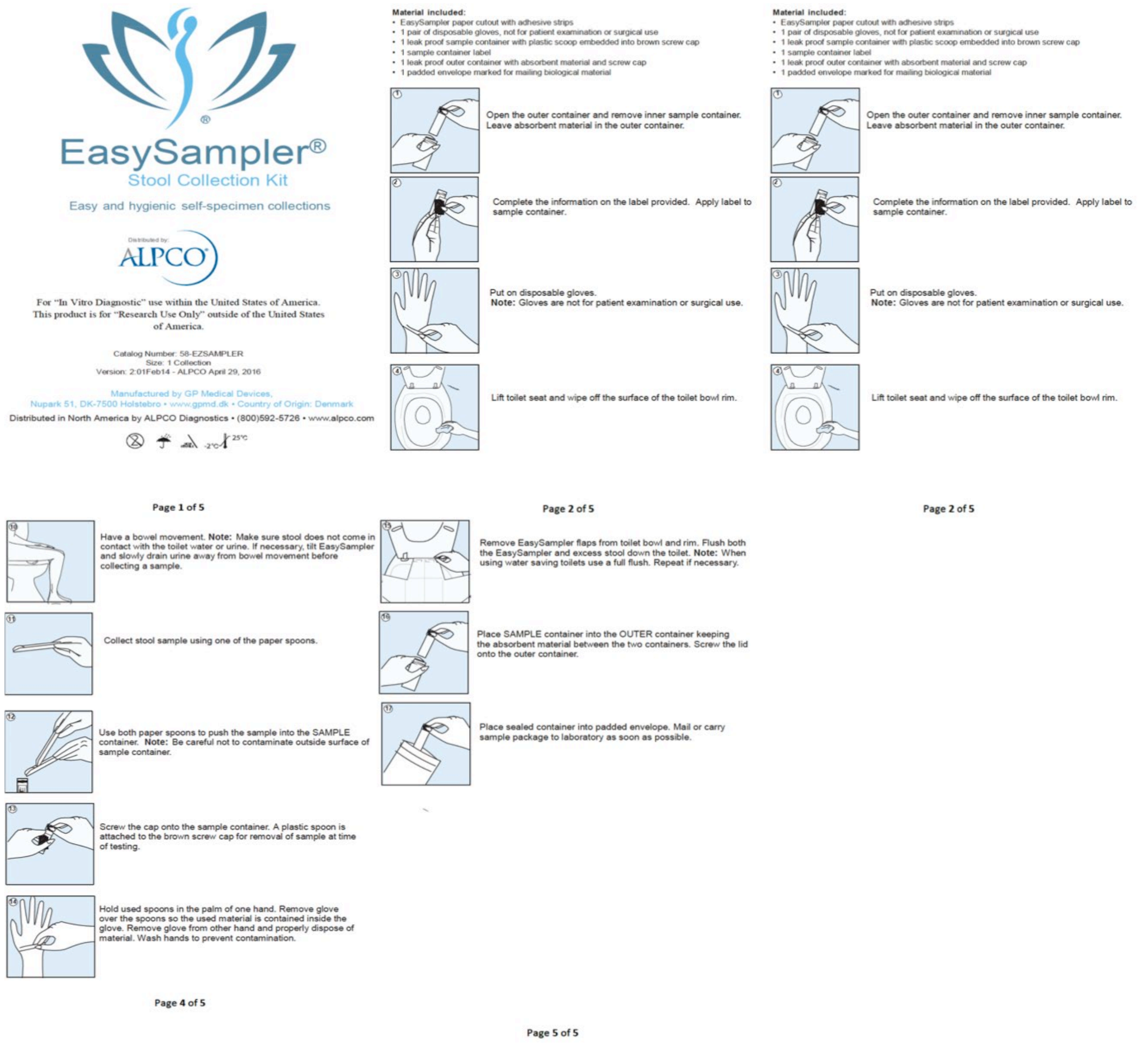

EasySampler®
Stool Collection Kit
Easy and hygienic self-specimen collections

Distributed by:
ALPCO

For "In Vitro Diagnostic" use within the United States of America. This product is for "Research Use Only" outside of the United States of America.

Catalog Number: 58-EZSAMPLER
Size: 1 Collection
Version: 2.01Feb14 - ALPCO April 29, 2016

Manufactured by GP Medical Devices,
Nupark 51, DK-7500 Holstebro • www.gpmd.dk • Country of Origin: Denmark
Distributed in North America by ALPCO Diagnostics • (800)592-5726 • www.alpco.com

-2°C 25°C

Page 1 of 5

Material included:
- EasySampler paper cutout with adhesive strips
- 1 pair of disposable gloves, not for patient examination or surgical use
- 1 leak proof sample container with plastic scoop embedded into brown screw cap
- 1 sample container label
- 1 leak proof outer container with absorbent material and screw cap
- 1 padded envelope marked for mailing biological material

1. Open the outer container and remove inner sample container. Leave absorbent material in the outer container.
2. Complete the information on the label provided. Apply label to sample container.
3. Put on disposable gloves. **Note:** Gloves are not for patient examination or surgical use.
4. Lift toilet seat and wipe off the surface of the toilet bowl rim.

Page 2 of 5

Material included:
- EasySampler paper cutout with adhesive strips
- 1 pair of disposable gloves, not for patient examination or surgical use
- 1 leak proof sample container with plastic scoop embedded into brown screw cap
- 1 sample container label
- 1 leak proof outer container with absorbent material and screw cap
- 1 padded envelope marked for mailing biological material

1. Open the outer container and remove inner sample container. Leave absorbent material in the outer container.
2. Complete the information on the label provided. Apply label to sample container.
3. Put on disposable gloves. **Note:** Gloves are not for patient examination or surgical use.
4. Lift toilet seat and wipe off the surface of the toilet bowl rim.

Page 2 of 5

10. Have a bowel movement. **Note:** Make sure stool does not come in contact with the toilet water or urine. If necessary, tilt EasySampler and slowly drain urine away from bowel movement before collecting a sample.
11. Collect stool sample using one of the paper spoons.
12. Use both paper spoons to push the sample into the SAMPLE container. **Note:** Be careful not to contaminate outside surface of sample container.
13. Screw the cap onto the sample container. A plastic spoon is attached to the brown screw cap for removal of sample at time of testing.
14. Hold used spoons in the palm of one hand. Remove glove over the spoons so the used material is contained inside the glove. Remove glove from other hand and properly dispose of material. Wash hands to prevent contamination.

Page 4 of 5

15. Remove EasySampler flaps from toilet bowl and rim. Flush both the EasySampler and excess stool down the toilet. **Note:** When using water saving toilets use a full flush. Repeat if necessary.
16. Place SAMPLE container into the OUTER container keeping the absorbent material between the two containers. Screw the lid onto the outer container.
17. Place sealed container into padded envelope. Mail or carry sample package to laboratory as soon as possible.

Page 5 of 5

Instructions for Stool Collection with Zymo DNA/RNA Shield Fecal Collection Tube®

DNA/RNA Shield™ Fecal Collection Tube

Catalog No. R1101

Quick Protocol

ZYMO RESEARCH
The Beauty of Science is to Make Things Simple

Description

The DNA/RNA Shield™ Fecal Collection Tube ensures sample stability during storage/transport at ambient temperatures without the need for refrigeration or specialized equipment. DNA/RNA Shield™ reagent effectively lyses samples and inactivates pathogens (e.g., virus, bacteria).

Each collection tube (with a spoon attached to the cap) is pre-filled with DNA/RNA Shield™ (9 mL). The nucleic acids (DNA & RNA) in samples are preserved at ambient temperature (DNA >1 year, RNA up to 1 month). Samples in the DNA/RNA Shield™ can be frozen (-20/-80°C) for prolonged storage.

Required Fecal Collection Accessories (Not included)

1. Fecal specimen collector set (e.g., hat-style specimen collector)
2. Labels for identification of samples
3. Appropriate waste container/biological waste container

Instructions

1. Prepare and collect fecal specimen using preferred fecal specimen collection set/kit.

   Note: Method of collecting the fecal sample must prevent feces from falling into toilet water to avoid sample contamination.
2. Unscrew the collection tube cap and use the spoon to scoop **one spoonful** of feces (approximately 1 gram or 1 mL in volume) from a sample.
3. Place the sample in the collection tube.
4. Tighten the cap and shake to mix the contents thoroughly (invert 10 times) to create a suspension.

   Note: Some fecal material may be difficult to re-suspend. As long as the material is suspended, the sample is stabilized. foaming/frothing during shaking is normal.
5. Dispose of unused fecal material and thoroughly wash hands according to your institution's guidelines.

Sample Purification

Samples in DNA/RNA Shield™ can be input directly into Zymo Research's (and others) nucleic acid purification kits.

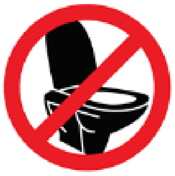

Don't let the sample go into the toilet

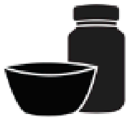

Collect stool into a clean container

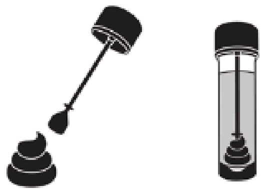

Scoop a portion of the stool sample into the DNA/RNA Shield™ Fecal Collection Tube

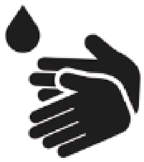

Wash hands well

Appendix 1-2: Skin Sample Collection for Microbiome Sampling

Supplies provided:

- BD BBL™ CultureSwab™ EZ II
- Collection instructions as below.
  - Sampling the skin microbiome with one single swab may limit volume of DNA to be used on more than one test.
  - To optimize downstream processes, we are using BD BBL™ CultureSwab™ EZ II (with two swabs) to maximize DNA content per sampling session.
  - Internal validations have shown that skin microbiome recovery is optimized using pre-moistened swabs as compared to dry swabs or skin tapes. This optimization results in overall higher microbial DNA content while minimizing host DNA content.

BASIC INSTRUCTIONS

1. **STEP 1**
   Dip a fresh swab in a moistening buffer such as saline or distilled water. With one hand, stretch the skin site taut. With the other hand, hold the swab so the shaft is parallel to the skin surface. Apply firm pressure and rub the swab back and forth rigorously 50 times (for 30 seconds).

2. **STEP 2**
   Carefully return the swab to the tube, ensuring that neither the swab, the swab stick nor the vessel touch any surface.

3. **STEP 3**
   Place the tube into a biospecimen bag and freeze until ready to ship.

Appendix 1-3: Oral Saliva Collection for Microbiome Sampling

Supplies provided:

- OmniGene ORAL (OM-501)
- Collection instructions as below.

USER INSTRUCTIONS

Most people take between 2 and 5 minutes to deliver a sample following steps 1 to 5.

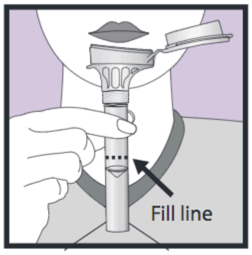

1 Spit into funnel until the amount of liquid (not bubbles) reaches the fill line shown in picture #1.

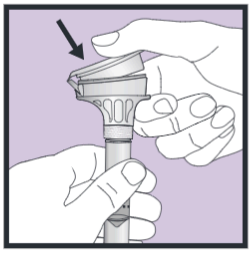

2 Hold the tube upright with one hand. Close the funnel lid with the other hand (as shown) by firmly pushing the lid until you hear a loud click. The liquid in the lid will be released into the tube to mix with the sample. Make sure that the lid is closed tightly.

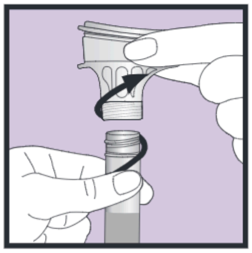

3 Hold the tube upright. Unscrew the funnel from the tube.

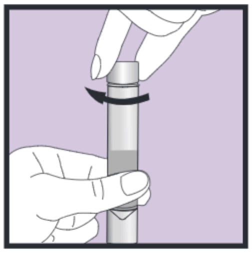

4 Use the small cap to close the tube tightly.

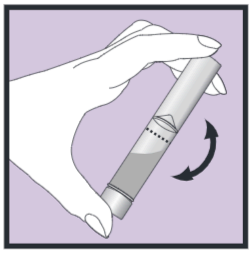

5 Shake the capped tube for 10 seconds. Discard or recycle the funnel.

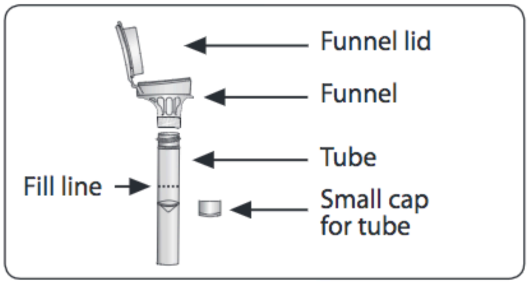

Collection precautions:

Do NOT eat, drink, smoke or chew gum for 30 minutes before giving your sample.

Do NOT remove the plastic film from the funnel lid.

Contents: Kit contains stabilizing liquid.

Warnings and precautions:
Wash with water if stabilizing liquid comes in contact with eyes or skin. Do NOT ingest. See MSDS at www.dnagenotek.com.

Small cap may pose a choking hazard.

Storage: 15°C–30°C

Summary and explanation of the kit:

OMNIgene•ORAL is a collection kit that provides the materials and instructions for collecting and stabilizing microbial DNA from oral fluids.

Label legend:

	Consult package insert
	Collect sample by (Use by)
REF	Catalog number
	Industrial Design Patent
	Caution, consult instructions for use
15°C–30°C	Storage instructions
	Manufacturer
LOT	Lot number

Collected specimen is potentially infectious and should be handled with appropriate biosafety practices.

Ship in accordance to applicable regulations covering transport of biological specimens.

Appendix 2: Gut Biopsy Sampling Schedule for ICI-COLITIS

	**1st colonoscopy**	**2nd colonoscopy (if performed)**
Inflamed tissue		
1st core (inflamed tissue)	Standard histopathology (ordered by GI)	Standard histopathology (ordered by GI)
2nd and 3rd core (inflamed tissue)	FFPE	FFPE
4th and 5th and 6th core (inflamed tissue)	Miltenyi medium	Miltenyi medium
7th and 8th core (inflamed tissue)	RNAlater	RNAlater
Uninflamed tissue (same patient control; if present)		
1st core (uninflamed tissue)	FFPE	FFPE
2nd and 3rd and 4th core (uninflamed tissue)	Miltenyi medium	Miltenyi medium
5th core and 6th (uninflamed tissue)	RNAlater	RNAlater
**Note:**<br>• **The colonoscopy request should state, "Enrolled in ICI colitis study, please obtain biopsies as above using jumbo forceps".**<br>• **The uninflamed tissue should be labelled by location.**		

Appendix 3: Dietary History Questionnaire for Microbiome Sampling

Dietary history questionnaire will be administered either on paper or electronically and data entered and stored securely electronically. Instrument will be administered by PI or designee.

In addition to DHQ-3, other questionnaires including food frequency questionnaires, and recall based questionnaires may be collected.

Detailed information for study staff:

1. Please obtain patient's username and password from study PI.
2. Please provide instructions (see below) to patients.
3. Patients are to be instructed to provide detailed dietary information either during visit (preferred) or at home

Detailed information for patients:

- Thank you for participating in this study. We are interested in evaluating your dietary history. To do this we are using a validated questionnaire termed the "Diet History Questionnaire (DHQ-3)".
- This questionnaire takes approximately 45 minutes to complete. You can do this either while receiving your therapy or at home.
- After completing the questionnaire, you will receive a Respondent Nutrition Report. This report shows estimated daily nutrient and food group intakes based on questionnaire responses. Recommended values are only available for some nutrients and food groups.
- Please feel free to discuss this with your study doctor.

Dietary study login URL:
Your username (case sensitive): __________
Your study password (case sensitive): __________

REFERENCES:

1. Dong, H., Zhu, G., Tamada, K. & Chen, L. B7-H1, a third member of the B7 family, co-stimulates T-cell proliferation and interleukin-10 secretion. *Nat Med* **5**, 1365-1369 (1999).
2. Freeman, G.J., *et al.* Engagement of the PD-1 immunoinhibitory receptor by a novel B7 family member leads to negative regulation of lymphocyte activation. *J Exp Med* **192**, 1027-1034 (2000).
3. Latchman, Y., *et al.* PD-L2 is a second ligand for PD-1 and inhibits T cell activation. *Nat Immunol* **2**, 261-268 (2001).
4. Tseng, S.Y., *et al.* B7-DC, a new dendritic cell molecule with potent costimulatory properties for T cells. *J Exp Med* **193**, 839-846 (2001).
5. Liang, S.C., *et al.* Regulation of PD-1, PD-L1, and PD-L2 expression during normal and autoimmune responses. *Eur J Immunol* **33**, 2706-2716 (2003).
6. Loke, P. & Allison, J.P. PD-L1 and PD-L2 are differentially regulated by Th1 and Th2 cells. *Proc Natl Acad Sci U S A* **100**, 5336-5341 (2003).
7. Brunet, J.F., *et al.* A new member of the immunoglobulin superfamily--CTLA-4. *Nature* **328**, 267-270 (1987).
8. Tivol, E.A., *et al.* Loss of CTLA-4 leads to massive lymphoproliferation and fatal multiorgan tissue destruction, revealing a critical negative regulatory role of CTLA-4. *Immunity* **3**, 541-547 (1995).
9. Waterhouse, P., *et al.* Lymphoproliferative disorders with early lethality in mice deficient in Ctla-4. *Science* **270**, 985-988 (1995).
10. Walunas, T.L., *et al.* CTLA-4 can function as a negative regulator of T cell activation. *Immunity* **1**, 405-413 (1994).
11. Brunner, M.C., *et al.* CTLA-4-Mediated inhibition of early events of T cell proliferation. *J Immunol* **162**, 5813-5820 (1999).
12. Freeman, G.J., *et al.* Uncovering of functional alternative CTLA-4 counter-receptor in B7-deficient mice. *Science* **262**, 907-909 (1993).
13. Freeman, G.J., *et al.* Cloning of B7-2: a CTLA-4 counter-receptor that costimulates human T cell proliferation. *Science* **262**, 909-911 (1993).
14. Linsley, P.S., Clark, E.A. & Ledbetter, J.A. T-cell antigen CD28 mediates adhesion with B cells by interacting with activation antigen B7/BB-1. *Proc Natl Acad Sci U S A* **87**, 5031-5035 (1990).
15. Linsley, P.S., *et al.* CTLA-4 is a second receptor for the B cell activation antigen B7. *J Exp Med* **174**, 561-569 (1991).
16. Linsley, P.S., *et al.* Human B7-1 (CD80) and B7-2 (CD86) bind with similar avidities but distinct kinetics to CD28 and CTLA-4 receptors. *Immunity* **1**, 793-801 (1994).
17. van der Merwe, P.A., Bodian, D.L., Daenke, S., Linsley, P. & Davis, S.J. CD80 (B7-1) binds both CD28 and CTLA-4 with a low affinity and very fast kinetics. *J Exp Med* **185**, 393-403 (1997).
18. Fourcade, J., *et al.* Upregulation of Tim-3 and PD-1 expression is associated with tumor antigen-specific CD8+ T cell dysfunction in melanoma patients. *J Exp Med* **207**, 2175-2186 (2010).
19. Wei, S.C., *et al.* Distinct Cellular Mechanisms Underlie Anti-CTLA-4 and Anti-PD-1 Checkpoint Blockade. *Cell* **170**, 1120-1133 e1117 (2017).
20. Das, R., *et al.* Combination therapy with anti-CTLA-4 and anti-PD-1 leads to distinct immunologic changes in vivo. *J Immunol* **194**, 950-959 (2015).
21. Robert, C., *et al.* Nivolumab in previously untreated melanoma without BRAF mutation. *N Engl J Med* **372**, 320-330 (2015).
22. Robert, C., *et al.* Pembrolizumab versus ipilimumab in advanced melanoma (KEYNOTE-006): post-hoc 5-year results from an open-label, multicentre, randomised, controlled, phase 3 study. *Lancet Oncol* **20**, 1239-1251 (2019).
23. Robert, C., *et al.* Pembrolizumab versus Ipilimumab in Advanced Melanoma. *N Engl J Med* **372**, 2521-2532 (2015).
24. Larkin, J., *et al.* Five-Year Survival with Combined Nivolumab and Ipilimumab in Advanced Melanoma. *N Engl J Med* **381**, 1535-1546 (2019).
25. Larkin, J., Hodi, F.S. & Wolchok, J.D. Combined Nivolumab and Ipilimumab or Monotherapy in Untreated Melanoma. *N Engl J Med* **373**, 1270-1271 (2015).
26. Wolchok, J.D., *et al.* Overall Survival with Combined Nivolumab and Ipilimumab in Advanced Melanoma. *N Engl J Med* **377**, 1345-1356 (2017).

27. Larkin, J., *et al.* Combined Nivolumab and Ipilimumab or Monotherapy in Untreated Melanoma. *N Engl J Med* **373**, 23-34 (2015).
28. Reck, M., *et al.* Pembrolizumab versus Chemotherapy for PD-L1-Positive Non-Small-Cell Lung Cancer. *N Engl J Med* **375**, 1823-1833 (2016).
29. Reck, M., *et al.* Updated Analysis of KEYNOTE-024: Pembrolizumab Versus Platinum-Based Chemotherapy for Advanced Non-Small-Cell Lung Cancer With PD-L1 Tumor Proportion Score of 50% or Greater. *J Clin Oncol* **37**, 537-546 (2019).
30. Hellmann, M.D., *et al.* Nivolumab plus Ipilimumab in Advanced Non-Small-Cell Lung Cancer. *N Engl J Med* **381**, 2020-2031 (2019).
31. Paz-Ares, L., *et al.* First-line nivolumab plus ipilimumab combined with two cycles of chemotherapy in patients with non-small-cell lung cancer (CheckMate 9LA): an international, randomised, open-label, phase 3 trial. *Lancet Oncol* (2021).
32. Motzer, R.J., *et al.* Nivolumab plus Ipilimumab versus Sunitinib in Advanced Renal-Cell Carcinoma. *N Engl J Med* **378**, 1277-1290 (2018).
33. Rini, B.I., *et al.* Pembrolizumab plus Axitinib versus Sunitinib for Advanced Renal-Cell Carcinoma. *N Engl J Med* **380**, 1116-1127 (2019).
34. McDermott, D.F., *et al.* Pembrolizumab monotherapy as first-line therapy in advanced clear cell renal cell carcinoma (accRCC): Results from cohort A of KEYNOTE-427. *Journal of Clinical Oncology* **36**, 4500-4500 (2018).
35. Motzer, R.J., *et al.* Nivolumab versus Everolimus in Advanced Renal-Cell Carcinoma. *N Engl J Med* **373**, 1803-1813 (2015).
36. Motzer, R.J., *et al.* Nivolumab plus ipilimumab versus sunitinib in first-line treatment for advanced renal cell carcinoma: extended follow-up of efficacy and safety results from a randomised, controlled, phase 3 trial. *Lancet Oncol* **20**, 1370-1385 (2019).
37. Tumeh, P.C., *et al.* PD-1 blockade induces responses by inhibiting adaptive immune resistance. *Nature* **515**, 568-571 (2014).
38. Taube, J.M., *et al.* Association of PD-1, PD-1 ligands, and other features of the tumor immune microenvironment with response to anti-PD-1 therapy. *Clin Cancer Res* **20**, 5064-5074 (2014).
39. Herbst, R.S., *et al.* Predictive correlates of response to the anti-PD-L1 antibody MPDL3280A in cancer patients. *Nature* **515**, 563-567 (2014).
40. Cristescu, R., *et al.* Pan-tumor genomic biomarkers for PD-1 checkpoint blockade-based immunotherapy. *Science (New York, N.Y.)* **362**, eaar3593 (2018).
41. Rizvi, H., *et al.* Molecular Determinants of Response to Anti-Programmed Cell Death (PD)-1 and Anti-Programmed Death-Ligand 1 (PD-L1) Blockade in Patients With Non-Small-Cell Lung Cancer Profiled With Targeted Next-Generation Sequencing. *J Clin Oncol* **36**, 633-641 (2018).
42. Huang, A.C., *et al.* A single dose of neoadjuvant PD-1 blockade predicts clinical outcomes in resectable melanoma. *Nat Med* **25**, 454-461 (2019).
43. Huang, A.C., *et al.* T-cell invigoration to tumour burden ratio associated with anti-PD-1 response. *Nature* **545**, 60-65 (2017).
44. Eggermont, A.M.M., *et al.* Adjuvant Pembrolizumab versus Placebo in Resected Stage III Melanoma. *N Engl J Med* **378**, 1789-1801 (2018).
45. Weber, J., *et al.* Adjuvant Nivolumab versus Ipilimumab in Resected Stage III or IV Melanoma. *N Engl J Med* **377**, 1824-1835 (2017).
46. Robert, C., *et al.* Long-term safety of pembrolizumab monotherapy and relationship with clinical outcome: A landmark analysis in patients with advanced melanoma. *Eur J Cancer* **144**, 182-191 (2020).
47. Dzutsev, A., Goldszmid, R.S., Viaud, S., Zitvogel, L. & Trinchieri, G. The role of the microbiota in inflammation, carcinogenesis, and cancer therapy. *Eur J Immunol* **45**, 17-31 (2015).
48. Finlay, B.B., *et al.* Can we harness the microbiota to enhance the efficacy of cancer immunotherapy? *Nat Rev Immunol* **20**, 522-528 (2020).
49. Goldszmid, R.S., *et al.* Microbiota modulation of myeloid cells in cancer therapy. *Cancer immunology research* **3**, 103-109 (2015).
50. Zarour, H.M. Reversing T-cell Dysfunction and Exhaustion in Cancer. *Clin Cancer Res* **22**, 1856-1864 (2016).

51. Chaput, N., *et al.* Baseline gut microbiota predicts clinical response and colitis in metastatic melanoma patients treated with ipilimumab. *Ann Oncol* **28**, 1368-1379 (2017).
52. Vetizou, M., *et al.* Anticancer immunotherapy by CTLA-4 blockade relies on the gut microbiota. *Science* **350**, 1079-1084 (2015).
53. Gopalakrishnan, V., *et al.* Gut microbiome modulates response to anti-PD-1 immunotherapy in melanoma patients. *Science* **359**, 97-103 (2018).
54. Matson, V., *et al.* The commensal microbiome is associated with anti-PD-1 efficacy in metastatic melanoma patients. *Science* **359**, 104-108 (2018).
55. Routy, B., *et al.* Gut microbiome influences efficacy of PD-1-based immunotherapy against epithelial tumors. *Science* **359**, 91-97 (2018).
56. Frankel, A.E., *et al.* Metagenomic Shotgun Sequencing and Unbiased Metabolomic Profiling Identify Specific Human Gut Microbiota and Metabolites Associated with Immune Checkpoint Therapy Efficacy in Melanoma Patients. *Neoplasia* **19**, 848-855 (2017).
57. Peters, B.A., *et al.* Relating the gut metagenome and metatranscriptome to immunotherapy responses in melanoma patients. *Genome Med* **11**, 61 (2019).
58. Davar, D., *et al.* Fecal microbiota transplant overcomes resistance to anti-PD-1 therapy in melanoma patients. *Science* **371**, 595-602 (2021).
59. Ascierto, P.A., *et al.* Proteomic test for anti-PD-1 checkpoint blockade treatment of metastatic melanoma with and without BRAF mutations. *J Immunother Cancer* **7**, 91 (2019).
60. Capone, M., *et al.* Baseline neutrophil-to-lymphocyte ratio (NLR) and derived NLR could predict overall survival in patients with advanced melanoma treated with nivolumab. *J Immunother Cancer* **6**, 74 (2018).
61. Valero, C., *et al.* Pretreatment neutrophil-to-lymphocyte ratio and mutational burden as biomarkers of tumor response to immune checkpoint inhibitors. *Nat Commun* **12**, 729 (2021).
62. Sanmamed, M.F., *et al.* Changes in serum interleukin-8 (IL-8) levels reflect and predict response to anti-PD-1 treatment in melanoma and non-small-cell lung cancer patients. *Ann Oncol* **28**, 1988-1995 (2017).
63. Schalper, K.A., *et al.* Elevated serum interleukin-8 is associated with enhanced intratumor neutrophils and reduced clinical benefit of immune-checkpoint inhibitors. *Nat Med* **26**, 688-692 (2020).
64. Knight, J.M., *et al.* Non-invasive analysis of intestinal development in preterm and term infants using RNA-Sequencing. *Sci Rep* **4**, 5453 (2014).
65. Eggermont, A.M., *et al.* Prolonged Survival in Stage III Melanoma with Ipilimumab Adjuvant Therapy. *N Engl J Med* **375**, 1845-1855 (2016).
66. Bertrand, A., Kostine, M., Barnetche, T., Truchetet, M.E. & Schaeverbeke, T. Immune related adverse events associated with anti-CTLA-4 antibodies: systematic review and meta-analysis. *BMC Med* **13**, 211 (2015).
67. Maughan, B.L., Bailey, E., Gill, D.M. & Agarwal, N. Incidence of Immune-Related Adverse Events with Program Death Receptor-1- and Program Death Receptor-1 Ligand-Directed Therapies in Genitourinary Cancers. *Front Oncol* **7**, 56 (2017).
68. Topalian, S.L., *et al.* Safety, activity, and immune correlates of anti-PD-1 antibody in cancer. *N Engl J Med* **366**, 2443-2454 (2012).
69. Villadolid, J. & Amin, A. Immune checkpoint inhibitors in clinical practice: update on management of immune-related toxicities. *Transl Lung Cancer Res* **4**, 560-575 (2015).
70. Kumar, V., *et al.* Current Diagnosis and Management of Immune Related Adverse Events (irAEs) Induced by Immune Checkpoint Inhibitor Therapy. *Front Pharmacol* **8**, 49 (2017).
71. Ascierto, P.A., *et al.* Ipilimumab 10 mg/kg versus ipilimumab 3 mg/kg in patients with unresectable or metastatic melanoma: a randomised, double-blind, multicentre, phase 3 trial. *Lancet Oncol* **18**, 611-622 (2017).
72. Collins, L.K., Chapman, M.S., Carter, J.B. & Samie, F.H. Cutaneous adverse effects of the immune checkpoint inhibitors. *Curr Probl Cancer* **41**, 125-128 (2017).
73. Lin, Z., *et al.* PD-1 Antibody Monotherapy for Malignant Melanoma: A Systematic Review and Meta-Analysis. *PLoS One* **11**, e0160485 (2016).
74. Tarhini, A.A., *et al.* Phase III Study of Adjuvant Ipilimumab (3 or 10 mg/kg) Versus High-Dose Interferon Alfa-2b for Resected High-Risk Melanoma: North American Intergroup E1609. *J Clin Oncol* **38**, 567-575 (2020).

75. Burla, J., *et al.* Retrospective Analysis of Treatment and Complications of Immune Checkpoint Inhibitor-Associated Colitis: Histological Ulcerations as Potential Predictor for a Steroid-Refractory Disease Course. *Inflamm Intest Dis* **5**, 109-116 (2020).
76. Geukes Foppen, M.H., *et al.* Immune checkpoint inhibition-related colitis: symptoms, endoscopic features, histology and response to management. *ESMO Open* **3**, e000278 (2018).
77. Suzman, D.L., Pelosof, L., Rosenberg, A. & Avigan, M.I. Hepatotoxicity of immune checkpoint inhibitors: An evolving picture of risk associated with a vital class of immunotherapy agents. *Liver Int* **38**, 976-987 (2018).
78. Mooradian, M.J., *et al.* Musculoskeletal rheumatic complications of immune checkpoint inhibitor therapy: A single center experience. *Semin Arthritis Rheum* **48**, 1127-1132 (2019).
79. Das, S., *et al.* Immune-Related Adverse Events and Immune Checkpoint Inhibitor Efficacy in Patients with Gastrointestinal Cancer with Food and Drug Administration-Approved Indications for Immunotherapy. *Oncologist* **25**, 669-679 (2020).
80. Matsuoka, H., *et al.* Correlation between immune-related adverse events and prognosis in patients with various cancers treated with anti PD-1 antibody. *BMC Cancer* **20**, 656 (2020).
81. Suo, A., *et al.* Anti-PD1-Induced Immune-Related Adverse Events and Survival Outcomes in Advanced Melanoma. *Oncologist* **25**, 438-446 (2020).
82. Dubin, K., *et al.* Intestinal microbiome analyses identify melanoma patients at risk for checkpoint-blockade-induced colitis. *Nat Commun* **7**, 10391 (2016).
83. Luoma, A.M., *et al.* Molecular Pathways of Colon Inflammation Induced by Cancer Immunotherapy. *Cell* **182**, 655-671 e622 (2020).
84. Seo, S.U., *et al.* Distinct Commensals Induce Interleukin-1beta via NLRP3 Inflammasome in Inflammatory Monocytes to Promote Intestinal Inflammation in Response to Injury. *Immunity* **42**, 744-755 (2015).
85. Andrews, M.C., *et al.* Gut microbiota signatures are associated with toxicity to combined CTLA-4 and PD-1 blockade. *Nat Med* (2021).
86. Li, B., Dewey, C.N. & C., B.M. RSEM: accurate transcript quantification from RNA-Seq data with or without a reference genome. *Aug 4323 PMCID PMC3163565* **12 SRC - GoogleScholar**(2011).
87. Qiu, J., *et al.* The aryl hydrocarbon receptor regulates gut immunity through modulation of innate lymphoid cells. *Immunity* **36**, 92-104 (2012).
88. Kim, M.H., Kang, S.G., Park, J.H., Yanagisawa, M. & Kim, C.H. Short-chain fatty acids activate GPR41 and GPR43 on intestinal epithelial cells to promote inflammatory responses in mice. *Gastroenterology* **145**, 396-406 e391-310 (2013).
89. Park, J., *et al.* Short-chain fatty acids induce both effector and regulatory T cells by suppression of histone deacetylases and regulation of the mTOR-S6K pathway. *Mucosal Immunol* **8**, 80-93 (2015).
90. Singh, Y., *et al.* Differential effect of DJ-1/PARK7 on development of natural and induced regulatory T cells. *Sci Rep* **5**, 17723 (2015).
91. Macia, L., *et al.* Metabolite-sensing receptors GPR43 and GPR109A facilitate dietary fibre-induced gut homeostasis through regulation of the inflammasome. *Nat Commun* **6**, 6734 (2015).
