# Supplementary material for: Sucralose Consumption Ablates Cancer Immunotherapy Response through Microbiome Disruption
Source: Cancer Discov. 2025 Jul 30;15(11):2278–97. doi: 10.1158/2159-8290.CD-25-0247 (PMC12580791; doi:10.1158/2159-8290.CD-25-0247)
Supplement: Appendix 3 — shows the IRB approval for HCC 17 to 169. [file cd-25-0247_appendix_3_suppsa3.pdf]

Appendix 3. IRB approval for HCC 17-169 protocol.

University of Pittsburgh | Institutional Review Board
Office of Research Protections

MODIFICATION APPROVAL (Expedited)

Date:	September 6, 2024
IRB:	MOD19040237-030
PI:	Diwakar Davar, MD
Title:	Neoadjuvant Phase II Study of TLR9 Agonist CMP-001 in Combination with Nivolumab in Stage IIIB/C/D Melanoma Patients with Clinically Apparent Lymph Node Disease
Funding:	Name: Melanoma Research Foundation; Name: Checkmate Pharm; Name: National Institute of Health, Grant Office ID: 13065495

The Institutional Review Board reviewed and approved the above referenced modification, and the study may continue as outlined in the University of Pittsburgh approved application and documents.

Approval Documentation

Review type:	Modification / Update
Approval Date:	9/6/2024
Expiration Date:	9/2/2025
Expedited Category:	(mm) Minor modification – study team update

As the Principal Investigator, you are responsible for the conduct of the research and to ensure accurate documentation, protocol compliance, reporting of possibly study-related adverse events and unanticipated problems involving risk to participants or others. The HRP Reportable Events policy, Chapter 17, is available at http://www.hrpo.pitt.edu/.

If you have any questions, please contact the University of Pittsburgh IRB Coordinator, Juliet Mancino.

Please take a moment to complete our Satisfaction Survey as we appreciate your feedback.

The University of Pittsburgh has a Federal Wide Assurance approved through the Office of Human Research Protections (FWA00006790).
