# Supplementary material for: Sucralose Consumption Ablates Cancer Immunotherapy Response through Microbiome Disruption
Source: Cancer Discov. 2025 Jul 30;15(11):2278–97. doi: 10.1158/2159-8290.CD-25-0247 (PMC12580791; doi:10.1158/2159-8290.CD-25-0247)
Supplement: Appendix 4 — shows the full protocol for HCC 17 to 169. [file cd-25-0247_appendix_4_suppsa4.pdf]

Appendix 4. Full protocol for HCC 17-169.

TITLE: Neoadjuvant Phase II Study of TLR9 Agonist CMP-001 in Combination with Nivolumab in Stage IIIB/C/D Melanoma Patients with Clinically Apparent Lymph Node Disease

**SPONSOR:**	**Diwakar Davar, MD**
**IND NUMBER:**	**18193**
**PI:**	**Diwakar Davar, MD**
**Source of Support:**	**Checkmate Pharmaceuticals**<br>**NIH – UPCI SPORE, RO1 CA257265**<br>**Melanoma Research Foundation**
**Version:**	**11 (08/18/2022)**

SUMMARY OF CHANGES

PROTOCOL

- **First Page, Protocol version and date change from v10 01/26/2022 to v11 08/18/2022**
- **First Page, added Funding sources**

Table of Contents

1. Trial Summary
2. Trial Design
    2.1.1. Trial Design
    2.1.2. Trial Diagram
3. Objective(s) and Hypothesis(es)
    3.1. Primary Objective and Hypothesis
    3.2. Secondary Objectives
4. Background and Rationale
    4.1. Background
        4.1.1. Pharmaceutical and Therapeutic Background
        4.1.2. Preclinical and Clinical Trial Data
    4.2. Rationale
        4.2.1. Rationale for Dose Selection/Regimen/Modification
5. Methodology
    5.1. Entry Criteria
        5.1.1. Diagnosis/Conditions for Study Entry
        5.1.2. Subject Inclusion Criteria
        5.1.3. Subject Exclusion Criteria
    5.2. Trial Treatments
        5.2.1. Nivolumab Dose Selection
        5.2.2. CMP-001 Dose Selection
    5.3. Treatment Allocation
    5.4. Stratification
    5.5. Concomitant Medications
    5.6. Rescue Medications and Supportive Care
        5.6.1. Supportive Care Guidelines
    5.7. Subject Withdrawal/Discontinuation Criteria
    5.8. Subject Replacement Strategy
    5.9. Clinical Criteria for Early Trial Termination
    5.10. Surgical Considerations
        5.10.1. Primary excision or primary cutaneous melanoma
        5.10.2. Regional lymphadenectomy
        5.10.3. Lymphadenectomy for nodal recurrence
6. Trial Flow Chart
        6.1.1. Study Flow Chart
7. Trial Procedures
    7.1. Trial Procedures
        7.1.1. Administrative Procedures
        7.1.2. Clinical Procedures/Assessments
        7.1.3. Laboratory Procedures/Assessments
        7.1.4. Other Procedures
        7.1.5. Visit Requirements
    7.2. Adverse and Serious Adverse Events
        7.2.1. Definitions
        7.2.2. Evaluation of Adverse Events and Serious Adverse Events
        7.2.3. Timeframe for Adverse Event/Serious Adverse Event Collection

7.2.4. Recording Adverse Events/Serious Adverse Events
7.2.5. Reporting Serious Adverse Events and Serious Unexpected Adverse Events
7.2.6. IND Safety Report
7.2.7. Follow-up of Adverse Events/Serious Adverse Events
8. Statistical Analysis Plan
8.1. Study Design
8.2. Safety Monitoring
8.3. Sample Size
8.4. Efficacy Analysis
8.5. Safety Analysis
8.6. Biomarker Analysis
8.7. Data Safety and Monitoring Plan
9. Labeling, Packaging, Storage and Return of Clinical Supplies
9.1. Investigational Product
9.2. Packaging and Labeling Information
9.3. Handling, Storage and Accountability
9.4. Dispensing
10. List of References

Appendix 1 Dietary History Questionnaire for Microbiome Sampling

Appendix 2 Pathologic Response Assessment (irPRC Criteria)

1 Trial Summary

Trial Phase	Non-randomized phase II single arm Simon two-stage
Clinical Indication	PD-1 naïve stage IIIB/C/D melanoma
Trial Type	Neoadjuvant study
Type of control	Not applicable
Route of administration	CMP-001 pre-operatively (intra-tumoral) and post-operatively (subcutaneous)<br>Nivolumab pre- and post- operatively (intravenous)
Trial Blinding	N/A
Treatment Groups	N/A
Number of trial subjects	Total sample size: 33 (1st stage 14; 2nd stage 16; 3 additional allowing for dropout)
Estimated enrollment period	15-20 months
Estimated duration of trial	24 months
Duration of Participation	24 months
Estimated average length of treatment per patient	52 weeks

2. Trial Design

2.1 Trial Design

This is a phase II single arm Simon two-stage single-center study of nivolumab in combination with Toll-like receptor 9 (TLR9) agonist CMP-001 in patients with PD-1 naïve stage IIIB-IIID cutaneous (or unknown primary) melanoma with clinically apparent lymph node (LN) and/or in-transit and/or satellite disease. The study will be conducted over a 52-week period.

Patients with stage IIIB-IIID cutaneous (or unknown primary) melanoma with palpable nodal disease and/or in-transit disease who have yet to undergo definitive surgery are eligible to enroll. Patients with nodal and/or in-transit relapse including those who have received prior adjuvant IFN and/or ipilimumab are eligible to enroll. However, patients who have received either nivolumab or pembrolizumab or ipilimumab/nivolumab are **NOT** eligible. Other eligibility criteria include, but are not limited to, absence of CNS disease and presence of disease amenable to biopsy.

Suitable patients will be identified pre-operatively. Patients will undergo a rapid (preferred 21 days, up to 28 days) screening evaluation consisting of systemic/CNS staging scans, tumor biopsy, and correlative blood studies to confirm suitability. Eligible patients will receive nivolumab/CMP-001 combination (prime phase) peri-operatively for a total of 7 weeks. In the peri-operative period, CMP-001 will be administered weekly via sub-cutaneous injection (week 1) then intra-tumorally (weeks 2-7). Following peri-operative therapy and restaging systemic scans, patients will undergo surgical resection. Post-operatively, patients will continue to receive nivolumab/CMP-001 combination (boost phase) every 2 (or 4) weeks (nivolumab) and 4 weeks (CMP-001) to complete 1 year (52 weeks) of therapy. In the post-operative period, CMP-001 will be administered subcutaneously.

2.2 Trial Diagram

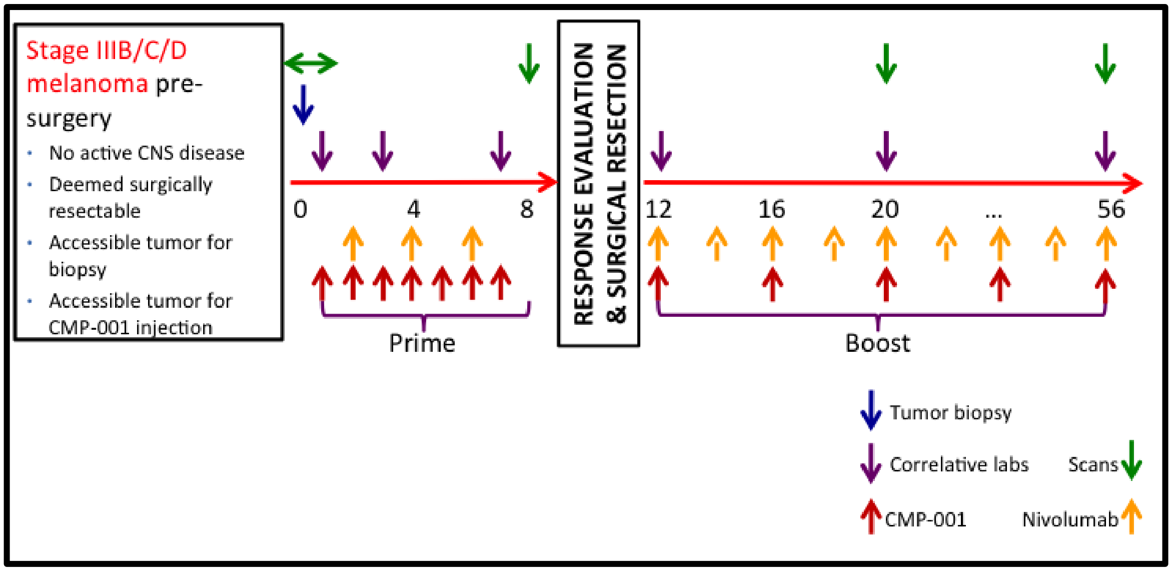

Figure 1: Trial Diagram

3. Objective(s) and Hypothesis(es)

3.1 Primary Objective and Hypothesis

Objective: To evaluate major pathologic response rate (MPR rate) in patients with stage IIIB/C melanoma following 7 weeks of nivolumab and injected CMP-001.

Hypothesis: That the combination of nivolumab and injected CMP-001 improves MPR rate in patients with stage IIIB/C melanoma.

3.2 Secondary Objectives

Objective: To evaluate safety, relapse-free survival (RFS) and overall survival (OS) in patients with stage IIIB/C melanoma following 52 weeks of nivolumab/CMP-001 combination.

Hypothesis: Nivolumab/CMP-001 combination is safe and improves RFS and OS in patients with stage IIIB/C/D melanoma.

3.3 Exploratory Objectives

Objective: To evaluate circulating and intra-tumoral immune cells, including T-cells (CD8, CD4, Tregs) and antigen-presenting cells (monocytes, macrophages, MDSCs), for the expression of inhibitory and activating receptors and ligands including functional analyses. To evaluate TCR clonality/diversity analyses of circulating and intra-tumoral CD8+ T-cells before and after nivolumab and CMP-001 combination. To evaluate genetic and transcriptomic signatures of response/non-response. To evaluate novel imaging characteristics in responders and non-responders.

Hypothesis: Nivolumab/CMP-001 combination improves T-cell functionality by augmenting antigen presentation and increasing T-cell infiltrate. Nivolumab/CMP-001 combination improves T-cell infiltrate by genetic, transcriptomic and imaging analyses.

4. Background and Rationale

4.1 Background

4.1.1 Pharmaceutical and Therapeutic Background: Nivolumab

The importance of intact immune surveillance in controlling outgrowth of neoplastic transformation has been known for decades (Disis 2010). The inability of the immune system to control tumor growth does not appear to result from an inability to recognize the tumor as foreign. Tumor cells have been shown to evade immune destruction despite displaying recognizable antigens on their surface and despite the presence of high-avidity T cells that are specific for these antigens (Boon and van der Bruggen 1996; Boon and Old 1997; Boon et al. 2006; Ercolini et al. 2005). Histologic evaluation of many human cancers show extensive infiltration by inflammatory and immune cells (Galon et al. 2006; Galon et al. 2012), suggesting that the immune system responds less effectively to malignancy. These observations have led to the hypothesis that dominant mechanisms of immune tolerance or immune suppression are responsible for the immune system's inability to effectively respond in a way that consistently results in rejection.

There are a number of inhibitory mechanisms that have been identified to be involved in tumor-mediated immune suppression and include expression of the programmed death ligand-1 (PD-L1), which can engage the inhibitory receptor PD-1 on activated T cells; the presence of the tryptophan-catabolizing enzyme IDO1, which exploits the exquisite sensitivity of T cells to tryptophan depletion and tryptophan metabolites; and infiltration with FoxP3+ regulatory T cells (Treg), which can mediate extrinsic suppression of effector T-cell function. Therefore, agents that target these negative regulatory pathways and thereby allow the expansion of effector T cells present in the tumor may be beneficial in the clinic.

The PD-1 receptor-ligand interaction is a major pathway stimulated by tumors to suppress immune control. The normal function of PD-1, expressed on the cell surface of activated T cells under healthy conditions, is to down-modulate unwanted or excessive immune responses, including autoimmune reactions. Programmed death receptor-1 (encoded by the gene Pdcd1) is an Ig superfamily member related to CD28 and cytotoxic T-lymphocyte-associated antigen-4 (CTLA-4), which has been shown to negatively regulate antigen receptor signaling upon engagement of its ligands (PD-L1 and/or PD-L2) (Freeman et al. 2000; Latchman et al. 2001; Liang et al. 2003). The mechanism by which PD-1 down modulates T-cell responses is similar to but distinct from that of CTLA-4, as both molecules regulate an overlapping set of signaling proteins (Parry et al. 2005; Hiraoka 2010).

Programmed death receptor-1 has been shown to be expressed on activated lymphocytes including peripheral CD4+ and CD8+ T cells, B cells, Tregs, and natural killer cells (Hodi et al. 2008). Expression has also been shown during thymic development on CD4-CD8- (double negative) T cells as well as subsets of macrophages and dendritic cells (DCs). The ligands for PD-1 (PD-L1 and PD-L2) are constitutively expressed or can be induced in a variety of cell types, including non-hematopoietic tissues as well as in various tumors (Latchman et al. 2001; Loke and Allison 2003; Liang et al. 2003; Francisco et al. 2009; Taube et al. 2014). Binding of either PD-1 ligand to PD-1 inhibits T-cell activation triggered through the T-cell receptor. PD-L1 is expressed at low levels on various non-hematopoietic tissues, most notably on vascular endothelium, whereas PD-L2 protein is only detectably expressed on antigen-presenting cells found in lymphoid tissue or chronic inflammatory environments. Programmed death ligand-2 is thought to control immune T-cell activation in lymphoid organs, whereas PD-L1 serves to dampen unwarranted T-cell function in peripheral tissues. Although healthy organs express little (if any) PD-L1, a variety of cancers were demonstrated to express abundant levels of this T-cell inhibitor. Programmed death receptor-1 has

been suggested to regulate tumor-specific T-cell expansion in subjects with melanoma (Liotta et al 2010). This suggests that the PD-1/PD-L1 pathway plays a critical role in tumor immune evasion and should be considered as an attractive target for therapeutic intervention.

Nivolumab is a fully human Ig G4 antibody that blocks PD-1. Nivolumab was initially approved by the FDA on December 22, 2014 for the treatment of patients with unresectable or metastatic melanoma and disease progression following ipilimumab and, if BRAF V600 mutation positive, a BRAF inhibitor. Nivolumab was also approved on March 4, 2015 to treat patients with advanced (metastatic) squamous non–small cell lung cancer (NSCLC) with progression on or after platinum-based chemotherapy. Additionally, FDA approved nivolumab on November 23, 2015 for advanced renal cell carcinoma. In Europe, it was approved on June 19, 2015 as a single agent (monotherapy) for the treatment of advanced (unresectable or metastatic) melanoma in adults. Another extension of indication was approved on October 28, 2015 to treat the advanced stages of squamous NSCLC after prior chemotherapy in adults. On February 25, 2016, it was also approved for advanced renal cell carcinoma after previous therapy in adults.

More recently, nivolumab was approved by the FDA (https://www.fda.gov/Drugs/InformationOnDrugs/ApprovedDrugs/ucm590004.htm) for the adjuvant treatment of resected stage IIIB-IVC melanoma patients on the basis of the data from the CheckMate-238 study (Weber et al. 2017). In this study, compared to ipilimumab, adjuvant therapy with nivolumab for 1 year resulted in significantly longer recurrence-free survival and overall survival. Compared to ipilimumab adjuvant therapy, nivolumab adjuvant therapy had a lower rate of grade 3 or 4 adverse events.

4.1.2 Pharmaceutical and Therapeutic Background: CMP-001

CMP-001 is a molecule comprised of a 30 nucleotide strand, flanked by 10 guanines on either end. The nucleotide strand is surrounded by a Qβ viral-like protein. The intended mechanism of action of CMP-001 in oncology is the activation of TLR9 in pDC within the tumor or the tumor-draining lymph nodes (tumor-associated pDC). Tumor-associated pDC activated by CMP-001 are expected to release large amounts of type I interferons (IFN), and increase their expression of costimulatory molecules and tumor antigen presentation to T cells, culminating in the generation of a clinically effective anti-tumor T cell response. In order for this response to be specific for the tumor, the pDC should have taken up the tumor antigens already, which requires them to be activated either within the tumor, or in the draining lymph nodes, which in turn requires the subcutaneous route of administration to reach a locally effective concentration of CMP-001. Systemic administration of TLR9 agonists results primarily in liver, spleen, and RES uptake with little specific activation of pDC in peripheral lymph nodes. Immature tumor-associated pDC contribute to tumor growth and an adverse prognosis in patients with cancer (Lombardi et al. 2015; Demoulin et al. 2013). Activating and maturing these pDC through either subcutaneous or intratumoral administration of CMP-001 is expected to reverse the pDC functional effects from promoting to antagonizing tumor growth, and from supporting immune tolerance to inducing an anti-tumor CD8+ T cell response in the tumor microenvironment.

CMP-001 is composed of (i) a virus-like particle (VLP) comprised of capsid proteins derived from bacteriophage Qbeta, which encapsulate (ii) a cytosine linked to a guanine by a phosphate bond (CpG) oligodeoxynucleotide (ODN) known as G10, which is a Toll-like receptor 9 (TLR9) agonist designed to induce high levels of type I interferon production and an anti-tumor CD8+ T cell response through activation of TLR9 in plasmacytoid dendritic cells (pDC). The therapeutic agent is a VLP referred to as QbG10. QbG10 has been previously studied in clinical trials under the name CYT003. In this protocol the name CYT003 will refer to historical data using the product in non-oncology settings. The name CMP-001 will refer to Checkmate Pharmaceuticals' plans for the agent.

4.1.3 Preclinical and Clinical Trial Data: Nivolumab

Refer to the Investigator's Brochure for Preclinical and Clinical data.

4.1.4 Preclinical and Clinical Trial Data: CMP-001

Preliminary Safety and Efficacy in Asthma

QbG10, currently known as CMP-001, has been previously studied in clinical trials under the name CYT003. CYT003 has been administered to 732 humans (volunteers or patients with non-cancer diagnoses) either alone or in combination with various allergens. CYT003 has been administered at doses as high as 2 mg weekly for seven doses by SC administration. CYT003 has reproducibly demonstrated evidence of TLR9 agonist activity with intended immune stimulatory effects. It has generally been well tolerated, with mild to moderate flu-like symptoms and/or injection site reactions noted in the majority of treated patients.

Preliminary Safety and Efficacy in PD-1 primary Refractory Melanoma

Intratumoral administration of CMP-001 is currently being evaluated in combination with pembrolizumab in subjects with metastatic melanoma resistant to checkpoint inhibitors. In study CMP-001-001, A Multicenter, Open-label Phase 1b clinical Study of CMP-001 in Combination with Pembrolizumab in Subjects with Advanced Melanoma, CMP-001 is being administered intratumorally on a weekly (Schedule A) and Q3W schedule (Schedule B) across a dose range of 1-10 mg. To date, approximately 45 subjects have been treated in the CMP-001-001 study. Preliminary analysis of safety demonstrates an acute toxicity profile that predominantly consists of Grade 1-2 adverse events including mild fever, chills, rigors, nausea, vomiting and occasional hypotension. In some subjects the severity of this adverse event profile is increased. These AEs typically present after the third CMP-001 dose and generally resolve within a few hours with standard supportive care. The mechanism of action associated with the acute toxicity profile appears to resemble symptoms associated with cytokine release. A treatment algorithm has been developed for the management of these reactions and can be found in **Section 5.2.2.7**. CMP-001-001 adverse event summary in Cohort 2 of the Dose Escalation Phase are presented in **Table 4.1.4-1**.

Table 4.1.4-1: Related AEs Reported in More than One Subject in CMP-001-001 Study

CMP001-001 Protocol<br><br>Related AEs Reported in More than One Subject*		
	N (%) of Subjects	
	Schedule A^<br><br>(N=28)	Schedule B^^<br><br>(n=11)
Anemia	4 (14.3%)	-
Chills	15 (53.6%)	-
Constipation	2 (7.1%)	-
Cough	2 (7.1%)	-
Diarrhea	3 (10.7%)	-

Fatigue	7 (25.0%)	2 (18.2%)
Fever	16 (57.1%)	-
Flushing	2 (7.1%)	-
Headache	6 (21.4%)	2 (18.2%)
Hypertension	2 (7.1%)	-
Hypotension	8 (28.6%)	-
Injection Site Pain	2 (7.1%)	3 (27.3%)
Nausea	8 (28.6%)	-
Pruritus	3 (10.7%)	-
Rigors	6 (21.4%)	-
Tachycardia	2 (7.1%)	-
Vomiting	8 (28.6%)	2 (18.2%)
*Based on EDC data as of 30 May 2017<br>^ Schedule A includes Cohort 1 (1 mg, n=3); Cohort 2 (3 mg, n=12); Cohort 4 (5 mg, n=6); Cohort 6 (10 mg, n=3); Cohort 8 (10 mg, n=4)<br>^^ Schedule B includes Cohort 3 (3 mg, n=4); Cohort 5 (5 mg, n=3); Cohort 7 (10 mg, n=3), Cohort 9 (10 mg, n=1)		

Preliminary efficacy data, based upon RECIST investigator reads, from the ITT population across both schedules from the 1, 3 and 5 mg cohorts (N=28) demonstrated on ORR of 21.4% (5 PRs, 1 CR). The ORR in subjects treated on Schedule A (N=21) was 24% (5/21; 95% CI 8.2%-47.2%) and on Schedule B (N=7) was 14.3% (1/7; 95 % CI 0.4-57.9%). All responses seen were evidenced at the subject's first follow up scan (12 weeks). Durability or all responses have been seen beyond 24 weeks with some responses being maintained beyond 1 year.

4.2 Rationale

4.2.1 Rationale for Dose Selection/Regimen/Modification: Nivolumab

Refer to the **eIB** and the nivolumab **SmPC** or **USPI** for preclinical and clinical study data.

Nivolumab at a dose of 3 mg/kg has been approved as monotherapy in melanoma and NSCLC patients. In the current study, nivolumab will be administered at 240 mg Q2W during the Prime Phase. During the Boost Phase, nivolumab will be administered at either 240 mg Q2W or 480mg Q4W depending on physician discretion. The choice of the 240 mg Q2W (or 480mg Q4W) as an appropriate dose for the switch to fixed dosing is based on simulations performed using the population PK model of nivolumab showing that the fixed dose of 240 mg every 2 weeks (and 480mg Q4W) will provide exposures that 1) are optimally consistent with those obtained with the 2 mg/kg dose every 2 weeks, 2) will maintain individual patient exposures in the exposure range established in melanoma as associated with maximal efficacy response and 3) will maintain individual patients exposure in the exposure range established in melanoma that are well tolerated and safe (Freshwater et al. 2017; Long et al. 2018).

A fixed dose regimen will simplify the dosing regimen to be more convenient for physicians and to reduce potential for dosing errors. A fixed dosing scheme will also reduce complexity in the logistical chain at treatment facilities and reduce wastage.

4.2.2 Rationale for Dose Selection/Regimen/Modification: CMP-001

During the peri-operative period (Prime Phase), CMP-001 5mg will be administered subcutaneously for the first dose (week 1). Thereafter, CMP-001 will be administered intra-tumorally at 10mg weekly (weeks 2-7).

During the post-surgical period (Boost Phase), CMP-001 5mg weekly will be administered subcutaneously every 4 weeks for up to 54 weeks.

Upon initial exposure to CMP-001, antibodies against the Q-beta capsid protein are formed. Thereafter the FC portion of the antibody bind to the FCR-γ on the pDC. These antibodies are necessary to facilitate the binding of the VLP to the FC. After the first dose approximately 100% of the subjects exposed to CMP-001 demonstrate the presence of these antibodies. Therefore, to maximize the therapeutic potential of CMP-001 and minimize the number of intratumoral injections, the first dose of CMP-001 will be administered subcutaneously.

The rationale for the weekly schedule is primarily based upon the safety and efficacy results from the ongoing study in metastatic melanoma noted above. A comparison of the weekly and Q3weekly schedule in that study demonstrated a trend towards improved efficacy in the weekly schedule. During the initial safety run-in of this study, a variety of doses were explored based on tumor size. The rationale for the fixed dose during Prime Phase weeks 2-7 is based on the safety and efficacy results from the initial safety run-in that showed no increased incidence of adverse events in patients treated with higher doses of CMP-001.

4.2.3 Rationale for Combining TLR9 Agonist with an Anti PD-1/PD-L1 Antibody

Toll-like receptors (TLRs) are part of the innate immune system where they recognize and bind conserved pathogen-associated molecular patterns (PAMPs) (Murad and Clay 2009). TLR9 recognizes unmethylated cytosine guanosine dinucleotides (CpG) oligodinucleotides (ODN); and TLR9 agonism activates plasmacytoid dendritic cells (pDC) and B cells, and results in potent T helper-1 (Th-1)-type immune and anti-tumor responses in mouse tumor models and in patients with human cancer (Fourcade et al. 2008). However, while CpG administration strongly induces tumor-specific CD8+ T cell responses, objective responses are rare and T cell responses are not sustained (Fourcade et al. 2008; Appay, Speiser, et al. 2006; Appay, Jandus, et al. 2006). There are 3 classes of CpG ODN: type A which strongly stimulate pDC IFN-α secretion and minimal B cell activation; type B which strongly stimulate B cell proliferation/differentiation and NF-κβ activation; and type C which stimulate B cell proliferation/differentiation and moderately stimulate pDC IFN-α secretion (Krieg 2007). Pre-clinically, intra-tumoral CpG synergizes with anti-PD-1 therapy by promoting infiltration of activated TA-specific CD8+ T cells expressing IFN-γ and TNF-α (Wang et al. 2016). In patients with advanced melanoma, the combination of type C TLR9 agonist SD-101 and PD-1 inhibitor pembrolizumab is associated with significant responses with minimal additional toxicity (Ribas et al. 2018).

5 Methodology

5.1 Entry Criteria

5.1.1 Diagnosis/Conditions for Study Entry

Patients eligible for this study should have locally and/or regionally advanced melanoma that is considered potentially surgically resectable and with biopsy-amemable tumor at baseline.

5.1.2 Subject Inclusion Criteria

A patient must meet all of the following criteria to be eligible to participate in this study:

1. Be willing and able to provide written informed consent for the study.
2. Be ≥ 18 years of age on day of signing informed consent.
3. Diagnosis of histologically or cytologically confirmed diagnosis of cutaneous melanoma belonging to one of the following AJCC TNM stages:
    a. T_x or T_{1-4} **and**
    b. N_{1b}, or N_{1c}, or N_{2b}, or N_{2c}, or N_{3b}, or N_{3c} **and**
    c. M_0

    Patients are eligible for this trial either at presentation for primary melanoma with concurrent regional nodal and/or in-transit metastasis; **or** at the time of clinical detected nodal and/or in-transit recurrence; **and may belong to any of the following groups:**
    - Primary cutaneous melanoma with clinically apparent regional lymph node metastases.
    - Clinically detected recurrent melanoma at the proximal regional lymph node(s) basin.
    - Clinically detected primary cutaneous melanoma involving multiple regional nodal groups.
    - Clinical detected nodal melanoma (if single site) arising from an unknown primary.
    - In-transit and/or satellite metastases with or without regional lymph node involved permitted if considered potentially surgically resectable at baseline.
    - **NOTE: Determination of potential resectability must be made at baseline to be eligible for this neoadjuvant study.**
    - **NOTE: Patients with mucosal and/or uveal melanoma are not permitted to enroll. Patients with melanomas of unknown primary may be enrolled at the discretion of the treating investigator in discussion with Principal Investigator.**

4. Presence of injectable and measureable disease based on RECIST 1.1.

5. Willing to undergo tumor biopsy (core, punch, incisional or excisional). Patients must undergo biopsy (core, punch) or open biopsy (incisional, excisional) within 4 weeks of registration on the study.

6. Performance status of 0 or 1 on the ECOG Performance Scale.

7. Demonstrate adequate organ function as defined in **Table 5.1.2-1.** performed on screening labs obtained within 4 weeks of registration.

Table 5.1.2-1: Adequate Organ Function Laboratory Values

System	Laboratory Value
**Hematological**	
Absolute neutrophil count (ANC)	≥1,500 /mcL
Hemoglobin	≥9 g/dL or ≥5.6 mmol/L
Platelets	≥100,000 / mcL
**Renal**	
Serum creatinine **OR**<br>Measured or calculateda creatinine clearance (GFR can also be used in place of creatinine or	≤1.5 X upper limit of normal (ULN) **OR**<br>≥60 mL/min for subject with creatinine levels > 1.5 X institutional ULN

CrCl)	
**Hepatic**	
Serum total bilirubin	≤ 1.5 X ULN **OR** Direct bilirubin ≤ ULN for subjects with total bilirubin levels > 1.5 ULN
AST (SGOT) and ALT (SGPT)	≤ 2.5 X ULN
**Coagulation**	
International Normalized Ratio (INR) or Prothrombin Time (PT)	≤1.5 X ULN unless subject is receiving anticoagulant therapy as long as PT or PTT is within therapeutic range of intended use of anticoagulants
Activated Partial Thromboplastin Time (aPTT)	≤1.5 X ULN unless subject is receiving anticoagulant therapy as long as PT or PTT is within therapeutic range of intended use of anticoagulants

8. Female subject of childbearing potential should have a negative urine or serum pregnancy within 72 hours prior to receiving the first dose of study medication. If the urine test is positive or cannot be confirmed as negative, a serum pregnancy test will be required.

9. Female subjects of childbearing potential should be willing to use 2 methods of birth control or be surgically sterile, or abstain from heterosexual activity for the course of the study through 26 weeks after the last dose of study medication (**Section 5.7.2**). Subjects of childbearing potential are those who have not been surgically sterilized or have not been free from menses for > 1 year.

10. Male subjects should agree to use an adequate method of contraception starting with the first dose of study therapy through 26 weeks after the last dose of study therapy.

Note: Abstinence is acceptable if this is the usual lifestyle and preferred contraception for the subject.

5.1.3 Subject Exclusion Criteria

The subject must be excluded from participating in the trial if the subject:

1. History of uveal or mucosal melanoma.

2. Is currently participating in or has participated in a study of an investigational agent or using an investigational device within 4 weeks of the first dose of treatment.

3. Has a diagnosis of immunodeficiency or is receiving systemic steroid therapy or any other form of immunosuppressive therapy within 7 days prior to the first dose of trial treatment.

4. Has had prior chemotherapy, targeted small molecule therapy, or radiation therapy within 2 weeks prior to study Day 1 or who has not recovered (i.e., ≤ Grade 1 or at baseline) from adverse events due to a previously administered agent.

    - Note: Subjects with ≤ Grade 2 neuropathy are an exception to this criterion and may qualify for the study.

    - Note: If subject received major surgery, they must have recovered adequately from the toxicity and/or complications from the intervention prior to starting therapy.

    - Note: Subjects with autoimmune disorders of Grade 4 while on prior immunotherapy will be excluded. Subjects who developed autoimmune disorders of Grade ≤ 3 may

enroll if the disorder has resolved to Grade ≤1 and the subject has been off systemic steroids at doses >10 mg/d for at least 2 weeks.

5. Active (i.e., symptomatic or growing) central nervous system (CNS) metastases.

6. Has a known additional malignancy that is progressing or requires active treatment. Exceptions include basal cell carcinoma of the skin, squamous cell carcinoma of the skin, or in situ cervical cancer that has undergone potentially curative therapy.

7. Has a systemic disease that requires systemic pharmacologic doses of corticosteroids greater than 10mg daily prednisone (or equivalent). Subjects who are currently receiving steroids at a dose of ≤10mg daily do not need to discontinue steroids prior to enrollment Subjects that require topical, ophthalmologic and inhalational steroids would not be excluded from the study. Subjects with hypothyroidism stable on hormone replacement or Sjogren's syndrome will not be excluded from the study. Subjects who require active immunosuppression (greater than steroid dose discussed above) for any reason are excluded.

8. Has evidence of interstitial lung disease or active, non-infectious pneumonitis.

9. Has an active infection requiring systemic therapy.

10. Has a history or current evidence of any condition, therapy, or laboratory abnormality that might confound the results of the trial, interfere with the subject's participation for the full duration of the trial, or is not in the best interest of the subject to participate, in the opinion of the treating investigator.

11. Has known psychiatric or substance abuse disorders that would interfere with cooperation with the requirements of the trial.

12. Is pregnant or breastfeeding, or expecting to conceive or father children within the projected duration of the trial, starting with the pre-screening or screening visit through 26 weeks after the last dose of trial treatment.

13. Has received prior therapy with an anti-PD-1, anti-PD-L1, anti-PD-L2, anti-CD137. Prior treatment with ipilimumab or interferon alfa is allowed. Patients with history of allergic or hypersensitivity reaction to interferon alfa or ipilimumab are also excluded.

14. Has a known history of Human Immunodeficiency Virus (HIV) (HIV 1/2 antibodies).

15. Has known active Hepatitis B (e.g., HBsAg reactive) or Hepatitis C (e.g., HCV RNA [qualitative] is detected. Patients with treated Hepatitis B/C with no evidence of active infection may be enrolled.

5.2 Trial Treatments

This is a neoadjuvant biotherapy study testing nivolumab with CMP-001 in patients with locally/regionally advanced/recurrent melanoma. Subjects will receive combination nivolumab/CMP-001 for 7 weeks (Prime Phase) prior to surgery. Surgery will be scheduled approximately 2 – 4 weeks after completion of Prime Phase depending on recovery. After recovery from surgery subjects will receive additional nivolumab/CMP-001 for approximately 46 additional weeks of study drug(s)

administration (Boost Phase). The treatment to be used in this trial is outlined below. Trial treatment should begin within 4 weeks of study registration.

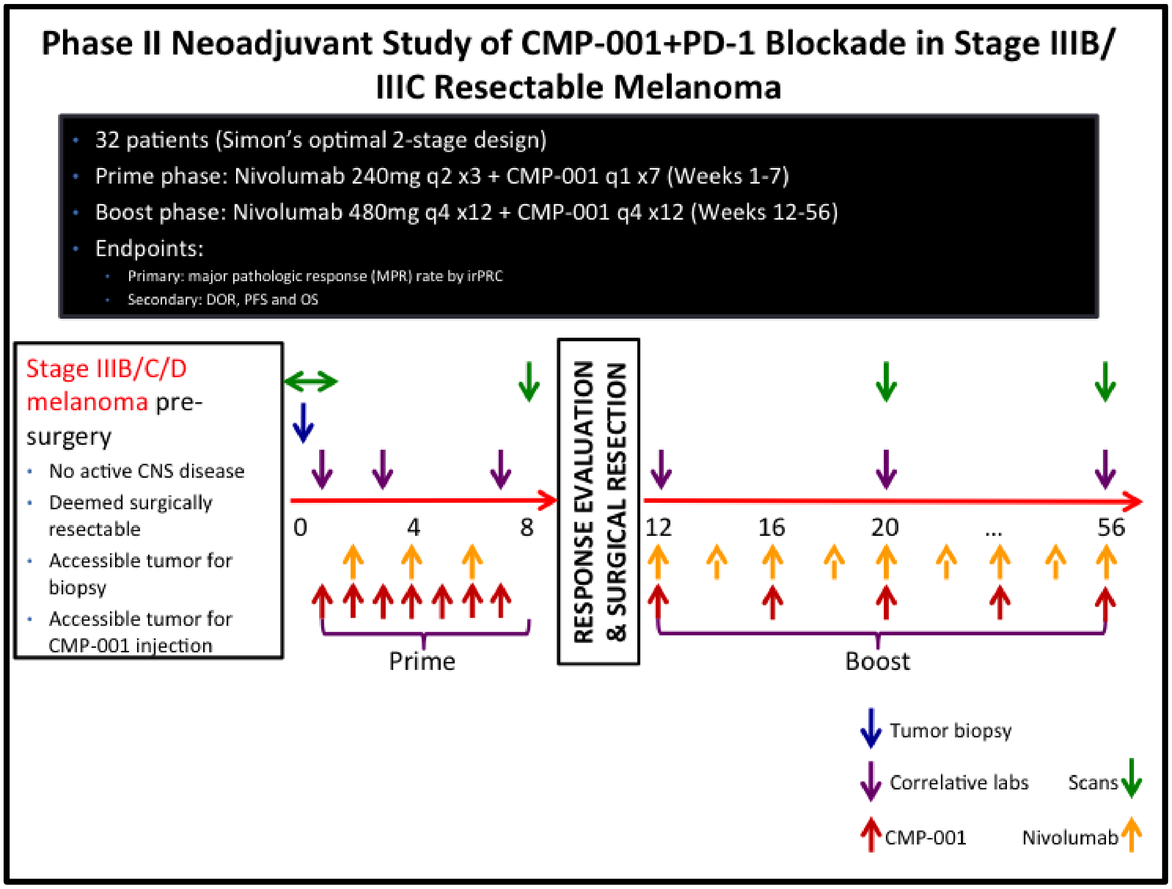

Figure 2: Trial Diagram

Allocation of study treatment arm: Up to 40 evaluable patients will be enrolled to receive nivolumab/CMP-001 combination.

5.2.1 Nivolumab Dose Selection/Modification

5.2.1.1 Nivolumab dose selection

Rationale is provided in **Section 4.2.1 – Rationale for Dose Selection/Regimen/Modification: Nivolumab**.

5.2.1.2 Nivolumab dose modification

Nivolumab will be withheld for drug-related Grade 4 hematologic toxicities, non-hematological toxicity ≥ Grade 3 including laboratory abnormalities, and severe or life-threatening AEs as per **Table 5.2.1.2-1** below.

Table 5.2.1.2-1: Dose Modification Guidelines for Drug-related Adverse Events.

Toxicity	Hold Treatment For Grade	Timing for Restarting Treatment	Treatment Discontinuation
Diarrhea/Colitis	2-3	Toxicity resolves to Grade 0-1	Toxicity does not resolve within 12 weeks of last dose or inability to reduce corticosteroid to 10 mg or less of prednisone or equivalent per day within 12 weeks
	4	Permanently discontinue	Permanently discontinue
AST, ALT, or Increased Bilirubin	2	Toxicity resolves to Grade 0-1	Toxicity does not resolve within 12 weeks of last dose
	3-4	Permanently discontinue (see exception below)[a]	Permanently discontinue
Type 1 diabetes mellitus (if new onset) or Hyperglycemia	T1DM or 3-4	Hold nivolumab and CMP-001 for new onset Type 1 diabetes mellitus or Grade 3-4 hyperglycemia associated with evidence of beta cell failure	Resume nivolumab when patients are clinically and metabolically stable
Hypophysitis	2-4	Toxicity resolves to Grade 0-1. Therapy with nivolumab and CMP-001 can be continued while endocrine replacement therapy is instituted	Toxicity does not resolve within 12 weeks of last dose or inability to reduce corticosteroid to 10 mg or less of prednisone or equivalent per day within 12 weeks
Hyperthyroidism	3	Toxicity resolves to Grade 0-1	Toxicity does not resolve within 12 weeks of last dose or inability to reduce corticosteroid to 10 mg or less of prednisone or equivalent per day within 12 weeks
	4	Permanently discontinue	Permanently discontinue
Hypothyroidism		Therapy with nivolumab and CMP-001 can be continued while thyroid replacement therapy is instituted	Therapy with nivolumab can be continued while thyroid replacement therapy is instituted
Infusion Reaction	2[b]	Toxicity resolves to Grade 0-1	Permanently discontinue if toxicity develops despite adequate premedication
	3-4	Permanently discontinue	Permanently discontinue
Pneumonitis	2	Toxicity resolves to Grade 0-1	Toxicity does not resolve within 12 weeks of last dose or inability to reduce corticosteroid to 10 mg or less of prednisone or equivalent per day within 12 weeks<br>Permanently discontinue for **recurrent** Grade 2 pneumonitis
	3-4	Permanently discontinue	Permanently discontinue
Renal Failure or Nephritis	2	Toxicity resolves to Grade 0-1	Toxicity does not resolve within 12 weeks of last dose or inability to reduce corticosteroid to 10 mg or less of prednisone or equivalent per day within 12 weeks
	3-4	Permanently discontinue	Permanently discontinue
All Other Drug-Related Toxicity[c]	3 or Severe	Toxicity resolves to Grade 0-1	Toxicity does not resolve within 12 weeks of last dose or inability to reduce corticosteroid to 10 mg or less of prednisone or equivalent per day within 12 weeks. Exceptions may be sought for neuropathy or other AE following discussion with Principle Investigator.
	4	Permanently discontinue	Permanently discontinue

Note: Permanently discontinue for any severe or Grade 3 drug-related AE that recurs or any life-threatening event.

[a] For patients with liver metastasis who begin treatment with Grade 2 AST or ALT, if AST or ALT increases by greater than or equal to 50% relative to baseline and lasts for at least 1 week then patients should be discontinued.

[b] If symptoms resolve within one hour of stopping drug infusion, the infusion may be restarted at 50% of the original infusion rate (e.g., from 100 mL/hr to 50 mL/hr). Otherwise dosing will be held until symptoms resolve and the subject should be premedicated for the next scheduled dose

[c] Patients with intolerable or persistent Grade 2 drug-related AE may hold study medication at physician discretion. Permanently discontinue study drug for persistent Grade 2 adverse reactions for which treatment with study drug has been held, that do not recover to Grade 0-1 within 12 weeks of the last dose.

In case toxicity does not resolve to Grade 0-1 within 12 weeks after last infusion, trial treatment should be discontinued after consultation with the Sponsor. With investigator and Sponsor agreement, subjects with a laboratory adverse event still at Grade 2 after 12 weeks may continue treatment in the trial only if asymptomatic and controlled. For information on the management of adverse events, see **Section 5.6.1**.

Subjects who experience a recurrence of the same severe or life-threatening event at the same grade or greater with re-challenge of nivolumab should be discontinued from trial treatment.

5.2.1.3 Nivolumab timing of dose administration

Trial treatment should be administered after all procedures/assessments have been completed as detailed on the Trial Flow Chart in **Section 6.0**. Trial treatment may be administered up to +/- 2 days before or after the scheduled Day 1 due to administrative reasons.

All trial treatments will be administered on an outpatient basis.

Nivolumab will be administered as a 30 minute IV infusion (treatment intervals may be increased due to toxicity as described). Sites should make every effort to target infusion timing to be as close to 30 minutes as possible. However, given the variability of infusion pumps from site to site, a window of -5 minutes and +10 minutes is permitted (i.e., infusion time is 30 minutes: -5 min/+10 min).

The Procedures Manual contains specific instructions for nivolumab dose calculation, reconstitution, preparation of the infusion fluid, and administration.

5.2.1.4 Nivolumab duration of administration

During the Prime Phase (Weeks 1-7), nivolumab 240mg will be administered every 2 weeks (W2D1, W4D1, W6D1). During this period, missed doses will not be made up.

During the Boost Phase (Weeks 12-56), nivolumab will be administered at either 240mg every 2 weeks or 480mg every 4 weeks. 12 doses (if Q4W) or 23 doses (if Q2W) of nivolumab will be administered over this period. During this period, missed doses will be made up. The choice of Q2W versus Q4W administration of nivolumab will be physician dependent.

5.2.2 CMP-001 Dose Selection/Modification

During the Prime Phase, the first dose of CMP-001 (week 1) is fixed at 5mg subcutaneously. Subsequent doses of CMP-001 administered during the Prime Phase (weeks 2-7) will be at 10mg administered intra-tumorally. Should patients develop side effects, intra-tumoral doses during this period of the Prime Phase (weeks 2-7) may be reduced (see **Table 5.2.2.3-1**).

Following resection, during the Boost Phase, the CMP-001 dose is fixed at 5mg injected subcutaneously at the site of the resected tumor.

Rationale for dose selection is provided in **Section 4.2.2 – Rationale for Dose Selection/Regimen/Modification: CMP-001**.

5.2.2.1 Recommended prophylaxis

To reduce the severity of symptoms associated with CMP-001 induced cytokines, prophylaxis is strongly recommended. Prophylactic regimens already in place at institutions should be followed, if not, below is a suggested regimen that has been effective for the treatment of CMP-001 induced adverse events:

- Fluids (e.g. 1000 cc IV normal saline)
- NSAIDS (e.g. 1000 mg acetaminophen and 50 mg Indomethacin)
- Anti-emetics (e.g.8 mg Zofran PO)
- Hydrocortisone prophylaxis (50-100mg hydrocortisone – only for patients with prior G2-3 CRS-like reaction)

If an Investigator incorporates prophylaxis, administration should occur 30 minutes prior to intra-tumoral injection of CMP-001. It is also highly recommended to continue to run additional fluids immediately following the CMP-001 injection, rather than waiting to initiate fluids when hypotension is detected. There is no waiting period between the end of prophylaxis and the start of CMP-001 intra-tumoral injection. All treatments given prophylactically pre-and post CMP-001 dosing should be recorded on the Prior and Concomitant Medications eCRF separately for each visit where it is given.

IMPORTANT: Subjects with a history of adrenal insufficiency are at increased risk for moderate to severe adverse events such as hypotension which may occur with 1-4 hours after injection. It is strongly recommended that these subjects receive stress dose steroids (eg. 50-100 mg hydrocortisone PO q 8 hours) prior to, or immediately after injection of CMP-001.

5.2.2.2 Timing of CMP-001 and nivolumab dosing

Initial study dose of nivolumab must be within 1 week of W1D1 (i.e., first CMP-001 injection). When CMP-001 and nivolumab dosing fall on the same day, it is recommended that CMP-001 dosing precede nivolumab dosing. There is no specified waiting period between the end of CMP-001 administration and the initiation of nivolumab infusion. When a study visit occurs where only nivolumab is given, pre-infusion vital signs should be collected and recorded on the eCRF.

5.2.2.3 Guidance of CMP-001 Injection

Intra-tumoral injections

Tumors selected for CMP-001 intra-tumoral injection should be at least 0.5cm in diameter and may be cutaneous, subcutaneous, and/or nodal tumors that are visible, palpable, or detectable by ultrasound guidance. Patients with visceral tumors are not candidates for this study. Patient candidates for CMP-001 injection may have more than one accessible tumor as a candidate for intra-tumoral injection.

- If 1 accessible tumor is present:
    - This should be selected as a candidate for intra-tumoral injection.
    - CMP-001 dose selection is outlined as in **Table 5.2.2.3-1**.
    - Total CMP-001 dose administered **should not exceed** 10mg/day.
- If >1 accessible tumors are present:
    - 1 or more accessible lesions can be injected (preferably all).
    - CMP-001 dose selection is outlined as in **Table 5.2.2.3-1**.
    - CMP-001 dose may be split at the discretion of the treating investigator (or designee).
    - Total CMP-001 dose administered across all tumors **should not exceed** 10mg/day.

Table 5.2.2.3-1: CMP-001 Injection Volume Guidelines Based on Tumor Size (Doses 2-6 in Prime Phase)

Dose Level	CMP-001 Dose (6mg/mL concentration)	Total Volume Injected
1	10mg	~2.0 mL
-1	7.5mg	~1.5 mL
-2	5mg	~1.0 mL
-3	2.5mg	~0.5 mL

Subcutaneous injections

CMP-001 should be injected using aseptic technique. Use of topical and/or local anesthetic is permitted. The injection may be given into any SC site in the body.

- Preferred Sites for Subcutaneous Injection
  - In principle, the injection may be given into any SC site in the body. Unsuitable sites for injection would include, for example, the palm of the hand or the sole of the foot.
  - The injection site should be within the area of lymphatic drainage associated with a site of metastatic disease or primary disease. For example, in a patient with a muscle or bone metastasis in the lower leg, the preferred SC injection site would be in the same leg, with the expectation that at least some of the CMP-001 will drain to lymph nodes that also contain tumor antigens. Likewise, in a patient with metastases in an upper lobe of the lung, a preferred SC injection site would be in the ipsilateral supraclavicular fossa, where the injection may activate pDC in the supraclavicular lymph nodes that also can drain the upper lung.
- Method of CMP-001 Subcutaneous Administration
  - Using standard aseptic technique, the needle is inserted into the subcutaneous tissue. After using gentle backward pressure on the syringe plunger to confirm extravascular location of the needle tip, the desired volume of CMP-001 is injected and the needle is withdrawn.

At Screening, the selected tumor(s) should be large enough to allow intratumoral injection of the intended volume of CMP-001 (**Table 5.2.2.3-1**). Initial administration of CMP-001 (W1D1) will be subcutaneous to allow for development of antibodies to the virus like particle which is necessary for uptake of CMP into the dendritic cell. Subsequent administrations of CMP-001 during the prime phase will be intra-tumoral. The same tumor(s) should be injected each week during therapy, if possible, except as outlined below to involuting tumors.

If the full volume cannot be injected within the tumor, then the remaining drug volume should be injected into a second accessible tumor, if present. If the full volume still cannot be administered into the tumor, peri-tumoral injection of any remaining volume is acceptable.

Following surgical resection, during the post-operative boost phase, CMP-001 will be administered subcutaneously at the resection site.

5.2.2.4 CMP-001 Dose Modification

CMP-001 will be dose-reduced in patients who develop CMP-001 related DLT and resume study therapy with Nivolumab/CMP-001 combination if Investigator determines that continued treatment with CMP-001 is in the subject’s best interest. In this event, CMP-001 will be dose reduced as in **Table 5.2.2.3-1**.

CMP-001 will be withheld for CMP-001 related Grade 4 hematologic toxicities, non-hematological toxicity ≥ Grade 3 including laboratory abnormalities, and severe or life-threatening AEs.

If CMP-001 is permanently discontinued for, subjects may continue to receive Nivolumab.

5.2.2.5 Method of CMP-001 Administration

Topical or local anesthesia may be given as appropriate in the Investigators’ judgment. Using standard aseptic technique, the needle is inserted near the tumor periphery and is advanced into the tumor to the desired depth (usually to the needle hub, if the tumor size permits) while maintaining

gentle backward pressure on the syringe plunger to confirm extravascular location of the needle tip. The syringe and needle are then slowly withdrawn to within a few millimeters of the skin or tumor surface while maintaining gentle downward pressure on the plunger to inject the desired volume of CMP-001 along the needle track (**Figure 3, middle panel**). With the tip of the needle still within the skin, the syringe is then rotated by ~20-40° and the process of insertion and injection during needle withdrawal is repeated (**Figure 3, right panel**). Using this process, CMP-001 is injected along multiple tracks through a single insertion point as far as the radial reach of the needle allows within the tumor; two insertion points can be used if the tumor is larger than the radial reach of the needle and the intended CMP-001 volume cannot be delivered from a single insertion point. If gentle injection pressure along 5 needle tracks within the tumor has not succeeded in delivering the desired volume, then the remainder of the CMP-001 may be injected peri-tumorally, around the same lesion.

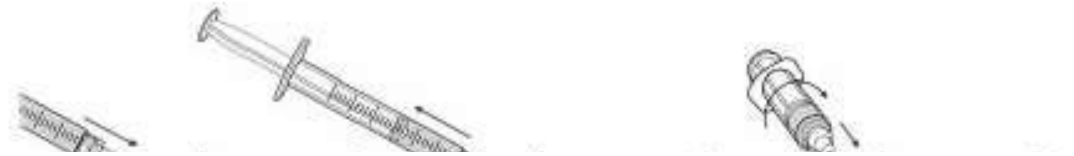

Figure 3: Method for CMP-001 Intra-Tumoral Injection

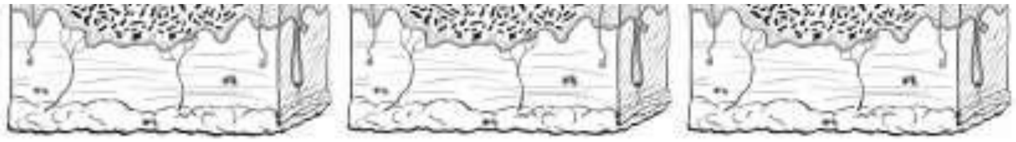

Essentially the same process is performed for cutaneous, subcutaneous, and nodal injections, but for deeper injections, the tip of the needle may be kept within the tumor and a longer needle may be used.

5.2.2.6 Observation periods

During the Prime Phase, subjects must be observed for a period of at least four hours following each of the first three CMP-001 injections (W1D1, W2D1, W3D1) for signs and symptoms of reactions to the injection and other adverse events. Beginning with the fourth CMP-001 injection (W4D1) the observation period may be reduced to 1 hour following CMP-001 injection or per the discretion of the Investigator and agreement by Sponsor.

During the Boost Phase, subjects must be observed for a minimum of 1 hour following each CMP-001 injection for signs and symptoms of reactions to the injection and other adverse events. Observation periods longer than 1 hour during the Boost Phase should be based on the adverse event profile of the individual subject and be performed at the discretion of the Investigator.

5.2.2.7 Management of CMP-001 toxicity

<u>CMP-001 Associated Adverse Events</u>

If subjects develop inflammation at the injection site this may be managed using cold compresses and/or acetaminophen or non-steroidal anti-inflammatory agents. If flu-like symptoms (i.e., fever, myalgia, and headache) arise, these may be managed using acetaminophen or non-steroidal anti-inflammatory agents.

It is expected that symptoms associated with a large release in cytokines at the time of injection could present 1-4 hours after the injection. These symptoms could include: fever, nausea/vomiting, chills/rigors, and hypotension. Subjects must remain in observation until all of these symptoms have resolved to ≤ Grade 1. Refer to **Table 4.1.4-1** for current CMP-001 safety profile in the ongoing CMP-001-001 clinical study.

Subjects who experience a DLT will have dosing of CMP-001 withheld until the toxicity has returned to ≤ Grade 1. If the Investigator determines that continued treatment with CMP-001 is in the subject's best interest, treatment may resume at a reduced dose of CMP-001. Only one dose reduction is allowed. Subjects who still experiences DLTs at Dose Level-1 must come off study.

<u>CMP-001 Associated Cytokine Release-like Syndrome</u>

As noted in **Section 4.2.2**, CMP-001 has been associated with symptoms associated with cytokine release. Most notable severe cases of hypotension have been noted, most often in subjects with a prior medical history of adrenal insufficiency secondary to ipilimumab. The backbone of the recommended treatment algorithm is the use of stress dose steroids and the IL6 antagonist tocilizumab (Maude et al. 2014; Lee et al. 2014; Tanaka et al. 2016; Brudno and Kochenderfer 2018).

The recommended algorithm for the treatment of symptoms associated with cytokine release below has been adapted for the treatment of cytokine release syndrome resulting from other immunotherapies such as CAR-T cell therapy (Lee et al. 2014) and is depicted in **Figure 4**. However, its effectiveness in treating symptoms associated with cytokine release resulting from CMP-001 has not been previously studied. The first line of treatment for hypotension unresponsive to supportive care such as fluids, is stress dose steroids as outlined below. Additional treatment measures include the use of drugs targeting specific cytokines believed to be involved in the development of cytokine release syndrome-induced hypotension. It is recommended that the treatment algorithm be implemented in subjects who present with acute hypotension following injection of CMP-001.

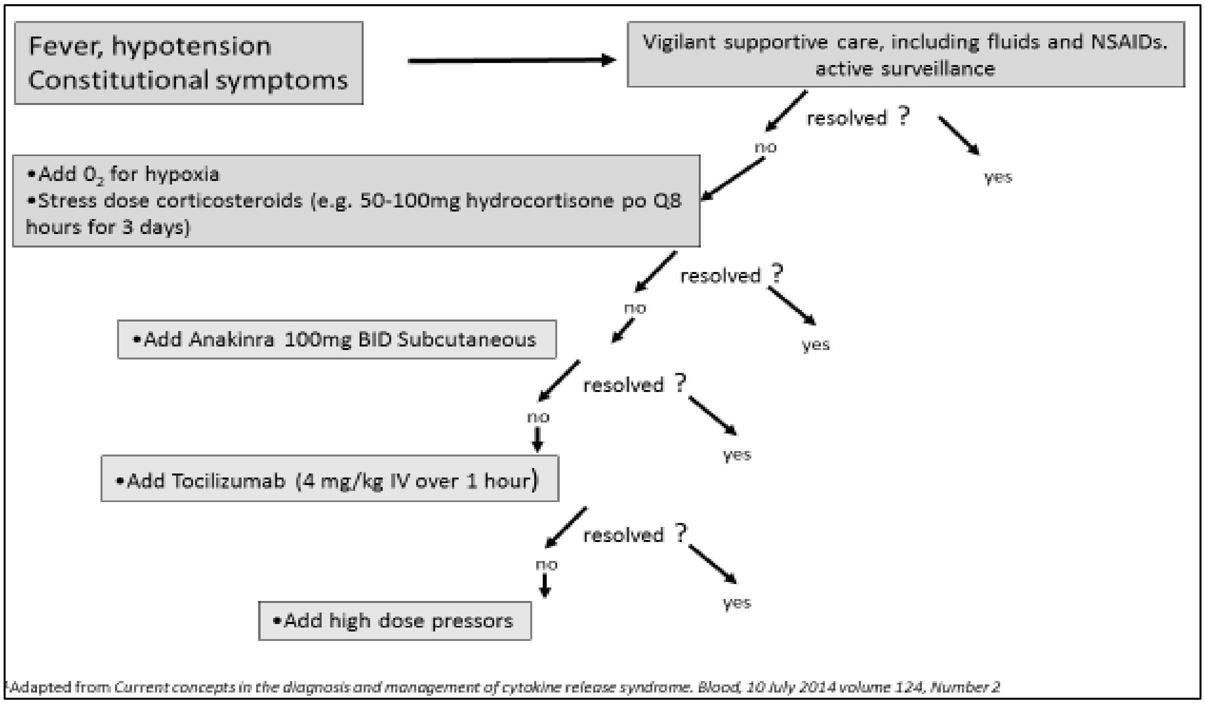

Figure 4: Management of CMP-001 Associated Cytokine Release-like Syndrome

5.2.2.8 Changing the selected tumor

Therapy of a selected lesion generally should continue per protocol unless the tumor is EITHER dramatically responding clinically OR dramatically progressing clinically. Inflammation of the injected tumor is expected following the 2nd and subsequent injections, but should not be mistaken for anti-tumor response or clinical progression.

If during therapy the injected tumor is clearly involuting and shrinking, and if a different eligible accessible tumor is progressing, then the Investigator should change the selection to the progressing tumor, injecting the full dose of CMP-001 into that tumor(s), and continuing to inject the new tumor(s) until EOT (or that tumor shows involution, in which case a third progressing tumor may be selected). If during therapy the injected tumor is clearly involuting and shrinking, and no other lesions are present, CMP-001 may be omitted or injected subcutaneously at the discretion of the treating investigator in consultation with the Principle Investigator.

If during therapy the injected tumor progresses, the treating investigator in consultation with the Principle Investigator may elect to continue therapy or transition to surgery directly. Every effort should be made to complete 7 weeks of perioperative therapy. The decision to proceed with surgery should be made (in consultation with the initial evaluating surgical oncologist) only if there is a danger of the subject being rendered inoperable.

5.3 Treatment Allocation

During the Prime Phase, patients will receive nivolumab 240mg every 2 weeks in combination with CMP-001 5mg (week 1), and CMP-001 10mg intra-tumorally (weeks 2-7). Subsequently patients will undergo surgical resection.

During the Boost Phase, patients will receive nivolumab either 240mg every 2 weeks (or 480mg every 4 weeks) at Investigator's discretion along with CMP-001 5mg every 4 weeks.

5.4 Stratification

None.

5.5 Concomitant Medications

Medications or vaccinations specifically prohibited in the exclusion criteria are not allowed during the ongoing trial. If there is a clinical indication for one of these or other medications or vaccinations specifically prohibited during the trial, discontinuation from trial therapy or vaccination may be required. The investigator should discuss any questions regarding this with the principal investigator. The final decision on any supportive therapy or vaccination rests with the investigator and/or the subject's primary physician. However, the decision to continue the subject on trial therapy or vaccination schedule requires the mutual agreement of the treating physician investigator, the principal investigator, and the subject.

5.5.1 Acceptable Concomitant Medications

All treatments that the investigator considers necessary for a subject's welfare may be administered at the discretion of the investigator in keeping with the community standards of medical care. All concomitant medication will be recorded on the case report form (CRF) including all prescription, over-the-counter (OTC), herbal supplements, and IV medications and fluids. If changes occur during

the trial period, documentation of drug dosage, frequency, route, and date may also be included on the CRF.

All concomitant medications received within 28 days before the first dose of trial treatment and 30 days after the last dose of trial treatment should be recorded. Concomitant medications administered after 30 days after the last dose of trial treatment should be recorded for SAEs and ECIs as defined in **Section 7.2**.

5.5.2 Prohibited Concomitant Medications

Subjects are prohibited from receiving the following therapies during the Screening and Treatment Phase (including retreatment for post-complete response relapse) of this trial:

- Anti-cancer systemic chemotherapy or biological therapy
- Immunotherapy not specified in this protocol
- Chemotherapy not specified in this protocol
- Investigational agents other than CMP-001
- Radiation therapy

Live vaccines within 30 days prior to the first dose of trial treatment and while participating in the trial. Examples of live vaccines include, but are not limited to, the following: measles, mumps, rubella, chicken pox, yellow fever, rabies, BCG, and typhoid (oral) vaccine. Seasonal influenza vaccines for injection are generally killed virus vaccines and are allowed; however intranasal influenza vaccines (e.g. Flu-Mist®) are live attenuated vaccines, and are not allowed.

Glucocorticoids for any purpose other than to modulate symptoms from an event of clinical interest of suspected immunologic etiology. The use of physiologic doses of corticosteroids may be approved after consultation with the Principle Investigator.

Subjects who, in the assessment by the investigator, require the use of any of the aforementioned treatments for clinical management should be removed from the trial. Subjects may receive other medications that the investigator deems to be medically necessary.

The Exclusion Criteria describes other medications which are prohibited in this trial.

There are no prohibited therapies during the Post-Treatment Follow-up Phase.

Agents known to have TLR9 antagonist activity are prohibited throughout the study. The known antagonists are chloroquine, hydroxychloroquine, and quinacrine.

Medications intended solely for supportive care (i.e., antiemetics, analgesics, megestrol acetate for anorexia) are allowed.

5.6 Rescue Medications and Supportive Care

5.6.1 Supportive Care Guidelines

Subjects should receive appropriate supportive care measures as deemed necessary by the treating investigator. Suggested supportive care measures for the management of adverse events with potential immunologic etiology are outlined below. Where appropriate, these guidelines include the use of oral or intravenous treatment with corticosteroids as well as additional anti-inflammatory agents if symptoms do not improve with administration of corticosteroids. Note that several courses of steroid tapering may be necessary as symptoms may worsen when the steroid dose is decreased.

For each disorder, attempts should be made to rule out other causes such as metastatic disease or bacterial or viral infection, which might require additional supportive care. The treatment guidelines are intended to be applied when the investigator determines the events to be related to nivolumab.

Note: if after the evaluation the event is determined not to be related, the investigator does not need to follow the treatment guidance (as outlined below). **Refer to Section 5.2.1 for dose modification.**

It may be necessary to perform conditional procedures such as bronchoscopy, endoscopy, or skin photography as part of evaluation of the event.

- **Pneumonitis**
  - For **Grade 2 events**, treat with systemic corticosteroids. When symptoms improve to Grade 1 or less, steroid taper should be started and continued over no less than 4 weeks.
  - For **Grade 3-4 events**, immediately treat with intravenous steroids. Administer additional anti-inflammatory measures, as needed.
  - Add prophylactic antibiotics for opportunistic infections in the case of prolonged steroid administration.
- **Diarrhea/Colitis**
  - Subjects should be carefully monitored for signs and symptoms of enterocolitis (such as diarrhea, abdominal pain, blood or mucus in stool, with or without fever) and of bowel perforation (such as peritoneal signs and ileus).
  - All subjects who experience diarrhea/colitis should be advised to drink liberal quantities of clear fluids. If sufficient oral fluid intake is not feasible, fluid and electrolytes should be substituted via IV infusion. For Grade 2 or higher diarrhea, consider GI consultation and endoscopy to confirm or rule out colitis.
  - For **Grade 2 diarrhea/colitis,** administer oral corticosteroids.
  - For **Grade 3 or 4 diarrhea/colitis,** treat with intravenous steroids followed by high dose oral steroids.
  - When symptoms improve to Grade 1 or less, steroid taper should be started and continued over no less than 4 weeks.
- **Type 1 diabetes mellitus (if new onset, including diabetic ketoacidosis [DKA]) or ≥ Grade 3 Hyperglycemia, if associated with ketosis (ketonuria) or metabolic acidosis (DKA)**
  - For **T1DM** or **Grade 3-4** Hyperglycemia
    - Insulin replacement therapy is recommended for Type I diabetes mellitus and for Grade 3-4 hyperglycemia associated with metabolic acidosis or ketonuria.
    - Evaluate patients with serum glucose and a metabolic panel, urine ketones, glycosylated hemoglobin, and C-peptide.
- **Hypophysitis**
  - For **Grade 2** events, treat with corticosteroids. When symptoms improve to Grade 1 or less, steroid taper should be started and continued over no less than 4 weeks. Replacement of appropriate hormones may be required as the steroid dose is tapered.
  - For **Grade 3-4** events, treat with an initial dose of IV corticosteroids followed by oral corticosteroids. When symptoms improve to Grade 1 or less, steroid taper should be started and continued over no less than 4 weeks. Replacement of appropriate hormones may be required as the steroid dose is tapered.

- **Hyperthyroidism or Hypothyroidism**
  - Thyroid disorders can occur at any time during treatment. Monitor patients for changes in thyroid function (at the start of treatment, periodically during treatment, and as indicated based on clinical evaluation) and for clinical signs and symptoms of thyroid disorders.
  - **Grade 2** hyperthyroidism events (and **Grade 2-4** hypothyroidism):
    - In hyperthyroidism, non-selective beta-blockers (e.g. propranolol) are suggested as initial therapy.
    - In hypothyroidism, thyroid hormone replacement therapy, with levothyroxine or liothyroinine, is indicated per standard of care.
  - **Grade 3-4** hyperthyroidism
    - Treat with an initial dose of IV corticosteroid followed by oral corticosteroids. When symptoms improve to Grade 1 or less, steroid taper should be started and continued over no less than 4 weeks. Replacement of appropriate hormones may be required as the steroid dose is tapered.
- **Hepatic**
  - For **Grade 2** events, monitor liver function tests more frequently until returned to baseline values (consider weekly).
    - Treat with IV or oral corticosteroids
  - For **Grade 3-4** events, treat with intravenous corticosteroids for 24 to 48 hours.
  - When symptoms improve to Grade 1 or less, a steroid taper should be started and continued over no less than 4 weeks.
- **Nephritis**
  - For **Grade 2** events, treat with corticosteroids.
  - For **Grade 3-4** events, treat with systemic corticosteroids.
  - When symptoms improve to Grade 1 or less, steroid taper should be started and continued over no less than 4 weeks.
- Management of infusion reactions
- Signs and symptoms usually develop during or shortly after drug infusion and generally resolve completely within 24 hours of completion of infusion.

  Table 5.6.1-1 below shows treatment guidelines for subjects who experience an infusion reaction associated with administration of nivolumab.

Table 5.6.1-1: Infusion Reaction Management Guidelines

**NCI CTCAE Grade**	**Treatment**	**Premedication at Subsequent Dosing**
Grade 1<br>Mild reaction; infusion interruption not indicated; intervention not indicated	Increase monitoring of vital signs as medically indicated until the subject is deemed medically stable in the opinion of the investigator.	None
Grade 2<br>Requires infusion interruption but responds promptly to symptomatic treatment (e.g., antihistamines, NSAIDS, narcotics, IV fluids); prophylactic medications indicated for < =24 hrs	**Stop Infusion and monitor symptoms.**<br>Additional appropriate medical therapy may include but is not limited to:<br>IV fluids<br>Antihistamines<br>NSAIDS<br>Acetaminophen<br>Narcotics	Subject may be premedicated 1.5h (± 30 minutes) prior to infusion of nivolumab with:<br><br>Diphenhydramine 50 mg po (or equivalent dose of antihistamine).

	Increase monitoring of vital signs as medically indicated until the subject is deemed medically stable in the opinion of the investigator.<br>If symptoms resolve within one hour of stopping drug infusion, the infusion may be restarted at 50% of the original infusion rate (e.g., from 100 mL/hr to 50 mL/hr). Otherwise dosing will be held until symptoms resolve and the subject should be premedicated for the next scheduled dose.<br>Subjects who develop Grade 2 toxicity despite adequate premedication should be permanently discontinued from further trial treatment administration.	Acetaminophen 500-1000 mg po (or equivalent dose of antipyretic).
Grades 3 or 4<br>Grade 3:<br>Prolonged (i.e., not rapidly responsive to symptomatic medication and/or brief interruption of infusion); recurrence of symptoms following initial improvement; hospitalization indicated for other clinical sequelae (e.g., renal impairment, pulmonary infiltrates)<br>Grade 4:<br>Life-threatening; pressor or ventilatory support indicated	**Stop Infusion.**<br>Additional appropriate medical therapy may include but is not limited to:<br>IV fluids<br>Antihistamines<br>NSAIDS<br>Acetaminophen<br>Narcotics<br>Oxygen<br>Pressors<br>Corticosteroids<br>Epinephrine<br>Increase monitoring of vital signs as medically indicated until the subject is deemed medically stable in the opinion of the investigator.<br>Hospitalization may be indicated.<br>Subject is permanently discontinued from further trial treatment administration.	No subsequent dosing
Appropriate resuscitation equipment should be available in the room and a physician readily available during the period of drug administration		

5.7 Subject Withdrawal/Discontinuation Criteria

Subjects are free to withdraw from the study at any time and without penalty or loss of future medical care, or any other benefits to which they are otherwise entitled. Subjects may discontinue study medication for any of the following conditions:

- Dose-limiting or other unacceptable toxicity considered to be related to either study medication;
  - Patients who develop unacceptable toxicity deemed related to CMP-001 but not Nivolumab may continue Nivolumab after CMP-001 discontinuation for duration as defined in **Section 5.2.2.4**.
  - Patients who develop unacceptable toxicity deemed related to Nivolumab but not CMP-001 must discontinue **both CMP-001 and Nivolumab.**
- PD by RECIST (Version 1.1) if accompanied by medically significant clinical deterioration, in the judgment of the Investigator. (Continuation of treatment through suspected pseudo-progression is permitted.);
- If, in the opinion of the Investigator, it is medically necessary;
- Subject withdraws consent for the study (note that subjects who withdraw consent for additional study treatment and procedures will continue to be followed for long-term
- survival unless they explicitly withdraw consent for any follow-up);
- Subject develops an intercurrent illness or adverse event that precludes further participation, or requires a prohibited concomitant treatment;
- Subject becomes pregnant or begins breastfeeding;

- Subject is lost to follow-up.

Subjects discontinuing study medication earlier than planned or withdrawing from the study should undergo the subsequent End of Treatment (EOT) clinical and laboratory assessments as soon as possible after study medication is stopped and the requisite safety follow-up period of 30 days should also be followed. The reason for withdrawal will be recorded in the electronic case report form (eCRF) and the subject’s source medical record. All subjects will continue to be followed every three months after the last CMP-001 treatment for long-term survival follow-up until death, loss to follow-up, or withdrawal of consent for follow-up.

5.8 Subject Replacement Strategy

Subjects who receive at least 1 dose of CMP-001 and nivolumab are evaluable for safety. Patients who complete pre-operative therapy and receive surgery will be evaluable for pathologic response rate and other efficacy endpoints. Patients who did not undergo surgery for reasons of disease progression precluding surgery (rapid disease progression) will be deemed non-evaluable. Non-evaluable subjects may be replaced.

If in the opinion of the treating investigator and treating surgical oncologist in consultation with Principle Investigator, subject’s disease is rapidly progressing to the point that window to intervene surgically is narrowing, treatment may be discontinued to permit surgery. These patients are evaluable for pathological response rate and other efficacy endpoints. These patients are eligible to receive CMP-001/nivolumab during “Boost” phase.

5.9 Clinical Criteria for Early Trial Termination

Early trial termination will be the result of the criteria specified below:

- Quality or quantity of data recording is inaccurate or incomplete
- Poor adherence to protocol and regulatory requirements
- Incidence or severity of adverse drug reaction in this or other studies indicates a potential health hazard to subjects
- Plans to modify or discontinue the development of the study drugs. In the event of CheckMate decision to no longer supply study drug, ample notification will be provided so that appropriate adjustments to subject treatment can be made.

5.10 Surgical Considerations

The team members will make every effort to comply with the surgical procedures within the time windows allowed in this study protocol. However, under certain circumstances and as discussed between the sub-investigator(s) and the principal investigator, an alteration in the surgical schedule/timing is allowed. These include (but are not limited to) the following:

- A 4 week window between the baseline biopsy and the first dose of nivolumab is allowed.
- The definitive surgery may be a lymphadenectomy or resection of lymphatic metastases as clinically indicated. The definitive surgery may be divided into more than one surgical procedure, scheduled at different time points as clinically/surgically indicated. Under certain circumstances, the definitive surgery may be done after completion of additional therapy (e.g., after the forth dose of nivolumab) or earlier if nivolumab was discontinued due to adverse events or other reasons. Tissue from any of these surgical procedures may be banked under this protocol following the same protocol procedures for tissue handling and banking. The tissue for banking may be digested, frozen or handled in another alternative method as discussed with the investigator.

- If for some reason, the baseline biopsy or definitive surgery could not be completed due to un-anticipated factors such as patient refusal, this will be documented.

NOTE: The surgical guidelines are general and may be followed or modified at the discretion of the expert surgical oncologist on the case as clinically indicated and depending on the specifics of the individual surgical case.

For the baseline biopsy required on this study, patients will undergo a core needle, punch biopsy, open surgical biopsy, or other technique at the discretion of the sub-investigator/investigator. The choice of specific surgical technique will be at the discretion of the surgeon or medical oncologist and actual techniques will be the same as those used as therapeutically or diagnostically indicated to surgically treat or confirm the clinical suspicion of recurrent or metastatic disease in any patient undergoing conventional evaluation and treatment (standard of care) within 21 days (preferred; and up to 4 weeks) prior to entry to the study.

5.10.1 Primary Excision or Primary Cutaneous Melanoma

All patients with initial presentation of melanoma T1-4 will be treated by wide excision of the primary. Definitive surgery will include wide excision of the primary and sentinel lymph node assessment. For patients with known primary cutaneous melanoma lesion and no history of wide local excision of that primary lesion, an adequate wide excision of the primary lesion (minimum margin 1 cm) is recommended. The wide local excision will be done at the time of complete lymphadenectomy. Patients with nodal relapse after an inadequate primary excision will undergo wide excision at the time of complete lymphadenectomy. The recommendation for adequate wide excision is the same for patients enrolled at the time of lymph node recurrence as for those enrolled at the time of initial treatment of the primary. For lesions whose Breslow's thickness is > 1 mm, a 2 cm minimum margin is preferred when anatomically feasible (i.e., for lesions of the trunk and proximal extremities). For subungual melanoma, a distal interphalangeal amputation with histologically negative margins constitutes an adequate wide excision. The specimen shall be excised to include skin and all subcutaneous tissue down to the muscular fascia. Fascia may be included at the discretion of the operating surgeon. Closure of the defect may be via primary closure, split thickness skin graft, or rotation-flap at the discretion of the surgeon.

5.10.2 Regional Lymphadenectomy

Note: For patients undergoing sentinel node mapping and lymphoscintigraphic and dye lymphographic identification of regional nodal drainage, the minimum number of nodes may be less than the mandatory minimum numbers of 5/groin, 10/axilla, and 15/cervical node dissection.

All patients should undergo one of the following staging lymphadenectomies, as found surgically applicable by the treating surgical oncologist.

5.10.2.1 Head and neck lesions

Face, ear and anterior scalp: Modified radical neck or radical neck dissection. Parotidectomy to be included if lymphoscintogram indicates flow to the area.

Submandibular and anterior neck: Modified radical or radical neck dissection.

Posterior scalp, posterior neck and uppermost trunk (areas that drain to posterior cervical triangle): Modified posterior triangle neck dissection with suboccipital nodes.

At all times, the number of

5.10.2.2 Upper extremity

Axillary node dissection to include at least 10 nodes taken from levels I and II. Level III nodes should be dissected if they are clinically involved. The pectoralis minor muscle may be divided or sacrificed at the surgeon's discretion.

5.10.2.3 Lower extremity

Superficial inguinal node dissection or deep inguinal node dissection will be performed at the discretion of the surgeon. Inguinal node dissection to include at least 5 nodes.

5.10.3 Lymphadenectomy for Nodal Recurrence

Regional node recurrences will be treated using the appropriate lymphadenectomy procedure. Whenever possible, diagnosis of regional node recurrence will be made using punch or open biopsy technique (if done as part of a clinically indicated baseline diagnostic procedure) to provide baseline pathologic material needed for evaluation of treatment effect. At the time of the definitive lymphadenectomy, the biopsy site will be included in the operative specimen.

6 Trial Flow Chart

The Investigator/Sub-Investigators are expected to make every reasonable effort to comply with the study calendar. However, if a treatment is missed or a subject's study treatment and/or testing days need to be rescheduled due to the subject’s inability to comply with the study calendar (i.e., hospitalizations, business and vacation travel plans, illness, transportation issues, holidays, family emergencies, etc.), a window of ± one week is available for rescheduling of study treatment and procedures per the discretion of the Sub- Investigator, and as discussed with the Investigator. For the baseline biopsy, a 4 week window is allowed before the first dose of nivolumab and/or CMP-001.

6.1 Study Flow Chart

**Procedure/ Assessment**	**Screening (Day -28 to -1)[A]**	**Prime Phase (Weeks 1-7)**								**Surgery**	**Boost Phase (Weeks 12-56)**	**End of Treatment[B]**	**30 Day Follow-Up[O]**	**Post-treatment surveillance[N]**
		**W1 D1**	**W2 D1**	**W3 D1**	**W4 D1**	**W5 D1**	**W6 D1**	**W7 D1**	**W7 D2**	**W8-W10**	**Every 4 weeks[D] starting 3 weeks after surgery**			
Visit Windows		+/- 2 days							+/- 5 days		+/- 3 days	+/- 7 days	+/- 7 days	+/- 14 days
CMP-001 dose number[C]	N/A	1	2	3	4	5	6	7	N/A	N/A	1-12 (12 doses)			
Nivolumab dose number[D]	N/A		1		2		3		N/A	N/A	[D]1-23 (23 doses – if q2)<br>[D]1-12 (12 doses – if q4)			
Tumor Biopsy[E]	X											X[B]		
Planned Surgical Evaluation and Resection[F]	X (evaluation)								X (evaluation)	X (resection)				
Exploratory Biomarker Analyses[G]		X		X				X			X (every 8 weeks starting with Week 12)			
Informed consent	X													
Eligibility Criteria Assessment	X													
Medical History, Demographics, Cancer History including Prior Cancer Medications	X													
Physical Exam[H]	X	X	X	X	X	X	X	X			X	X	X	X
Electrocardiogram (ECG)[I]	X	X		X				X						
Autoimmune lab panel[J]	X											X		

Clinical laboratory tests (hematology, serum chemistry, other chemistry, urinalysis, coagulation tests and thyroid function)[J]	X		X		X		X				X		X (CBC, CMP, LDH only)	X (CBC, CMP, LDH only)
Urine Pregnancy Test[K]	X													
Disease Assessment (CT/MRI)[L]	X								X		X (every 8 weeks starting with Week 20)			
Stool sampling[M]	X			X				X			X (every 8 weeks starting with Week 12)			
Dietary history questionnaire[N]	X			X				X			X (every 8 weeks starting with Week 12)			
Adverse Event Monitoring	Assessed continually from informed consent through EOT + 30 days													
Concomitant Medications	Assessed continually from first CMP-001 dose through EOT + 30 days													
30 Day Follow Up[O]													X	

Study Calendar Notes:

[A]≤4 weeks prior to first dose; magnetic resonance imaging (MRI)/computed tomography (CT) scans may be performed ≤4 weeks prior to first CMP-001 dose**.**

- During Screening, MRI brain with contrast (or CT equivalent) should be performed to exclude CNS metastatic disease. CNS imaging need not be repeated on study unless patient is symptomatic.

[B]End-of-treatment (EOT) assessments to be performed within 7 days following removal of subject from CMP-001 treatment. Removal of a subject from CMP-001 treatment is defined as the time in which the Investigator decides to discontinue CMP-001 treatment for a subject.

- Management of patients who develop unacceptable toxicity is defined in **Section 5.6**.
    - Patients who develop unacceptable toxicity deemed related to CMP-001 but not Nivolumab may continue Nivolumab after CMP-001 discontinuation for duration as defined in **Section 5.2.2.4**.
    - Patients who develop unacceptable toxicity deemed related to Nivolumab but not CMP-001 must discontinue both CMP-001 and Nivolumab.
- Patients who progress will undergo an optional biopsy to confirm progression at the time of RECIST v1.1 confirmed progression. This biopsy should be done within 14 days of RECIST v1.1 confirmed progression determination.

[C]CMP-001 is dosed weekly during the Prime Phase and q4 weekly during the Boost Phase of this study.

- Vital sign monitoring.
    - Weeks 1-3 (Prime Phase): vital signs are to be collected within 15 minutes prior to CMP-001 injection, and at 30 (±15) minute intervals for 4 hours following each of the first three CMP-001 injections.
    - Weeks 4-7 (Prime Phase): vital signs are to be collected within 15 minutes prior to CMP-001 injection, and at 30 (±15) minute intervals for 1 hour following each of the first three CMP-001 injections.
    - Boost Phase (all doses): vital signs are to be collected within 15 minutes prior to CMP-001 injection, and at 30 (±15) minute intervals for 1 hour following each of the first three CMP-001 injections.

[D]Nivolumab dosing schedule is q2 weekly during the Prime Phase and either q2 weekly or q4 weekly during the Boost.

- During the Boost Phase of this study, Nivolumab can be dosed q2 or q4 weekly at the discretion of the treating investigator.
- If Nivolumab is dosed at q2 weeks during Boost phase, the patient will receive 23 doses of Nivolumab (doses 1-23; weeks 12-56).
- If Nivolumab is dosed at q4 weeks during Boost phase, the patient will receive 12 doses of Nivolumab (doses 1-12; weeks 12-56).

[E]Tumor biopsy must be performed prior to commencing CMP-001/nivolumab treatment. Tumor biopsy must be of lesion that is planned for resection. Punch, core (>4 18gauge) and/or surgical biopsies are acceptable.

- If core biopsy is performed, pathology confirmation is required to ensure adequate tissue is obtained.

[F]Assessment of planned resectability must be made DURING screening. Planned surgery after Prime Phase must be with surgeon who initially made determination of planned resectability.

- Extent of surgery to follow guidelines based on location of involved lymph node as delineated in **Section 5.10**.

[G]Exploratory biomarker blood samples are to be collected at the following times:

- Prior to administration of the 1st, 3rd, and 7th CMP-001 doses (W1D1, W3D1, W7D1) respectively.
- Every 8 weeks prior to administration of nivolumab following surgery starting with Week 12.

[H]A full physical exam will be conducted at screening, each treatment visit and EOT.

- Screening physical exam may be performed up to 72 hours prior to the Week 1 Day 1 visit. If the full physical exam is performed >72 hours prior to the Week 1 Day 1 (W1D1) visit, then a brief (symptom directed) physical exam must be performed within 72 hours prior to initiation of CMP-001.
- Brief physical exams focused on areas of disease or adverse events are performed at the 1st, 3rd, 5th and 7th CMP-001 doses at W1D1, W3D1, W5D1, W7D1 and at any other time as clinically indicated.
- Both full and brief physical exams should include assessment of ECOG performance status.
- Full physical exams should include height and weight.

[I]Electrocardiograms (ECGs) should be obtained at Screening and prior to administration of CMP-001 at the 1st, 3rd, and 7th CMP-001 dosing visits (on W1D1, W3D1, and W7D1.

- ECG testing is not required during Boost Phase

[J]Clinical laboratory tests may be performed up to 72 hours prior to administration of CMP-001.

- Laboratory tests to be obtained are delineated in **Table 7.1.5.1-1**.

- Autoimmune lab panel to be collected at Screening and EOT only.

[K]Pregnancy testing should be performed in women of childbearing potential and at Screening only.

- This should be done within 7 days prior to receiving the first dose of study drug).
- Any method (urine pregnancy test; serum β-human chorionic gonadotropin) can be utilized for this purpose.

[L]CT or MRI scans to assess tumor status will be obtained at Screening (baseline value should be performed ≤4 weeks prior to first CMP-001 dose).

- Screening:
  - Scans should be performed ≤4 weeks prior to first CMP-001 dose).
- Prime Phase:
  - CT (or PET/CT) scans should be repeated prior to surgery (+/- 2 week window).
- During Boost Phase, CT or MRI scans will be repeated every 8 weeks starting with week 20 (or after 2 doses of CMP-001 administered during the Boost Phase **whichever is sooner**).
  - If Nivolumab is dosed at q2 weeks during Boost phase, 1st restaging scan will occur after 4 doses of nivolumab at Week 20 and be repeated q8 weeks.
  - If Nivolumab is dosed at q4 weeks during Boost phase, 1st restaging scan will occur after 2 doses of nivolumab at Week 20 and be repeated q8 weeks.

[M]Stool sampling will be performed at Screening and at W3D1 and W7D1 during Prime Phase. During Boost Phase, these will be repeated every 8 weeks starting Week 12.

[N]Dietary history questionnaire will be performed concordantly with stool sampling at Screening and at W3D1 and W7D1 during Prime Phase. During Boost Phase, these will be repeated every 4 weeks starting with Week 12. Dietary questionnaire is detailed in **Appendix 1**.

[O]30 day follow up

  - This begins +30 days following completion of Cycle 12 Boost Phase.
  - Procedures to be followed during this phase are outlined in **Section 7.1.7.3** and will include a visit, bs (CBC, CMP, LDH, TSH, free T4) and any other studied deemed necessary at the discretion of the treating physicians and according to established Standard of Care**.**

[N]Post-treatment surveillance (for patients that have completed treatment or removed due to toxicity):

- This phase begins upon completion of 30 day follow up for patients who have completed treatment or come off study due to toxicity.
- Procedures to be followed during this phase are outlined in **Section 7.1.7.2** and will include imaging (CT or PET/CT), labs (CBC, CMP, LDH, TSH, free T4) and any other studied deemed necessary at the discretion of the treating physicians and according to established Standard of Care.

7 Trial Procedures

7.1 Trial Procedures

The Trial Flow Chart - Section 6.0 summarizes the trial procedures to be performed at each visit. Individual trial procedures are described in detail below. It may be necessary to perform these procedures at unscheduled time points if deemed clinically necessary by the investigator.

Furthermore, additional evaluations/testing may be deemed necessary by the Sponsor and/or CheckMate for reasons related to subject safety. In some cases, such evaluation/testing may be potentially sensitive in nature (e.g., HIV, Hepatitis C, etc.), and thus local regulations may require that additional informed consent be obtained from the subject. In these cases, such evaluations/testing will be performed in accordance with those regulations.

7.1.1 Treatment period

The study consists of 3 main phases:

- **Prime Phase (Weeks 1-7)**:
  - Nivolumab (240 mg) I.V. infusion every 2 weeks for 3 doses (starting 2nd week of CMP-001 administration – W2D1) given concurrently with CMP-001.
  - CMP-001 will be administered subcutaneously (week 1), then intra-tumorally (weeks 2-7) on a weekly schedule.
- **Definitive Surgery**: Follows completion of prime phase.
- **Boost Phase** (following recovery from surgery) (**Weeks 12-56**):
  - Nivolumab (240 mg I.V. infusion every 2 weeks; or 480 mg I.V infusion every 4 weeks) given concurrently with CMP-001 5mg every 4 weeks.
  - CMP-001 will be administered subcutaneously (peri-tumoral resection site).
  - Total of 12 doses of CMP-001 will be administered.
  - Total of 46 weeks of Nivolumab will be administered (every 2 weeks - 23 doses; every 4 weeks – 12 doses).

7.1.2 Administrative Procedures

Informed consent must be documented by the subject's dated signature or by the subject's legally acceptable representative's dated signature on a consent form along with the dated signature of the person conducting the consent discussion prior to enrollment.

During initial enrollment visit, study investigator (or designee) must discuss the following with patients:

- Inclusion and exclusion criteria
- Medical history – pertinently any condition diagnosed within the prior 10 years that are considered to be clinically significant by the Investigator.
- Prior and concomitant medications. All medications related to reportable SAEs and ECIs should be recorded as defined in **Section 7.2.**
- Disease details and treatments.

7.1.3 Clinical Procedures/Assessments

7.1.3.1 Adverse event (AE) monitoring

The investigator or qualified designee will assess each subject to evaluate for potential new or worsening AEs as specified in the Trial Flow Chart and more frequently if clinically indicated. Adverse experiences will be graded and recorded throughout the study and during the follow-up period according to NCI CTCAE Version 4.0 (see Section 12.2). Toxicities will be characterized in terms regarding seriousness, causality, toxicity grading, and action taken with regard to trial treatment.

All AEs of unknown etiology associated with nivolumab exposure should be evaluated to determine if it is possibly an event of clinical interest (ECI) of a potentially immunologic etiology (irAE). See **Section 5.6.1.1** regarding the identification, evaluation and management of AEs of a potential immunological etiology.

Related and clinically significant AEs occurring within 30 days after the last dose of study drug should be recorded. Patients who are discontinued from the study due to an unacceptable drug-related AE will be followed until the resolution of the AE to Grade 0-1 or stabilization or until initiation of a new therapy for their cancer, whichever occurs first. Related SAEs that occur within 90 days of the end of treatment or before initiation of a new antineoplastic treatment should also be followed and recorded.

Please refer to section 7.2 for detailed information regarding the assessment and recording of AEs.

7.1.3.2 Full physical exam

The investigator or qualified designee will perform a complete physical exam during the screening period. Clinically significant abnormal findings should be recorded as medical history. A full physical exam should be performed during screening.

7.1.3.3 Directed physical exam

For visits that do not require a full physical exam per the Trial Flow Chart, the investigator or qualified designee will perform a directed physical exam as clinically indicated prior to trial treatment administration.

7.1.3.4 Vital signs

The investigator or qualified designee will take vital signs at screening, prior to the administration of each dose of trial treatment and at treatment discontinuation as specified in the Trial Flow Chart (Section 6.1). Vital signs should include temperature, pulse, respiratory rate, weight and blood pressure. Height will be measured at screening only.

7.1.3.5 Eastern Cooperative Oncology Group (ECOG) performance scale

The investigator or qualified designee will assess ECOG status (see Section 12.4) at screening, prior to the administration of each dose of trial treatment and discontinuation of trial treatment as specified in the Trial Flow Chart.

7.1.3.6 Tumor imaging and assessment of disease

Response and progression will be evaluated in this study using the international criteria proposed by the Response Evaluation Criteria in Solid Tumors (RECIST v.1.1) Committee [Eisenhauer. Eur J Cancer 2009; (45):228-47].

Acceptable imaging studies to evaluate disease at baseline include:

- Whole body PET-CT scan (except brain)
- Contrast-enhanced CT scan of neck, chest, abdomen and pelvis (and affected body part if needed).
- Brain imaging is to done only at Screening and need not be repeated. For brain imaging an contrast enhanced MRI is ideal. If an MRI of the brain cannot be done (or is contraindicated) a contrast enhanced CT of the brain is acceptable.

A restaging PET-CT (preferred if possible, or CT) is required after completion of Prime Phase (+/- 2 weeks) and prior to definitive surgery (i.e. after the completion of induction doses of nivolumab and CMP-001). MRI brain is not required for follow up studies unless clinically indicated. Additional staging studies will be done as clinically indicated at the discretion of the treating physician. During the maintenance phase imaging studies will be done every 8 weeks (-/+ 2 weeks).

7.1.4 Tumor Tissue Collection and Correlative Studies

Planned correlative analyses will include flow cytometric analyses of tumor samples, TIL and peripheral blood mononuclear cells (PBMC), IHC analyses of pre-/post- treatment tumor samples, tumor whole exome/RNA sequencing. Single-cell RNA sequencing will be performed on a subset of treated patients. Studies will be performed under the direction of the Principle Investigator along with co-Investigator Hassane Zarour in Dr. Zarour's lab.

Tumor tissue

Patients must undergo biopsy (e.g. punch or open biopsy) within 21 days (preferred; and up to 4 weeks) of entry to the study (baseline biopsy). Definitive surgery will be performed after 2 weeks of the third dose of nivolumab and any clinically indicated variation from this schedule should be discussed between the investigator, treating sub-investigator and surgical oncologist. Tumor tissue will be processed as described in the lab manual:

Primary melanoma (if available)

FFPE tissue block(s) or 10 unstained slides (air dried) from patients' originally resected primary melanoma will be requested as well.

Blood biospecimens

Blood biospecimens will be collected at baseline, and per the schedule outlined in Study Chart in **Section 6.1**.

At **EACH time point** please submit the following:
- One (1) 10mL RED top tube
- One (1) 8.5mL YELLOW top tube
- Eight (8) 10mL GREEN top tubes
- One (1) 2.5mL PAXgene DNA tube
- One (1) 2.5mL PAXgene RNA tube

At **Baseline and Week 12 ONLY** please submit the following:

- One (1) 2mL BD™ P100.

Each tube must be clearly labeled to include:

- Protocol number
- Patient sequence number

- Patient initials
- Originating institution/investigator name
- Date and time drawn
- Collection time point

<u>Stool specimens</u>
Stool biospecimens will be collected at baseline, and per the schedule outlined in Study Chart in **Section 6.1**.

Dietary questionnaire
<u>DHQ-3 questionnaire may be administered electronically or on paper. Electronic administration is preferred. Data will be entered into DHQ-3 online per instructions as outlined in **Appendix 1**.</u>

7.1.5 Laboratory Procedures/Assessments

Details regarding specific laboratory procedures/assessments to be performed in this trial are provided below. The total amount of blood/tissue to be drawn/collected over the course of the trial (from pre-trial to post-trial visits), including approximate blood/tissue volumes drawn/collected by visit and by sample type per subject can be found in the Procedures Manual.

7.1.5.1 Laboratory safety evaluations

Laboratory tests for hematology, chemistry, urinalysis, and others are specified in **Table 7.1.5.1-1**.

Table 7.1.5.1-1: Laboratory Tests

Hematology	Chemistry	Urinalysis	Other
Hematocrit<br>Hemoglobin<br>Platelet count<br>WBC (total and differential including absolute neutrophil count)	Albumin<br>Alkaline phosphatase<br>Alanine aminotransferase (ALT)<br>Aspartate aminotransferase (AST)<br>Bilirubin (total; directly only if total is elevated above ULN)<br>Total protein<br>Blood urea nitrogen<br>Bicarbonate<br>Calcium<br>Chloride<br>Creatinine<br>Glucose<br>Phosphorus<br>Potassium<br>Magnesium<br>Sodium<br>Lactate dehydrogenase (LDH)<br>Uric acid	pH<br>Specific gravity<br>Glucose<br>Protein<br>Blood<br>Nitrites<br>WBCs<br>Microscopic battery (RBCs, WBCs, epithelial cells, casts) - only if significant positive findings on urinalysis	aPTT<br>PT (INR)<br>Serum or urine pregnancy test (β-human chorionic gonadotropin)[+]<br>Thyroid function studies (thyroid stimulating hormone [TSH], Free T3, Free T4)[#]<br>Autoimmune lab panel (anti-dsDNA, ANA, ANCA, RF, and anti-RNP)<br>HIV[$]<br>Hepatitis B surface antigen (HBsAg) and surface antibody HBsAb) [$]<br>Hepatitis C RNA (HCV RNA)[$]
[+]Perform on women of childbearing potential and at Screening only. If urine pregnancy results cannot be confirmed as negative, serum β-human chorionic gonadotropin will be required.<br>[#]Autoimmune lab panel will be performed at Screening and EOT only.<br>[$]HIV, hepatitis B and C studies will be performed at Screening only in patients as clinically indicated.			

Laboratory tests for screening should be performed within 28 days of registration. After W1D1, pre-dose laboratory procedures can be conducted up to 72 hours prior to dosing at the frequency

delineated in Section 6.1. Results must be reviewed by the investigator or qualified designee and found to be acceptable prior to each dose of trial treatment.

7.1.6 Other Procedures

7.1.6.1 Withdrawal and discontinuation

When a subject discontinues/withdraws prior to trial completion, all applicable activities scheduled for the final trial visit should be performed at the time of discontinuation. Any adverse events which are present at the time of discontinuation/withdrawal should be followed in accordance with the safety requirements outlined in **Section 7.2 - Assessing and Recording Adverse Events**. The schedule for Post-Treatment Visits including Safety Follow-up Visits are delineated in **Section 7.1.7.2**. Following completion of treatment, patients will enter the Follow-Up Period of the study (described in **Section 7.1.7.2.2**).

7.1.6.2 Blinding and unblinding

Not applicable.

7.1.7 Visit Requirements

Visit requirements are outlined in Section 6.0 - Trial Flow Chart. Specific procedure-related details are provided above in Section 7.1 - Trial Procedures.

7.1.7.1 Screening

<u>Screening period</u>

Screening starts with the subject's provision of a written informed consent and should be completed within 21 days (preferred; and up to 4 weeks) of initiation of study drug(s).

7.1.7.2 Post-treatment surveillance

Follow up period will start 12 weeks after the last on-treatment time point for patients have completed study treatments or discontinued study therapy for toxicity and have no disease progression. These patients are evaluated per the standard follow-up schedule including imaging (CT vs. CT/PET vs. PET at the discretion of the treating physician) at the intervals directed below. Every 3 months (±2 weeks) if patient is < 2 years from start of follow up period, every 6 months (±4 weeks) if patient is 2-5 years from start of follow up period, and every 12 months (±4 weeks) if patient is > 5 years from start of follow up period for up to 15 years. Patients who develop recurrent melanoma will be followed for information on survival and for information on salvage patterns. The schedule of clinical follow up for these patients will be at the discretion of the treating physicians and according to established Standard of Care. Adverse Events Assessment on the study will continue for all patients until 30 days after the last study drug administration.

7.1.7.3 Safety follow up visit

The mandatory Safety Follow-Up Visit should be conducted approximately 30 days after the last dose of trial treatment or before the initiation of a new anti-cancer treatment, whichever comes first. All AEs that occur prior to the Safety Follow-Up Visit should be recorded. Subjects with an AE of Grade > 1 will be followed until the resolution of the AE to Grade 0-1 or until the beginning of a new anti-neoplastic therapy, whichever occurs first. SAEs that occur within 90 days of the end of

treatment or before initiation of a new anti-cancer treatment should also be followed and recorded.

7.1.7.4 Follow up visit

Subjects who discontinue trial treatment for a reason other than disease progression will move into the Follow-Up Phase and should be assessed by radiologic imaging to monitor disease status. Every effort should be made to collect information regarding disease status until the start of new anti-neoplastic therapy, disease progression, death, end of the study or if the subject begins retreatment with nivolumab as detailed in **Section 7.1.5.2.1**. Information regarding post-study anti-neoplastic treatment will be collected if new treatment is initiated.

7.1.7.5 Survival follow up

Once a subject experiences confirmed disease progression or starts a new anti-cancer therapy, the subject moves into the survival follow-up phase and should be contacted to assess for survival status until death, withdrawal of consent, or the end of the study, whichever occurs first. This will be done every 3 months.

7.2 Adverse and Serious Adverse Events

7.2.1 Definitions

7.2.1.1 Adverse events (AE)

An AE is an untoward or medical occurrence associated with the use of study drug (active or placebo drug, biologic, or device) in clinical investigation subjects, which does not necessarily have a causal relationship with the study drug. An AE can therefore be any unfavorable and unintended symptom, sign, disease or condition, or test abnormality whether or not considered related to study drug. AEs that do not meet the definition for an SAE are considered non-serious AEs.

AEs include:

- Changes described by the subject or signs observed by the Investigator or medical staff.
- Test abnormalities (i.e., laboratory tests, ECGs) that result in an alteration in medical care (diagnostic or therapeutic).

Disease Progression is **not** considered an AE in this study.

Abnormalities present at baseline are considered AEs only if they reoccur after resolution or they worsen during the study.

7.2.1.2 Serious adverse events (SAE) and serious unexpected adverse events (SUSAR)

An SAE is any AE that fulfills one of the criteria outlined in **Table 7.2.1.2-1**.

Table 7.2.1.2-1: Criteria for Determination of Serious Adverse Events

Death	An AE that results in death.<br>*In this study, deaths that are unequivocally due to Disease Progression are **not** to be reported as SAEs.

Life-threatening AE	An AE that places the subject, in the view of the Investigator, at immediate risk of death from the AE as it occurred (i.e., does not include an AE that had it occurred in a more severe form, might have caused death).
Required or prolonged inpatient hospitalization	An AE that results in an initial inpatient hospitalization or prolongs an existing hospitalization of the subject. If a subject is hospitalized as part of the clinical use of the study drug, a period of normal hospitalization will be outlined in the protocol or by the judgment of the Investigator. Hospitalizations longer than this period will be prolonged hospitalizations.
Persistent or significant disability/incapacity	An AE that results in a substantial disruption of a subject's ability to conduct normal life functions.
Congenital anomaly/birth defect	A congenital anomaly/birth defect that occurs in the offspring of a subject exposed to the study drug.
Important medical event	An AE that may not result in death, be life-threatening, or require hospitalization may be considered an SAE when, based upon appropriate medical judgment, the event may jeopardize the subject and may require medical or surgical intervention to prevent 1 of the outcomes listed above.

Examples of such "important medical events" include allergic bronchospasm requiring intensive treatment in an emergency room or at home, blood dyscrasias or convulsions that do not result in inpatient hospitalization, or the development of drug dependency or drug abuse.

Medical and scientific judgment should be exercised in deciding whether expedited reporting is appropriate in other situations, such as with important medical events described above.

Events that meet SAE criteria must be recorded and reported regardless of expectedness or assessed association with study drug.

Note: Planned hospital admissions or surgical procedures for elective procedures or for an illness or disease that existed before the signing of the ICF or before the subject was enrolled in the study will not be captured as SAEs. If planned admissions or procedures occur at a time other than what was planned (i.e., due to an exacerbation in the preexisting illness or disease), they should be reported as SAEs.

7.2.1.3 Unexpected AE

An unexpected AE is any AE that is not consistent in specificity or severity with the current Investigator's Brochure (IB).

7.2.2 Evaluation of Adverse Events and Serious Adverse Events

The Investigator or designee is responsible for making an assessment as to the severity/grade (as defined in **Section 10.18.2.1**, below), causality/relationship (as defined in **Section 10.18.2.2**, below), and outcome of AEs and SAEs (as defined in **Section 10.18.2.3**, below). In addition, the Investigator or designee must report any actions taken as a result of an AE or SAE.

7.2.2.1 AE severity/grade

For each recorded AE or SAE, the Investigator or designee must make an assessment of Grade using the Common Terminology Criteria for Adverse Events (CTCAE) version 4.03. Grade refers to the severity of the AE. Note that severity is not the same as "seriousness". Seriousness (not severity) serves as a guide for defining regulatory reporting obligations.

7.2.2.2 Causality and relationship to study drug(s)

For each AE or SAE, the Investigator will determine whether there is a reasonable possibility demonstrated by evidence that suggests a causal relationship between the study drug regimen (CMP-001 pus nivolumab), and the AE according to the categories provided in **Table 7.2.2.2-1**. Attribution of adverse events specifically to either CMP-001 or nivolumab is challenging, therefore, the relationship to study drug should be based on attribution to the combination of the two drugs, not a single drug.

An AE with causal relationship not initially determined will require follow-up to assign causality which must be made by the Investigator prior to completion of the study. The Investigator may change his/her opinion of causality in light of follow-up information; if this occurs, the Investigator must amend the AE or SAE information accordingly.

Table 7.2.2.2-1: Classifications for Adverse Event Causality/Relationship

Classification	Definition
Unrelated	There is no suspicion of a causal relationship between exposure to the study drug regimen and the AE; another cause of the AE has been identified, no temporal association with study drug has been identified, or the study drug cannot be implicated.
Possibly related	There is some evidence supporting the possibility of a causal relationship between study drug regimen exposure and the AE; an alternative explanation (i.e., concomitant drug or concomitant disease) is inconclusive, the temporal association with study drug is reasonable, and the causal relationship cannot be excluded.
Probably related	An adverse event that has a timely relationship to the administration of the investigational drug regimen and follows a known pattern of response, but for which a potential alternative cause may be present.
Definitely related	There is strong evidence that there is a causal relationship between study drug regimen and the AE; the AE cannot be reasonably explained by an alternative explanation (i.e., concomitant drug or concomitant disease) and the temporal association with study drug is suggestive of a causal relationship.

An AE is considered related to treatment if the attribution is “possibly related”, “probably related”, or “definitely related.”

7.2.2.3 Classification of AE outcome

Adverse event outcome describes the status of the AE at the last observation. The Investigator will document the outcome of each AE or SAE using the categories provided in **Table 7.2.2.3-1**.

Table 7.2.2.3-1: Classifications for Adverse Event Outcome

Classification	Definition
Fatal	Termination of life as a result of an AE.
Not recovered/not resolved	Subject has not recuperated or the AE has not improved.
Recovering/resolving	Subject is recuperating or the AE is improving.
Recovered/resolved	Subject has recuperated, the AE resolved, or returned to baseline status / stabilized.
Recovered/resolved with sequelae	Adverse event has resolved, but the subject has been left with symptoms or pathology.
Unknown	Not known, not observed, not recorded, or refused.

7.2.2.4 Action taken regarding AE

The Investigator will provide the action taken regarding study drug in response to the AE. Classifications for each of the potential actions taken are provided in **Table 7.2.2.4-1**. More than one option may apply to a single AE/SAE. For example, study drug may be delayed and the dose reduced in response to an AE. Action related to CMP-001 and nivolumab will be assessed and recorded separately in the EDC.

Table 7.2.2.4-1: Classifications for Actions Taken Regarding an Adverse Event

Classification	Definition
Dose not changed	No change in administration of study drug
Dose reduced[1]	Reduction in the amount of study drug administered
Study drug interrupted	Temporary interruption (termination) in administration of the study drug
Study drug withdrawn	Administration of the study drug terminated (no further dosing)
Not applicable	Determination of a value is not relevant in the current context
Unknown	Not known, not observed, not recorded, or refused
[1]Refer to Section 5.2.1. and 5.2.3. regarding dose reductions of either Nivolumab or CMP-001 due to dose-limiting toxicities.	

7.2.3 Timeframe for Adverse Event/Serious Adverse Event Collection

The Investigator is required to record all AEs occurring during the clinical study (21 CFR 312.64[b] and ICH E6 [R1]) starting from the time the subject signs the ICF until 30 days after the last dose of CMP-001 on the AE page of the eCRF. SAEs that occur within 90 days of the end of treatment or before initiation of a new anti-cancer treatment should also be followed and recorded. SAEs as defined in Section 7.2.1.2. must also be reported to the Sponsor or its representative within 24 hours of knowledge of their occurrence, in accordance with Section 7.2.5.

7.2.3.1 Treatment-related AE

Treatment-related AE/SAE is defined as an AE that started or worsened in severity on or after the date the study drug was first administered. TEAE information will be collected from the time of the subject's first receipt of CMP-001 until 30 days after the last dose of CMP-001.

7.2.3.2 SAE

SAE information will be collected from the point the subject starts study treatment until 30 days after the last dose of CMP-001. If, at any time after the subject has completed participation in the study, the Investigator or study staff becomes aware of an SAE that they believe is possibly related or related to CMP-001 (see Section **7.2.1.2**), then the event and any known details must be reported promptly to the Sponsor or its designee. The reporting instructions described in **Section 7.2.5** must be followed.

7.2.4 Recording Adverse Events/Serious Adverse Events

All AEs and SAEs experienced by a subject will be recorded on the appropriate eCRF. Information including a concise description of the event; date and time of event onset and resolution; determination of seriousness, severity, corrective treatment, outcome, relationship to study drug; and action taken regarding the study drug will be recorded. Vital signs, laboratory results, and other safety

assessments noted in **Section 7.1.2** will be recorded as AEs if they are determined to be clinically significant findings in the opinion of the Investigator. When possible, a diagnosis should be recorded as an AE, rather than symptoms or isolated laboratory abnormalities related to that diagnosis. However, when flu-like or cytokine release-like symptoms are reported, each individual symptom should be recorded as a separate AE in the Electronic Data Capture (EDC) system. A medical or surgical procedure is not an AE; rather the condition leading to the procedure should be recorded as the AE. If the condition is not known, the procedure must be recorded as an AE instead. Similarly, death is not an AE, but is rather the outcome of the AE(s) that resulted in death. If the AE(s) leading to death are not known, then death must be reported as an AE.

All SAEs experienced by the subject will be entered into the CTMA and reported to the Sponsor or designee, in accordance with Section **7.2.5.1**.

7.2.5 Reporting Serious Adverse Events and Serious Unexpected Adverse Events

7.2.5.1 SAE and SUAE Reporting

All events meeting the definition of a serious adverse event should be recorded on a MedWatch 3500AForm (http://www.fda.gov/downloads/AboutFDA/ReportsManualsForms/Forms/UCM048334.pdf) or departmental SAE form. Copies should be sent to:

- Principle Investigator (and Sponsor)
- The Checkmate Drug Safety contact information is available for SAE reporting on a 24-hour basis and is reviewed during normal business hours. The contact information is as follows:
  Email: cfrench@checkmatepharma.com
- The Bristol-Myers Squibb Drug Safety contact information is available for SAE reporting on a 24-hour basis and is reviewed during normal business hours. The contact information is as follows:
  Email: Worldwide.Safety@BMS.com
- UPMC Institutional Review Board per institutional reporting requirements
- In addition to completing appropriate patient demographic and suspect medication information, the report should include as applicable the following information that is available at the time of report within the Event Description (section 5) of the MedWatch 3500A form or departmental SAE form:
  - CTCAE term(s) and grade(s)
  - Current status of study drug
  - Intervention(s) to address the AE (testing and result, treatment and response)
  - Hospitalization and/or discharge dates
  - Event relationship to study drug combination

7.2.5.2 Events of Clinical Interest

Selected non-serious and serious adverse events are also known as Events of Clinical Interest (ECI) and must be reported within 24 hours to the Sponsor and within 2 working days to Checkmate Drug Safety Contact (see **Section 7.2.5.1.**).

For the time period beginning when the consent form is signed until treatment initiation, any ECI, or follow up to an ECI, that occurs to any subject must be reported within 24 hours to the Sponsor and within 2 working days to Checkmate Drug Safety Contact if it causes the subject to be excluded from the trial, or is the result of a protocol-specified intervention, including but not limited to washout or discontinuation of usual therapy, diet, placebo treatment or a procedure.

For the time period beginning at treatment initiation through 90 days following cessation of treatment, or 30 days following cessation of treatment if the subject initiates new anticancer therapy, whichever is earlier, any ECI, or follow up to an ECI, whether or not related to Checkmate product, must be reported within 24 hours to the Sponsor and within 24 hours to Checkmate Drug Safety Contact.

Events of clinical interest for this trial include:

- An overdose of Checkmate product
- Cutaneous adverse events at the injection site

7.2.5.3 Pregnancies

Female subjects or the partners of male subjects who discover they are pregnant within a year of their last CMP-001 dose will be instructed to notify the Investigator immediately.

If the Investigator learns of a report of pregnancy at any time after signing the ICF, the Investigator must complete a Pregnancy Form and report the pregnancy to Checkmate Pharmaceuticals Drug Safety within 24 hours (following the same reporting process outlined in **Section 7.2.5.1**).

The Investigator will inform the subject that the Sponsor or its designee is required to gather information regarding the course and outcome of a pregnancy that has occurred after exposure to a study drug. The progress of the pregnancy must be followed until the outcome of the pregnancy is known (i.e., delivery, elective termination, or spontaneous abortion). If the pregnancy results in the birth of a child, additional follow-up information may be requested.

The Investigator will be asked to obtain follow-up information no later than 2 months after the gestational period to obtain maternal/fetal/neonatal outcome and any other relevant information.

Follow-up information may be requested at additional time points. All study-related contacts involving a known pregnancy should include pregnancy status assessment until pregnancy outcome is known.

Please note that pregnancy in and of itself is not an AE or an SAE. Pregnancy should not be entered into the eCRF as an AE unless the Investigator suspects an interaction between the study drug and the contraceptive method. Additionally, all information received will be assessed for any AEs and SAEs and processed per study guidelines. If the subject is discontinued because of pregnancy, pregnancy will be documented as the reason for study discontinuation. Spontaneous abortions and stillbirths will be reported as SAEs.

7.2.6 IND Safety Report

The sponsor must notify FDA and all participating investigators (i.e., all investigators to whom the sponsor is providing drug under its INDs or under any investigator's IND) in an IND safety report of potential serious risks, from clinical trials or any other source, as soon as possible, but in no case later than 15 calendar days after the sponsor determines that the information qualifies for reporting under **Sections 7.2.6.1. to 7.2.6.3.** below. In each IND safety report, the sponsor must identify all IND safety reports previously submitted to FDA concerning a similar suspected adverse reaction, and must analyze the significance of the suspected adverse reaction in light of previous, similar reports or any other relevant information.

7.2.6.1 Serious and unexpected adverse reaction

The sponsor must report any suspected adverse reaction that is both serious and unexpected. The sponsor must report an adverse event as a suspected adverse reaction only if there is evidence to suggest a causal relationship between the drug and the adverse event, such as:

- A single occurrence of an event that is uncommon and known to be strongly associated with drug exposure (e.g., angioedema, hepatic injury, Stevens-Johnson Syndrome);
- One or more occurrences of an event that is not commonly associated with drug exposure, but is otherwise uncommon in the population exposed to the drug (e.g., tendon rupture);
- An aggregate analysis of specific events observed in a clinical trial (such as known consequences of the underlying disease or condition under investigation or other events that commonly occur in the study population independent of drug therapy) that indicates those events occur more frequently in the drug treatment group than in a concurrent or historical control group.

7.2.6.2 Findings from other studies

The sponsor must report any findings from epidemiological studies, pooled analysis of multiple studies, or clinical studies (other than those reported under section 7.2.6.1), whether or not conducted under an IND, and whether or not conducted by the sponsor, that suggest a significant risk in humans exposed to the drug. Ordinarily, such a finding would result in a safety-related change in the protocol, informed consent, investigator brochure (excluding routine updates of these documents), or other aspects of the overall conduct of the clinical investigation.

7.2.6.3 Findings from animal or *in vitro* testing

The sponsor must report any findings from animal or in vitro testing, whether or not conducted by the sponsor, that suggest a significant risk in humans exposed to the drug, such as reports of mutagenicity, teratogenicity, or carcinogenicity, or reports of significant organ toxicity at or near the expected human exposure. Ordinarily, any such findings would result in a safety-related change in the protocol, informed consent, investigator brochure (excluding routine updates of these documents), or other aspects of the overall conduct of the clinical investigation.

7.2.6.4 Increase rate of occurrence of serious suspected adverse reactions

The sponsor must report any clinically important increase in the rate of a serious suspected adverse reaction over that listed in the protocol or investigator brochure.

7.2.6.5 Submission of IND safety reports

The sponsor must submit each IND safety report in a narrative format or on FDA Form 3500A or in an electronic format that FDA can process, review, and archive. FDA will periodically issue guidance on how to provide the electronic submission (e.g., method of transmission, media, file formats, preparation and organization of files). The sponsor may submit foreign suspected adverse reactions on a Council for International Organizations of Medical Sciences (CIOMS) I Form instead of a FDA Form 3500A. Reports of overall findings or pooled analyses from published and unpublished in vitro, animal, epidemiological, or clinical studies must be submitted in a narrative format. Each notification to FDA must bear prominent identification of its contents, i.e., "IND Safety Report," and must be transmitted to the review division in the Center for Drug Evaluation and Research or in the Center for Biologics Evaluation and Research that has responsibility for review of the IND. Upon request from FDA, the sponsor must submit to FDA any additional data or information that the agency deems necessary, as soon as possible, but in no case later than 15 calendar days after receiving the request.

7.2.6.5.1 Unexpected fatal or life-threatining suspected adverse reaction reports

The sponsor must also notify FDA of any unexpected fatal or life-threatening suspected adverse reaction as soon as possible but in no case later than 7 calendar days after the sponsor's initial receipt of the information.

7.2.6.5.2 Reporting format or frequency

FDA may require a sponsor to submit IND safety reports in a format or at a frequency different than that required under this paragraph. The sponsor may also propose and adopt a different reporting format or frequency if the change is agreed to in advance by the director of the FDA review division that has responsibility for review of the IND.

7.2.6.5.3 Investigations of marketed drugs

A sponsor of a clinical study of a drug marketed or approved in the United States that is conducted under an IND is required to submit IND safety reports for suspected adverse reactions that are observed in the clinical study, at domestic or foreign study sites. The sponsor must also submit safety information from the clinical study as prescribed by the post marketing safety reporting requirements.

7.2.6.5.4 Reporting study endpoints

Study endpoints (e.g., mortality or major morbidity) must be reported to FDA by the sponsor as described in the protocol and ordinarily would not be reported under **Section 7.4.6**. However, if a serious and unexpected adverse event occurs for which there is evidence suggesting a causal relationship between the drug and the event (e.g., death from anaphylaxis), the event must be reported under Serious and unexpected suspected adverse reaction as a serious and unexpected suspected adverse reaction even if it is a component of the study endpoint (e.g., all-cause mortality).

7.2.7. Follow-up of Adverse Events/Serious Adverse Events

All AEs and SAEs documented at a previous visit that are designated as either recovering/resolving or not recovered/resolved, will be reviewed by the Investigator at subsequent visits.

All AEs will be followed until resolution of AE, completion of the subject’s participation, or study termination, whichever occurs first.

Serious AEs and AEs resulting in discontinuation will be followed until one of the following occurs:

- The event resolves.
- The event stabilizes.
- The event returns to a baseline value, if a baseline value is available.
- The event can be attributed to agents other than the study drug or to factors unrelated to study conduct.
- The Investigator agrees that follow-up is no longer necessary.

Follow-up reports from the Investigator must be provided as indicated using the SAE report form within **24 hours** of the Investigator’s first knowledge of the new information. Additional information (i.e., hospital records, laboratory, or other diagnostic test results) should be provided if requested and/or indicated.

Rules for AE/SAE follow up apply to all subjects, including those who withdraw consent prior to study completion (to the extent allowed). The Investigator will ensure that follow up includes further investigations to elucidate the nature and/or causality of the AE/SAE. These investigations must be consistent with appropriate medical management and subject consent.

Investigators are not obligated to actively seek AEs or SAEs in former study subjects that occur pursuant to the follow-up period. However, if the Investigator or designee learns of any AE or SAE at any time after a subject has been discharged from the study and the event is considered as reasonably related to the study drug, the Investigator will notify the Sponsor.

8 Statistical Analysis Plan

8.1 Study Design

This is a non-randomized phase II single arm Simon's minimax two-stage study of neoadjuvant nivolumab and CMP-001. Nivolumab (I.V.) and CMP-001 (intra-tumorally) will be administered for 7 weeks pre-operatively (weeks 1-7) followed by planned surgical resection at week 8-10. Following recovery from surgery, nivolumab (I.V.) and CMP-001 (S.C. or peri-tumoral resection site) will commence and continue for 48 weeks. Details for the two-stage design are in **Section 8.3**.

8.2 Safety Monitoring

The PD-1/CMP-001 combination has been tested in the PD-1 refractory setting in melanoma including in a phase I study that evaluated multiple doses of CMP-001 including 5mg, 7.5mg and 10mg. The safety profile observed thus far suggests that the **risk of additive toxicities are unlikely to be significantly increased** in the neoadjuvant setting. Further, the dose being tested in this study (5mg) is 50% the ceiling dose tested in other CMP-001 studies in combination with PD-1 inhibitors, none of which reported a toxicity rate >33%.

However, in the context of this neoadjuvant trial, we will monitor dose limiting toxicities (DLTs) for 4 weeks for the 1st 3 patients accrued. DLT is defined as any adverse event(s) (AEs) considered possibly, probably, or definitely related to the CMP-001 plus nivolumab regimen, which occur during the first 15 days of therapy (i.e. following 3 doses of CMP-001; W1D1, W2D1, W3D1) IN the Prime phase only. Attribution of AEs specifically to either CMP-001 or nivolumab is challenging, therefore, the relationship to CMP-001 should be based on attribution to the combination of the two drugs. During DLT monitoring period, no further accrual will be permitted. Any patient who has started the studied treatment will be evaluable for safety.

The following AEs will be considered DLTs if deemed related to study therapy:

- Hematologic
    - Grade 4 neutropenia
    - Febrile neutropenia, defined as absolute neutrophil count (ANC) ≤1000/mm3 with a temperature of ≥ 38.3 degrees °C
    - Grade ≥ 3 neutropenic infection
    - Grade ≥ 3 thrombocytopenia with bleeding
    - Grade 4 thrombocytopenia
- Non-hematologic
    - Grade ≥ 3 toxicities (non-laboratory)
    - Grade ≥ 3 nausea, vomiting or diarrhea despite maximal medical intervention
    - Grade 4 aspartate aminotransferase (AST) and alanine aminotransferase (ALT)

- Other (non AST/ALT) non-hematologic Grade ≥ 3 laboratory value if the abnormality leads to overnight hospitalization

IMPORTANT: CMP-001 is designed to specifically bind to Toll like receptor 9 (TLR9) which is expressed in the endosomes of pDCs. Upon activation through TLR9, pDCs will secrete large amounts of type 1 interferons and associated Th1-promoting cytokines. Given this mechanism of action, it is expected that the adverse event profile associated with intratumoral injection of CMP-001 may be similar to that seen after administration of interferons (e.g. flu-like symptoms such as fever, rigors, nausea & vomiting). In addition, given the release of IFN-inducible chemokines and cytokines, symptoms associated with cytokine release, such as chills/rigors, fever, nausea/vomiting and hypotension, may occur within hours of a CMP-001 intratumoral injection. Recommended prophylaxis, outlined above in **Section 5.2.2.1.** may help to minimize the severity of these reactions.

An AE believed to be caused by tumor flare or pseudoprogression does not necessarily need to be considered a DLT. Any such cases that would otherwise meet DLT criteria must be discussed immediately with the Principle Investigator. If the Principle Investigator and Sponsor agree that tumor flare is a likely explanation for the AE, treatment with CMP-001 may continue so long as the subject is closely monitored by the Principle Investigator or study staff while on study.

Subjects who experience a DLT will have CMP-001 and nivolumab dosing withheld until the toxicity has returned to ≤ Grade 1. If the Investigator determines that continued treatment with CMP-001 is in the subject's best interest, treatment may resume at a reduced dose (2.5mg weekly or every 4 weeks; 1 dose level lower) at the same schedule after consultation with the Principle Investigator. If a CMP-001 injection is delayed due to toxicity it should be given as soon as possible once the toxicity has resolved. If a subject experiences DLT on a reduced dose of CMP-001, further accrual will be halted.

Subjects may remain on the same dose of nivolumab. Any dose modification should follow the approved nivolumab label.

The Investigator may escalate the subject back to the original CMP-001 dose level if: 1) the lower dose level was tolerated without any DLTs; 2) Sponsor approval is given.

8.3 Sample Size

Several of the agents being utilized in the melanoma armamentarium have been evaluated in the neoadjuvant context including: HDI (Moschos et al. 2006), ipilimumab (Tarhini et al. 2014), pembrolizumab (Forde et al. 2018) and nivolumab/ipilimumab combination (Blank et al. 2018; Amaria et al. 2018). In earlier studies, response was typically assessed using chemotherapy criteria and reported as pathologic complete response (pCR) rates. Cognizant of the different mechanism of action of immuno-oncologic agents, more recent studies have utilized alternative means of assessing response using novel immuno-oncologic agent-specific criteria (major pathologic response or MPR rate) as delineated in **Section 8.4**. In the latter studies, MPR rates reported with PD-1 inhibitors range from 28% (Nivolumab, melanoma) to 45% (Nivolumab/Ipilimumab, melanoma) (Huang et al. 2019; Blank et al. 2018; Amaria et al. 2018).

However, the PD-1/CMP-001 combination has not been evaluated in the neoadjuvant setting and the MPR rate for this combination is unknown. To balance efficacy while minimizing exposure of patients to a potentially ineffective combination, we utilize Simon's minimax two-stage design with MPR as the primary endpoint.

In this study, we will test the null hypothesis of 28% MPR versus the alternative hypothesis of 55% MPR. In the 1st stage, we will enroll 14 evaluable patients (defined in **Section 5.8**). If we see at least 5/14 major pathologic responses, we will enroll a further 16 patients to the 2nd stage. The combination will be considered worthy of further evaluation if ≥13 responses are observed in both stages in 30 evaluable patients. The Simon's minimax two-stage design and sample size of 30 evaluable patients provides a type I error rate of 5%, with a power of 0.91. Allowing for 10% drop out rate, we set a total sample size of 33 patients.

8.4 Efficacy Analysis

Definition of evaluable patient
See **Section 5.8**.

Determination of residual volume of tumor (RVT)
Early neoadjuvant studies in melanoma reported pCR utilizing criteria to define the residual volume of tumor (RVT) derived from neoadjuvant studies of chemotherapy in select tumors including lung cancer (Hellmann et al. 2014)

Neoadjuvant use of immuno-oncologic agents results in distinct histopathologic features compared to neoadjuvant chemotherapy. Specifically, neoadjuvant PD-1 therapy produces unusual features beyond CD8+ T cell infiltrate and tumor necrosis previously associated with response (Tumeh et al. 2014) including features in areas adjacent to the actual tumor – a location termed "the regression bed". Histopathologic features associated with response herein include neovascularization; proliferative fibrosis; presence of cholesterol clefts; development of tertiary lymphoid structures; presence of plasma cells, giant cells, foamy macrophages and granulomas (Cottrell et al. 2018; Tetzlaff et al. 2018).

This observations have led investigators to propose new criteria – immune related pathologic response criteria (irPRC) – wherein immune related RVT (% irRVT) is defined as the total surface area of RVT divided by the total tumor bed area (comprising regression bed area + RVT area + areas of necrosis) (Cottrell et al. 2018; Tetzlaff et al. 2018). Compared to standard methods, irPRC had lower inter-observer variability better correlated with imaging assessments. The neoadjuvant administration of PD-1 and/or CTLA-4 inhibitors is associated with high PRR; ranging from 25-30% pCR (nivolumab monotherapy) to 45% pCR (ipilimumab/nivolumab combination) (Amaria et al. 2018; Blank et al. 2018). Primary endpoint of this study is MPR rate, where MPR is defined as ≤10% RVT remaining in post-therapy specimen using immunotherapy-specific criteria based on residual volume of tumor (RVT) (Cottrell et al. 2018; Tetzlaff et al. 2018).

Residual tumor will be determined in each resection specimen as outlined in **Appendix 2**. All tumors will be macroscopically localized after correlation with radiologic findings. Specimens were sectioned in 5 mm-thick slices. In patients with multiple metastases, each lesion will be extensively sampled from the center to the periphery to include multiple sections of tumor and non-neoplastic parenchyma. Sampling of the tumor nodules in the pathology suite will be directed at the most viable section of the tumor nodule. More than one section per centimeter will be reviewed from each tumor nodule. The number of hematoxylin-eosin slides reviewed per tumor nodule will be at least four (range, 1 to 14 slides). Hematoxylin-eosin stained sections will be reviewed by two melanoma pathologists blinded with respect to clinical information and treatment regimen.

Definition of major pathologic response (MPR)
MPR is defined as ≤10% RVT remaining in post-therapy specimen using immunotherapy-specific pathologic response criteria (irRPRC) (Cottrell et al. 2018; Tetzlaff et al. 2018). MPR rate will be

calculated along with its exact 95% confidence interval. Other categories of response based on %RVT are given below:

- Pathologic complete response (pCR): 0% RVT
- Major pathologic response (irMPR): ≤10% RVT
- Partial pathologic response: <10% but ≤50% RVT
- Pathologic non-response: >50% RVT.

Definition of other endpoints

RFS is defined as the time from initiation of treatment till melanoma relapse or death. All suspected relapse should be biopsied to confirm relapse. In the event where this is difficult and/or dangerous, imaging may be used as a surrogate. OS is defined as the time from initiation of treatment till death. Kaplan-Meier estimates of RFS and OS will be provided. The corresponding median survival time (with 95% confidence intervals) will be determined, along with survival estimates at selected time points (e.g. 6 months, 1 years, and 2 years).

8.5 Safety Analysis

As per NCI CTCAE Version 4.0, the term toxicity is defined as adverse events that are classified as either possibly, probably, or definitely related to study treatment. The maximum grade for each type of toxicity will be recorded for each patient, and frequency tables will be reviewed to determine toxicity patterns.

As CMP-001 has previously been dose-escalated in combination with PD-1 inhibitors pembrolizumab and nivolumab with minimal additional toxicities, no “dose-escalation” will be performed in this study. However, to accurately capture toxicities for this novel combination, we will be monitoring toxicities closely. The detailed data and safety monitoring plan is in **Section 8.7**.

8.6 Biomarker Analysis

To search for potential prognostic biomarkers (and toxicity marker) for the investigation regimen, logistic regression will be used to assess the association between each marker and clinical response (and occurrence of SAEs). The Cox proportional hazards model will be used to assess the association of each marker and survival endpoints (i.e. RFS and OS).

8.7 Data Safety and Monitoring Plan

All enrolled patients will be reviewed weekly to discuss AEs, in particular during DLT period.

Principle Investigator/Sub-investigators, regulatory, CRS management, clinical research coordinators, clinical research associates, data managers, and clinic staff meet monthly in disease center Data Safety Monitoring Boards (DSMB) to review and discuss study data to include, but not limited to, the following:

- serious adverse events
- subject safety issues
- recruitment issues
- accrual
- protocol deviations
- unanticipated problems
- breaches of confidentiality

All toxicities encountered during the study will be evaluated on an ongoing basis according to the NCI Common Toxicity Criteria version 4.0. All study treatment associated adverse events that are serious, at least possibly related and unexpected will be reported to the IRB. Any modifications necessary to ensure subject safety and decisions to continue, or close the trial to accrual are also discussed during these meetings. If any literature becomes available which changes the risk/benefit ratio or suggests that conducting the trial is no longer ethical, the IRB will be notified in the form of an Unanticipated Problem submission and the study may be terminated.

All study data reviewed and discussed during these meetings will be kept confidential. Any breach in subject confidentiality will be reported to the IRB in the form of an Unanticipated Problem submission. The summaries of these meetings are forwarded to the UPCI DSMC which also meets monthly following a designated format.

For all research protocols, there will be a commitment to comply with the IRB's policies for reporting unanticipated problems involving risk to subjects or others (including adverse events). DSMC progress reports, to include a summary of all serious adverse events and modifications, and approval will be submitted to the IRB at the time of renewal.

Protocols with subjects in long-term (survival) follow-up or protocols in data analysis only, will be reviewed twice a year rather than monthly by the disease center DSMB.

Both the UPCI DSMC as well as the individual disease center DSMB have the authority to suspend accrual or further investigate treatment on any trial based on information discussed at these meetings.

All records related to this research study will be stored in a double locked environment. Only the researchers affiliated with the research study and their staff will have access to the research records.

9 Labeling, Packaging, Storage and Return of Clinical Supplies

9.1 Investigational Product

The investigator shall take responsibility for and shall take all steps to maintain appropriate records and ensure appropriate supply, storage, handling, distribution and usage of investigational product in accordance with the protocol and any applicable laws and regulations.

Clinical supplies provided by Checkmate are summarized in **Table 9.1-1**.

Table 9.1-1: Product Descriptions

Product Name and Potency	Dosage Form
CMP-001	Working concentration: 5.0 mg/mL.<br>CMP-001 is a clear, colorless to pale yellow/brown liquid solution, essentially free of particles. Each single use vial contains 1.0 mL of CMP-001 (extractable volume of 1.0 mL = 5.0 mg dose) with a 0.1 mL overfill.

9.2 Packaging and Labeling Information

The CMP-001drug product vials will be labeled with the following information:

- The protocol number;
- The kit number;
- The batch number of the drug;
- The drug name, concentration, and nominal volume per vial;
- The recommended storage conditions of the drug;
- Cautionary statement to keep away from children;
- The route of administration;
- Cautionary statement indicating that the drug is for investigational use only;
- The name and address of the Sponsor;

The CMP-001 kit carton will be labeled with the following information:

- The protocol number;
- The kit number;
- Number of vials per kit;
- The batch number of the drug;
- The drug name, concentration, and nominal volume per vial;
- The recommended storage conditions of the drug;
- Cautionary statement to keep away from children;
- The route of administration;
- Cautionary statement indicating that the drug is for investigational use only;
- The name and address of the Sponsor;

9.3 Handling, Storage and Accountability

All CMP-001 drug product vials will be transported, received, stored, and handled in accordance with the container or drug product kit/vial label, the instructions supplied to the site and its designated pharmacy personnel, the site's standard operating procedures (SOPs), and applicable regulations.

Appropriate storage and transportation conditions will be maintained for the CMP-001 drug product vials from the point of manufacture up to delivery of CMP-001. All shipments of CMP-001 drug product vials will include a temperature monitoring device that records required storage conditions for the vials, at regular intervals for the entire time the shipment is in transit.

Upon receipt by the site, the designated site personnel will examine the shipment and temperature monitoring devices to verify the CMP-001 drug product vials were received in acceptable condition. If not received in acceptable condition, the site must notify the CRA and the site should quarantine the drug until a decision has been made by Checkmate. Once inspected, vials should be stored at the specified temperature (2°C to 8°C) in a locked area accessible only to designated site personnel until dispensing. Once dispensed, CMP-001 drug product vials will be stored in a limited access area under appropriate environmental conditions.

The designated site personnel will be responsible for maintaining accurate records of the quantity and dates of all study drug supplies received, dispensed, and returned, in accordance with applicable regulations and the site's SOPs. The quantity of study drug lost, destroyed, etc. must also be accounted for and documented.

All original vials, whether empty or containing CMP-001 will be kept at the site and destroyed according to the site's drug destruction standard operating procedures. Used CMP-001 vials will not be dispensed again (even to the same subject) nor will they be relabeled or reassigned for use by other subjects. Contents of the CMP-001 drug product vials will not be combined. At the termination

of the study, a final drug accountability review and reconciliation must be completed and any discrepancies must be investigated and their resolution documented.

All CMP-001 drug product vials will be destroyed onsite as per institutional standard operating procedures, after site close out has been completed.

9.4 Dispensing

The CMP-001 drug product vials will be dispensed and CMP-001 drug product will be administered according to applicable site SOPs. Details regarding the preparation and administration of the study drug will be outlined in a study-specific pharmacy manual. Only eligible subjects participating in the study will receive CMP-001. Only authorized and qualified site staff may supply or administer CMP-001.

List of References

Amaria, R.N., Reddy, S.M., Tawbi, H.A., Davies, M.A., Ross, M.I., Glitza, I.C., Cormier, J.N., Lewis, C., Hwu, W.-J., Hanna, E., Diab, A., Wong, M.K., Royal, R., Gross, N., Weber, R., Lai, S.Y., Ehlers, R., Blando, J., Milton, D.R., Woodman, S., Kageyama, R., Wells, D.K., Hwu, P., Patel, S.P., Lucci, A., Hessel, A., Lee, J.E., Gershenwald, J., Simpson, L., Burton, E.M., Posada, L., Haydu, L., Wang, L., Zhang, S., Lazar, A.J., Hudgens, C.W., Gopalakrishnan, V., Reuben, A., Andrews, M.C., Spencer, C.N., Prieto, V., Sharma, P., Allison, J., Tetzlaff, M.T. and Wargo, J.A. 2018. Neoadjuvant immune checkpoint blockade in high-risk resectable melanoma. *Nature Medicine* 24(11), pp. 1649–1654.

Appay, V., Jandus, C., Voelter, V., Reynard, S., Coupland, S.E., Rimoldi, D., Lienard, D., Guillaume, P., Krieg, A.M., Cerottini, J.-C., Romero, P., Leyvraz, S., Rufer, N. and Speiser, D.E. 2006. New generation vaccine induces effective melanoma-specific CD8+ T cells in the circulation but not in the tumor site. *Journal of Immunology* 177(3), pp. 1670–1678.

Appay, V., Speiser, D.E., Rufer, N., Reynard, S., Barbey, C., Cerottini, J.-C., Leyvraz, S., Pinilla, C. and Romero, P. 2006. Decreased specific CD8+ T cell cross-reactivity of antigen recognition following vaccination with Melan-A peptide. *European Journal of Immunology* 36(7), pp. 1805–1814.

Blank, C.U., Rozeman, E.A., Fanchi, L.F., Sikorska, K., van de Wiel, B., Kvistborg, P., Krijgsman, O., van den Braber, M., Philips, D., Broeks, A., van Thienen, J.V., Mallo, H.A., Adriaansz, S., Meulen, S. Ter, Pronk, L.M., Grijpink-Ongering, L.G., Bruining, A., Gittelman, R.M., Warren, S., van Tinteren, H., Peeper, D.S., Haanen, J.B.A.G., van Akkooi, A.C.J. and Schumacher, T.N. 2018. Neoadjuvant versus adjuvant ipilimumab plus nivolumab in macroscopic stage III melanoma. *Nature Medicine* 24(11), pp. 1655–1661.

Boon, T. and van der Bruggen, P. 1996. Human tumor antigens recognized by T lymphocytes. *The Journal of Experimental Medicine* 183(3), pp. 725–729.

Boon, T., Coulie, P.G., Van den Eynde, B.J. and van der Bruggen, P. 2006. Human T cell responses against melanoma. *Annual Review of Immunology* 24, pp. 175–208.

Boon, T. and Old, L.J. 1997. Cancer Tumor antigens. *Current Opinion in Immunology* 9(5), pp. 681–683.

Brudno, J.N. and Kochenderfer, J.N. 2018. Chimeric antigen receptor T-cell therapies for lymphoma. *Nature Reviews. Clinical Oncology* 15(1), pp. 31–46.

Cottrell, T.R., Thompson, E.D., Forde, P.M., Stein, J.E., Duffield, A.S., Anagnostou, V., Rekhtman, N., Anders, R.A., Cuda, J.D., Illei, P.B., Gabrielson, E., Askin, F.B., Niknafs, N., Smith, K.N., Velez, M.J., Sauter, J.L., Isbell, J.M., Jones, D.R., Battafarano, R.J., Yang, S.C., Danilova, L., Wolchok, J.D., Topalian, S.L., Velculescu, V.E., Pardoll, D.M., Brahmer, J.R., Hellmann, M.D., Chaft, J.E., Cimino-Mathews, A. and Taube, J.M. 2018. Pathologic Features of Response to Neoadjuvant Anti-PD-1 in Resected Non-Small Cell Lung Carcinoma: A Proposal for Quantitative Immune-Related Pathologic Response Criteria (irPRC). *Annals of Oncology* 29(8), pp. 1853–1860.

Demoulin, S., Herfs, M., Delvenne, P. and Hubert, P. 2013. Tumor microenvironment converts plasmacytoid dendritic cells into immunosuppressive/tolerogenic cells: insight into the molecular mechanisms. *Journal of Leukocyte Biology* 93(3), pp. 343–352.

Disis, M.L. 2010. Immune regulation of cancer. *Journal of Clinical Oncology* 28(29), pp. 4531–4538.

Ercolini, A.M., Ladle, B.H., Manning, E.A., Pfannenstiel, L.W., Armstrong, T.D., Machiels, J.-P.H., Bieler, J.G., Emens, L.A., Reilly, R.T. and Jaffee, E.M. 2005. Recruitment of latent pools of high-avidity CD8(+) T cells to the antitumor immune response. *The Journal of Experimental Medicine* 201(10), pp. 1591–1602.

Forde, P.M., Chaft, J.E., Smith, K.N., Anagnostou, V., Cottrell, T.R., Hellmann, M.D., Zahurak, M., Yang, S.C., Jones, D.R., Broderick, S., Battafarano, R.J., Velez, M.J., Rekhtman, N., Olah, Z., Naidoo, J., Marrone, K.A., Verde, F., Guo, H., Zhang, J., Caushi, J.X., Chan, H.Y., Sidhom, J.W., Scharpf, R.B., White, J., Gabrielson, E., Wang, H., Rosner, G.L., Rusch, V., Wolchok, J.D., Merghoub, T., Taube, J.M., Velculescu, V.E., Topalian, S.L., Brahmer, J.R. and Pardoll, D.M. 2018. Neoadjuvant PD-1 Blockade in Resectable Lung Cancer. *The New England Journal of Medicine*.

Fourcade, J., Kudela, P., Andrade Filho, P.A., Janjic, B., Land, S.R., Sander, C., Krieg, A., Donnenberg, A., Shen, H., Kirkwood, J.M. and Zarour, H.M. 2008. Immunization with analog peptide in combination with CpG and montanide expands tumor antigen-specific CD8+ T cells in melanoma patients. *Journal of Immunotherapy* 31(8), pp. 781–791.

Francisco, L.M., Salinas, V.H., Brown, K.E., Vanguri, V.K., Freeman, G.J., Kuchroo, V.K. and Sharpe, A.H. 2009. PD-L1 regulates the development, maintenance, and function of induced regulatory T cells. *The Journal of Experimental Medicine* 206(13), pp. 3015–3029.

Freeman, G.J., Long, A.J., Iwai, Y., Bourque, K., Chernova, T., Nishimura, H., Fitz, L.J., Malenkovich, N., Okazaki, T., Byrne, M.C., Horton, H.F., Fouser, L., Carter, L., Ling, V., Bowman, M.R., Carreno, B.M., Collins, M., Wood, C.R. and Honjo, T. 2000. Engagement of the PD-1 immunoinhibitory receptor by a novel B7 family member leads to negative regulation of lymphocyte activation. *The Journal of Experimental Medicine* 192(7), pp. 1027–1034.

Freshwater, T., Kondic, A., Ahamadi, M., Li, C.H., de Greef, R., de Alwis, D. and Stone, J.A. 2017. Evaluation of dosing strategy for pembrolizumab for oncology indications. *Journal for immunotherapy of cancer* 5, p. 43.

Galon, J., Costes, A., Sanchez-Cabo, F., Kirilovsky, A., Mlecnik, B., Lagorce-Pagès, C., Tosolini, M., Camus, M., Berger, A., Wind, P., Zinzindohoué, F., Bruneval, P., Cugnenc, P.-H., Trajanoski, Z., Fridman, W.-H. and Pagès, F. 2006. Type, density, and location of immune cells within human colorectal tumors predict clinical outcome. *Science* 313(5795), pp. 1960–1964.

Galon, J., Pagès, F., Marincola, F.M., Thurin, M., Trinchieri, G., Fox, B.A., Gajewski, T.F. and Ascierto, P.A. 2012. The immune score as a new possible approach for the classification of cancer. *Journal of Translational Medicine* 10, p. 1.

Hellmann, M.D., Chaft, J.E., William, W.N., Rusch, V., Pisters, K.M.W., Kalhor, N., Pataer, A., Travis, W.D., Swisher, S.G., Kris, M.G. and University of Texas MD Anderson Lung Cancer Collaborative Group 2014. Pathological response after neoadjuvant chemotherapy in resectable non-small-cell lung cancers: proposal for the use of major pathological response as a surrogate endpoint. *The Lancet Oncology* 15(1), pp. e42–50.

Hiraoka, N. 2010. Tumor-infiltrating lymphocytes and hepatocellular carcinoma: molecular biology. *International journal of clinical oncology / Japan Society of Clinical Oncology* 15(6), pp. 544–551.

Hodi, F.S., Butler, M., Oble, D.A., Seiden, M.V., Haluska, F.G., Kruse, A., Macrae, S., Nelson, M., Canning, C., Lowy, I., Korman, A., Lautz, D., Russell, S., Jaklitsch, M.T., Ramaiya, N., Chen, T.C., Neuberg, D., Allison, J.P., Mihm, M.C. and Dranoff, G. 2008. Immunologic and clinical effects of

antibody blockade of cytotoxic T lymphocyte-associated antigen 4 in previously vaccinated cancer patients. *Proceedings of the National Academy of Sciences of the United States of America* 105(8), pp. 3005–3010.

Krieg, A.M. 2007. Development of TLR9 agonists for cancer therapy. *The Journal of Clinical Investigation* 117(5), pp. 1184–1194.

Latchman, Y., Wood, C.R., Chernova, T., Chaudhary, D., Borde, M., Chernova, I., Iwai, Y., Long, A.J., Brown, J.A., Nunes, R., Greenfield, E.A., Bourque, K., Boussiotis, V.A., Carter, L.L., Carreno, B.M., Malenkovich, N., Nishimura, H., Okazaki, T., Honjo, T., Sharpe, A.H. and Freeman, G.J. 2001. PD-L2 is a second ligand for PD-1 and inhibits T cell activation. *Nature Immunology* 2(3), pp. 261–268.

Lee, D.W., Gardner, R., Porter, D.L., Louis, C.U., Ahmed, N., Jensen, M., Grupp, S.A. and Mackall, C.L. 2014. Current concepts in the diagnosis and management of cytokine release syndrome. *Blood* 124(2), pp. 188–195.

Liang, S.C., Latchman, Y.E., Buhlmann, J.E., Tomczak, M.F., Horwitz, B.H., Freeman, G.J. and Sharpe, A.H. 2003. Regulation of PD-1, PD-L1, and PD-L2 expression during normal and autoimmune responses. *European Journal of Immunology* 33(10), pp. 2706–2716.

Loke, P. and Allison, J.P. 2003. PD-L1 and PD-L2 are differentially regulated by Th1 and Th2 cells. *Proceedings of the National Academy of Sciences of the United States of America* 100(9), pp. 5336–5341.

Lombardi, V.C., Khaiboullina, S.F. and Rizvanov, A.A. 2015. Plasmacytoid dendritic cells, a role in neoplastic prevention and progression. *European Journal of Clinical Investigation* 45 Suppl 1, pp. 1–8.

Long, G.V., Tykodi, S.S., Schneider, J.G., Garbe, C., Gravis, G., Rashford, M., Agrawal, S., Grigoryeva, E., Bello, A., Roy, A., Rollin, L. and Zhao, X. 2018. Assessment of nivolumab exposure and clinical safety of 480 mg every 4 weeks flat-dosing schedule in patients with cancer. *Annals of Oncology* 29(11), pp. 2208–2213.

Maude, S.L., Frey, N., Shaw, P.A., Aplenc, R., Barrett, D.M., Bunin, N.J., Chew, A., Gonzalez, V.E., Zheng, Z., Lacey, S.F., Mahnke, Y.D., Melenhorst, J.J., Rheingold, S.R., Shen, A., Teachey, D.T., Levine, B.L., June, C.H., Porter, D.L. and Grupp, S.A. 2014. Chimeric antigen receptor T cells for sustained remissions in leukemia. *The New England Journal of Medicine* 371(16), pp. 1507–1517.

Moschos, S.J., Edington, H.D., Land, S.R., Rao, U.N., Jukic, D., Shipe-Spotloe, J. and Kirkwood, J.M. 2006. Neoadjuvant treatment of regional stage IIIB melanoma with high-dose interferon alfa-2b induces objective tumor regression in association with modulation of tumor infiltrating host cellular immune responses. *Journal of Clinical Oncology* 24(19), pp. 3164–3171.

Murad, Y.M. and Clay, T.M. 2009. CpG oligodeoxynucleotides as TLR9 agonists: therapeutic applications in cancer. *Biodrugs: Clinical Immunotherapeutics, Biopharmaceuticals and Gene Therapy* 23(6), pp. 361–375.

Parry, R.V., Chemnitz, J.M., Frauwirth, K.A., Lanfranco, A.R., Braunstein, I., Kobayashi, S.V., Linsley, P.S., Thompson, C.B. and Riley, J.L. 2005. CTLA-4 and PD-1 receptors inhibit T-cell activation by distinct mechanisms. *Molecular and Cellular Biology* 25(21), pp. 9543–9553.

Ribas, A., Medina, T., Kummar, S., Amin, A., Kalbasi, A., Drabick, J.J., Barve, M., Daniels, G.A., Wong, D.J., Schmidt, E.V., Candia, A.F., Coffman, R.L., Leung, A.C.F. and Janssen, R.S. 2018. SD-101 in Combination with Pembrolizumab in Advanced Melanoma: Results of a Phase Ib, Multicenter Study. *Cancer discovery* 8(10), pp. 1250–1257.

Tanaka, T., Narazaki, M. and Kishimoto, T. 2016. Immunotherapeutic implications of IL-6 blockade for cytokine storm. *Immunotherapy* 8(8), pp. 959–970.

Tarhini, A.A., Edington, H., Butterfield, L.H., Lin, Y., Shuai, Y., Tawbi, H., Sander, C., Yin, Y., Holtzman, M., Johnson, J., Rao, U.N.M. and Kirkwood, J.M. 2014. Immune monitoring of the circulation and the tumor microenvironment in patients with regionally advanced melanoma receiving neoadjuvant ipilimumab. *Plos One* 9(2), p. e87705.

Taube, J.M., Klein, A., Brahmer, J.R., Xu, H., Pan, X., Kim, J.H., Chen, L., Pardoll, D.M., Topalian, S.L. and Anders, R.A. 2014. Association of PD-1, PD-1 ligands, and other features of the tumor immune microenvironment with response to anti-PD-1 therapy. *Clinical Cancer Research* 20(19), pp. 5064–5074.

Tetzlaff, M.T., Messina, J.L., Stein, J.E., Xu, X., Amaria, R.N., Blank, C.U., van de Wiel, B.A., Ferguson, P.M., Rawson, R.V., Ross, M.I., Spillane, A.J., Gershenwald, J.E., Saw, R.P.M., van Akkooi, A.C.J., van Houdt, W.J., Mitchell, T.C., Menzies, A.M., Long, G.V., Wargo, J.A., Davies, M.A., Prieto, V.G., Taube, J.M. and Scolyer, R.A. 2018. Pathological assessment of resection specimens after neoadjuvant therapy for metastatic melanoma. *Annals of Oncology* 29(8), pp. 1861–1868.

Tumeh, P.C., Harview, C.L., Yearley, J.H., Shintaku, I.P., Taylor, E.J.M., Robert, L., Chmielowski, B., Spasic, M., Henry, G., Ciobanu, V., West, A.N., Carmona, M., Kivork, C., Seja, E., Cherry, G., Gutierrez, A.J., Grogan, T.R., Mateus, C., Tomasic, G., Glaspy, J.A., Emerson, R.O., Robins, H., Pierce, R.H., Elashoff, D.A., Robert, C. and Ribas, A. 2014. PD-1 blockade induces responses by inhibiting adaptive immune resistance. *Nature* 515(7528), pp. 568–571.

Wang, S., Campos, J., Gallotta, M., Gong, M., Crain, C., Naik, E., Coffman, R.L. and Guiducci, C. 2016. Intratumoral injection of a CpG oligonucleotide reverts resistance to PD-1 blockade by expanding multifunctional CD8+ T cells. *Proceedings of the National Academy of Sciences of the United States of America* 113(46), pp. E7240–E7249.

Weber, J., Mandala, M., Del Vecchio, M., Gogas, H.J., Arance, A.M., Cowey, C.L., Dalle, S., Schenker, M., Chiarion-Sileni, V., Marquez-Rodas, I., Grob, J.-J., Butler, M.O., Middleton, M.R., Maio, M., Atkinson, V., Queirolo, P., Gonzalez, R., Kudchadkar, R.R., Smylie, M., Meyer, N., Mortier, L., Atkins, M.B., Long, G.V., Bhatia, S., Lebbé, C., Rutkowski, P., Yokota, K., Yamazaki, N., Kim, T.M., de Pril, V., Sabater, J., Qureshi, A., Larkin, J., Ascierto, P.A. and CheckMate 238 Collaborators 2017. Adjuvant Nivolumab versus Ipilimumab in Resected Stage III or IV Melanoma. *The New England Journal of Medicine* 377(19), pp. 1824–1835.

Appendix 1: Dietary History Questionnaire for Microbiome Sampling

Dietary history questionnaire will be administered either on paper or electronically and data entered and stored securely electronically. Instrument will be administered by PI or designee.

Detailed information for study staff:

1. Please obtain patient's username and password from study PI.
2. Please provide instructions (see below) to patients.
3. Patients are to be instructed to provide detailed dietary information either during visit (preferred) or at home.
4. The instrument that will be utilized is the DHQ-3 "past month with portion size" obtained from **https://epi.grants.cancer.gov/dhq3/**

Detailed information for patients:

- Thank you for participating in this study. We are interested in evaluating your dietary history. To do this we are using a validated questionnaire termed the “Diet History Questionnaire (DHQ-3)”.
- This questionnaire takes approximately 30 minutes to complete. You can do this either while receiving your therapy or at home.
- After completing the questionnaire, you will receive a Respondent Nutrition Report. This report shows estimated daily nutrient and food group intakes based on questionnaire responses. Recommended values are only available for some nutrients and food groups.
- Please feel free to discuss this with your study doctor.

Dietary study login URL: https://epi.grants.cancer.gov/dhq3/
Your username (case sensitive): __________
Your study password (case sensitive): __________

Appendix 2: Pathologic Response Assessment (irPRC Criteria)
Study ID:

Background and Demographic Information:
- Sex/Age:

Pre-Treatment Pathology
- Location of tumor
- Nature of tumor:
- Initial pathologic stage:
- Date of initial diagnosis:
- Pre-treatment imaging findings:
- Pre-treatment biopsy:
    - Date:
    - Nature of biopsy:
    - Location/anatomic site:
    - Tumor present/absence:

Neoadjuvant Therapy
- Nature of therapy
- Duration of therapy
- Location of injection

Surgical Resection:
- Date:
- Type of surgery:
- Location/anatomic site:
- Post-treatment imaging findings

Post-Treatment Pathological Response Assessment
- Lymph node or In-transit nodule:
- Number of lymph nodes/nodule:
- Largest positive LN/nodule size:
- Sizes of other lymph nodes/nodules:
- Grossly positive:
- Block number of positive LN/nodules:
- Tissue banked (if any):
- Treatment response evaluation metrics:
    - Size of tumor bed:
    - Gross/microscopy:
        - Percentage of viable tumor:
        - Percentage of stromal fibrosis:
        - Percentage of necrosis:
        - Percentage of tumoral melanosis:
        - Extra-capsular extension
            - Y/N
            - Viable/regressed
- Response assessment (immune related pathologic response rate; based on %RVT):
    - Pathologic NR (pNR; %RVT>50%)
    - Partial PR (pPR; 10%<%RVT<50%)

- Major PR (MPR; %RVT≤10%)
- Pathologic CR (pCR; %RVT=0%)
